# Study on the synthesis of the cyclopenta[*f*]indole core of raputindole A

**DOI:** 10.3762/bjoc.12.36

**Published:** 2016-02-23

**Authors:** Nils Marsch, Mario Kock, Thomas Lindel

**Affiliations:** 1 Institute of Organic Chemistry, TU Braunschweig, Hagenring 30, 38106 Braunschweig, Germany

**Keywords:** gold-catalyzed reactions, heterocycles, indole alkaloids, natural products, synthesis

## Abstract

The raputindoles from the rutaceous tree *Raputia simulans* share a cyclopenta[*f*]indole partial structure the synthesis of which is subject of this investigation. An efficient route to a series of 1,5-di(indol-6-yl)pentenones was developed via Mo/Au-catalyzed Meyer–Schuster rearrangement of tertiary propargylic alcohol precursors. However, none of the enones underwent the desired Nazarov cyclization to a cyclopenta[*f*]indole. More suitable were 6-hydroxyallylated indolines which gave good yields of cyclopenta[*f*]indolines after treatment with SnCl_4_, as soon as sterically demanding β-cyclocitral adducts were reacted. Most successful were Pt(II) and Au(I)-catalyzed cyclizations of *N-*TIPS-protected indolin-6-yl-substituted propargylacetates which provided the hydrogenated tricyclic cyclopenta[*f*]indole core system in high yield.

## Introduction

**Figure 1:**
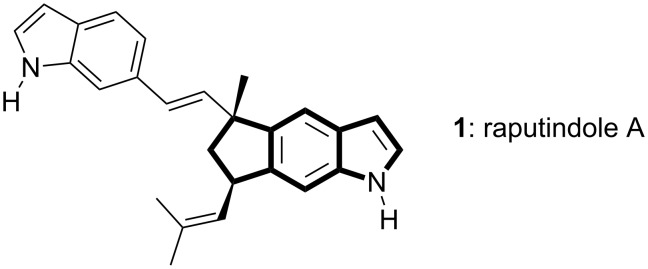
Bisindole alkaloid raputindole A (**1**) from the Amazonian tree *Raputia simulans*.

The raputindoles (**1**, raputindole A, Figure 1) from the rutaceous tree *Raputia simulans* Kallunki constitute a unique group of terpenoid bisindole natural products [1] sharing a linear cyclopenta[*f*]indole tricyclic partial structure. The cyclopenta[*f*]indole system also occurs as partial structure of the nodulisporic acids [2], the shearinines [3–6] and janthitrems [7–10]. While work has been done on the total syntheses of herbindoles [11] and trikentrines [12], which contain angular cyclopenta[*g*]indole partial structures [13–29], there is only little known on the assembly of linear cyclopenta[*f*]indole systems. Both existing approaches rely on the anellation of the pyrrole part to indene-based starting materials [30–33].

**Scheme 1:**
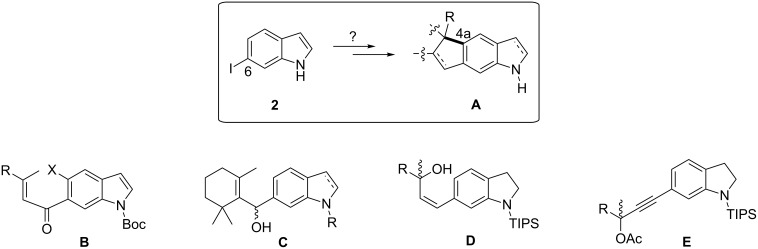
Investigated synthetic precursors **B**–**E** of the cyclopenta[*f*]indole moiety (**A**) of raputindole A (**1**), all to be assembled from 6-iodoindole (**2**).

In this paper we discuss our experiences with the assembly of cyclopenta[*f*]indole and -indoline systems (**A**, Scheme 1). A bond between the indole 5-position (4a in the resulting tricycle) and the quaternary center was to be formed. Ideally, anellation of a cyclopentane would be possible at an indole with an intact enamine partial structure (**B**, Scheme 1). Such an approach seemed possible, because we had already cyclized 6-prenoylindole to a mixture of cyclopenta[*f*]- and -[*g*]indoles in a Nazarov-type reaction [34]. By installation of a triflyloxy group in the indole 5-position, Pd-catalyzed cyclization also would become possible. As an alternative to the cyclization of enones, Lewis acid-induced cyclizations of allylic alcohols could afford the desired cyclopenta[*f*]indole system (**C**, **D**). Here, it was unclear whether the indole enamine section would be tolerated or have to be reduced prior to cyclization. Finally, propargyl alcohol derivatives (**E**) were to be investigated as substrates of Pt(II) and Au(I)-catalyzed reactions. All of the investigated building blocks were to be obtained from 6-iodoindole (**2**), which was synthesized via the Batcho–Leimgruber route and purified by sublimation [34].

## Results and Discussion

**Scheme 2:**
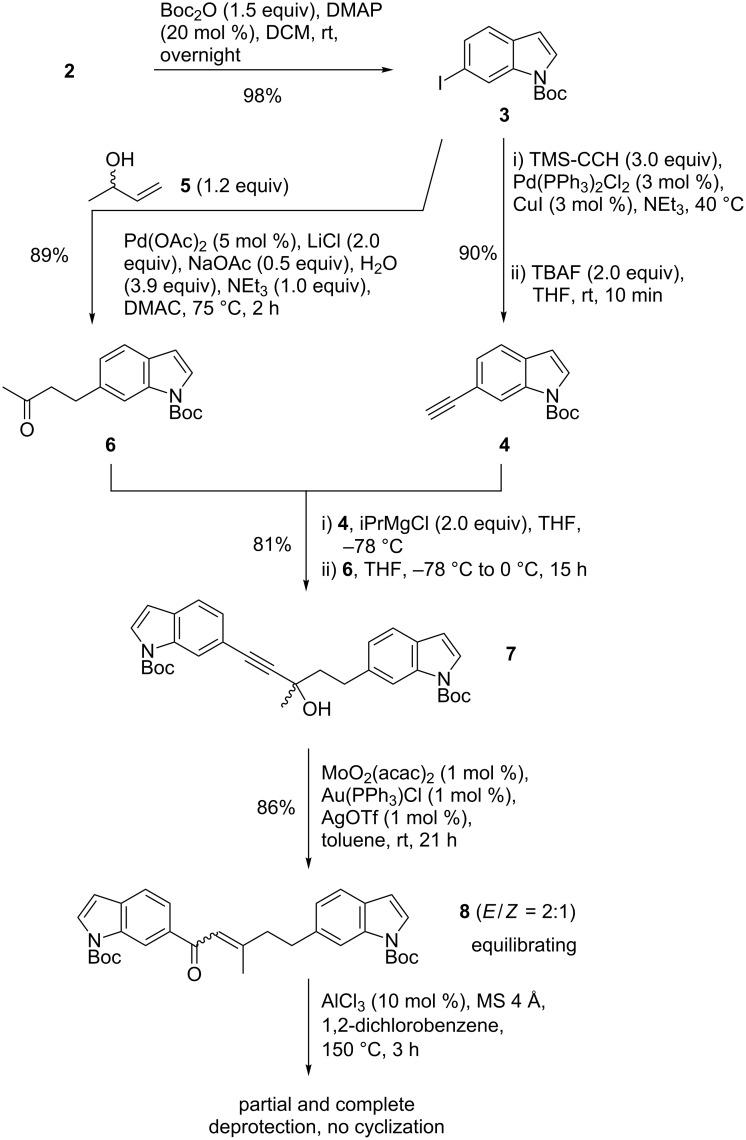
6-Iodoindole (**2**) serves twice as starting material towards indole-6-yl-substituted enone **8**, obtained via Au-catalyzed Meyer–Schuster rearrangement.

**Di(indol-6-yl)pentenones.** Regarding the investigation of 6-acryloylindoles, we aimed at the synthesis of methyl-branched di(indol-6-yl)pentenones from the beginning, which already included the second indole moiety of raputindole A (**1**). Boc protection of 6-iodoindole (**2**) [35], Sonogashira reaction of **3** with TMS-acetylene, and desilylation gave the N-protected alkynylindole **4** in excellent yield (Scheme 2). Boc-iodoindole **3** was also the precursor of the coupling partner, ketone **6**, which was synthesized via Heck reaction with but-3-en-2-ol (**5**, 89%) in the presence of LiCl, inspired by a procedure by Camp and coworkers [36].

Propargyl bisindole **7** was obtained after conversion of **4** (2 equiv) to the magnesium acetylide (iPrMgCl in THF) and Grignard reaction with ketone **6**. Due to the presence of Boc-protected indole nitrogens an acid-catalyzed Meyer–Schuster rearrangement was dismissed. Instead, a transition metal-catalyzed rearrangement employing 1 mol % of MoO_2_(acac)_2_/[Au(PPh_3_)Cl]/AgOTf [37] afforded the α,β-unsaturated ketone **8** as a 2:1 mixture of *E*/*Z* isomers (86%), which could not be separated by HPLC due to a reisomerization upon concentration of the fractions. When bisindole **8** was subjected to AlCl_3_, no cyclization product was observed and only deprotection occurred. This was somewhat surprising, because treatment of 6-prenoylindole with AlCl_3_ in 1,2-dichlorobenzene at 150 °C had induced Nazarov cyclization affording a mixture of regioisomeric cyclopenta[*f*]- and -[*g*]indolones [34]. Kern and coworkers had obtained an indanone under the same conditions [38]. Other attempts to cyclize **8** also failed.

**Scheme 3:**
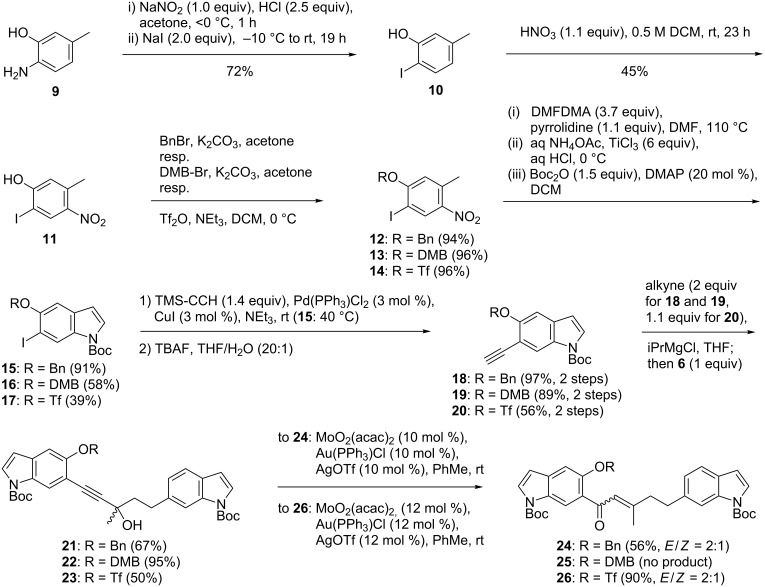
Assembly of 5-oxygenated bisindolylpentenones. DMB: 3,4-dimethoxybenzyl, DMFDMA: *N*,*N*-dimethylformaldehyde dimethyl acetal.

We turned to Pd-catalyzed reductive cyclization. As precursor, a 5-triflyloxyindole was preferred over a 5-bromoindole, because 2-iodo-5-methyl-4-nitrophenol (**11**, Scheme 3), to be used in the Batcho–Leimgruber protocol, appeared to be more readily accessible than 1-bromo-2-iodo-5-methyl-4-nitrobenzene. Moreover, aryl triflates have been used in intramolecular cyclization reactions with α,β-unsaturated ketones to obtain indanones and dihydronaphthones [39–40]. Aminophenol **9** was converted to iodophenol **10** in good yield through a Sandmeyer reaction (Scheme 3) [41]. Various nitration conditions were tested, yet only the use of concentrated HNO_3_ in CH_2_Cl_2_, as reported by Chancellor and coworkers [42], gave nitrophenol **11** in a reasonable yield (45%). The Batcho–Leimgruber protocol failed for nitrophenol **11** [43]. However, after *O*-benzylation of **11** to **12**, Boc-indole **15** was obtained in the very good yield of 91%. Coupling with TMS-acetylene was followed by desilylation to obtain the terminal alkyne **18** (97%). Magnesiation (iPrMgCl, THF) and reaction with ketone **6** afforded the benzyloxy-substituted bisindole **21**. Transition metal-catalyzed Meyer–Schuster rearrangement afforded **24** (56%, *E*/*Z* mixture as above) albeit the catalyst loading had to be increased from 1 to 10 mol %, when compared to the synthesis of **8** (Scheme 2). We also observed elimination of water from **21**. However, it proved to be impossible to debenzylate **24**, which would have been necessary to access the corresponding triflate. For instance, we treated **24** with BCl_3_ in pentamethylbenzene/DCM which was used by Tokuyama and coworkers for benzyl ether cleavage in the presence of Boc-protected amines when studying the late stages of the total synthesis of the trisindole alkaloid yatakemycin [44].

As an alternative, the 3,4-dimethoxybenzyl (DMB) protecting group was installed (**13**). We favored the DMB over the related PMB group, because the oxidation potential of DMB ethers is lower and the cleavage was expected to be more facile [45]. Indole synthesis from DMB-protected **13** and subsequent Boc protection afforded **16** (58%). Alkynylation and desilylation of **16** to **19** and coupling of **19** with ketone **6** proceeded smoothly providing the DMB-protected bisindole **22** in 18% overall yield over seven steps (Scheme 3). However, in the presence of the DMB group, the Meyer–Schuster rearrangement of alkyne **22** to the envisaged ketone **25** failed. Mixtures of elimination products dominated along with the cleavage of the DMB ether. Meyer–Schuster products could also be observed, but in small amounts and not as pure compounds.

**Scheme 4:**
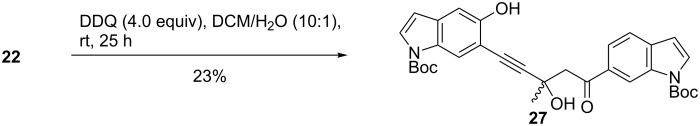
Benzylic oxidation as side reaction of DMB removal.

It is worth mentioning that treatment of **22** with DDQ led to removal of the DMB group, affording the major product **27** (23%) exhibiting a keto group in the benzylic indole position (Scheme 4). This transformation might become useful in a future total synthesis of raputindole A (**1**) as a reduction–elimination sequence of the benzylic ketone could be used to introduce the olefinic double bond.

Since neither from **22** nor from **24** the indole-5-OH group could be liberated, we turned back to the originally dismissed idea of installing the triflate group prior to indole assembly. There are no 5-triflyloxy-6-iodoindoles described in the literature. First, iodonitrophenol **11** was converted to triflate **14** in 96% yield. Subsequent Batcho–Leimgruber synthesis afforded the 6-iodo-5-triflyloxyindole, which was Boc-protected (**17**, 39%, three steps, Scheme 3). Sonogashira coupling occurred preferably at the iodinated 6-position affording 6-alkynylindole **20** after desilylation (56%). The reaction with ketone **6** after magnesiation led to propargylic alcohol **23** (50%). Interestingly, the Meyer–Schuster rearrangement of triflated alkyne **23** to ketone **26** proceeded in the highest yield of all our Meyer–Schuster rearrangements. With triflated α,β-unsaturated ketone **26** in hand, we attempted reductive Heck cyclizations to the raputindole core structure. For instance, we employed Pd(dba)_2_ (12 mol %), QPhos (24 mol %), and NEt_3_ (1.20 equiv) in DMF at 100 °C. Unfortunately, all reactions gave intractably complex mixtures. ^1^H and ^19^F NMR analysis suggested that cleavage of at least one Boc-protecting group had occurred. Additionally, the triflate was still visible in the ^19^F NMR spectrum, thus indicating that oxidative addition to the Pd catalyst had not taken place.

At this point, we abandoned our attempts of assembling the cyclopenta[*f*]indole unit of raputindole A (**1**) starting from Boc-protected indoles. None of the investigated bisindolylpentenones **8**, **24**, or **26** could be cyclized to a cyclopentaindole. At least, we learned how to synthesize the open-chain molecules and also their propargylic precursors.

**Cyclization of allyl cations.** Besides Nazarov and reductive Pd-catalyzed cyclizations there was the possibility of generating an allyl cation which would have to undergo cyclization to the cyclopenta[*f*]indole. There are not many examples of cyclopentene anellation by cyclization of aryl-substituted allyl cations. High yields were reported by Alvarez-Manzaneda et al. in the course of their total synthesis of taiwaniaquinone H. They induced the cyclization by treatment of arylvinylcarbinols with the mild Lewis acid SnCl_4_, which were synthesized by hydroxyalkylation with β-cyclocitral [46].

**Scheme 5:**
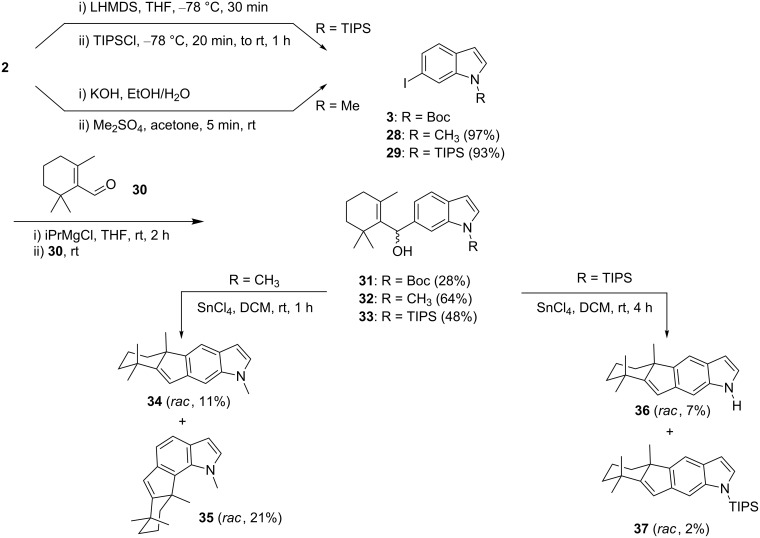
Hydroxyalkylation of N-protected indoles with β-cyclocitral and SnCl_4_-induced cyclization.

Hydroxyalkylation of Boc-protected 6-iodoindole (**3**) with 6-β-cyclocitral (**30**) was possible after iodine/magnesium exchange, affording adduct **31** (Scheme 5). However, treatment of **31** with SnCl_4_ in DCM did not afford any defined product. This changed after replacement of the Boc by a methyl group. The 6-hydroxyalkylated *N*-methylindole **32** (64%) was prepared from 6-iodo-*N-*methylindole (**28**) with β-cyclocitral (**30**). The subsequent treatment of **32** with SnCl_4_ in DCM afforded a 1:2 mixture of regioisomeric indeno[1,2-*f*]indole **34** (11%) and indeno[2,1-*g*]indole **35** (21%).

For the corresponding *N-*TIPS-protected indole **33**, obtained from **29**, yields of tetracyclic products **36** (7%) and **37** (2%) were very low and we observed the loss of the TIPS protecting group. Noteworthy, we did not detect regioisomeric indeno[2,1-*g*]indole products when starting from **33**, which points at a shielding effect of the TIPS group towards the indole 7-position.

**Scheme 6:**
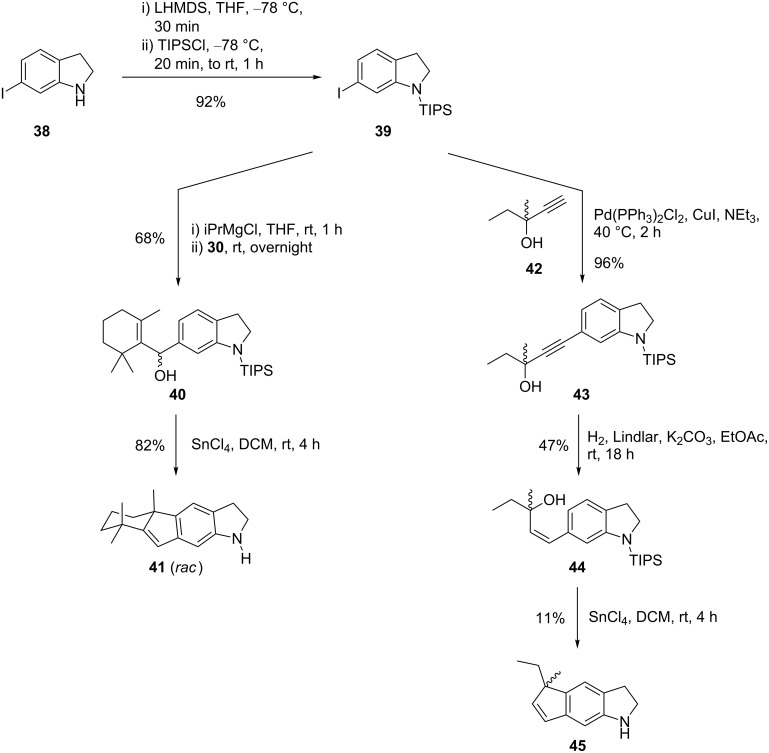
Behavior of indolines after SnCl_4_-induced generation of allyl cations.

Since the SnCl_4_-mediated cyclization yields with indoles were much lower than those obtained with benzene derivatives, we investigated the behavior of indolines which lack the reactive enamine moiety. We also abandoned the use of Boc-protecting groups. Reduction of 6-iodoindole with NaBH_3_CN in HOAc afforded 6-iodoindoline (**38**, 90%) [47], which was subsequently TIPS-protected (**39**, Scheme 6).

Hydroxyalkylation of **39** with β-cyclocitral (**30**) gave cyclization precursor **40** (68%). We were pleased to find that this time the SnCl_4_-induced cyclization afforded the desilylated tetracyclic indeno[1,2-*f*]indoline **41** in high yield (82%). We did not detect any regioisomer, probably because the TIPS group was still in place in the regioselecting step. In order to obtain a tricyclic cyclopentaindoline, the propargylic alcohol **43** (96%) was synthesized by Sonogashira coupling of *N*-TIPS-6-iodoindoline (**39**) and **42**. Conversion of **43** to the (*Z*)-allylic alcohol **44** by modified (K_2_CO_3_) Lindlar hydrogenation (47%) followed. Treatment of **44** with SnCl_4_ in DCM afforded cyclopenta[*f*]indoline **45**, albeit in the rather disappointing yield of 11%.

A reason for the much better yield of β-cyclocitral adduct **41** when compared to **45** may be a conformational restriction of the allyl cation, caused by the geminal methyl groups of **40**. The intermediate is probably kept in the *cisoid* conformation required for the cyclization. Thus, switching from indole to indoline and changing the N-protecting group significantly improved the yield for the cyclocitral adduct, but this still does not appear to be sufficient for a strategy towards raputindole A (**1**).

**Scheme 7:**
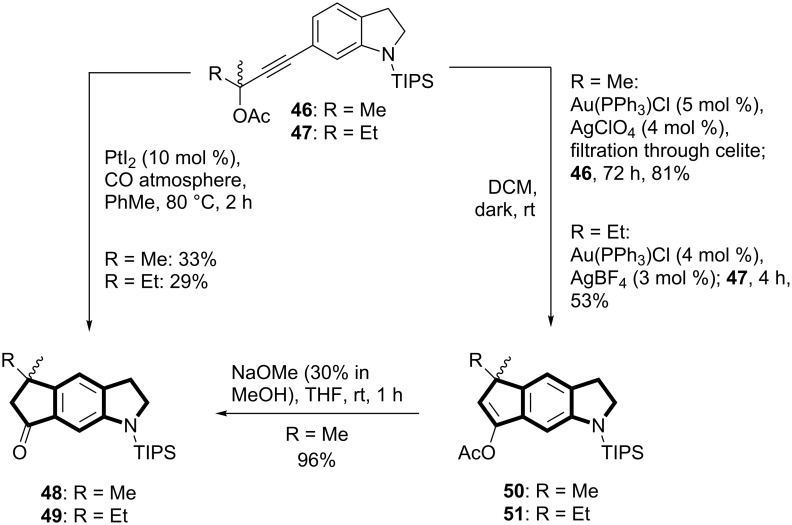
Pt(II) and Au(I)-catalyzed cyclizations of propargylacetates **46** and **47** afforded cyclopenta[*f*]indolinones **48** and **49**, and tricyclic cyclopentenylacetates **50** and **51**.

**Cyclization of propargylacetates.** Key progress came when applying platinum and gold chemistry to propargylacetates **46** and **47** (Scheme 7), which were obtained by Sonogashira alkynylations of *N-*TIPS-6-iodoindoline (**39**), followed by acetylation (for experimental procedures, see Supporting Information File 1). Xuegong She and coworkers had obtained indanone derivatives from arylpropargylic esters in a Pt(II)-catalyzed reaction for which they propose a formal rearrangement of the acetoxy group, followed by cyclization [48]. When we treated **46** and **47** with PtI_2_ (10 mol %) in a CO atmosphere at elevated temperature (PhMe, 80 °C) we indeed obtained the tricyclic cyclopentanones **48** and **49**, respectively. Yields were moderate in both cases (33% and 29%), but already better than in the case of the SnCl_4_-induced cyclization of (*Z*)-allylic alcohol **44** (Scheme 6). We only isolated the cyclopentanones, formed from the corresponding cyclopentenyl acetates. Presumably, the propargylacetate first undergoes a [3,3]-sigmatropic shift to the allenyl acetate, followed by a Pt(II)-catalyzed cyclization to the tricycle.

When propargylacetates **46** and **47** were treated with catalytic amounts of Au(PPh_3_)Cl/AgBF_4_ (DCM, rt, dark), cyclopenta[*f*]indolines **50** and **51** were isolated in even better yields. It proved to be beneficial to filter the catalyst solution through a pad of Celite prior to addition of the starting material to remove AgCl. In that way we reached an 81% yield of tricycle **50**. The acetoxy group again formally underwent a 1,3-shift to the benzylic position, which may also be based on a [3,3]-sigmatropic rearrangement. Alternatively, for a similar 1,3-shift, Nolan and coworkers proposed two sequential 1,2-shifts to occur after π-complexation of the triple bond by Au(I) [49]. Methanolysis (NaOMe/MeOH) of cyclopentenylacetate **50** afforded cyclopenta[*f*]indolinone **48** (96%).

## Conclusion

It was our goal to explore how to efficiently assemble the cyclopenta[*f*]indole section present in the natural product raputindole A (**1**). As long as the cyclopentane ring would not be installed, it was indeed possible to work with 2,3-unsubstituted, *N-*Boc-protected indoles. In particular, the Mo/Au-catalyzed Meyer–Schuster rearrangement of propargylalcohols **7**, **21**, and **23** worked nicely, even in the presence of a triflyloxy group. However, all attempts of cyclization failed with the Boc-protected bisindoles. Two modifications changed things for the better regarding the cyclization: replacement of the Boc- by a non-coordinating TIPS-protecting group and the use of indolines instead of sensitive indoles. Cyclopentane anellation by SnCl_4_-induced cyclization of phenylvinylcarbinols became possible, at least for the sterically congested β-cyclocitral adduct **40** of *N-*TIPS-indoline. The less sterically demanding substrate **44** gave lower yields. Experiments employing Au(I) and Pt(II) catalysts point at how to continue, since tricycles **48**, **49**, and **50** were obtained in good yields. We will now investigate the synthesis and Au(I) and Pt(II)-catalyzed cyclizations of TIPS-protected bisindolines, following a modified retrosynthesis of raputindole A (**1**).

## Supporting Information

File 1 Experimental procedures and NMR spectra.
