# Supplementary material for: Study on the synthesis of the cyclopenta[f]indole core of raputindole A
Source: Beilstein J Org Chem. 2016 Feb 23;12:334–42. doi: 10.3762/bjoc.12.36 (PMC4778506; doi:10.3762/bjoc.12.36)

# Supporting Information

# for

# Study on the synthesis of the cyclopenta[*f*]indole core of raputindole A

Nils Marsch, Mario Kock, and Thomas Lindel*§

Address: Institute of Organic Chemistry, TU Braunschweig, Hagenring 30, 38106 Braunschweig, Germany, Fax: (+49) 531-391-7744 E-mail: th.lindel@tu-braunschweig.de, www.oc.tu-bs.de/lindel

Email: Thomas Lindel - th.lindel@tu-braunschweig.de

§www.oc.tu-bs.de/lindel

* Corresponding author

## Experimental procedures and NMR spectra

**Table of Contents:**

1. General Methods ........ S2

2. Experimental Procedures ........ S3

3. References: ........ S38

4. Spectra ........ S39

## 1. General Methods

NMR spectra were recorded on a Bruker AV II-300 (300 MHz for $^{1}$H, 75 MHz for $^{13}$C), a Bruker DRX 400 or a Bruker AV III-400 (400 MHz for $^{1}$H, 100 MHz for $^{13}$C, 376 MHz for $^{19}$F) and a Bruker AV-II 600 (600 MHz for $^{1}$H, 150 MHz for $^{13}$C) spectrometer at 299 K. Chemical shifts are given in ppm (δ scale) and are referenced to TMS or the solvent residual peak. Mass spectra were obtained with a LTQ Orbitrap Velos, a Thermo Finnigan LTQ FT, a Thermo Finnigan MAT95 and a Finnigan MAT 95 XLT. GC–MS was performed on an Agilent Technologies 6890 gas chromatograph using a Phenomenex ZB5-MS 0.25 μm column (internal diameter: 0.25 mm, length: 30 m) and a JMST100GC (GCAccuTOF, JEOL, Japan) apparatus at 70 eV (EI). IR spectra were recorded on a Bruker Tensor 27 spectrometer, wavenumbers are given in $cm^{-1}$. UV–vis spectra were measured with a Varian Cary 100 Bio UV–vis spectrometer and wavelengths are given in nm. Melting points were measured with a Büchi 530 melting point apparatus and are uncorrected. Chemicals were purchased from commercial suppliers and used without further purification. Solvents were dried prior to use using standard methods unless otherwise noted. Flash column chromatography was performed on Merck silica gel 60 (40–63 μm). Thin layer chromatography (TLC) was done on Merck silica gel 60 $F_{254}$ and Merck silica gel 60 RP-18 $F_{254S}$ aluminum sheets. HPLC separation was carried out with a Merck Hitachi intelligent pump.

## 2. Experimental Procedures

***tert*-Butyl 6-iodo-1*H*-indole-1-carboxylate (3) [S1]:** 6-Iodo-1*H*-indole (**2**, 1.513 g, 6.30 mmol, 1.00 equiv, [S2]) was dissolved in $CH_2Cl_2$ (90 mL). $Boc_2O$ (2.063 g, 9.45 mmol, 1.50 equiv) and DMAP (154 mg, 1.26 mmol, 20 mol %) were added and the mixture was stirred at room temperature overnight. The solvent was evaporated and the crude product was subjected to column chromatography using petroleum ether/EtOAc (15:1) as the eluent to yield *N*-Boc-6-iodoindole (**3**, 2.117 g, 6.17 mmol, 98%) as colorless solid. TLC [petroleum ether/EtOAc (15:1)]: $R_f$ = 0.63. Mp: 77-79 °C. $^1$H NMR (400 MHz, $CDCl_3$): $\delta$ = 8.57 (s, 1H, 7-*H*), 7.51 (dd, 1H, *J* = 8.3 Hz, *J* = 1.5 Hz, 5-*H*), 7.51-7.49 (m, 1H, 2-*H*), 7.29 (d, 1H, *J* = 8.2 Hz, 4-*H*), 6.51 (dd, 1H, *J* = 3.7 Hz, *J* = 0.6 Hz, 3-*H*), 1.67 (s, 9H, C($CH_3$)$_3$). $^{13}$C NMR (100 MHz, $CDCl_3$): $\delta$ = 149.3 (1C, N*C*(=O)), 136.2 (1C, *C*-7a), 131.5 (1C, *C*-5), 129.8 (1C, *C*-3a), 126.1 (1C, *C*-2), 124.3 (1C, *C*-7), 122.3 (1C, *C*-4), 107.1 (1C, *C*-3), 88.6 (1C, *C*I), 84.2 (1C, *C*($CH_3$)$_3$), 28.1 (3C, C(*C*$H_3$)$_3$). IR (diamond ATR): $\tilde{\nu}$ = 3158 (w), 3120 (w), 3067 (w), 3000 (w), 2969 (w), 2928 (w), 1747 (m), 1726 (m), 1598 (w), 1571 (w), 1529 (w), 1456 (w), 1432 (m), 1373 (m), 1340 (s), 1247 (s), 1205 (w), 1155 (s), 1119 (s), 1086 (m), 1042 (w), 1024 (m), 884 (m), 857 (m), 802 (m), 778 (m), 756 (m), 709 (m), 667 (w), 584 (m), 544 (m). UV/Vis (MeOH): $\lambda_{max}$ (log $\varepsilon$) = 231 (4.42), 269 (4.20), 297 (3.43). HRESIMS: calculated ([$C_{13}H_{14}INO_2$+$Na^+$]) 365.99614, found 365.99628 (0.38 ppm).

***tert*-Butyl 6-ethynyl-1*H*-indole-1-carboxylate (4):** *tert*-Butyl 6-iodo-1*H*-indole-1-carboxylate (**3**, 1.50 g, 4.37 mmol, 1.00 equiv), $Pd(PPh_3)_2Cl_2$ (91 mg, 0.13 mmol, 3 mol %) and ethynyltrimethylsilane (650 µL, 4.60 mmol, 1.05 equiv) were degassed under argon for 30 min. Simultaneously, $NEt_3$ (5 mL) was degassed in a separate flask for 10 min and CuI (3 mg, 17.49 mmol, 3 mol %) was added. The CuI was dissolved by sonication for further 5 min. The obtained CuI solution was added to the reaction mixture and heated to 40 °C. At this temperature, another portion of ethynyltrimethylsilane (325 µL, 2.30 mmol, 0.53 equiv) was added and the reaction mixture changed color from yellow to brown. After stirring for further 2 h at this temperature the mixture was filtered through Celite 545. The Celite was washed with EtOAc (50 mL) and the solvent was evaporated. The crude product was dissolved in THF (50 mL) and cooled to 0 °C with an ice bath. A solution of 1 M TBAF in THF with 5% $H_2O$ (8.74 mL, 8.74 mmol, 2.00 equiv) was added and the mixture was stirred for 10 min. The solvent was evaporated and the crude product was subjected to column chromatography using petroleum ether/EtOAc (30:1) as the eluent. The terminal alkyne **4** (910 mg, 3.77 mmol, 86%) was isolated as colorless solid. TLC [petroleum ether/EtOAc (30:1)]: $R_f$ = 0.63. Mp: 60-62 °C. $^1$H NMR (400 MHz, $CDCl_3$): $\delta$ = 8.33 (s, 1H, 7-*H*), 7.64 (d, 1H, *J* = 3.7 Hz, 2-*H*), 7.49 (dd, 1H, *J* = 8.1Hz, *J* = 0.5 Hz, 4-*H*), 7.35 (dd, 1H, *J* = 8.1 Hz, *J* = 1.4 Hz, 5-*H*), 6.55 (dd, 1H, *J* = 3.7 Hz, *J* = 0.6 Hz, 3-*H*), 3.06 (s, 1H, *H*C≡C), 1.68 (s, 9H,

C(C$H_3$)$_3$). $^{13}$C NMR (100 MHz, $CDCl_3$): $\delta$ = 149.4 (1C, NC(=O)), 134.7 (1C, C-7a), 130.9 (1C, C-3a), 127.3 (1C, C-2), 126.6 (1C, C-5), 120.8 (1C, C-4), 119.3 (1C, C-7), 117.6 (1C, C-6), 107.2 (1C, C-3), 84.7 (1C, HC≡C), 84.1 (1C, C($CH_3$)$_3$), 76.2 (1C, HC≡C), 28.2 (3C, C($CH_3$)$_3$). IR (diamond ATR): $\tilde{\nu}$ = 3293 (m), 3158 (w), 3122 (w), 3075 (w), 2978 (w), 2933 (w), 1719 (s), 1525 (w), 1469 (w), 1439 (m), 1381 (w), 1346 (s), 1307 (w), 1268 (m), 1257 (m), 1209 (m), 1166 (m), 1141 (s), 1122 (s), 1083 (m), 1042 (m), 1027 (m), 928 (m), 886 (m), 849 (m), 824 (s), 788 (m), 769 (s), 733 (m), 656 (s), 622 (s), 574 (m). UV/Vis (MeOH): $\lambda_{max}$ (log $\varepsilon$) = 202 (4.23), 229 (4.42), 279 (4.32), 294 (3.97), 306 (3.80). HRESIMS: calculated ([$C_{15}H_{15}NO_2$+$Na^+$]) 264.09950, found 264.09961 (0.42 ppm).

***tert*-Butyl 6-(3-oxobutyl)-1*H*-indole-1-carboxylate (6):** *tert*-Butyl 6-iodo-1*H*-indole-1-carboxylate (**3**, 1.00 g, 2.91 mmol, 1.00 equiv), LiCl (245 mg, 5.82 mmol, 2.00 equiv), NaOAc (120 mg, 1.46 mg, 0.50 equiv) and $H_2O$ (203 µL, 11.29 mmol, 3.88 equiv) were dissolved in DMAC (11 mL) and the mixture was degassed by sonication. But-3-en-2-ol (**5**, 313 µL, 3.49 mmol, 1.20 equiv), $Pd(OAc)_2$ (34 mg, 0.15 mmol, 5 mol %) and $NEt_3$ (130 µL, first portion of 420 µL, 3.02 mmol, 1.04 equiv) were added and the mixture was heated to 75 °C. At this temperature the second portion of $NEt_3$ (170 µL) was added and stirring was continued for 15 min followed by the addition of the third portion of $NEt_3$ (120 µL). After stirring for another 2 h the mixture was cooled to room temperature and stirred overnight. The mixture was filtered through Celite 545. The Celite pad was washed with TBME (150 mL) and the organic phase was washed with $H_2O$ and brine (100 mL each). The combined aqueous phases were extracted further with TBME (50 mL). After drying of the combined organic phases with $MgSO_4$ and filtration, the solvent was evaporated and the crude product was subjected to column chromatography using petroleum ether/EtOAc (10:1 to 5:1) as the eluent. The product was isolated as slightly yellow oil (743 mg, 2.58 mmol, 89%). TLC [petroleum ether/EtOAc (10:1)]: $R_f$ = 0.29. $^1$H NMR (400 MHz, $CDCl_3$): $\delta$ = 8.03 (s, 1H, 7-*H*), 7.52 (d, 1H, $J$ = 3.7 Hz, 2-*H*), 7.45 (d, 1H, $J$ = 8.0 Hz, 4-*H*), 7.06 (dd, 1H, $J$ = 8.1 Hz, $J$ = 1.5 Hz, 5-*H*), 6.51 (dd, 1H, $J$ = 3.7 Hz, $J$ = 0.6 Hz, 3-*H*), 3.02 (t, 2H, $J$ = 7.7 Hz, C(=O)$CH_2$C$H_2$), 2.82 (t, 2H, $J$ = 7.7 Hz, C(=O)C$H_2$), 2.15 (s, 3H, C$H_3$C(=O)), 1.67 (s, 9H, C(C$H_3$)$_3$). $^{13}$C NMR (100 MHz, $CDCl_3$): $\delta$ = 208.0 (1C, C(=O)), 149.8 (1C, NC(=O)), 137.3 (1C, C-6), 135.6 (1C, C-7a), 128.8 (1C, C-3a), 125.6 (1C, C-2), 123.3 (1C, C-5), 120.8 (1C, C-4), 114.7 (1C, C-7), 107.1 (1C, C-3), 83.5 (1C, C($CH_3$)$_3$), 45.8 (1C, C(=O)$CH_2$), 30.3 (1C, C(=O)$CH_2CH_2$), 30.1 (1C, $CH_3$C(=O)), 28.2 (3C, C($CH_3$)$_3$). IR (diamond ATR): $\tilde{\nu}$ = 2979 (w), 2933 (w), 1715 (s), 1616 (w), 1530 (w), 1481 (w), 1439 (m), 1370 (m), 1336 (s), 1253 (s), 1210 (m), 1169 (m), 1145 (s), 1077 (m), 1044 (w), 1022 (m), 930 (w), 885 (w), 852 (w), 812 (m), 767 (m), 724 (m), 646 (w), 590 (w), 571 (w). UV/Vis (MeOH): $\lambda_{max}$ (log $\varepsilon$) = 229 (4.40),

259 (4.07), 283 (3.56), 294 (3.47). HRESIMS: calculated ([$C_{17}H_{21}NO_3+Na^+$]) 310.14136, found 310.14155 (0.61 ppm).

**Di-*tert*-butyl 6,6'-(3-hydroxy-3-methylpent-1-yne-1,5-diyl)bis(1*H*-indole-1-carboxylate) (7):** *tert*-Butyl 6-ethynyl-1*H*-indole-1-carboxylate (**4**, 587 mg, 2.43 mmol, 2.00 equiv) was dissolved in THF (44 mL) and 4-(Boc-indol-6-yl)-butan-2-one (**6**, 350 mg, 1.22 mmol, 1.00 equiv) was dissolved in THF (23 mL). Both solutions were dried overnight with molecular sieves (4 Å). The alkyne solution was cooled to −78 °C and a 2 M solution of iPrMgCl in THF (1.22 mL, 2.43 mmol, 2.00 equiv) was added within 30 min via a syringe pump. After the addition, the mixture was warmed to 0 °C then cooled again to –78 °C followed by the addition of the ketone solution within 1 h via syringe pump and the reaction mixture was warmed to room temperature within 19 h. Monitoring by TLC showed complete consumption of the ketone. The reaction was quenched by addition of saturated $NaHCO_3$ solution (1 mL) and the mixture was filtered through Celite 545. The Celite was washed with TBME (100 mL) and the mixture was concentrated in vacuo to an estimated volume of 10 mL. The residue was diluted with TBME (100 mL). The organic phase was washed with $H_2O$ (3 x 5 0 mL) and brine (50 mL), dried with $MgSO_4$ and filtered. After evaporation of the solvent, the crude product was subjected to column chromatography using petroleum ether/EtOAc (15:1 to 5:1) as the eluent to yield the product as colorless foam/oil (520 mg, 0.98 mmol, 81%). TLC [petroleum ether/EtOAc (10:1)]: $R_f$ = 0.17. Alkynyl indole atoms are marked with '. $^1$H NMR (400 MHz, $CDCl_3$): $\delta$ = 8.31 (s, 1H, 7'-*H*), 8.10 (s, 1H, 7-*H*), 7.62 (d, 1H, *J* = 3.7 Hz, 2'-*H*), 7.52 (d, 1H, *J* = 3.7 Hz, 2-*H*), 7.49 (d, 1H, *J* = 8.1 Hz, 4'-*H*), 7.47 (d, 1H, *J* = 7.9 Hz, 4-*H*), 7.32 (dd, 1H, *J* = 8.1 Hz, *J* = 1.4 Hz, 5'-*H*), 7.15 (dd, 1H, *J* = 8.0 Hz, *J* = 1.5 Hz, 5-*H*), 6.55 (dd, 1H, *J* = 3.7 Hz, *J* = 0.6 Hz, 3'-*H*), 6.52 (dd,1H, *J* = 3.7 Hz, *J* = 0.5 Hz, 3-*H*), 3.12-3.04 (m, 2H, $OCCH_2CH_2$), 2.19-2.12 (m, 2H, $OCCH_2$), 2.11-2.06 (m, 1H, O*H*), 1.67 (s, 9H, $C(CH_3)_3$), 1.66 (s, 3H, $C(OH)CH_3$), 1.65 (s, 9H, $C(CH'_3)_3$). $^{13}$C NMR (100 MHz, $CDCl_3$): $\delta$ = 149.8 (1C, N*C*(=O)), 149.5 (1C, N*C*'(=O)), 138.4 (1C, 6-*C*), 135.7 (2C, *C*-7a), 134.8 (1C, *C*'-7a) 130.5 (1C, *C*'-3a), 128.7 (1C, *C*-3a), 127.0 (1C, *C*'-2), 126.3 (1C, *C*'-5), 125.4 (1C, *C*-2), 123.6 (1C, *C*-5), 120.7 (2C, *C*-4, *C*'-4), 118.8 (1C, *C*'-7), 118.3 (1C, *C*'-6), 114.9 (1C, *C*-7), 107.2 (1C, *C*'-3), 107.1 (1C, *C*-3), 91.6 (1C, C(OH)*C*≡C), 84.9 (1C, C(OH)C≡*C*), 84.0 (1C, $C(CH_3)_3$), 83.5 ($C'(CH_3)_3$), 68.6 (1C, *C*(OH), 46.1 (1C, $C(OH)CH_2$), 31.9 (1C, $C(OH)CH_2CH_2$), 30.2 (1C, $C(OH)CH_3$), 28.2 (3C, $C(C'H_3)_3$), 28.1 (3C, $C(CH_3)_3$). IR (diamond ATR): $\tilde{\nu}$ = 3465 (w), 2978 (w), 2932 (w), 1731 (s), 1615 (w), 1527 (w), 1477 (m), 1437 (m), 1373 (m), 1336 (s), 1305 (m), 1251 (s), 1209 (m), 1147 (s), 1124 (s), 1076 (m), 1041 (m), 1023 (m), 932 (m), 885 (w), 849 (m), 821 (m), 766 (s), 723 (m), 629 (w), 573 (w). UV/Vis (MeOH): $\lambda_{max}$ (log $\varepsilon$) = 229 (4.70), 272 (4.40), 282 (4.43), 294 (4.20), 307 (3.77). HRESIMS: calculated ([$C_{32}H_{36}N_2O_5+Na^+$]) 551.25164, found 551.25181 (0.31 ppm).

***(E/Z)*-Di-*tert*-butyl 6,6'-(3-methyl-1-oxopent-2-ene-1,5-diyl)bis(1*H*-indole-1-carboxylate) (8):** Bisindole **7** (303 mg, 0.57 mmol, 1.00 equiv) was dissolved in dry toluene under argon. $MoO_2(acac)_2$ (1.9 mg, 5.7 µmol, 1 mol %), $AuPPh_3Cl$ (2.8 mg, 5.7 µmol, 1 mol %) and AgOTf (1.5 mg, 5.7 µmol, 1 mol %) were added in that order and the mixture was stirred for 21 h at room temperature. The reaction was quenched with saturated $NaHCO_3$ solution (10 mL), diluted with TBME (60 mL) and washed with brine (60 mL). The organic phase was dried with $MgSO_4$ and filtered. Subjection to column chromatography using petroleum ether/EtOAc (15:1 to 8:1) as the eluent yielded the product **8** (260 mg, 0.49 mmol, 86%) as a mixture of *E*/*Z* isomers as colorless oil, as well as the enyne as a byproduct. Separation of the *E*/*Z* isomers was possible by HPLC. Nevertheless, isomerization was observed. Atoms of the acylated indole are marked with ‘. HRESIMS: calculated ([$C_{32}H_{36}N_2O_5$+$Na^+$]) 551.25164, found 551.25183 (0.35 ppm). *E*-isomer: $^1$H NMR (400 MHz, $CDCl_3$): $\delta$ = 8.73 (s, 1H, 7’-*H*), 8.08 (s, 1H, 7-*H*), 7.75 (d, 1H, *J* = 3.7 Hz, 2’-*H*), 7.69 (dd, 1H, *J* = 8.2 Hz, *J* = 1.5 Hz, 5’-*H*), 7.56-7.53 (m, 1H, 4’-*H*), 7.53-7.51 (m, 1H, 2-*H*), 7.50 (d, 1H, *J* = 8.0 Hz, 4-*H*), 7.12 (dd, 1H, *J* = 8.0 Hz, *J* = 1.5 Hz, 5-*H*), 6.82-6.78 (m, 1H, C(=O)C*H*), 6.60 (dd, 1H, *J* = 3.7 Hz, *J* = 0.4 Hz, 3’-*H*), 6.54 (d, 1H, *J* = 3.7 Hz, 3-*H*), 3.02 (dd, 2H, *J* = 8.7 Hz, *J* = 7.3 Hz, $C(CH_3)CH_2CH_2$), 2.64 (dd, 2H, *J* = 9.4 Hz, *J* = 6.5 Hz, $C(CH_3)CH_2$), 2.27 (d, 3H, *J* = 1.2 Hz, $CH_2CCH_3$), 1.71 (s, 9H, N’C(=O)OC($CH_3$)$_3$), 1.65 (s, 9H, NC(=O)OC($CH_3$)$_3$). $^{13}$C NMR (100 MHz, $CDCl_3$): $\delta$ = 191.9 (1C, *C*(=O)CHC), 157.5 (1C, C(=O)CH*C*), 149.8/149.5 (2C, N’*C*(=O)/N*C*(=O)), 137.6 (1C, *C*-6), 135.7 (1C, *C*-7a), 135.5 (1C, *C*-6’), 134.7 (1C, *C*-7a’), 133.9 (1C, *C*-3a’), 128.9 (1C, *C*-2’), 128.8 (1C, *C*-3a), 125.6 (1C, *C*-4), 123.4 (1C, *C*-5), 122.9 (1C, *C*-5’), 121.8 (1C, C(=O)*C*H), 120.8 (1C, *C*-2), 120.7 (1C, *C*-4’), 115.9 (1C, *C*-7’), 114.9 (1C, *C*-7), 107.2 (1C, *C*-3’), 107.1 (1C, *C*-3), 84.3 (1C, N’C(=O)O*C*), 83.5 (1C, NC(=O)O*C*), 43.9 (1C, $C(CH_3)CH_2$), 34.8 (1C, $C(CH_3)CH_2CH_2$), 28.2 (6C, N’C(=O)OC($CH_3$)$_3$/NC(=O)OC($CH_3$)$_3$), 19.8 (1C, C(=O)CH$CH_3$). IR (diamond ATR): $\tilde{\nu}$ = 2978 (w), 2934 (w), 1730 (s), 1657 (m), 1610 (m), 1524 (w), 1479 (w), 1437 (m), 1370 (m), 1335 (s), 1293 (m), 1252 (s), 1212 (m), 1145 (s), 1121 (s), 1080 (m), 1023 (m), 923 (w), 850 (m), 815 (m), 762 (s), 723 (s), 667 (w), 581 (w), 553 (w). UV/Vis (MeOH): $\lambda_{max}$ (log $\varepsilon$) = 202 (4.33 ), 229 (4.42), 289 (4.02), 294 (4.03). *Z*-isomer: $^1$H NMR (400 MHz, $CDCl_3$): $\delta$ = 8.68 (broad s, 1H, 7‘-*H*), 8.04 (broad s, 1H, 7-*H*), 7.84 (dd, 1H, *J* = 8.2 Hz, *J* = 1.5 Hz, 5‘-*H*), 7.78-7.72 (m, 1H, 2‘-*H*), 7.57 (d, 1H, *J* = 8.3 Hz, 4‘-*H*), 7.46 (d, 1H, *J* = 3.7 Hz, 2-*H*), 7.42 (d, 1H, *J* = 8.0 Hz, 4-*H*), 7.20 (dd, 1H, *J* = 8.0 Hz, *J* = 1.2 Hz, 5-*H*), 6.84 (d, 1H, *J* = 1.0 Hz, C(=O)C*H*), 6.63-6.58 (m, 1H, 3‘-*H*), 6.46 (dd, 1H, *J* = 3.7 Hz, *J* = 0.6 Hz, 3-*H*), 2.99-2.96 (m, 4H, $CH_2CH_2$), 2.02 (d, 3H, *J* = 1.2 Hz, $C(CH_3)CH_2$), 1.71 (s, 9H, N’C(=O)OC($CH_3$)$_3$), 1.67 (s, 9H, NC(=O)OC($CH_3$)$_3$). $^{13}$C NMR (100 MHz, $CDCl_3$): $\delta$ = 191.3 (1C, *C*(=O)CHC), 157.7 (1C, C(=O)CH*C*), 149.8 (1C, N‘CO/NCO), 149.5 (1C, N‘CO/NCO), 138.3 (1C, *C*-6), 135.4 (2C, *C*-6’/*C*-7a), 134.6 (1C, *C*-

7a'), 133.8 (1C, *C*-3a'), 128.8 (1C, *C*-2'), 128.7 (1C, *C*-3a), 125.3 (1C, *C*-2), 123.7 (1C, *C*-5), 122.7 (1C, *C*-5'), 122.4 (1C, C(=O)*C*HC), 120.7 (1C, *C*-4'), 120.6 (1C, *C*-4), 116.0 (1C, *C*-7'), 114.9 (1C, *C*-7), 107.2 (1C, *C*-3'), 107.0 (1C, *C*-3), 84.2 (1C, N'C(=O)O*C*), 83.5 (1C, NC(=O)O*C*), 36.8 (1C, C($CH_3$)*C*$H_2$), 35.1 (1C, C($CH_3$)$CH_2$*C*$H_2$), 28.2 (6C, N'C(=O)OC(*C*$H_3$)$_3$/ NC(=O)OC(*C*$H_3$)$_3$), 25.9 (1C, C(*C*$H_3$)$CH_2$). IR (diamond ATR): $\tilde{\nu}$ = 2975 (w), 2928 (w), 2856 (w), 1730 (s), 1657 (m), 1610 (m), 1524 (w), 1478 (w), 1437 (m), 1386 (m), 1371 (m), 1335 (s), 1293 (m), 1253 (s), 1212 (m), 1145 (s), 1121 (s), 1079 (m), 1022 (m), 924 (w), 849 (m), 814 (m), 763 (s), 722 (m), 647 (w), 580 (w), 552 (w). UV/Vis (MeOH): $\lambda_{max}$ (log $\varepsilon$) = 201 (4.29), 229 (4.39), 294 (4.01).

**2-Iodo-5-methylphenol (10) [S3]:** 2-Amino-5-methylphenol (**9**, 2.00 g, 16.24 mmol, 1.00 equiv) was dissolved in acetone (28 mL) and cooled with an ice/salt mixture. Concentrated HCl (3.4 mL, 40.60 mmol, 2.5 equiv) was added in that way that the temperature did not rise above 0 °C. Then a solution of $NaNO_2$ (1.12 g, 16.24 mmol, 1.00 equiv) dissolved in 3.3 mL $H_2O$ was added within 15 min below 0 °C. The mixture was further stirred for 1 h at this temperature and then cooled to −10 °C. A solution of NaI (4.87 g, 32.48 mmol, 2.00 equiv) in 5.4 mL $H_2O$ was added within 10 min. Afterwards the temperature was elevated to 0 °C and the mixture was warmed to room temperature. After the addition of $H_2O$ (100 mL) the reaction mixture was extracted four times with EtOAc (50 mL each) and the organic phase was washed with $Na_2S_2O_3$ solution (100 mL), $H_2O$ (100 mL) and brine (100 mL). The combined aqueous phases were further extracted with EtOAc (50 mL). The combined organic phases were dried with $MgSO_4$ and filtered. After filtration the solvent was removed in vacuo and the raw material was subjected to column chromatography on silica gel using petroleum ether/acetone (20:1) as the eluent. The product **10** was obtained as red solid (2.74 g, 11.71 mmol, 72%). TLC [petroleum ether/acetone (20:1)]: $R_f$ = 0.15. Mp: 34-36 °C. $^1$H NMR (400 MHz, $CDCl_3$): $\delta$ = 7.50 (d, 1H, *J* = 8.1 Hz, ClC*H*), 6.82 (dd, 1H, *J* = 1.9 Hz, *J* = 0.6 Hz, C(OH)C*H*), 6.53-6.49 (m, 1H, ClCHC*H*), 5.18 (s, 1H, O*H*), 2.28 (s, 3H, C*H*$_3$). $^{13}$C NMR (100 MHz, $CDCl_3$): $\delta$ = 154.6 (1C, *C*(OH)), 140.7 (1C, *C*$CH_3$), 137.7 (1C, Cl*C*H), 123.5 (1C, ClCH*C*H), 115.9 (1C, C(OH)*C*H), 81.6 (1C, *C*I), 21.0 (1C, *C*$H_3$). IR (diamond ATR): $\tilde{\nu}$ = 3474 (s), 3060 (w), 3032 (w), 2949 (w), 2918 (w), 2858 (w), 1572 (m), 1477 (m), 1447 (m), 1405 (m), 1308 (m), 1291 (m), 1247 (m), 1195 (s), 1159 (s), 1123 (m), 1015 (s), 939 (m), 856 (m), 794 (s), 728 (m), 577 (m), 553 (m). UV/Vis (MeOH): $\lambda_{max}$ (log $\varepsilon$) = 206 (4.47), 229 (3.95), 282 (3.47). MS (EI, 70 eV): *m/z* (%) = 234 (100), 205 (1), 165 (2), 127 (18), 117 (1), 107 (41), 89 (2), 77 (45), 63 (6), 51 (13). HREIMS: calculated ([$C_7H_7IO^+$]) 233.95361, found 233.95269 (3.93 ppm).

**2-Iodo-5-methyl-4-nitrophenol (11):** 2-Iodo-5-methylphenol (**10**, 5.00 g, 21.36 mmol, 1.00 equiv) was dissolved in $CH_2Cl_2$ (43 mL). $HNO_3$ (65%, 1.63 mL, 23.50 mmol, 1.10 equiv) was added dropwise while the reaction mixture was cooled with a water bath. After the addition of $HNO_3$ was completed, the mixture was stirred for further 23 h at room temperature. Then $CH_2Cl_2$ (50 mL) was added and the organic phase was washed with $H_2O$ (100 mL), $Na_2S_2O_3$ solution (150 mL) and brine (100 mL). The combined aqueous phases were further extracted with EtOAc (100 mL). The combined organic phases were washed dried with $MgSO_4$ and filtered. After filtration and evaporation of the solvent the crude product was subjected to column chromatography using petroleum ether/EtOAc (5:1) as the eluent. The obtained product had a strong smell and was dissolved in TBME (200 mL). The solution was extracted with 2 N NaOH (100 mL). After neutralization of the aqueous phase with concentrated HCl, the mixture was extracted with TBME (2 x 100 mL). The organic phase was dried with $MgSO_4$ and filtered. Evaporation of the solvent gave the product as yellow solid (2.66 g, 9.53 mmol, 45%). TLC [petroleum ether/acetone (5:1)]: $R_f = 0.28$. Mp: 136-138 °C. $^1$H NMR (400 MHz, $CDCl_3$): $\delta = 8.44$ (s, 1H, ClC*H*), 6.92-6.90 (m, 1H, C(OH)C*H*), 5.87 (s, 1H, O*H*), 2.60-2.58 (m, 3H, C*H*$_3$). $^{13}$C NMR (100 MHz, $CDCl_3$): $\delta = 158.8$ (1C, *C*(OH)), 142.8 (1C, *C*NO$_2$), 137.7 (1C, *C*CH$_3$), 135.6 (1C, Cl*C*H), 117.9 (1C, C(OH)*C*H), 81.3 (1C, *C*I), 21.2 (1C, *C*H$_3$). IR (diamond ATR): $\tilde{\nu}$ = 3288 (m), 3082 (m), 2922 (m), 2853 (m), 1591 (m), 1570 (m), 1499 (s), 1475 (s), 1450 (s), 1395 (w), 1379 (m), 1311 (s), 1258 (s), 1196 (s), 1091 (s), 1030 (m), 983 (m), 897 (s), 851 (s), 754 (m), 732 (m), 704 (w), 670 (s), 579 (m), 549 (m). UV/Vis (MeOH): $\lambda_{max}$ (log $\varepsilon$) = 202 (4.08), 227 (4.01), 248 (3.80), 288 (3.60), 322 (3.58), 390 (3.42). MS (EI, 70 eV): *m/z* (%) = 279 (39), 262 (100), 249 (9), 207 (3), 179 (3), 152 (1), 135 (16), 127 (23), 105 (17), 94 (6), 78 (33), 63 (9), 51 (27). HREIMS: calculated ([$C_7H_6INO_3^+$]) 278.93869, found 278.93890 (0.75 ppm).

**1-(Benzyloxy)-2-iodo-5-methyl-4-nitrobenzene (12):** In a dried Schlenk flask 2-iodo-5-methyl-4-nitrophenol (**11**, 92 mg, 0.33 mmol, 1.00 equiv) was dissolved in acetone (5 mL). Then $K_2CO_3$ (91 mg, 0.66 mmol, 2.00 equiv) and pulverized molecular sieves (4 Å) were added and the mixture was stirred for 10 min. Benzyl bromide (40 µL, 0.33 mmol, 1.00 equiv) was added and the mixture was stirred overnight. Monitoring by TLC showed incomplete consumption of the starting material and further benzyl bromide (40 µL, 0.33 mmol, 1.00 equiv) was added. After stirring for further 5 h the reaction mixture was filtered through a pad of Celite 545 and the solvent was evaporated. The crude product was subjected to column chromatography using petroleum ether/acetone (10:1) as the eluent to yield product **12** as colorless solid (113 mg, 0.31 mmol, 94%). TLC [petroleum ether/acetone (10:1)]: $R_f = 0.37$. Mp: 99-100 °C. $^1$H NMR (400 MHz, $CDCl_3$): $\delta = 8.54$ (s, 1H, ClC*H*), 7.52-7.33 (m, 5H, CH$_2$C$_6$*H*$_5$), 6.71 (s, 1H, C(CH$_3$)C*H*), 5.22 (s, 2H, C*H*$_2$), 2.60 (s, 3H, C*H*$_3$). $^{13}$C NMR

(100 MHz, $CDCl_3$): $\delta$ = 160.7 (1C, *C*O$CH_2$), 142.8 (1C, *C*$NO_2$), 137.0 (1C, *C*$CH_3$), 136.4 (1C, CI*C*H), 135.2 (1C, *C*$CH_2$), 128.8 (2C, *m*-benzyl-*C*), 128.4 (1C, *p*-benzyl-*C*), 127.0 (2C, *o*-benzyl-*C*), 114.7 (1C, C($CH_3$)*C*H), 82.5 (1C, *C*I), 71.4 (1C, *C*$H_2$), 21.7 (1C, *C*$H_3$). IR (diamond ATR): $\tilde{\nu}$ = 3099 (w), 3032 (w), 2911 (w), 2860 (w), 1593 (m), 1561 (m), 1500 (m), 1481 (m), 1444 (m), 1375 (m), 1323 (s), 1262 (s), 1178 (m), 1155 (w), 1091 (m), 1079 (m), 1044 (m), 1018 (m), 970 (w), 901 (m), 848 (m), 826 (w), 756 (w), 729 (s), 681 (m), 613 (w), 598 (w). UV/Vis (MeOH): $\lambda_{max}$ (log $\varepsilon$) = 203 (4.42), 229 (4.16), 246 (4.03), 289 (3.85). MS (EI, 70 eV): *m/z* (%) = 369 (6), 248 (3), 91 (100), 77 (2), 65 (6). HREIMS: calculated ([$C_{14}H_{12}INO_3^+$]) 368.98564, found 368.98612 (1.30 ppm).

**5-(Benzyloxy)-6-iodo-1*H*-indole:** 1-(Benzyloxy)-2-iodo-5-methyl-4-nitrobenzene (**12**, 1.025 g, 2.78 mmol, 1.00 equiv) was dissolved in DMF (60 mL) and DMFDMA (1.2 mL, 8.96 mmol, 3.22 equiv) and pyrrolidine (228 µL, 2.78 mmol, 1.00 equiv) were added in that order. The mixture was heated to 110 °C for 3 h. Monitoring by TLC indicated remaining starting material and additional DMFDMA (186 µL, 1.39 mmol, 0.5 equiv) and pyrrolidine (12 µL, 146 µmol, 0.05 equiv) were added and the mixture was heated to 110 °C for further 60 min after which monitoring by TLC showed complete consumption of the starting material. Then a 4 M solution of $NH_4OAc$ (3.76 g, 48.79 mmol, 17.55 equiv) in $H_2O$ (12 mL) was added and the mixture was cooled to 0 °C. At this temperature, a solution of 20% $TiCl_3$ in 3% HCl (10.7 mL, 16.68 mmol, 6.00 equiv) was added dropwise within 15 min and stirred for another 30 min. The mixture was warmed to room temperature, stirred for 60 min, diluted with TBME (100 mL) and washed with half concentrated brine (400 mL). The aqueous phase was extracted with TBME (100 mL) and the combined organic phases were washed with half concentrated brine (200 mL) and brine (200 mL). The organic phase was dried with $MgSO_4$, filtered and the solvent evaporated. The crude product was subjected to column chromatography using petroleum ether/EtOAc (10:1) as the eluent to yield 5-(benzyloxy)-6-iodo-1*H*-indole (917 mg, 2.63 mmol, 95%) as colorless oil. TLC [petroleum ether/acetone (10:1)]: $R_f$ = 0.22. $^1$H NMR (400 MHz, $CDCl_3$): $\delta$ = 8.03 (s, 1H, N*H*), 7.84 (d, 1H, *J* = 0.8 Hz, 7-*H*), 7.58-7.54 (m, 2H, *o*-benzyl-*H*), 7.42-7.38 (m, 2H, *m*-benzyl-*H*), 7.34-7.30 (m, 1H, *p*-benzyl-*H*), 7.15 (dd, 1H, *J* = 3.1 Hz, *J* = 2.5 Hz, 2-*H*), 7.14 (s, 1H, 4-*H*), 6.45 (ddd, 1H, *J* = 3.1 Hz, *J* = 2.0 Hz, *J* = 0.9 Hz, 3-*H*), 5.16 (s, 2H, C*$H_2$*). $^{13}$C NMR (100 MHz, $CDCl_3$): $\delta$ = 151.1 (1C, *C*-5), 137.2 (1C, *C*$CH_2$), 132.5 (1C, *C*-7a), 128.6 (1C, *C*-3a), 128.5 (2C, *m*-benzyl-*C*), 127.7 (1C, *p*-benzyl-*C*), 127.1 (2C, *o*-benzyl-*C*), 125.3 (1C, *C*-2), 121.4 (1C, *C*-7), 103.8 (1C, *C*-4), 102.6 (1C, *C*-3), 81.3 (1C, *C*I), 71.7 (1C, *C*$H_2$). IR (diamond ATR): $\tilde{\nu}$ = 3422 (m), 3062 (w), 3030 (w), 2867 (w), 1566 (w), 1496 (w), 1451 (s), 1407 (w), 1379 (w), 1302 (s), 1240 (w), 1208 (m), 1157 (s), 1096 (w), 1024 (m), 900 (w), 838 (m), 815 (w), 738 (s), 720 (s),

696 (s), 664 (w), 605 (w), 550 (w). UV/Vis (MeOH): $\lambda_{max}$ (log $\varepsilon$) = 206 (4.56), 281 (3.93). MS (EI, 70 eV): *m/z* (%) = 349 (51), 288 (17), 258 (61), 248 (13), 230 (27), 222 (69), 204 (3), 194 (7), 177 (1), 165 (5), 146 (2), 131 (10), 115 (5), 103 (21), 91 (100), 76 (14), 65 (14), 51 (7), 44 (43). HREIMS: calculated ([$C_{14}H_{12}INO_3^+$]) 348.99581, found 348.99584 (0.09 ppm).

***tert*-Butyl 5-(benzyloxy)-6-iodo-1*H*-indole-1-carboxylate (15):** 5-(Benzyloxy)-6-iodo-1*H*-indole (313 mg, 0.90 mmol, 1.00 equiv) was dissolved in DCM (50 mL) and di-*tert*-butyl dicarbonate (295 mg, 1.35 mmol, 1.50 equiv) and DMAP (22 mg, 0.18 mmol, 20 mol %) were added at room temperature. After stirring for 3 h the solvent was evaporated. Purification by column chromatography using petroleum ether/acetone (30:1) as the eluent yielded the product (386 mg, 0.86 mmol, 96%) as colorless oil. TLC [petroleum ether/acetone (30:1)]: $R_f$ = 0.39. Mp: 114-115 °C. $^1$H NMR (400 MHz, $CDCl_3$): $\delta$ = 8.63 (s, 1H, 7-*H*), 7.56-7.49 (m, 3H, *o*-benzyl-*H*, 2-*H*), 7.43-7.37 (m, 2H, *m*-benzyl-*H*), 7.35-7.29 (m, 1H, *p*-benzyl-*H*), 7.02 (s, 1H, 4-*H*), 6.45 (dd, 1H, *J* = 3.7 Hz, *J* = 0.5 Hz, 3-*H*), 5.17 (s, 2H, C*H*$_2$), 1.66 (s, 9H, C(C*H*$_3$)$_3$). $^{13}$C NMR (100 MHz, $CDCl_3$): $\delta$ = 153.0 (1C, *C*-5), 148.0 (1C, N*C*O), 136.8 (1C, CH$_2$*C*), 131.4 (1C, *C*-7a), 131.1 (1C, *C*-3a), 128.5 (2C, *m*-benzyl-*C*), 127.8 (1C, *p*-benzyl-*C*), 127.1 (2C, *o*-benzyl-*C*), 126.7 (1C, *C*-2), 125.8 (1C, *C*-7), 106.9 (1C, *C*-3), 103.8 (1C, *C*-4), 84.0 (1C, *C*(CH$_3$)$_3$), 83.2 (1C, *C*-6), 71.4 (1C, *C*H$_2$), 28.1 (3C, *C*(CH$_3$)$_3$). IR (diamond ATR): $\tilde{\nu}$ = 3167 (w), 3107 (w), 2974 (w), 2931 (w), 2895 (w), 2863 (w), 1724 (s), 1606 (w), 1574 (w), 1449 (s), 1426 (m), 1367 (s), 1282 (s), 1193 (m), 1166 (m), 1137 (s), 1088 (s), 1027 (s), 969 (w), 908 (w), 875 (w), 820 (m), 765 (m), 735 (s), 718 (m), 694 (m), 676 (m), 608 (w), 568 (m), 541 (w). UV/Vis (MeOH): $\lambda_{max}$ (log $\varepsilon$) = 203 (4.35), 241 (4.27), 273 (4.12), 302 (3.57), 312 (3.57). MS (EI, 70 eV): *m/z* (%) = 449 (7), 393 (11), 349 (40), 266 (9), 258 (61), 230 (23), 222 (68), 204 (3), 194 (7), 165 (4), 131 (10), 115 (4), 103 (15), 91 (100), 76 (10), 57 (14). HREIMS: calculated ([$C_{20}H_{20}INO_3^+$]) 449.04824, found 449.04912 (1.96 ppm).

***tert*-Butyl 5-(benzyloxy)-6-((trimethylsilyl)ethynyl)-1*H*-indole-1-carboxylate:** *tert*-Butyl 5-(benzyloxy)-6-iodo-1*H*-indole-1-carboxylate (**15**, 375 mg, 0.84 mmol, 1.00 equiv) and $Pd(PPh_3)_2Cl_2$ (18 mg, 25.2 µmol, 3 mol %) were placed in a Schlenk flask and oxygen was removed. Then $NEt_3$ (15 mL) and ethynyltrimethylsilane (242 µL, 1.68 mmol, 2.00 equiv) were added and the solution was degassed by sonication and simultaneous washing of the solution with argon for 15 min. Separately, CuI (5 mg, 25.2 µmol, 3 mol %) was placed in a Schlenk flask and oxygen was removed. After the addition of $NEt_3$ (3 mL) the suspension was degassed as described above for 5 min (CuI will be dissolved afterwards). The CuI solution was added to the reaction mixture and it was heated to 40 °C for 2 h. The reaction mixture was filtered through Celite 545, the solvent was evaporated and the crude product

was purified by column chromatography using petroleum ether/acetone (30:1) as the eluent to yield the product (344 mg, 0.82 mmol, 98%) as colorless solid. TLC [petroleum ether/acetone (30:1)]: $R_f$ = 0.35. Mp: 103-105 °C. $^1$H NMR (400 MHz, $CDCl_3$): $\delta$ = 8.29 (broad s, 1H, 7-*H*), 7.61-7.52 (m, 3H, 2-*H*, *o*-benzyl-*H*), 7.41-7.35 (m, 2H, *m*-benzyl-*H*), 7.33-7.28 (m, 1H, *p*-benzyl-*H*), 7.03 (s, 1H, 4-*H*), 6.46 (dd, 1H, *J* = 3.6 Hz, *J* = 0.6 Hz, 3-*H*), 5.17 (s, 2H, C*H*$_2$), 1.66 (s, 9H, C(C*H*$_3$)$_3$), 0.27 (s, 9H, Si(C*H*$_3$)$_3$). $^{13}$C NMR (100 MHz, $CDCl_3$): $\delta$ = 155.8 (1C, *C*-6), 149.4 (1C, N*C*O), 137.3 (1C, CH$_2$*C*), 131.6 (1C, *C*-3a), 129.6 (1C, *C*-7a), 128.3 (2C, *m*-benzyl-*C*), 127.62 (1C, *C*-2), 127.57 (1C, *p*-benzyl-*C*), 126.8 (2C, *o*-benzyl-*C*), 120.4 (1C, *C*-7), 110.5 (1C, *C*-6), 107.1 (1C, *C*-3), 104.2 (1C, *C*-4), 102.4 (1C, *C*≡CSi), 97.8 (1C, C≡*C*Si), 83.9 (1C, *C*(CH$_3$)$_3$), 70.8 (1C, *C*H$_2$), 28.2 (3C, C(*C*H$_3$)$_3$), 0.1 (3C, Si(*C*H$_3$)$_3$). IR (diamond ATR): $\tilde{\nu}$ = 3063 (w), 3034 (w), 2963 (w), 2928 (w), 2141 (m), 1729 (s), 1620 (w), 1572 (w), 1469 (m), 1444 (s), 1388 (s), 1369 (s), 1289 (s), 1247 (m), 1202 (m), 1178 (m), 1124 (s), 1043 (w), 1029 (w), 1005 (m), 880 (s), 839 (s), 750 (s), 718 (s), 685 (s), 634 (m), 571 (w), 546 (w). UV/Vis (MeOH): $\lambda_{max}$ (log $\varepsilon$) = 209 (4.46), 224 (4.35), 236 (4.39), 291 (4.44), 330 (3.81). HRESIMS: calculated ([$C_{25}H_{29}NO_3Si+Na^+$]) 442.18089, found 442.18132 (0.97 ppm).

***tert*-Butyl 5-(benzyloxy)-6-ethynyl-1*H*-indole-1-carboxylate (18):** *tert*-Butyl 5-(benzyloxy)-6-((trimethylsilyl)ethynyl)-1*H*-indole-1-carboxylate (333 mg, 0.79 mmol, 1.00 equiv) was dissolved in THF (20 mL) and cooled to 0 °C. Then a 1 M solution of TBAF in THF containing 5% $H_2O$ (790 µL, 0.79 mmol, 1.00 equiv) was added and stirring continued for 10 min at this temperature. The mixture was warmed to room temperature and stirred for another 10 min. The solution was diluted with TBME (100 mL) and washed with $H_2O$ (200 mL). After extraction of the aqueous phase with TBME (50 mL) the organic phases were combined and washed with brine (50 mL). After drying with $MgSO_4$, filtration and evaporation of the solvent, the crude product was subjected to column chromatography using petroleum ether/acetone (15:1) as the eluent. The product was obtained as colorless oil (270 mg, 0.78 mmol, 99%). TLC [petroleum ether/acetone (15:1)]: $R_f$ = 0.31. $^1$H NMR (400 MHz, $CDCl_3$): $\delta$ = 8.28 (broad s, 1H, 7-*H*), 7.59 (d, 1H, *J* = 3.6 Hz, 2-*H*), 7.53-7.48 (m, 2H, *o*-benzyl-*H*), 7.40-7.35 (m, 2H, *m*-benzyl-*H*), 7.33 (m, 1H, *p*-benzyl-*H*), 7.02 (s, 1H, 4-*H*), 6.45 (d, 1H, *J* = 3.7 Hz, 3-*H*), 5.22 (s, 2H, C*H*$_2$), 3.30 (s, 1H, C≡C*H*), 1.67 (s, 9H, C(C*H*$_3$)$_3$). $^{13}$C NMR (100 MHz, $CDCl_3$): $\delta$ = 155.8 (1C, *C*-5), 149.4 (1C, N*C*O), 137.1 (1C, CH$_2$*C*), 131.9 (1C, *C*-3a), 129.4 (1C, *C*-7a), 128.5 (2C, *m*-benzyl-*C*), 127.8 (1C, *C*-2), 127.7 (1C, *p*-benzyl-*C*), 126.9 (2C, *o*-benzyl-*C*), 120.8 (1C, *C*-7), 109.3 (1C, *C*-6), 107.1 (1C, *C*-3), 104.3 (1C, *C*-4), 84.0 (1C, *C*(CH$_3$)$_3$), 81.1 (1C, *C*≡CH), 80.5 (1C, C≡*C*H), 71.0 (1C, C*H*$_2$), 28.2 (3C, C(*C*H$_3$)$_3$). IR (diamond ATR): $\tilde{\nu}$ = 3286 (w), 2978 (w), 2931 (w), 2104 (w), 1729 (s), 1617 (w), 1572 (w), 1459 (m), 1443 (m),

1370 (s), 1286 (s), 1197 (m), 1158 (m), 1113 (s), 1027 (w), 991 (m), 889 (w), 865 (m), 842 (m), 824 (m), 761 (m), 729 (m), 694 (m), 646 (m), 597 (m), 563 (w). UV/Vis (MeOH): $\lambda_{max}$ (log $\varepsilon$) = 208 (4.46), 237 (4.36), 284 (4.35), 313 (3.73), 326 (3.72). HRESIMS: calculated ([$C_{22}H_{21}NO_3$+$Na^+$]) 370.14136 , found 370.14151 (0.41 ppm).

***tert*-Butyl 5-(benzyloxy)-6-(5-(1-(*tert*-butoxycarbonyl)-1*H*-indol-6-yl)-3-hydroxy-3-methylpent-1-yn-1-yl)-1*H*-indole-1-carboxylate (21):** Alkyne **18** (260 mg, 0.75 mmol, 2.00 equiv) and *tert*-butyl 6-(3-oxobutyl)-1*H*-indole-1-carboxylate (**6**, 106 mg, 0.37 mmol, 1.00 equiv) were dried in separate Schlenk flasks. Both compounds were dissolved in THF (13 mL and 6 mL, respectively) and stirred with molecular sieves (4 Å) for 1 h at room temperature. The alkyne solution was cooled to −78 °C and a 2 M solution of iPrMgCl (375 µL, 0.75 mmol, 2.00 equiv) was added dropwise within 20 min with a syringe pump. The solution was warmed to 0 °C and then cooled again to −78 °C. At this temperature, the ketone solution was added dropwise with a syringe pump within 30 min (flask washed with 2 mL THF). The reaction mixture was warmed to 12 °C and TLC analysis showed complete consumption of the ketone. The reaction was quenched with saturated $NaHCO_3$ solution (1 mL) and filtered through a pad of Celite 545. The Celite pad was washed with TBME (100 mL). The organic phase was washed with $H_2O$ (150 mL) and the aqueous phase was further extracted with TBME (50 mL). The combined organic phases were washed with brine (120 mL), dried with $MgSO_4$ and filtered. After evaporation of the solvent the crude product was subjected to column chromatography on silica gel using petroleum ether/EtOAc (15:1 to 5:1) as the eluent to yield the product as colorless oil (157 mg, 0.25 mmol, 67%). Excess alkyne **18** was recovered (139 mg, 0.40 mmol). TLC [petroleum ether/acetone (5:1)]: $R_f$ = 0.31. Alkynyl indole atoms are marked with ‘. $^1$H NMR (400 MHz, $CDCl_3$): $\delta$ = 8.26 (broad s, 1H, 7’-*H*), 8.06 (broad s, 1H, 7-*H*), 7.58 (broad s, 1H, 2’-*H*), 7.53-7.49 (m, 3H, *o*-benzyl-*H*, 2-*H*), 7.43 (d, 1H, *J* = 8.0 Hz, 4-*H*), 7.31-7.27 (m, 2H, *m*-benzyl-*H*), 7.24-7.20 (m, 1H, *p*-benzyl-*H*), 7.08 (dd, 1H, *J* = 8.0 Hz, *J* = 1.3 Hz, 5-*H*), 7.05 (s, 1H, 4’-*H*), 6.51 (d, 1H, *J* = 3.7 Hz, 3-*H*), 6.47 (d, 1H, *J* = 3.6 Hz, 3’-*H*), 5.18 (s, 2H, OC*H*$_2$), 3.10-3.01 (m, 2H, C(OH)CH$_2$C*H*$_2$), 2.18-2.09 (m, 2H, C(OH)C*H*$_2$), 1.66 (s, 9H, N’CO$_2$C(C*H*$_3$)$_3$), 1.65 (s, 3H, C(OH)C*H*$_3$), 1.64 (s, 9H, NCO$_2$C(C*H*$_3$)$_3$). $^{13}$C NMR (100 MHz, $CDCl_3$): $\delta$ = 155.5 (1C, *C*-5’), 149.8 (N*C*O), 149.4 (N’*C*O), 138.6 (1C, *C*-6), 137.1 (1C, OCH$_2$*C*), 135.6 (1C, *C*-7a), 131.5 (1C, *C*-3a’), 129.5 (1C, *C*-7a’), 128.6 (1C, *C*-3a), 128.4 (2C, *m*-benzyl-*C*), 127.7 (1C, *p*-benzyl-*C*), 127.5 (1C, *C*-2’), 127.1 (2C, *o*-benzyl-*C*), 125.4 (1C, *C*-2), 123.7 (1C, *C*-5), 120.6 (1C, *C*-4), 120.1 (1C, *C*-7’), 114.9 (1C, *C*-7), 109.9 (1C, *C*-6’), 107.14 (1C, *C*-3’), 107.11 (1C, *C*-3), 104.0 (1C, *C*-4’), 96.1 (1C, C(OH)*C*≡C), 83.9 (1C, N’CO$_2$*C*(CH$_3$)$_3$), 83.4 (1C, NCO$_2$*C*(CH$_3$)$_3$), 81.3 (1C, C(OH)C≡*C*), 70.9 (1C, O*C*H$_2$), 68.8 (1C, *C*(OH)), 46.2 (1C, C(OH)*C*H$_2$), 31.8 (1C, C(OH)CH$_2$*C*H$_2$), 30.2 (1C, C(OH)*C*H$_3$), 28.2 (6C, C(*C*H$_3$)$_3$). IR (diamond ATR): $\tilde{\nu}$ = 3507 (w), 2978 (w), 2930 (w), 2865 (w), 1730 (s),

1618 (w), 1530 (w), 1442 (s), 1371 (s), 1339 (s), 1292 (s), 1253 (m), 1211 (w), 1125 (s), 1077 (m), 1024 (m), 999 (m), 930 (w), 885 (w), 846 (m), 822 (m), 764 (m), 728 (m), 695 (m), 653 (w), 563 (w). UV/Vis (MeOH): $\lambda_{max}$ (log $\varepsilon$) = 206 (4.61), 232 (4.66), 265 (4.35), 287 (4.44), 313 (3.77), 326 (3.76). HRESIMS: calculated ([$C_{39}H_{42}N_2O_6$+$Na^+$]) 657.29351, found 657.29396 (0.68 ppm).

**(*E*/*Z*)-*tert*-Butyl 5-(benzyloxy)-6-(5-(1-(*tert*-butoxycarbonyl)-1*H*-indol-6-yl)-3-methylpent-2-enoyl)-1*H*-indole-1-carboxylate (24):** Bisindole **21** (41 mg, 64.59 μmol, 1.00 equiv) was dried in high vacuum along with $MoO_2(acac)_2$ (2.1 mg, 6.46 μmol, 10 mol %) and $AuPPh_3Cl$ (3.2 mg, 6.46 μmol, 10 mol %). Dry toluene (2 mL) and AgOTf (1.7 mg, 6.46 μmol, 10 mol %) were added to the solution at room temperature. After stirring for 1 h at room temperature reaction monitoring by TLC revealed complete consumption of the starting material. The reaction was quenched with saturated $NaHCO_3$ solution (1 mL), diluted with TBME (20 mL) and washed with brine (40 mL). The organic phase was dried with $MgSO_4$, filtered and the solvent was evaporated. The crude product was subjected to column chromatography on silica gel using petroleum ether/EtOAc (15:1 to 10:1) as the eluent to yield *E*-**24** (14 mg, 22.06 μmol, 34%) and *Z*-**24** (9 mg, 14.18 μmol, 22%) as colorless oils. Acyl indole atoms are marked with ‘. *E*-**24**: TLC [petroleum ether/EtOAc (10:1)]: $R_f$ = 0.26. $^1$H NMR (400 MHz, $CDCl_3$): $\delta$ = 8.38 (broad s, 1H, 7’-*H*), 8.00 (broad s, 1H, 7-*H*), 7.68 (broad s, 1H, 2’-*H*), 7.54-7.47 (m, 1H, 2-*H*), 7.46-7.42 (m, 3H, *o*-benzyl-*H*, 4-*H*), 7.38-7.33 (m, 2H, *m*-benzyl-*H*), 7.32-7.26 (m, 1H, *p*-benzyl-*H*), 7.106 (s, 1H, 4’-*H*), 6.98 (dd, 1H, *J* = 8.0 Hz, *J* = 1.4 Hz, 5-*H*), 6.78 (d, 1H, *J* = 1.0 Hz, C(=O)C*H*), 6.52-6.49 (m, 2H, 3’-*H*, 3-*H*), 5.13 (s, 2H, OC$H_2$), 2.81-2.76 (m, 2H, $CH_3CCH_2CH_2$), 2.47-2.42 (m, 2H, $CH_3CCH_2$), 2.25 (d, 3H, *J* = 1.0 Hz, $CH_3CCH_2$), 1.683 (s, 9H, N’$CO_2C(CH_3)_3$), 1.66 (s, 9H, $NCO_2C(CH_3)_3$). $^{13}$C NMR (100 MHz, $CDCl_3$): $\delta$ = 193.3 (1C, *C*(=O)CH), 156.1 (1C, C(=O)CH*C*), 153.4 (1C, *C*-5’), 149.8/149.5 (2C, $CO_2C(CH_3)_3$), 137.9 (1C, *C*-6), 136.8 (1C, $OCH_2C$), 135.6 (1C, *C*-7a), 133.8 (1C, *C*-3a’), 129.6 (1C, *C*-7a’), 128.9 (1C, *C*-6’), 128.72 (1C, *C*-2’), 128.65 (1C, *C*-3a), 128.5 (2C, *m*-benzyl-*C*), 128.0 (1C, *p*-benzyl-*C*), 127.3 (2C, *o*-benzyl-*C*), 125.8 (1C, C(=O)*C*H), 125.5 (1C, *C*-2), 123.4 (1C, *C*-5), 120.6 (1C, *C*-4), 117.4 (1C, *C*-7’), 114.7 (1C, *C*-7), 107.1 (1C, *C*-3), 106.8 (1C, *C*-3’), 104.2 (1C, *C*-4’), 84.1 (1C, N’$CO_2C$), 83.5 (1C, $NCO_2C$), 71.2 (1C, $OCH_2$), 43.8 (1C, $CH_3CCH_2$), 34.5 (1C, $CH_3CCH_2CH_2$), 28.2 (6C, $C(CH_3)_3$), 20.0 (1C, $CH_3CCH_2$). IR (diamond ATR): $\tilde{\nu}$ = 2978 (w), 2932 (w), 2871 (w), 1729 (s), 1655 (w), 1618 (m), 1529 (w), 1441 (s), 1370 (s), 1340 (s), 1282 (s), 1251 (m), 1212 (m), 1126 (s), 1088 (m), 1022 (m), 986 (w), 931 (w), 847 (m), 812 (m), 764 (m), 721 (m), 697 (m), 678 (w), 644 (w), 579 (w), 545 (w). UV/Vis (MeOH): $\lambda_{max}$ (log $\varepsilon$) = 241 (4.55), 296 (4.21). HRESIMS: calculated ([$C_{39}H_{42}N_2O_6$+$Na^+$]) 657.29351, found 657.29393 (0.64 ppm). *Z*-**27**: TLC [petroleum ether/EtOAc (10:1)]: $R_f$ = 0.32. $^1$H NMR

(400 MHz, $CDCl_3$): $\delta$ = 8.41 (broad s, 1H, 7'-*H*), 8.08 (broad s, 1H, 7-*H*), 7.68 (broad s, 1H, 2'-*H*), 7.54-7.47 (m, 1H, 2-*H*), 7.46-7.42 (m, 3H, *o*-benzyl-*H*, 4-*H*), 7.38-7.33 (m, 2H, *m*-benzyl-*H*), 7.32-7.26 (m, 1H, *p*-benzyl-*H*), 7.23 (d, 1H, *J* = 8.0 Hz, 5-*H*), 7.112 (s, 1H, 4'-*H*), 6.74 (d, 1H, *J* = 1.2 Hz, C(=O)C*H*), 6.52-6.49 (m, 2H, 3'-*H*, 3-*H*), 5.14 (s, 2H, $OCH_2$), 2.99-2.92 (m, 4H, $CH_2CH_2$), 1.85 (d, 3H, *J* = 1.2 Hz, $CH_3CCH_2$), 1.678 (s, 9H, $N'CO_2C(CH_3)_3$), 1.67 (s, 9H, $NCO_2C(CH_3)_3$). $^{13}C$ NMR (100 MHz, $CDCl_3$): $\delta$ = 192.4 (1C, *C*(=O)CH), 156.6 (1C, C(=O)CH*C*), 153.4 (1C, *C*-5'), 149.8/149.5 (2C, $CO_2(CH_3)_3$), 138.7 (1C, *C*-6), 136.9 (1C, $OCH_2C$), 135.6 (1C, *C*-7a), 133.8 (1C, *C*-3a'), 129.6 (1C, *C*-7a'), 128.9 (1C, *C*-6'), 128.65 (1C, *C*-2'), 128.5 (1C, *C*-3a), 128.4 (2C, *m*-benzyl-*C*), 127.8 (1C, *p*-benzyl-*C*), 127.2 (2C, *o*-benzyl-*C*), 126.6 (1C, C(=O)*C*H), 125.3 (1C, *C*-2), 123.7 (1C, *C*-5), 120.6 (1C, *C*-4), 117.4 (1C, *C*-7'), 114.8 (1C, *C*-7), 107.2 (1C, *C*-3), 106.8 (1C, *C*-3'), 104.3 (1C, C-4'), 84.1 (1C, $N'CO_2C$), 83.5 (1C, $NCO_2C$), 71.1 (1C, $OCH_2$), 37.0 (1C, $CH_3CCH_2$), 35.2 (1C, $CH_3CCH_2CH_2$), 28.2 (6C, $C(CH_3)_3$), 25.8 (1C, $CH_3CCH_2$). IR (diamond ATR): $\tilde{\nu}$ = 2977 (w), 2931 (w), 2868 (w), 1729 (s), 1654 (w), 1618 (m), 1530 (s), 1441 (s), 1371 (s), 1338 (s), 1282 (s), 1252 (m), 1210 (m), 1167 (m), 1127 (s), 1089 (m), 1020 (m), 986 (w), 930 (w), 847 (m), 809 (m), 764 (m), 722 (m), 697 (m), 672 (w), 578 (w), 547(w). UV/Vis (MeOH): $\lambda_{max}$ (log $\varepsilon$) = 240 (4.60), 296 (4.29). HRESIMS: calculated ($[C_{39}H_{42}N_2O_6+Na^+]$) 657.29351, found 657.29398 (0.72 ppm).

**1-((3,4-Dimethoxybenzyl)oxy)-2-iodo-5-methyl-4-nitrobenzene (13):** 2-Iodo-5-methyl-4-nitrophenol (**11**, 500 mg, 1.79 mmol, 1.00 equiv) was placed in a Schlenk flask with potassium carbonate (495 mg, 3.58 mmol, 2.00 equiv) and acetone (18 mL) under argon. Dimethoxybenzylbromide (827 mg, 3.58 mmol, 2.00 equiv) was added at room temperature and the reaction mixture was stirred for 15 h before filtration through a pad of Celite 545 which was washed subsequently with acetone (30 mL). The crude product was absorbed on silica gel (60–200 µm) and then subjected to column chromatography using petroleum ether/acetone (10:1 to 5:1) as the eluent to yield the product as pale yellow solid (735 mg, 1.71 mmol, 96%). TLC [petroleum ether/acetone (5:1)]: $R_f$ = 0.33. Mp: 126-129 °C. $^1H$ NMR (400 MHz, $CDCl_3$): $\delta$ = 8.53 (s, 1H, C*H*CN), 7.06 (d, 1H, *J* = 2.0 Hz, $CH_2CCHCO$), 6.99 (dd, 1H, *J* = 8.1 Hz, *J* = 2.0 Hz, $CH_2CCHCH$), 6.89 (d, 1H, *J* = 8.2 Hz, $CH_2CCHCH$), 6.72 (s, 1H, ClCC*H*), 5.16 (s, 2H, $CH_2$), 3.92 (s, 3H, $CH_3OCCHCCH_2$), 3.90 (s, 3H, $CH_3OCCHCH$), 2.61 (s, 3H, $CH_3CCN$). $^{13}C$ NMR (100 MHz, $CDCl_3$): $\delta$ = 160.7 (1C, ClCO), 149.3 (1C, $CH_3OCCHCCH_2$), 149.1 (1C, $CH_3OCCHCH$), 142.8 (1C, *C*N), 137.0 (1C, $CH_3C$), 136.4 (1C, *C*HCN), 127.6 (1C, $CH_2C$), 119.6 (1C, $CH_3OCCHCH$), 114.8 (1C, $CH_3CCH$), 111.1 (1C, $CH_3OCCHCH$), 110.4 (1C, $CH_3OCCHCCH_2$), 82.7 (1C, *C*I), 71.4 (1C, $CH_2$), 55.97 (1C, $CH_3OCCHCCH_2$), 55.94 (1C, $CH_3OCCHCH$), 21.8 (1C, $CH_3C$). IR (diamond ATR): $\tilde{\nu}$ = 2949

(w), 2843 (w), 1591 (m), 1560 (m), 1521 (m), 1499 (s), 1466 (m), 1451 (m), 1422 (m), 1382 (w), 1365 (w), 1326 (s), 1249 (s), 1163 (s), 1139 (s), 1091 (m), 1020 (s), 992 (s), 951 (m), 895 (m), 865 (m), 847 (m), 815 (s), 778 (w), 757 (s), 721 (w), 677 (m), 609 (w), 562 (m). UV/Vis (MeOH): $\lambda_{max}$ (log $\varepsilon$) = 204 (4.56), 230 (4.22), 283 (3.85). HRESIMS: calculated ([$C_{16}H_{16}INO_5$+$Na^+$]) 451.99654, found 451.99635 (0.42 ppm).

**5-((3,4-Dimethoxybenzyl)oxy)-6-iodo-1*H*-indole:** 1-((3,4-Dimethoxybenzyl)oxy)-2-iodo-5-methyl-4-nitrobenzene (**13**, 716 mg, 1.67 mmol, 1.00 equiv) was dissolved in DMF (36 mL). DMFDMA (671 µL, 5.01 mmol, 3.00 equiv) and pyrrolidine (137 µL, 1.67 mmol, 1.00 equiv) were added and the solution was heated to 110 °C for 5 h until TLC showed complete consumption of the starting material. The reaction mixture was cooled to 0 °C and a solution of $NH_4OAc$ (2.26 g, 29.31 mmol, 17.55 equiv) in $H_2O$ (7.3 mL) was added. Then 20% $TiCl_3$ in 3% HCl (6.44 mL, 10.02 mmol, 6.00 equiv) was added dropwise at this temperature followed by stirring for 4 h at room temperature. Afterwards the reaction mixture was diluted with EtOAc (100 mL) and washed with a 1:1 mixture of brine/$H_2O$ (150 mL). The aqueous phase was further extracted with EtOAc (3 x 50 mL). The combined organic phases were washed with a 1:1 mixture of brine/$H_2O$ (250 mL) and brine (150 mL) before drying with $MgSO_4$. After filtration the crude product was subjected to column chromatography using petroleum ether/acetone (3:1) as the eluent to yield 5-((3,4-dimethoxybenzyl)oxy)-6-iodo-1*H*-indole as yellowish solid (454 mg, 1.11 mmol, 67%). TLC [petroleum ether/acetone (5:1)]: $R_f$ = 0.20. Mp: 150-152 °C. $^1$H NMR (400 MHz, $CDCl_3$): $\delta$ = 8.08 (broad s, 1H, N*H*), 7.83 (d, 1H, *J* = 0.8 Hz, 7-*H*), 7.20 (d, 1H, *J* = 1.9 Hz, $CH_3OCC$*H*$CCH_2$), 7.16-7.14 (m, 2H, 2-*H*, 4-*H*), 7.04 (dd, 1H, *J* = 8.1 Hz, *J* = 2.0 Hz, $CH_3OCCHC$*H*), 6.88 (d, 1H, *J* = 8.2 Hz, $CH_3OCC$*H*CH), 6.45 (ddd, 1H, *J* = 3.0 Hz, *J* = 2.0 Hz, *J* = 0.9 Hz, 3-*H*), 5.09 (s, 2H, C*H*$_2$), 3.93 (s, 3H, C*H*$_3OCCHCCH_2$), 3.89 (s, 3H, C*H*$_3$OCCHCH). $^{13}$C NMR (100 MHz, $CDCl_3$): $\delta$ = 151.0 (1C, *C*$OCH_2$), 149.1 (1C, $CH_3O$*C*$CHCCH_2$), 148.6 (1C, $CH_3O$*C*CHCH), 132.6 (1C, *C*-7a), 129.8 (1C, $OCH_2$*C*), 128.7 (1C, *C*-3a), 125.4 (1C, *C*-2), 121.4 (1C, *C*-7), 119.5 (1C, $CH_3OCCH$*C*H), 111.0 (1C, $CH_3OC$*C*HCH), 110.8 (1C, $CH_3OC$*C*$HCCH_2$), 104.1 (1C, *C*-4), 102.5 (1C, *C*-3), 81.5 (1C, *C*I), 71.8 (1C, C*H*$_2$), 55.95 (1C, *C*$H_3OCCHCCH_2$), 55.93 (1C, *C*$H_3$OCCHCH). IR (diamond ATR): $\tilde{\nu}$ = 3377 (m), 3080 (w), 3013 (w), 2935 (w), 2876 (w), 2837 (w), 1709 (w), 1593 (w), 1566 (w), 1515 (s), 1452 (s), 1418 (m), 1375 (m), 1325 (m), 1298 (m), 1266 (s), 1231 (s), 1205 (m), 1154 (s), 1136 (s), 1101 (m), 1030 (m), 1007 (s), 899 (w), 863 (m), 833 (s), 808 (s), 778 (w), 751 (s), 717 (m), 664 (m), 628 (m), 572 (m). UV/Vis (MeOH): $\lambda_{max}$ (log $\varepsilon$) = 204 (4.61), 226 (4.42), 280 (3.91). MS (EI, 70 eV): *m/z* (%) = 409 (6), 283 (2), 271 (4), 256 (12), 258 (1), 230 (2), 207 (1), 151 (100), 138 (4), 107 (5), 91 (2), 77 (3), 51 (2). HRESIMS: calculated ([$C_{17}H_{16}INO_3$+$Na^+$]) 432.00671, found 432.00702 (0.72 ppm).

***tert*-Butyl 5-((3,4-dimethoxybenzyl)oxy)-6-iodo-1*H*-indole-1-carboxylate (16):** 5-((3,4-Dimethoxybenzyl)oxy)-6-iodo-1*H*-indole (442 mg, 1.08 mmol, 1.00 equiv) was dissolved in $CH_2Cl_2$ at room temperature. $Boc_2O$ (354 mg, 1.62 mmol, 1.50 equiv) and DMAP (27 mg, 0.22 mmol, 20 mol %) were added and the mixture was stirred for 19 h. The solvent was evaporated and the crude product was purified by column chromatography using petroleum ether/acetone (5:1) as the eluent to yield the product as colorless solid (480 mg, 0.94 mmol, 87%). TLC [petroleum ether/acetone (5:1)]: $R_f$ = 0.42. Mp: 108-109 °C. $^1$H NMR (400 MHz, $CDCl_3$): $\delta$ = 8.63 (s, 1H, 7-*H*), 7.52 (d, 1H, *J* = 3.3 Hz, 2-*H*), 7.17 (d, 1H, *J* = 1.9 Hz, $CH_3OCCHCCH_2$), 7.03 (s, 1H, 4-*H*), 7.02 (dd, 1H, *J* = 8.2 Hz, *J* = 1.9 Hz, $CH_3OCCHCH$), 6.87 (d, 1H, *J* = 8.2 Hz, $CH_3OCCHCH$), 6.45 (d, 1H, *J* = 3.7 Hz, 3-*H*), 5.11 (s, 2H, $CH_2$), 3.93 (s, 3H, $CH_3OCCHCCH_2$), 3.89 (s, 3H, $CH_3OCCHCH$), 1.67 (s, 9H, $C(CH_3)_3$). $^{13}$C NMR (100 MHz, $CDCl_3$): $\delta$ = 152.9 (1C, $COCH_2$), 149.4 (1C, $CH_3OCCHCCH_2$), 149.2 (1C, $CH_3OCCHCH$), 148.7 (1C, NCO), 131.5 (1C, *C*-3a), 131.1 (1C, *C*-7a), 129.4 (1C, $OCH_2C$), 126.7 (1C, *C*-2), 125.8 (1C, *C*-7), 119.4 (1C, $CH_3OCCHCH$), 111.0 (1C, $CH_3OCCHCH$), 110.7 (1C, $CH_3OCCHCCH_2$), 106.9 (1C, *C*-3), 104.1 (1C, *C*-4), 84.0 (1C, $C(CH_3)_3$), 83.4 (1C, *C*-6), 71.4 (1C, $CH_2$), 56.0 (1C, $CH_3OCCHCCH_2$), 55.9 (1C, $CH_3OCCHCH$), 28.1 (3C, $C(CH_3)_3$). IR (diamond ATR): $\tilde{\nu}$ = 3163 (w), 3104 (w), 2978 (w), 2934 (w), 2872 (w), 2836 (w), 1719 (s), 1601 (w), 1573 (w), 1518 (m), 1445 (s), 1420 (m), 1368 (s), 1343 (s), 1273 (s), 1238 (m), 1194 (m), 1161 (m), 1134 (s), 1088 (m), 1023 (s), 966 (w), 934 (w), 884 (w), 858 (w), 837 (m), 823 (m), 805 (m), 764 (s), 721 (m), 674 (w), 625 (w), 582 (w), 556 (m). UV/Vis (MeOH): $\lambda_{max}$ (log $\varepsilon$) = 204 (4.71), 239 (4.48), 273 (4.30), 301 (3.69), 312 (3.68). HRESIMS: calculated ($[C_{22}H_{24}INO_5+Na^+]$) 532.05914, found 532.05936 (0.41 ppm).

***tert*-Butyl 5-((3,4-dimethoxybenzyl)oxy)-6-((trimethylsilyl)ethynyl)-1*H*-indole-1-carboxylate:** Indole **16** (254 mg, 0.5 mmol, 1.00 equiv) and $Pd(PPh_3)_2Cl_2$ (11 mg, 15.0 µmol, 3 mol %) were dried in a Schlenk flask and placed under argon. Dry $NEt_3$ (10 mL) and ethynyltrimethylsilane (118 µL, 1.14 mmol, 2.00 equiv) were added and the mixture was degassed for 15 min. In a separate Schlenk flask CuI (3 mg, 15.0 µmol, 3 mol %) was dried under vacuum, then $NEt_3$ (2 mL) was added and the CuI mixture was degassed for 5 min. The CuI solution was added to the reaction mixture at room temperature followed by THF (2 mL). The reaction mixture was filtered through a pad of Celite 545 which was washed with acetone (100 mL). The solvent was evaporated and the crude product was subjected to column chromatography using petroleum ether/acetone (5:1) as the eluent to give the product as colorless solid (231 mg, 0.48 mmol, 96%). TLC [petroleum ether/acetone (5:1)]: $R_f$ = 0.40. Mp: 155-156 °C. $^1$H NMR (400 MHz, $CDCl_3$): $\delta$ = 8.26 (s, 1H, 7-*H*), 7.57 (d, 1H,

$J$ = 3.5 Hz, 2-$H$), 7.07 (dd, 1H, $J$ = 8.1 Hz, $J$ = 2.0 Hz, $CH_3OCCHCH$), 7.05 (d, 1H, $J$ = 1.8 Hz, $CH_3OCCHCCH_2$), 7.02 (s, 1H, 4-$H$), 6.86 (d, 1H, $J$ = 8.1 Hz, $CH_3OCCHCH$), 6.45 (dd, 1H, $J$ = 3.7 Hz, $J$ = 0.5 Hz, 3-$H$), 5.11 (s, 2H, $CH_2$), 3.90 (s, 3H, $CH_3OCCHCCH_2$), 3.89 (s, 3H, $CH_3OCCHCH$), 1.66 (s, 9H, $C(CH_3)_3$), 0.24 (s, 9H, $Si(CH_3)_3$). $^{13}$C NMR (100 MHz, $CDCl_3$): $\delta$ = 155.7 (1C, $CH_2OC$), 149.4 (1C, NCO), 149.1 (1C, $CH_3OCCHCCH_2$), 148.7 (1C, $CH_3OCCHCH$), 131.6 (1C, $C$-7a), 129.8 (1C, $CH_2C$), 129.6 (1C, $C$-3a), 127.6 (1C, $C$-2), 120.6 (1C, C-7), 119.8 (1C, $CH_3OCCHCH$), 111.0 (1C, $CH_3OCCHCH$), 110.7 (1C, $CH_3OCCHCCH_2$), 110.6 (1C, $C$-6), 107.1 (1C, $C$-3), 104.7 (1C, $C$-4), 102.5 (1C, $C$≡CSi), 97.7 (1C, C≡$C$Si), 83.8 (1C, $C(CH_3)_3$), 71.1 (1C, $CH_2$), 56.0 (2C, $OCH_3$), 28.1 (3C, $C(CH_3)_3$), 0.1 (3C, $Si(CH_3)_3$). IR (diamond ATR): $\tilde{\nu}$ = 3140 (w), 2989 (w), 2955 (w), 2902 (w), 2858 (w), 2831 (w), 2158 (w), 1729 (s), 1619 (w), 1516 (m), 1467 (m), 1448 (m), 1422 (w), 1385 (m), 1370 (m), 1336 (w), 1295 (s), 1247 (m), 1230 (m), 1202 (m), 1185 (w), 1162 (m), 1131 (s), 1079 (w), 1055 (w), 1031 (m), 1008 (w), 933 (w), 883 (m), 839 (s), 806 (m), 760 (s), 728 (w), 694 (w), 674 (w), 634 (w), 588 (w), 554 (w). UV/Vis (MeOH): $\lambda_{max}$ (log $\varepsilon$) = 204 (4.65), 236 (4.50), 291 (4.45), 3.30 (3.80). HRESIMS: calculated ($[C_{27}H_{33}NO_5Si+Na^+]$) 502.20202, found 502.20180 (0.42 ppm).

***tert*-Butyl 5-((3,4-dimethoxybenzyl)oxy)-6-ethynyl-1*H*-indole-1-carboxylate (19):** *tert*-Butyl 5-((3,4-dimethoxybenzyl)oxy)-6-((trimethylsilyl)ethynyl)-1*H*-indole-1-carboxylate (214 mg, 0.45 mmol, 1.00 equiv) was dissolved in THF (11 mL) and cooled to 0 °C. Then a TBAF solution (1 M in THF + 5% $H_2O$, 450 µL, 0.45 mmol, 1.00 equiv) was added and the mixture was stirred for 1 h at 0 °C and 30 min at room temperature. The reaction mixture was diluted with TBME (100 mL) and washed with water (200 mL). The aqueous phase was extracted with TBME (50 mL) and the combined organic phases were washed with brine (50 mL). After drying with $MgSO_4$ and filtration, the solvent was evaporated. The crude product was purified by column chromatography using petroleum ether/acetone (5:1) as the eluent to give the product as colorless solid (152 mg, 0.37 mmol, 83%). TLC [petroleum ether/acetone (5:1)]: $R_f$ = 0.35. Mp: 160-162 °C. $^1$H NMR (400 MHz, $CDCl_3$): $\delta$ = 8.28 (broad s, 1H, 7-$H$), 7.59 (d, 1H, $J$ = 3.4 Hz, 2-$H$), 7.12 (d, 1H, $J$ = 1.9 Hz, $CCHCOCH_3$), 7.03 (s, 1H, 4-$H$), 7.00 (dd, 1H, $J$ = 8.2 Hz, $J$ = 1.9 Hz, $CHCHCOCH_3$), 6.86 (d, 1H, $J$ = 8.2 Hz, $CHCHCOCH_3$), 6.45 (d, 1H, $J$ = 3.6 Hz, 3-$H$), 5.15 (s, 2H, $CH_2$), 3.90 (s, 3H, $CCHCOCH_3$), 3.88 (s, 3H, $CHCHCOCH_3$), 3.29 (s, 1H, C≡C$H$), 1.67 (s, 9H, $C(CH_3)_3$). $^{13}$C NMR (100 MHz, $CDCl_3$): $\delta$ = 155.8 (1C, $COCH_2$), 149.4 (1C, NCO), 149.1 (1C, $CCHCOCH_3$), 148.6 (1C, $CHCHCOCH_3$), 131.9 (1C, $C$-3a), 129.7 (1C, $COCH_2C$), 129.4 (1C, $C$-7a), 127.8 (1C, $C$-2), 120.8 (1C, $C$-7), 119.4 (1C, $CHCHCOCH_3$), 111.0 (1C, $CHCHCOCH_3$), 110.4 (1C, $CCHCOCH_3$), 109.3 (1C, $C$-6), 107.1 (1C, $C$-3), 104.5 (1C, $C$-4), 84.0 (1C, $C(CH_3)_3$), 81.2 (1C, $C$≡CH), 80.3 (1C, C≡$C$H), 71.0 (1C, $CH_2$), 55.9 (1C, $CHCHCOCH_3$), 55.8 (1C,

$CCHCOCH_3$), 28.1 (3C, $C(CH_3)_3$). IR (diamond ATR): $\tilde{\nu}$ = 3249 (m), 3126 (w), 2981 (w), 2941 (w), 2882 (w), 2842 (w), 1733 (s), 1616 (w), 1593 (w), 1573 (w), 1516 (m), 1465 (m), 1443 (s), 1422 (m), 1369 (s), 1338 (w), 1271 (s), 1236 (s), 1198 (m), 1159 (s), 1116 (s), 1022 (s), 990 (s), 890 (w), 873 (m), 840 (s), 812 (m), 785 (w), 759 (s), 714 (m), 644 (s), 597 (w), 568 (m). UV/Vis (MeOH): $\lambda_{max}$ (log $\varepsilon$) = 204 (4.65), 234 (4.46), 284 (4.38), 314 (3.71), 326 (3.69). HRESIMS: calculated ([$C_{24}H_{25}NO_5$+$Na^+$]) 430.16249, found 430.16268 (0.44 ppm).

***tert*-Butyl 6-(5-(1-(*tert*-butoxycarbonyl)-1*H*-indol-6-yl)-3-hydroxy-3-methylpent-1-yn-1-yl)-5-((3,4-dimethoxybenzyl)oxy)-1*H*-indole-1-carboxylate (22):** Alkyne **19** (1.371 g, 3.27 mmol, 2.00 equiv) and ketone **6** (486 mg, 1.69 mmol, 1.00 equiv) were dried in separate Schlenk flasks. The alkyne was dissolved in THF (45 mL), cooled to 0 °C and a solution of iPrMgCl (2 M in THF, 1.69 mL, 3.37 mmol, 2.00 equiv) was added dropwise. After stirring for 10 min ketone **6** dissolved in THF (17 mL) was added within 5 min. The reaction mixture was stirred for 30 min at 0 °C and for 1 h at room temperature. The TLC showed complete consumption of the ketone. The reaction was quenched with saturated $NaHCO_3$ solution (10 mL), diluted with $CH_2Cl_2$ (50 mL) and washed with $H_2O$ (250 mL). The aqueous phase was extracted with $CH_2Cl_2$ (2 x 50 mL each). The combined organic phases were washed with $H_2O$ (250 mL) and brine (150 mL). After drying with $MgSO_4$ and filtration the solvent was evaporated. Purification by column chromatography using petroleum ether/acetone (7:1 to 4:1) as the eluent gave the product as colorless solid (1.112 g, 1.60 mmol, 95%). The alkyne was recovered in good yield (639 mg, 1.57 mmol, 47% of the starting material). TLC [petroleum ether/acetone (5:1)]: $R_f$ = 0.19. Mp: 89 °C. Alkynyl indole atoms are marked with ‘. $^1$H NMR (400 MHz, $CDCl_3$): $\delta$ = 8.26 (s, 1H, 7’-*H*), 8.06 (s, 1H, 7-*H*), 7.59 (d, 1H, *J* = 3.4 Hz, 2’-*H*), 7.51 (d, 1H, *J* = 3.4 Hz, 2-*H*), 7.42 (d, 1H, *J* = 8.0 Hz, 4-*H*), 7.10-7.03 (m, 3H, 4-*H*, 4’-*H*, $CH_3OCCHCH$), 7.00 (d, 1H, *J* = 1.9 Hz, $CH_3OCCHCCH_2$), 6.72 (d, 1H, *J* = 8.2 Hz, $CH_3OCCHCH$), 6.51 (d, 1H, *J* = 3.7 Hz, 3-*H*), 6.47 (d, 1H, *J* = 3.7 Hz, 3’-*H*), 5.11 (s, 2H, $CH_2O$), 3.84 (s, 3H, $CH_3OCCHCCH_2$), 3.76 (s, 3H, $CH_3OCCHCH$), 3.04 (dd, 2H, *J* = 11.1 Hz, *J* = 6.0 Hz, $C(OH)CH_2CH_2$), 2.12 (ddd, 1H, *J* = 6.8 Hz, *J* = 6.6 Hz, *J* = 1.8 Hz, $C(OH)CH_2$), 2.20-2.06 (m, 1H, O*H*), 1.66/1.64 (s, 18H, $C(CH_3)_3$)., 1.63 (s, 3H, $CH_3C(OH)$)). $^{13}$C NMR (100 MHz, $CDCl_3$): $\delta$ = 155.5 (1C, $CH_2O$), 149.8 (1C, NCO), 149.4 (1C, NC’O), 149.0 (1C, $CH_3OCCHCCH_2$), 148.7 (1C, $CH_3OCCHCH$), 138.6 (1C, *C*-6), 135.6 (1C, *C*-7a), 131.5 (1C, *C*-7a’), 129.5 (1C, *C*-3a’), 128.6 (1C, *C*-3a), 127.5 (1C, *C*-2’), 125.4 (1C, *C*-2), 123.6 (1C, *C*-5), 120.6 (1C, *C*-4), 120.1 (1C, *C*-7’), 119.9 (1C, $CH_3OCCHCH$), 114.8 (1C, *C*-7), 111.0 (1C, $CH_3OCCHCH$), 110.7 (1C, $CH_3OCCHCCH_2$), 110.0 (1C, *C*-6’), 107.14 (1C, *C*-3’), 107.11 (1C, *C*-3), 104.4 (1C, *C*-4’), 96.0 (1C, C(OH)*C*≡C), 83.9 (1C, $C(CH_3)_3$), 83.4 (1C, $C(CH_3)_3$), 81.3 (1C, C(OH)C≡*C*), 71.1 (1C, $OCH_2$), 68.8 (1C, *C*(OH), 55.9 (1C, $CH_3OCCHCCH_2$), 55.8 (1C, $CH_3OCCHCH$), 46.2 (1C, $C(OH)CH_2$), 31.9 (1C, $C(OH)CH_2CH_2$), 30.2 (1C, $C(OH)CH_3$), 28.2

(6C, $C(CH_3)_3$). IR (diamond ATR): $\tilde{\nu}$ = 3507 (w), 2977 (w), 2932 (w), 2866 (w), 1730 (s), 1616 (w), 1595 (w), 1517 (m), 1443 (m), 1371 (m), 1339 (m), 1292 (m), 1255 (m), 1212 (m), 1125 (s), 1077 (m), 1026 (m), 997 (m), 930 (w), 843 (m), 806 (w), 764 (m), 725 (m), 691 (w), 655 (w), 556 (w). UV/Vis (MeOH): $\lambda_{max}$ (log $\varepsilon$) = 204 (4.75), 232 (4.71), 286 (4.48), 314 (3.78), 326 (3.76). HRESIMS: calculated ([$C_{41}H_{46}N_2O_8$+$Na^+$]) 717.31464, found 717.31516 (0.73 ppm).

***tert*-Butyl 6-(5-(1-(*tert*-butoxycarbonyl)-1*H*-indol-6-yl)-3-hydroxy-3-methyl-5-oxopent-1-yn-1-yl)-5-hydroxy-1*H*-indole-1-carboxylate (27):** Bisindole **22** (20 mg, 28.30 µmol, 1.00 equiv) was dissolved in $CH_2Cl_2$ (10 mL) and $H_2O$ (1 mL). DDQ (26 mg, 115.21 µmol, 4.00 equiv) was added in one portion at room temperature and the suspension was vigorously stirred for 25 h. Then, the reaction was quenched with saturated $NaHCO_3$ solution (2 mL), diluted with EtOAc (30 mL) and washed with saturated $NaHCO_3$ solution (25 mL). The aqueous phase was extracted with EtOAc (2 x 20 mL) and the combined organic phases were washed with brine (25 mL) and dried with $MgSO_4$. After filtration and evaporation of the solvent the crude product was subjected to column chromatography on silica gel using petroleum ether/acetone (10:1) as the eluent to give the product as yellow oil (3.6 mg, 6.44 µmol, 23%). TLC [petroleum ether/acetone (10:1)]: $R_f$ = 0.08. Alkynyl indole atoms are marked with ‘. $^1$H NMR (600 MHz, $CDCl_3$): $\delta$ = 8.86 (s, 1H, 7-*H*), 8.07 (broad s, 1H, 7’-*H*), 7.95 (1H, dd, *J* = 8.3 Hz, *J* = 1.4 Hz, 5-*H*), 7.79 (d, 1H, *J* = 2.5 Hz, 2-*H*), 7.64 (d, 1H, *J* = 8.3 Hz, 4-*H*), 7.57-7.51 (m, 1H, 2’-*H*), 7.03 (s, 1H, 4’-*H*), 6.63 (d, 1H, *J* = 3.7 Hz, 3-*H*), 6.42 (d, 1H, *J* = 3.6 Hz, 3’-*H*), 6.32 (broad s, 1H, CHCO*H*), 4.45 (broad s, 1H, $CH_3$CO*H*), 3.74 (d, 1H, *J* = 17.1 Hz, $CH_2$), 3.47 (d, 1H, *J* = 17.1 Hz, $CH_2$), 1.75 (s, 3H, $CH_3$COH), 1.72 (s, 9H, $C(CH_3)_3$), 1.63 (s, 9H, $C(CH_3)_3$). $^{13}$C NMR (150 MHz, $CDCl_3$): $\delta$ = 200.5 (1C, *C*(=O)$CH_2$), 153.4 (1C, *C*-5’), 149.4 (1C, N*C*’O), 149.3 (1C, N*C*O), 135.2 (1C, *C*-3a), 134.7 (1C, *C*-7a), 132.7 (1C, *C*-6), 132.6 (1C, *C*-3a’), 129.8 (1C, *C*-2), 129.1 (1C, *C*-7a’), 127.9 (1C, *C*-2’), 122.6 (1C, *C*-5), 121.1 (1C, *C*-4), 117.6 (1C, *C*-7’), 116.4 (1C, *C*-7), 107.2 (1C, *C*-3), 107.0 (1C, *C*-3’), 106.0 (1C, *C*-6’), 105.2 (1C, *C*-4’), 99.0 (1C, C≡*C*C$CH_3$), 84.7 (1C, *C*$(CH_3)_3$), 83.6 (1C, *C*$(CH_3)_3$), 79.0 (1C, *C*≡CC$CH_3$), 66.2 (1C, *C*(OH)$CH_3$), 50.3 (1C, $CH_2$), 29.7 (1C, C(OH)$CH_3$), 28.2 (3C, $C(CH_3)_3$), 28.1 (1C, $C(CH_3)_3$). IR (diamond ATR): $\tilde{\nu}$ = 3416 (w), 3152 (w), 2926 (m), 2854 (w), 2222 (w), 1731 (s), 1633 (m), 1609 (w), 1575 (w), 1522 (w), 1457 (m), 1369 (s), 1340 (s), 1253 (m), 1210 (m), 1153 (s), 1122 (s), 1080 (m), 1011 (m), 905 (w), 888 (w), 837 (m), 809 (w), 764 (s), 727 (m), 551 (m). UV/Vis (MeOH): $\lambda_{max}$ (log $\varepsilon$) = 215 (4.44), 228 (4.43), 287 (4.35). HREIMS: calculated ([$C_{32}H_{34}N_2O_7$+$Na^+$]) 581.22582, found 581.22601 (0.33 ppm).

**2-Iodo-5-methyl-4-nitrophenyl trifluoromethanesulfonate (14):** Phenol **11** (2.00 g, 7.17 mmol, 1.00 equiv) was dried in a Schlenk flask under vacuum and suspended in dry $CH_2Cl_2$ (24 mL). Then dry $NEt_3$ (1.10 mL, 7.89 mmol, 1.10 equiv) was added to give a clear solution. After cooling to 0 °C $Tf_2O$ (1.43 mL, 8.60 mmol, 1.20 equiv) was added within 10 min. The mixture was stirred for 1.5 h at this temperature before the addition of saturated $NaHCO_3$ solution (15 mL). The organic phase was washed with saturated $NaHCO_3$ solution (30 mL), saturated $Na_2S_2O_3$ solution (30 mL) and brine (30 mL) and then dried with $MgSO_4$. After filtration the solvent was evaporated. Purification by column chromatography using petroleum ether/acetone (10:1) as the eluent yielded the product as pale yellow solid (2.83 g, 6.88 mmol, 96%). TLC [petroleum ether/acetone (5:1)]: $R_f$ = 0.45. Mp: 55-57 °C. $^1$H NMR (400 MHz, $CDCl_3$): $\delta$ = 8.48 (s, 1H, C*H*CI), 7.30 (s, 1H, C*H*COTf), 2.61 (s, 3H, C*H*$_3$). $^{13}$C NMR (100 MHz, $CDCl_3$): $\delta$ = 152.4 (1C, *C*OTf), 148.1 (1C, *C*$NO_2$), 136.7 (1C, *C*HCI), 136.5 (1C, *C*$H_3$), 125.5 (1C, *C*HCOTf), 118.6 (1C, q, *J* = 320.8 Hz, *C*$F_3$), 85.3 (1C, *C*I), 20.5 (1C, *C*$H_3$). $^{19}$F NMR (376 MHz, $CDCl_3$): $\delta$ = –73.2. IR (diamond ATR): $\tilde{\nu}$ = 3106 (w), 2998 (w), 2861 (w), 1608 (w), 1556 (w), 1524 (m), 1470 (w), 1426 (m), 1346 (m), 1278 (w), 1204 (s), 1157 (m), 1129 (s), 1035 (w), 1018 (m), 969 (m), 910 (m), 880 (m), 863 (m), 813 (s), 763 (m), 725 (m), 694 (m), 670 (m), 616 (s), 586 (s). UV/Vis (MeOH): $\lambda_{max}$ (log $\varepsilon$) = 202 (4.24), 231 (4.23), 308 (3.21). MS (EI, 70 eV): *m/z* (%) = 410 (75), 393 (100), 277 (98), 261 (20), 247 (49), 219 (19), 203 (14), 188 (4), 175 (12), 162 (3), 147 (7), 126 (14), 93 (25), 68 (98). HREIMS: calculated ([$C_8H_5F_3INO_5S^+$]) 410.88797, found 410.88611 (4.52 ppm).

***tert*-Butyl 6-iodo-5-(((trifluoromethyl)sulfonyl)oxy)-1*H*-indole-1-carboxylate (17):** 2-Iodo-5-methyl-4-nitrophenyl trifluoromethanesulfonate (**14**, 200 mg, 0.49 mmol, 1.00 equiv) was dissolved in DMF (11 mL), DMFDMA (196 µL, 1.46 mmol, 3.00 equiv) was added and the mixture was heated to 110 °C for 4 h. Afterwards the mixture was cooled to 0 °C and an aqueous solution of $NH_4OAc$ (4 M, 2.1 mL, 17.55 equiv) was added followed by the dropwise addition of 20% $TiCl_3$ in 3% HCl (1.88 mL, 2.92 mmol, 6.00 equiv). The mixture was stirred for 45 min at 0°C and then diluted with TBME (20 mL) followed by washing with $H_2O$, saturated $NaHCO_3$ solution and brine (30 mL each). The organic phase was dried with $MgSO_4$ and filtered. After evaporation of the solvent the obtained yellow residue was immediately dissolved in $CH_2Cl_2$ (25 mL). $Boc_2O$ (161 mg, 0.74 mmol, 1.50 equiv) and DMAP (1 mg, 0.01 mmol, 20 mol %) were added at room temperature and the mixture was stirred until TLC analysis showed complete consumption of the starting material. The solvent was evaporated and the residue was purified by column chromatography using petroleum ether/acetone (40:1) as the eluent to give the product as colorless solid (95 mg, 0.19 mmol, 39%). TLC [petroleum ether/EtOAc (30:1)]: $R_f$ = 0.42. Mp: 83-85 °C. $^1$H NMR (400 MHz, $CDCl_3$): $\delta$ = 8.75 (s, 1H, 7-*H*), 7.64 (d, 1H, *J* = 3.7 Hz, 2-*H*), 7.53 (s, 1H, 4-*H*), 6.57 (dd, 1H,

$J$ = 3.7 Hz, $J$ = 0.7 Hz, 3-*H*), 1.68 (s, 9H, C(C$H_3$)$_3$). $^{13}$C NMR (100 MHz, $CDCl_3$): $\delta$ = 148.9 (1C, N*C*O), 145.4 (1C, *C*-5), 134.9 (1C, *C*-7a), 131.3 (1C, *C*-3a), 128.4 (1C, *C*-2), 126.7 (1C, *C*-7), 118.8 (1C, q, $J$ = 320.8 Hz, *C*$F_3$), 113.5 (1C, *C*-4), 106.9 (1C, *C*-3), 85.0 (1C, *C*($CH_3$)$_3$), 82.9 (1C, *C*-6), 28.1 (3C, C(*C*$H_3$)$_3$). $^{19}$F NMR (376 MHz, $CDCl_3$): $\delta$ = –73.5. IR (diamond ATR): $\tilde{\nu}$ = 3178 (w), 3104 (w), 2980 (w), 2938 (w), 1736 (s), 1573 (w), 1524 (w), 1451 (m), 1419 (s), 1370 (s), 1347 (m), 1270 (m), 1242 (m), 1224 (m), 1198 (s), 1127 (s), 1085 (s), 1025 (m), 961 (m), 876 (s), 850 (s), 758 (s), 721 (m), 670 (w), 648 (m), 630 (w), 602 (s), 582 (m), 563 (s). UV/Vis (MeOH): $\lambda_{max}$ (log $\varepsilon$) = 202 (4.31), 233 (4.50), 274 (4.15). MS (EI, 70 eV): *m/z* (%) = 491 (22), 434 (46), 417 (6), 390 (44), 301 (17), 257 (94), 229 (23), 132 (12), 103 (19), 64 (49), 57 (100), 43 (64). HREIMS: calculated ([$C_{14}H_{13}F_3INO_5S^+$]) 490.95057, found 490.95106 (1.00 ppm).

***tert*-Butyl 5-(((trifluoromethyl)sulfonyl)oxy)-6-((trimethylsilyl)ethynyl)-1*H*-indole-1-carboxylate:** Indole **17** (279 mg, 0.57 mmol, 1.00 equiv) was placed in a Schlenk flask with $Pd(PPh_3)_2Cl_2$ (12 mg, 17.1 µmol, 3 mol %) and the flask was evacuated and flushed with argon (3 times). Then dry $NEt_3$ (11 mL) and ethynyltrimethylsilane (118 µL, 0.78 mmol, 1.37 equiv) were added and the mixture was degassed by sonication for 10 min. In a separate flask, CuI was placed under argon (evacuate and flush three times). $NEt_3$ (2 mL) was added and the solution was degassed by sonication for 5 min. The CuI solution was added to the reaction mixture at room temperature. After stirring for 23 h the mixture was filtered through a pad of Celite 545 which was washed with acetone (50 mL). After evaporation of the solvent the crude product was subjected to column chromatography using petroleum ether/EtOAc (30:1) as the eluent to yield the product as colorless oil (249 mg, 0.54 mmol, 95%). TLC [petroleum ether/EtOAc (30:1)]: $R_f$ = 0.22. $^1$H NMR (400 MHz, $CDCl_3$): $\delta$ = 8.41 (s, 1H, 7-*H*), 7.69 (d, 1H, $J$ = 3.7 Hz, 2-*H*), 7.44 (s, 1H, 4-*H*), 6.58 (dd, 1H, $J$ = 3.7 Hz, $J$ = 0.6 Hz, 3-*H*), 1.67 (s, 9H, OC(C$H_3$)$_3$), 0.27 (s, 9H, Si(C$H_3$)$_3$). $^{13}$C NMR (100 MHz, $CDCl_3$): $\delta$ = 148.9 (1C, N*C*O), 145.8 (1C, *C*-5), 133.5 (1C, *C*-7a), 130.9 (1C, *C*-3a), 129.2 (1C, *C*-2), 126.7 (1C, *C*-6), 120.6 (1C, *C*-7), 118.8 (1C, q, $J$ = 320.7 Hz, *C*$F_3$), 113.5 (1C, *C*-4), 107.2 (1C, *C*-3), 101.2 (1C, *C*Si($CH_3$)$_3$), 98.6 (1C, *C*CSi($CH_3$)$_3$), 84.9 (1C, *C*($CH_3$)$_3$), 28.1 (3C, C(*C*$H_3$)$_3$), -0.4 (3C, Si(*C*$H_3$)$_3$). $^{19}$F NMR (376 MHz, $CDCl_3$): $\delta$ = –73.9. IR (diamond ATR): $\tilde{\nu}$ = 2979 (w), 2161 (w), 1741 (m), 1461 (m), 1444 (m), 1424 (m), 1372 (m), 1353 (m), 1286 (m), 1245 (m), 1204 (s), 1140 (s), 1088 (s), 1071 (m), 1027 (w), 1009 (w), 945 (w), 884 (s), 843 (s), 761 (s), 722 (m), 697 (w), 662 (w), 631 (w), 607 (s). UV/Vis (MeOH): $\lambda_{max}$ (log $\varepsilon$) = 202 (4.24), 231 (4.51), 288 (4.33). HRESIMS: calculated ([$C_{19}H_{22}F_3NO_5SSi+Na^+$]) 484.08323, found 484.08319 (0.08 ppm).

***tert*-Butyl 6-ethynyl-5-(((trifluoromethyl)sulfonyl)oxy)-1*H*-indole-1-carboxylate (20):** *tert*-Butyl 5-*O*Tf-6-((trimethylsilyl)ethynyl)-1*H*-indole-1-carboxylate (235 mg, 0.51 mmol, 1.00 equiv) was dissolved in THF (12 mL) and cooled to 0 °C. A 1 M solution of TBAF in THF with 5% $H_2O$ (510 µL, 0.51 mmol, 1.00 equiv) was added and the reaction mixture was stirred for 2 h. The solution was diluted with TBME (50 mL) and washed with $H_2O$ and brine (100 mL each). The organic phase was dried with $MgSO_4$ and filtered. After evaporation of the solvent the product was purified by column chromatography using petroleum ether/EtOAc (40:1) as the eluent to give the product as colorless solid (117 mg, 0.30 mmol, 59%). TLC [petroleum ether/EtOAc (30:1)]: $R_f$ = 0.30. Mp: 86-88 °C. $^1$H NMR (600 MHz, $CDCl_3$): $\delta$ = 8.43 (s, 1H, 7-*H*), 7.73 (d, 1H, *J* = 3.4 Hz, 2-*H*), 7.48 (s, 1H, 4-*H*), 6.60 (dd, 1H, *J* = 3.7 Hz, *J* = 0.7 Hz, 3-*H*), 3.38 (s, 1H, alkynyl-*H*), 1.68 (s, 9H, $C(CH_3)_3$). $^{13}$C NMR (150 MHz, $CDCl_3$): $\delta$ = 148.9 (1C, N*C*O), 145.8 (1C, *C*-5), 133.4 (1C, *C*-7a), 131.3 (1C, *C*-3a), 129.4 (1C, *C*-2), 120.9 (1C, *C*-7), 118.7 (1C, q, *J* = 320.7 Hz, $CF_3$), 113.6 (1C, *C*-4), 112.2 (1C, *C*-6), 107.2 (1C, *C*-3), 85.0 (1C, $C(CH_3)_3$), 83.1 (1C, alkynyl-C*C*H), 77.9 (1C, alkynyl-*C*CH), 28.1 (3C, $C(CH_3)_3$). $^{19}$F NMR (376 MHz, $CDCl_3$): $\delta$ = –73.7. IR (diamond ATR): $\tilde{\nu}$ = 3262 (m), 3166 (w), 3117 (w), 2981 (w), 2940 (w), 1737 (s), 1617 (w), 1524 (w), 1461 (m), 1440 (w), 1415 (s), 1375 (s), 1355 (m), 1316 (w), 1284 (m), 1243 (m), 1203 (s), 1150 (s), 1132 (s), 1086 (s), 1061 (s), 1005 (w), 887 (s), 851 (m), 808 (m), 765 (s), 721 (m), 685 (m), 665 (m), 631 (m), 599 (m), 583 (m), 566 (m). UV/Vis (MeOH): $\lambda_{max}$ (log $\varepsilon$) = 201 (3.79), 233 (4.52), 282 (4.28), 296 (4.08), 308 (3.79). MS (EI, 70 eV): *m/z* (%) = 389 (8), 333 (24), 316 (5), 289 (37), 224 (3), 200 (16), 156 (100), 128 (50), 101 (10), 75 (7), 64 (13), 57 (85). HREIMS: calculated ([$C_{16}H_{14}F_3NO_5S^+$]) 389.05370, found 389.05393 (0.59 ppm).

***tert*-Butyl 6-(5-(1-(*tert*-butoxycarbonyl)-1*H*-indol-6-yl)-3-hydroxy-3-methylpent-1-yn-1-yl)-5-(((trifluoromethyl)sulfonyl)oxy)-1*H*-indole-1-carboxylate (23):** Alkynyl indole **20** (110 mg, 0.28 mmol, 1.10 equiv) and ketone **6** (75 mg, 0.26 mg, 1.00 equiv) were dried in separate Schlenk flasks overnight. Both starting materials were dissolved in THF (5 mL and 3 mL, respectively). The alkyne solution was cooled to 0 °C and a 2 M solution of iPrMgCl in THF (140 µL, 0.28 mmol, 1.10 equiv) was added and the mixture was stirred for 10 min. Then the ketone was added within 5 min and the flask was washed with THF (1 mL). The reaction mixture was stirred at 0 °C for 30 min and then warmed to room temperature. After stirring for further 21 h the reaction was quenched with saturated $NaHCO_3$ solution (2 mL) and diluted with TBME (20 mL). The organic phase was washed with $H_2O$ (50 mL) and the aqueous phase was further extracted with TBME (15 mL). The combined organic phases were washed with $H_2O$ and brine (50 mL each), dried with $MgSO_4$ and filtered. After evaporation of the solvent the crude product was subjected to column chromatography using petroleum ether/acetone (30:1 to 10:1) as the eluent to yield the product as colorless oil

(94 mg, 0.14 mmol, 50%). TLC [petroleum ether/acetone (5:1)]: $R_f$ = 0.33. Alkynyl indole atoms are marked with ‘. $^1$H NMR (600 MHz, $CDCl_3$): $\delta$ = 8.40 (s, 1H, 7’-*H*), 8.08 (s, 1H, 7-*H*), 7.71 (d, 1H, *J* = 3.5 Hz, 2’-*H*), 7.51 (d, 1H, *J* = 3.1 Hz, 2-*H*), 7.47 (d, 1H, *J* = 8.0 Hz, 4-*H*), 7.44 (s, 1H, 4’-*H*), 7.15 (dd, 1H, *J* = 8.0 Hz, *J* = 1.5 Hz, 5-*H*), 6.59 (dd, 1H, *J* = 3.7 Hz, *J* = 0.6 Hz, 3’-*H*), 6.51 (dd, 1H, *J* = 3.7 Hz, *J* = 0.6 Hz, 3-*H*), 3.12-3.01 (m, 2H, C(OH)CHC*H*), 2.34 (s, 1H, O*H*), 2.21-2.12 (m, 2H, C(OH)C*H*), 1.680 (s, 3H, $CH_3$C(OH), 1.675 (s, 9H, $C(CH_3)_3$), 1.65 (s, 9H, $C(CH_3)_3$). $^{13}$C NMR (150 MHz, $CDCl_3$): $\delta$ = 149.8 (1C, N*C*(=O)), 148.9 (1C, N*C*’(=O)), 145.7 (1C, *C*-5’), 138.3 (1C, *C*-6), 135.6 (1C, *C*-7a), 133.6 (1C, *C*-7a’), 130.9 (1C, *C*-3a’), 129.1 (1C, *C*-2’), 128.6 (1C, *C*-3a), 125.4 (1C, *C*-2), 123.6 (1C, *C*-6), 120.7 (1C, *C*-4), 120.1 (1C, *C*-7’), 118.7 (1C, q, *J* = 320.5 Hz, $CF_3$), 114.9 (1C, *C*-7), 113.33 (1C, *C*-6’), 113.32 (1C, *C*-4’), 107.14 (1C, *C*-3’), 107.11 (1C, *C*-3), 98.7 (1C, C(OH)*C*≡C), 84.9 (1C, $C(CH_3)_3$), 83.4 (1C, $C(CH_3)_3$), 78.5 (1C, C(OH)C≡*C*), 68.6 (1C, *C*(OH)), 45.7 (1C, C(OH)$CH_2$), 31.6 (1C, C(OH)$CH_2CH_2$), 29.7 (1C, C(OH)$CH_3$), 28.2 (3C, $C(CH_3)_3$), 28.1 (3C, $C(CH_3)_3$). $^{19}$F NMR (376 MHz, $CDCl_3$): $\delta$ = –74.9. IR (diamond ATR): $\tilde{\nu}$ = 3468 (w), 2980 (w), 2933 (w), 2868 (w), 1743 (s), 1616 (w), 1529 (w), 1464 (w), 1441 (m), 1423 (m), 1372 (m), 1341 (s), 1290 (m), 1246 (m), 1207 (s), 1142 (s), 1087 (s), 1072 (s), 1024 (m), 913 (m), 877 (s), 849 (s), 814 (w), 765 (m), 724 (s), 648 (w), 604 (s), 570 (m). UV/Vis (MeOH): $\lambda_{max}$ (log $\varepsilon$) = 231 (4.70), 267 (4.30), 285 (4.35). HRESIMS: calculated ([$C_{33}H_{35}F_3N_2O_8S+Na^+$]) 699.19584, found 699.19608 (0.34 ppm).

***(E/Z)-tert*-Butyl-6-(5-(1-(*tert*-butoxycarbonyl)-1*H*-indol-6-yl)-3-methylpent-2-enoyl)-5-(((trifluoromethyl)sulfonyl)oxy)-1*H*-indole-1-carboxylate (26):** Bisindole **23** (20 mg, 29.56 µmol, 1.00 equiv) was dried under high vacuum overnight and then dissolved in dry toluene (1.1 mL). $MoO_2(acac)_2$ (1.1 mg, 3.40 µmol, 12 mol %), $AuPPh_3Cl$ (1.7 mg, 3.40 µmol, 12 mol %) and AgOTf (0.9 mg, 3.40 µmol, 12 mol %, weighed in a glove box and transferred to the reaction in an Eppendorf vial) were added in that order at room temperature. The reaction mixture was stirred for 5.5 h until TLC analysis showed complete consumption of the starting material. The reaction was quenched with saturated $NaHCO_3$ (0.5 mL) and diluted with TBME (15 mL). The organic phase was washed with brine (30 mL), dried with $MgSO_4$ and filtered followed by evaporation of the solvent. Purification by column chromatography using petroleum ether/acetone (10:1) as the eluent, afforded the product as colorless oil (18 mg, 26.60 µmol, 90%, 2:1 mixture of *E*/*Z*-isomers). TLC [petroleum ether/acetone (10:1)]: $R_f$ = 0.33. IR (diamond ATR): $\tilde{\nu}$ = 3346 (w), 2925 (w), 2855 (w), 1732 (m), 1658 (w), 1618 (m), 1530 (w), 1479 (w), 1461 (w), 1439 (m), 1419 (m), 1372 (w), 1340 (s), 1247 (m), 1204 (s), 1168 (m), 1141 (s), 1095 (m), 1043 (m), 1022 (m), 931 (w), 885 (s), 848 (m), 815 (m), 764 (s), 724 (s), 602 (s), 565 (m). UV/Vis (MeOH): $\lambda_{max}$ (log $\varepsilon$) = 239 (4.40), 258 (4.41), 285 (4.11),

295 (4.13). HREIMS: calculated ([$C_{33}H_{35}F_3N_2O_8S+Na^+$]) 699.19584, found 699.19617 (0.47 ppm). Atoms of the acylated indole are marked with ‘. *E*-isomer: $^1$H NMR (600 MHz, $CDCl_3$): $\delta$ = 8.55 (broad s, 1H, 7‘-*H*), 8.15-7.99 (m, 1H, 7-*H*), 7.81 (d, 1H, *J* = 3.3 Hz, 2‘-*H*), 7.52 (d, 1H, *J* = 3.5 Hz, 2-*H*), 7.483 (s, 1H, 4‘-*H*), 7.478 (dd, 1H, *J* = 8.2 Hz, *J* = 0.5 Hz, 4-*H*), 7.10 (dd, 1H, *J* = 8.0 Hz, *J* = 1.5 Hz, 5-*H*), 6.642 (dd, 1H, *J* = 3.7 Hz, *J* = 0.7 Hz, 3‘-*H*), 6.61 (dd, 1H, *J* = 2.4 Hz, *J* = 1.1 Hz, C(=O)C*H*CC$H_3$), 6.52 (dd, 1H, *J* = 3.7 Hz, *J* = 0.7 Hz, 3-*H*), 3.01-2.97 (m, 2H, C(C$H_3$)C$H_2$C*H*$_2$), 2.64-2.60 (m, 2H, C(C$H_3$)C*H*$_2$), 2.31 (d, 3H, *J* = 1.2 Hz, C(C*H*$_3$)C$H_2$), 1.69 (s, 9H, alkyl indole C(C*H*$_3$)$_3$), 1.67 (s, 9H, acyl indole C(C*H*$_3$)$_3$). $^{13}$C NMR (150 MHz, $CDCl_3$): $\delta$ = 189.8 (1C, *C*(=O)CH), 161.1 (1C, C(=O)*C*H), 149.8 (1C, alkyl indole N*C*O), 148.9 (1C, acyl indole N*C*O), 142.8 (1C, *C*‘-5), 137.6 (1C, *C*-6), 135.6 (1C, *C*-7a), 133.3 (1C, *C*’-7a), 133.0 (1C, *C*’-3a), 130.3 (1C, *C*’-6), 130.24 (1C, *C*’-2), 128.8 (1C, *C*-3a), 125.6 (1C, *C*-2), 123.3 (1C, *C*-5), 123.2 (1C, C(=O)C*H*), 120.8 (1C, *C*-4), 118.7 (1C, q, *J* = 321.0 Hz, *C*$F_3$), 117.55 (1C, *C*’-7), 114.7 (1C, *C*-7), 107.1 (1C, *C*-3), 107.0 (1C, *C*‘-3), 85.13 (1C, alkyl indole N*C*O), 83.6 (1C, acyl indole N*C*O), 44.0 (1C, C(C$H_3$)*C*$H_2$), 34.6 (1C, C(C$H_3$)C$H_2$*C*$H_2$), 28.2 (3C, acyl indole C(*C*$H_3$)$_3$), 28.10 (3C, alkyl indole C(*C*$H_3$)$_3$), 20.2 (1C, C(*C*$H_3$)C$H_2$). $^{19}$F NMR (376 MHz, $CDCl_3$): $\delta$ = –73.5. *Z*-isomer: $^1$H NMR (600 MHz, $CDCl_3$): $\delta$ = 8.52 (broad s, 1H, 7‘-*H*), 8.15-7.99 (m, 1H, 7-*H*), 7.80 (d, 1H, *J* = 3.7 Hz, 2‘-*H*), 7.50 (d, 1H, *J* = 3.2 Hz, 2-*H*), 7.483 (s, 1H, 4‘-*H*), 7.46 (dd, 1H, *J* = 7.9 Hz, *J* = 0.5 Hz, 4-*H*), 7.25 (d, 1H, *J* = 7.7 Hz, 5-*H*), 6.638 (dd, 1H, *J* = 3.6 Hz, *J* = 0.7 Hz, 3‘-*H*), 6.59-6.58 (m, 1H, C(=O)C*H*CC$H_3$), 6.49 (dd, 1H, *J* = 3.7 Hz, *J* = 0.7 Hz, 3-*H*), 3.01-2.97 (m, 4H, C(C$H_3$)C*H*$_2$C*H*$_2$), 2.02 (d, 3H, *J* = 1.3 Hz, C(C*H*$_3$)C$H_2$), 1.684 (s, 9H, acyl indole C(C*H*$_3$)$_3$), 1.677 (s, 9H, alkyl indole C(C*H*$_3$)$_3$). $^{13}$C NMR (150 MHz, $CDCl_3$): $\delta$ = 189.0 (1C, *C*(=O)CH), 161.7 (1C, C(=O)*C*H), 149.9 (1C, alkyl indole N*C*O), 148.9 (1C, acyl indole N*C*O), 142.7 (1C, *C*’-5), 138.2 (1C, C-6), 135.5 (1C, *C*-7a), 133.3 (1C, *C*’-7a), 132.9 (1C, *C*’-3a), 130.3 (1C, *C*’-6), 130.16 (1C, *C*’-2), 128.7 (1C, *C*-3a), 125.4 (1C, *C*-2), 123.8 (1C, C(=O)*C*H), 123.7 (1C, *C*-5), 120.7 (1C, *C*-4), 117.52 (1C, *C*’-7), 114.9 (1C, *C*-7), 114.42 (1C, *C*’-4), 107.1 (1C, *C*-3), 107.0 (1C, *C*’-3), 85.05 (1C, acyl indole *C*(C$H_3$)$_3$), 83.6 (1C, alkyl indole *C*(C$H_3$)$_3$), 37.2 (1C, C(C$H_3$)*C*$H_2$), 35.0 (1C, C(C$H_3$)C$H_2$*C*$H_2$), 28.2 (3C, alkyl indole C(*C*$H_3$)$_3$), 28.08 (3C, acyl indole C(*C*$H_3$)$_3$), 26.2 (1C, C(*C*$H_3$)C$H_2$). $^{19}$F NMR (376 MHz, $CDCl_3$): $\delta$ = –73.6.

**6-Iodo-1-methyl-1*H*-indole (28):** 6-Iodoindole (**2**, 1000 mg, 4.11 mmol, 1.00 equiv) and KOH (288 mg, 5.14 mmol, 1.25 equiv) were dissolved in EtOH (50 mL). After removal of the solvent under reduced pressure the remaining residue was dissolved in acetone (50 mL) and dimethylsulfate (400 µL, 4.20 mmol, 1.02 equiv) was added. The reaction mixture was stirred for 5 min at room temperature, filtered and concentrated. The crude product was purified by flash column chromatography on silica gel using petroleum ether/EtOAc (15:1) as the eluent

to afford the product as a yellow oil (1029 mg, 4.00 mmol, 97%). TLC [petroleum ether/EtOAc (15:1)]: $R_f$ = 0.68. $^1$H NMR (400 MHz, $CDCl_3$): $\delta$ = 7.68 (dd, 1H, $^4J$ = 1.0 Hz, $^4J$ = 0.8 Hz, 4-*H*), 7.37-7.36 (m, 2H, 5-*H*, 7-*H*), 6.97 (d, 1H, $^3J$ = 3.1 Hz, 2-*H*), 6.44 (dd, 1H, $^3J$ = 3.0 Hz, $^4J$ = 0.8 Hz, 3-*H*), 3.74 (s, 1H, N$CH_3$). $^{13}$C NMR (100 MHz, $CDCl_3$): $\delta$ = 138.0 (1C, *C*-7a), 129.2 (1C, *C*-2), 128.0 (1C, *C*-5), 127.8 (1C, *C*-3a), 122.4 (1C, *C*-7), 118.4 (1C, *C*-4), 101.2 (1C, *C*-3), 85.3 (1C, *C*-6), 32.9 (1C, N$CH_3$). IR (diamond ATR): $\tilde{\nu}$ = 3095 (w), 3054 (w), 2934 (w), 2906 (w), 2808 (w), 1695 (w), 1597 (m), 1557 (w), 1507 (m), 1467 (m), 1447 (m), 1415 (m), 1318 (m), 1298 (m), 1234 (m), 1204 (w), 1138 (w), 1104 (w), 1079 (m), 1045 (w), 1006 (w), 883 (s), 841 (m), 798 (s), 753 (w), 713 (s), 611 (m), 585 (s), 547 (w). UV/Vis (MeOH): $\lambda_{max}$ (log $\varepsilon$) = 288 (3.92), 231 (4.65), 202 (4.35). MS (EI, 70 eV): *m*/*z* (%) = 257 (100), 130 (22), 115 (3), 103 (12), 77 (9). HRGCMS: calculated ([$C_9H_8IN^+$]) 256.97014, found 256.96935 (3.08 ppm).

**(1-Methyl-1*H*-indol-6-yl)-(2,6,6-trimethylcyclohex-1-en-1-yl)-methanol (32):** *N*-Methyl-6-iodoindole (**28**, 500 mg, 1.95 mmol, 1.00 equiv) was dissolved in dry THF (25 mL) and cooled to 0 °C under $N_2$. A 2 M solution of iPrMgCl in THF (1.20 mL, 2.40 mmol, 1.23 equiv) was added slowly and the solution was stirred for 2 h at room temperature. β-Cyclocitral (**30**, 320 µL, 1.95 mmol, 1.00 equiv) was added and the reaction mixture was stirred again for 4 h at room temperature. After removal of the solvent under reduced pressure the precipitate was dissolved in TBME (40 mL) and washed with water (30 mL) and brine (30 mL). After drying with $MgSO_4$ and filtration the solution was concentrated. Flash column chromatography on silica gel using petroleum ether/EtOAc (8:1), primed with petroleum ether/EtOAc/$NEt_3$ (80:10:1) as the eluent afforded the product as a slightly yellow solid (353 mg, 1.25 mmol, 64%). TLC [petroleum ether/EtOAc (8:1)]: $R_f$ = 0.17. Mp: 124 °C. $^1$H NMR (400 MHz, acetone-$d_6$): $\delta$ = 7.53 (s, 1H, 7-*H*), 7.44 (d, 1H, $^3J$ = 8.4 Hz, 4-*H*), 7.13 (d, 1H, $^3J$ = 3.1 Hz, 2-*H*), 7.10 (ddd, 1H, $^3J$ = 8.4 Hz, $^4J$ = 1.5 Hz, $^5J$ = 0.8 Hz, 5-*H*), 6.36 (dd, 1H, $^3J$ = 3.0 Hz, $^5J$ = 0.9 Hz, 3-*H*), 5.58 (d, 1H, $^3J$ = 4.8 Hz, C*H*OH), 3.95 (d, 1H, $^3J$ = 4.8 Hz, CHO*H*), 3.79 (s, 3H, N$CH_3$), 1.99 (t, 2H, $^3J$ = 6.3 Hz, C=C-$CH_2$), 1.74-1.61 (m, 2H, $CH_2CH_2CH_2$), 1.59-1.47 (m, 2H, $C(CH_3)_2CH_2$), 1.43 (s, 3H, C=C-$CH_3$), 1.17 (s, 3H, $C(CH_3)_2$), 1.12 (s, 3H, $C(CH_3)_2$). $^{13}$C NMR (100 MHz, acetone-$d_6$): $\delta$ = 141.4 (1C, $CH_2$-C=*C*), 140.5 (1C, *C*-6), 138.0 (1C, *C*-7a), 133.0 (1C, $CH_2$-*C*=C), 129.6 (1C, *C*-2), 127.9 (1C, *C*-3a), 120.5 (1C, *C*-4), 118.9 (1C, *C*-5), 107.5 (1C, *C*-7), 101.0 (1C, *C*-3), 71.3 (1C, *C*HOH), 41.0 (1C, $C(CH_3)_2CH_2$), 35.5 (1C, $C(CH_3)_2$), 34.3 (1C, C=C-$CH_2$), 32.7 (1C, N$CH_3$), 29.4 (1C, $C(CH_3)_2$), 29.2 (1C, $C(CH_3)_2$), 22.0 (1C, C=C-$CH_3$), 20.2 (1C, $CH_2CH_2CH_2$). IR (diamond ATR): $\tilde{\nu}$ = 3546 (m), 3004 (w), 2938 (m), 2906 (m), 1616 (w), 1557 (w), 1508 (m), 1461 (s), 1413 (w), 1363 (w), 1341 (w), 1321 (m), 1303 (m), 1236 (m), 1176 (w), 1131 (w),

1114 (w), 1075 (m), 1037 (m), 1012 (w), 989 (s), 942 (w), 897 (w), 864 (w), 848 (w), 809 (s), 767 (m), 755 (m), 713 (s), 674 (w), 610 (w), 558 (m). UV/Vis (MeOH): $\lambda_{max}$ (log $\varepsilon$) = 283 (3.85), 277 (3.86), 225 (4.58), 205 (4.40). HRESIMS: calculated ([$C_{19}H_{25}NONa^+$]) 306.18284, found 306.18297 (0.42 ppm).

**1,4b,8,8-Tetramethyl-1,4b,5,6,7,8-hexahydroindeno[1,2-*f*]indole (34) and 1,7,7,10a-tetramethyl-1,7,8,9,10,10a-hexahydroindeno[2,1-*g*]indole (35):** (1-Methyl-1*H*-indol-6-yl)-(2,6,6-trimethylcyclohex-1-en-1-yl)-methanol (**32**, 110 mg, 0.39 mmol, 1.00 equiv) was dissolved in dry DCM (15 mL) under $N_2$ and cooled to 0 °C. A 1.0 M solution of $SnCl_4$ in DCM (580 µL, 0.58 mmol, 1.50 equiv) was added slowly and the reaction mixture was stirred for 5 min at 0 °C, followed by 1 h at room temperature. Water (0.5 mL) was added and the solvent was removed under reduced pressure. The precipitate was dissolved in TBME (20 mL), washed with water (2 x 15 mL), saturated $NaHCO_3$, (15 mL), water (15 mL) and brine (15 mL). Then it was dried with $MgSO_4$, filtered and concentrated. Flash column chromatography on silica gel using petroleum ether/EtOAc (50:1) as the elunet afforded the products as colorless liquids (33 mg, 0.12 mmol, 32%, combined yield). TLC [petroleum ether/EtOAc (50:1)]: $R_f$ = 0.26. **37:** $^1$H NMR (600 MHz, $CDCl_3$): $\delta$ = 7.43 (s, 1H, 4-*H*), 7.17 (s, 1H, 10-*H*), 6.96 (d, 1H, $^3J$ = 3.1 Hz, 2-*H*), 6.44-6.42 (m, 1H, 3-*H*), 6.44 (s, 1H, 9-*H*), 3.76 (s, 3H, $NCH_3$), 2.21-2.16 (m, 1H, 5-$H_{(equiv)}$), 2.02-1.93 (m, 1H, 6-$H_{(ax.)}$), 1.71-1.58 (m, 2H, 6-$H_{(equiv)}$, 7-$H_{(equiv)}$), 1.42 (s, 3H, $CH_2CCH_3$), 1.31 (s, 3H, $C(CH_3)_{2,equiv}$, 1.26 (s, 3H, $C(CH_3)_{2,ax.}$), 1.21-1.10 (m, 1H, 7-$H_{(ax.)}$), 1.08-1.01 (m, 1H, 5-$H_{(ax.)}$). $^{13}$C NMR (150 MHz, $CDCl_3$): $\delta$ = 163.1 (1C, *C*-8a), 147.8 (1C, *C*-4a), 137.6 (1C, *C*-9a), 136.3 (1C, *C*-10a), 127.7 (1C, *C*-2), 125.9 (1C, *C*-3a), 121.1 (1C, *C*-9), 112.9 (1C, *C*-4), 100.8 (1C, *C*-3), 100.7 (1C, *C*-10), 49.8 (1C, *C*-4b), 42.7 (1C, *C*-7), 38.9 (1C, *C*-5), 35.5 (1C, *C*-8), 32.9 (1C, $NCH_3$), 31.4 (1C, $C(CH_3)_{2,equiv}$, 25.5 (1C, $C(CH_3)_{2,ax.}$), 24.7 (1C, $CH_2CCH_3$), 20.0 (1C, *C*-6). GCMS (EI, 70 eV): *m*/*z* (%) = 265 (100), 250 (95), 235 (25), 220 (10), 209 (28), 196 (98), 181 (20), 167 (7). **35:** $^1$H NMR (600 MHz, $CDCl_3$): $\delta$ = 7.47 (d, 1H, $^3J$ = 7.8 Hz, 4-*H*), 7.10 (d, 1H, $^3J$ = 7.8 Hz, 5-*H*), 6.90 (d, 1H, $^3J$ = 3.1 Hz, 2-*H*), 6.49 (d, 1H, $^3J$ = 3.2 Hz, 3-*H*), 6.43 (s, 1H, 6-*H*), 4.07 (s, 3H, $NCH_3$), 2.68-2.63 (m, 1H, 10-$H_{(equiv)}$), 2.08-1.98 (m, 1H, 9-$H_{(ax.)}$), 1.71-1.58 (m, 2H, 8-$H_{(equiv)}$, 9-$H_{(equiv)}$), 1.55 (s, 3H, $CH_2CCH_3$), 1.35-1.28 (m, 1H, 10-$H_{(ax.)}$), 1.33 (s, 3H, $C(CH_3)_{2,equiv}$, 1.28 (s, 3H, $C(CH_3)_{2,ax.}$), 1.21-1.10 (m, 1H, 8-$H_{(ax.)}$). $^{13}$C NMR (150 MHz, $CDCl_3$): $\delta$ = 160.5 (1C, *C*-6a), 137.7 (1C, *C*-5a), 137.0 (1C, *C*-10b), 133.2 (1C, *C*-10c), 130.1 (1C, *C*-2), 128.9 (1C, *C*-3a), 121.9 (1C, *C*-6), 119.7 (1C, *C*-4), 114.0 (1C, *C*-5), 102.5 (1C, *C*-3), 52.1 (1C, *C*-10a), 42.0 (1C, *C*-8), 38.6 (1C, $NCH_3$), 38.0 (1C, *C*-10), 35.3 (1C, *C*-7), 31.7 (1C, $C(CH_3)_{2,equiv}$, 25.8 (1C, $C(CH_3)_{2,ax.}$), 23.1 (1C, $CH_2CCH_3$), 19.9 (1C, *C*-9). GCMS (EI, 70 eV): *m*/*z* (%) = 265 (87), 250 (100), 235 (30), 220 (11), 209 (18), 196 (34), 181 (13), 167 (7). **34+35**: IR (diamond ATR,

1:2-mixture): $\tilde{\nu}$ = 3098 (w), 3058 (w), 2956 (m), 2923 (s), 2864 (m), 2844 (m), 1593 (w), 1526 (w), 1508 (w), 1461 (m), 1438 (m), 1407 (w), 1389 (w), 1365 (w), 1321 (m), 1304 (w), 1278 (w), 1228 (w), 1210 (w), 1181 (w), 1112 (w), 1084 (w), 1023 (w), 981 (w), 965 (w), 938 (w), 863 (s), 802 (s), 718 (s), 696 (s), 670 (s), 652 (m), 592 (w), 547 (w). UV/Vis (MeOH, 1:2-mixture): $\lambda_{max}$ (log $\varepsilon$) = 306 (4.08), 298 (4.09), 252 (4.44), 246 (4.43), 206 (4.25).

**6-Iodo-1-(triisopropylsilyl)-1*H*-indole (29):** 6-Iodo-1*H*-indole (100 mg, 0.41 mmol, 1.00 equiv) was dissolved in dry THF (10 mL) under $N_2$ and cooled to −78 °C. At this temperature a 1.0 M solution of LHMDS in THF (450 µL, 0.45 mmol, 1.10 equiv) was added and the reaction was stirred for 30 min before TIPSCl (95 µL, 0.43 mmol, 1.05 equiv) was added. After stirring for 20 min at −78 °C and 1 h at room temperature the reaction was quenched with saturated $NH_4Cl$ (1 mL). Water (5 mL) was added and the reaction mixture was extracted with EtOAc (3 x 10 mL), dried, filtered and concentrated. Flash column chromatography on silica gel using petroleum ether, primed with petroleum ether/$NEt_3$ (100:1) as the eluent afforded the product as colorless oil (152 mg, 0.38 mmol, 93%). TLC [petroleum ether]: $R_f$ = 0.41. $^1$H NMR (400 MHz, $CDCl_3$): $\delta$ = 7.83 (d, 1H, $^4J$ = 1.0 Hz, 7-*H*), 7.38-7.37 (m, 2H, 5-*H*, 4-*H*), 7.17 (d, 1H, $^3J$ = 3.2 Hz, 2-*H*), 6.58 (dd, 1H, $^3J$ = 3.2 Hz, $^5J$ = 0.9 Hz, 3-*H*), 1.66 (sept, 3H, $^3J$ = 7.6 Hz, C*H*($CH_3$)$_2$), 1.14 (d, 18H, $^3J$ = 7.6 Hz, CH(C*H*$_3$)$_2$). $^{13}$C NMR (100 MHz, $CDCl_3$): $\delta$ = 142.2 (1C, *C*-7a), 131.6 (1C, *C*-2), 130.7 (1C, *C*-3a), 128.5 (1C, *C*-5), 122.7 (1C, *C*-7), 122.1 (1C, *C*-4), 104.8 (1C, *C*-3), 85.3 (1C, *C*-6), 18.1 (6C, CH(*C*H$_3$)$_2$), 12.8 (3C, *C*H($CH_3$)$_2$). IR (diamond ATR): $\tilde{\nu}$ = 2946 (m), 2891 (w), 2867 (m), 1595 (w), 1505 (w), 1450 (m), 1423 (m), 1388 (w), 1367 (w), 1315 (w), 1273 (m), 1142 (s), 1075 (m), 1015 (m), 995 (m), 977 (m), 922 (w), 881 (s), 803 (s), 775 (w), 722 (m), 682 (s), 647 (s), 604 (m), 584 (s), 562 (m). UV/Vis (MeOH): $\lambda_{max}$ (log $\varepsilon$) = 278 (3.93), 231 (4.60), 202 (4.27). MS (EI, 70 eV): *m*/*z* (%) = 399 (100), 356 (60), 328 (18), 314 (7), 300 (9), 286 (13), 242 (7), 229 (72), 214 (17), 201 (15), 186 (29), 172 (12), 159 (10), 144 (17), 117 (9). HRGCMS: calculated ([$C_{17}H_{26}INSi^+$]) 399.08792, found 399.08559 (5.84 ppm).

**(1-Triisopropylsilyl-1*H*-indol-6-yl)-(2,6,6-trimethylcyclohex-1-en-1-yl)-methanol (33):** *N*-Triisopropylsilyl-6-iodoindole (**29**, 152 mg, 0.38 mmol, 1.00 equiv) was dissolved in dry THF (20 mL) and cooled to 0 °C under $N_2$. A 2 M solution of iPrMgCl in THF (290 µL, 0.58 mmol, 1.50 equiv) was added slowly and the solution was stirred for 2 h at room temperature. β-Cyclocitral (**30**, 75 µL, 0.42 mmol, 1.10 equiv) was added and the reaction mixture stirred overnight at room temperature. After quenching the reaction with water (10 mL) it was extracted with TBME (3 x 20 mL), washed with water (20 mL) and brine (20 mL), dried, filtered and concentrated. Flash column chromatography on silica gel using petroleum

ether/EtOAc (20:1), primed with petroleum ether/EtOAc/$NEt_3$ (200:10:2) as the eluent afforded the product as a colorless liquid (78 mg, 0.18 mmol, 48%). TLC [petroleum ether/EtOAc (20:1)]: $R_f$ = 0.21. $^1$H NMR (400 MHz, acetone-$d_6$): $\delta$ = 7.53 (s, 1H, 7-*H*), 7.52 (d, 1H, $^3J$ = 8.1 Hz, 4-*H*), 7.36 (ddd, 1H, $^3J$ = 8.4 Hz, $^4J$ = 1.4 Hz, $^5J$ = 0.6 Hz, 5-*H*), 7.30 (d, 1H, $^3J$ = 3.2 Hz, 2-*H*), 6.56 (dd, 1H, $^3J$ = 3.2 Hz, $^5J$ = 0.9 Hz, 3-*H*), 5.57 (d, 1H, $^3J$ = 4.6 Hz, C*H*OH), 4.04 (d, 1H, $^3J$ = 4.7 Hz, CHO*H*), 2.00 (t, 2H, $^3J$ = 6.4 Hz, C=C-C$H_2$), 1.76 (sept, 3H, $^3J$ = 7.6 Hz, C*H*($CH_3$)$_2$), 1.73-1.62 (m, 2H, $CH_2$C$H_2$$CH_2$), 1.57-1.50 (m, 2H, C($CH_3$)$_2$C$H_2$), 1.44 (s, 3H, C=C-C$H_3$), 1.18 (s, 3H, C(C$H_3$)$_2$), 1.15 (d, 18H, $^3J$ = 7.5 Hz, CH(C$H_3$)$_2$), 1.08 (s, 3H, C(C$H_3$)$_2$). $^{13}$C NMR (100 MHz, acetone-$d_6$): $\delta$ = 141.7 (1C, $CH_2$-C=*C*), 141.4 (1C, *C*-6), 140.2 (1C, *C*-7a), 132.9 (1C, $CH_2$-*C*=C), 131.7 (1C, *C*-2), 130.7 (1C, *C*-3a), 120.5 (1C, *C*-4), 119.9 (1C, *C*-5), 112.2 (1C, *C*-7), 105.4 (1C, *C*-3), 71.3 (1C, *C*HOH), 41.0 (1C, C($CH_3$)$_2$*C*$H_2$), 35.4 (1C, *C*($CH_3$)$_2$), 34.4 (1C, C=C-$CH_2$), 29.3 (1C, C(*C*$H_3$)$_2$), 29.0 (1C, C(*C*$H_3$)$_2$), 22.2 (1C, C=C-*C*$H_3$), 20.2 (1C, $CH_2$*C*$H_2$$CH_2$), 18.5 (6C, CH(*C*$H_3$)$_2$), 13.6 (3C, *C*H($CH_3$)$_2$). IR (diamond ATR): $\tilde{\nu}$ = 3608 (w), 3499 (w), 2948 (m), 2924 (m), 2865 (m), 1616 (w), 1555 (w), 1509 (w), 1463 (m), 1431 (m), 1365 (w), 1317 (w), 1277 (m), 1235 (w), 1206 (w), 1167 (m), 1139 (m), 1076 (w), 1020 (m), 994 (m), 973 (m), 923 (m), 882 (s), 837 (w), 809 (m), 789 (w), 771 (w), 758 (w), 712 (s), 689 (s), 648 (s), 601 (m), 574 (m). UV/Vis (MeOH): $\lambda_{max}$ (log $\varepsilon$) = 271 (3.89), 227 (4.56), 203 (4.39). HRESIMS: calculated ([$C_{27}H_{43}NOSiNa^+$]) 448.30061, found 448.30084 (0.51 ppm).

**4b,8,8-Trimethyl-1,4b,5,6,7,8-hexahydroindeno[1,2-*f*]indole (36):** (1-Triisopropylsilyl-1*H*-indol-6-yl)-(2,6,6-trimethylcyclohex-1-en-1-yl)-methanol (**33**, 50 mg, 0.12 mmol, 1.00 equiv) was dissolved in dry DCM (5 mL) under $N_2$ and cooled to 0 °C. A 1.0 M solution of $SnCl_4$ in DCM (180 µL, 0.18 mmol, 1.50 equiv) was added slowly and the reaction mixture was stirred for 5 min at 0 °C, followed by 4 h at room temperature. A saturated solution of $NaHCO_3$ (0.5 mL) was added to quench the reaction. After dilution with water (5 mL) and DCM (5 mL) the organic phase was washed with saturated $NaHCO_3$ (2 x 10 mL), water (10 mL) and brine (10 mL), dried, filtered and concentrated. Flash column chromatography on silica gel using petroleum ether/EtOAc (100:1), gradient to (20:1) afforded the product as a colorless oil (2 mg, 0.01 mmol, 7%). TLC [petroleum ether/EtOAc (20:1)]: $R_f$ = 0.23. $^1$H NMR (400 MHz, $CDCl_3$): $\delta$ = 8.05 (broad s, 1H, N*H*), 7.45 (d, 1H, $^4J$ = 0.5 Hz, 4-*H*), 7.24 (s, 1H, 10-*H*), 7.12 (dd, 1H, $^3J$ = 3.1 Hz, $^3J$ = 0.7 Hz, 2-*H*), 6.51-6.50 (m, 1H, 3-*H*), 6.41 (s, 1H, 9-*H*), 2.23-2.15 (m, 1H, 5-$H_{(equiv)}$), 1.98 (qt, 1H, $^3J$ = 13.6 Hz, $^3J$ = 3.5 Hz, 6-$H_{(ax.)}$), 1.69-1.63 (m, 1H, 7-$H_{(equiv)}$), 1.63-1.57 (m, 1H, 6-$H_{(equiv)}$), 1.42 (s, 3H, $CH_2$CC$H_3$), 1.31 (s, 3H, C(C$H_3$)$_{2,equiv}$), 1.26 (s, 3H, C(C$H_3$)$_{2,ax.}$), 1.14 (dt, 1H, $^3J$ = 13.5 Hz, $^3J$ = 4.3 Hz, 7-$H_{(ax.)}$), 1.10-1.01 (m, 1H, 5-$H_{(ax.)}$). $^{13}$C NMR (100 MHz, $CDCl_3$): $\delta$ = 163.2 (1C, *C*-8a), 148.5 (1C, *C*-4a), 138.1 (1C, *C*-9a), 135.3

(1C, *C*-10a), 125.4 (1C, *C*-3a), 123.1 (1C, *C*-2), 121.1 (1C, *C*-9), 112.8 (1C, *C*-4), 102.6 (1C, *C*-3), 102.4 (1C, *C*-10), 49.9 (1C, *C*-4b), 42.8 (1C, *C*-7), 38.9 (1C, *C*-5), 35.5 (1C, *C*-8), 31.3 (1C, $C(CH_3)_{2,equiv)}$, 25.5 (1C, $C(CH_3)_{2,ax.}$), 24.7 (1C, $CH_2CCH3$), 20.1 (1C, *C*-6). IR (diamond ATR): $\tilde{\nu}$ = 3400 (s), 3100 (w), 3052 (w), 2996 (w), 2959 (m), 2927 (m), 2908 (m), 2863 (w), 2842 (w), 1677 (w), 1627 (w), 1593 (w), 1493 (w), 1461 (m), 1414 (w), 1361 (m), 1303 (w), 1283 (w), 1261 (w), 1233 (w), 1207 (w), 1179 (w), 1142 (w), 1098 (m), 1065 (w), 1016 (m), 967 (w), 938 (w), 879 (m), 868 (m), 834 (w), 786 (m), 769 (w), 738 (s), 702 (m), 671 (m), 613 (w), 545 (w). UV/Vis (MeOH):$\lambda_{max}$ (log $\varepsilon$) = 318 (3.68), 292 (4.25), 252 (4.07), 235 (4.32). MS (EI, 70 eV): *m*/*z* (%) = 251 (99), 236 (100), 221 (26), 204 (19), 182 (100), 167 (27), 154 (8), 139 (5). HRGCMS: calculated ($[C_{18}H_{21}N^+]$) 251.16685, found 251.16833 (5.89 ppm).

**6-Iodo-1-(triisopropylsilyl)indoline (39):** 6-Iodoindoline (**38**, 265 mg, 1.08 mmol, 1.00 equiv) was dissolved in dry THF (15 mL) under $N_2$ and cooled to −78 °C. At this temperature a 1.0 M solution of LHMDS in THF (1.20 mL, 1.19 mmol, 1.10 equiv) was added and the reaction stirred for 30 min, before TIPSCl (240 µL, 1.14 mmol, 1.05 equiv) was added. After stirring for 20 min at −78 °C and 1 h at room temperature the reaction was quenched with saturated $NH_4Cl$ (2 mL). Water (10 mL) was added and the reaction mixture was extracted with EtOAc (3 x 15 mL), dried, filtered and concentrated. Flash column chromatography on silica gel using petroleum ether, primed with petroleum ether/$NEt_3$ (100:1) as the eluent afforded the product as a colorless solid (398 mg, 0.99 mmol, 92%). TLC [petroleum ether]: $R_f$ = 0.43. Mp: 68 °C. $^1$H NMR (400 MHz, $CDCl_3$): $\delta$ = 6.92 (d, 1H, $^4J$ = 1.4 Hz, 7-*H*), 6.90 (dd, 1H, $^3J$ = 7.5 Hz, $^4J$ = 1.5 Hz, 5-*H*), 6.77 (dt, 1H, $^3J$ = 7.6 Hz, $^4J$ = 1.1 Hz, 4-*H*), 3.69 (t, 2H, $^3J$ = 8.7 Hz, 2-*H*), 2.93 (dt, 2H, $^3J$ = 8.6 Hz, $^4J$ = 1.0 Hz, 3-*H*), 1.40 (sept, 3H, $^3J$ = 7.5 Hz, $CH(CH_3)_2$), 1.13 (d, 18H, $^3J$ = 7.5 Hz, $CH(CH_3)_2$). $^{13}$C NMR (100 MHz, $CDCl_3$): $\delta$ = 154.7 (1C, *C*-7a), 131.7 (1C, *C*-3a), 125.8 (1C, *C*-5), 125.6 (1C, *C*-4), 118.8 (1C, *C*-7), 91.9 (1C, *C*-6), 50.9 (1C, *C*-2), 29.9 (1C, *C*-3), 18.4 (6C, $CH(CH_3)_2$), 12.8 (3C, $CH(CH_3)_2$). IR (diamond ATR): $\tilde{\nu}$ = 2940 (m), 2864 (m), 1594 (m), 1563 (w), 1465 (s), 1401 (m), 1355 (w), 1324 (m), 1293 (w), 1243 (m), 1225 (m), 1169 (w), 1110 (w), 1070 (w), 1038 (m), 1013 (w), 990 (w), 957 (s), 919 (w), 879 (s), 828 (m), 792 (m), 732 (w), 684 (s), 642 (s), 578 (s). UV/Vis (MeOH): $\lambda_{max}$ (log $\varepsilon$) = 317 (3.59), 265 (4.12), 222 (4.45). MS (EI, 70 eV): *m*/*z* (%) = 401 (61), 358 (100), 316 (16), 274 (4), 231 (56), 203 (6), 188 (26), 160 (11), 117 (8), 59 (13). HRGCMS: calculated ($[C_7H_{28}INSi^+]$) 401.10357, found 401.10092 (6.60 ppm).

**(1-Triisopropylsilyl-1*H*-indolin-6-yl)-(2,6,6-trimethylcyclohex-1-en-1-yl)methanol (40):** *N*-Triisopropylsilyl-6-iodoindoline (**39**, 400 mg, 1.00 mmol, 1.00 equiv) was dissolved in dry THF (10 mL) and cooled to 0 °C under $N_2$. A 2 M solution of iPrMgCl in THF (750 µL, 1.50 mmol, 1.50 equiv) was added slowly and the solution was stirred for 1 h at room temperature. β-Cyclocitral (**30**, 200 µL, 1.10 mmol, 1.10 equiv) was added and the reaction was stirred overnight at room temperature. After quenching the reaction with water (10 mL) it was extracted with TBME (3 x 20 mL), washed with water (20 mL) and brine (20 mL), dried, filtered and concentrated. Flash column chromatography on silica gel using petroleum ether/EtOAc (20:1), primed with petroleum ether/EtOAc/$NEt_3$ (200:10:2) as the eluent afforded the product as a colorless solid (288 mg, 0.67 mmol, 68%). TLC [petroleum ether/EtOAc (20:1)]: $R_f$ = 0.21. Mp: 96 °C. $^1$H NMR (400 MHz, acetone-$d_6$): $\delta$ = 6.97 (d, 1H, $^3J$ = 7.5 Hz, 4-*H*), 6.86 (d, 1H, $^3J$ = 7.4 Hz, 5-*H*), 6.65 (s, 1H, 7-*H*), 5.32 (d, 1H, $^3J$ = 4.9 Hz, C*H*OH), 3.83 (d, 1H, $^3J$ = 4.9 Hz, CHO*H*), 3.73 (t, 2H, $^3J$ = 8.6 Hz, 2-*H*), 2.94 (t, 2H, $^3J$ = 8.5 Hz, 3-*H*), 2.00-1.86 (m, 2H, C=C-C$H_2$), 1.69-1.60 (m, 2H, $CH_2CH_2CH_2$), 1.59-1.47 (m, 2H, $C(CH_3)_2CH_2$), 1.47 (sept, 3H, $^3J$ = 7.6 Hz, C*H*$(CH_3)_2$), 1.43 (s, 3H, C=C-C$H_3$), 1.15 (s, 3H, $C(CH_3)_2$), 1.14 (d, 18H, $^3J$ = 7.5 Hz, $CH(CH_3)_2$), 1.07 (s, 3H, $C(CH_3)_2$). $^{13}$C NMR (100 MHz, acetone-$d_6$): $\delta$ = 153.1 (1C, *C*-7a), 146.2 (1C, *C*-6), 141.4 (1C, $CH_2$-C=*C*), 132.9 (1C, $CH_2$-*C*=C), 130.1 (1C, *C*-3a), 124.3 (1C, *C*-4), 116.3 (1C, *C*-5), 109.0 (1C, *C*-7), 71.0 (1C, *C*HOH), 51.6 (1C, *C*-2), 40.8 (1C, $C(CH_3)_2CH_2$), 35.4 (1C, *C*$(CH_3)_2$), 34.4 (1C, C=C-$CH_2$), 30.9 (1C, *C*-3), 29.3 (1C, $C(CH_3)_2$), 29.0 (1C, $C(CH_3)_2$), 21.9 (1C, C=C-$CH_3$), 20.2 (1C, $CH_2CH_2CH_2$), 18.9 (6C, $CH(CH_3)_2$), 13.8 (3C, *C*H$(CH_3)_2$). IR (diamond ATR): $\tilde{\nu}$ = 3606 (w), 3508 (w), 2924 (m), 2861 (m), 1607 (m), 1489 (m), 1460 (m), 1419 (m), 1360 (w), 1324 (w), 1254 (s), 1229 (m), 1176 (w), 1114 (w), 1042 (m), 1018 (w), 990 (m), 962 (s), 923 (w), 881 (m), 857 (m), 834 (m), 804 (m), 773 (w), 722 (m), 683 (s), 638 (s), 585 (m), 567 (m). UV/Vis (MeOH): $\lambda_{max}$ (log $\varepsilon$) = 310 (3.52), 262 (4.01), 212 (4.38). HRESIMS: calculated ($[C_{27}H_{45}NOSiNa^+]$) 450.31626, found 450.31662 (0.80 ppm).

**4b,8,8-Trimethyl-1,2,3,4b,5,6,7,8-octahydroindeno[1,2-*f*]indole (41):** (1-Triisopropylsilyl-1*H*-indolin-6-yl)-(2,6,6-trimethylcyclohex-1-en-1-yl)methanol (**40**, 60 mg, 0.14 mmol, 1.00 equiv) was dissolved in dry DCM (5 mL) under $N_2$ and cooled to 0 °C. A 1.0 M solution of $SnCl_4$ in DCM (210 µL, 0.21 mmol, 1.50 equiv) was added slowly and the reaction was stirred for 5 min at 0 °C, followed by 4 h at room temperature. A saturated solution of $NaHCO_3$ (0.5 mL) was added to quench the reaction. After dilution with water (5 mL) and DCM (5 mL) the organic phase was washed with saturated $NaHCO_3$ (2 x 10 mL), water (10 mL) and brine (10 mL), dried, filtered and concentrated. Flash column chromatography on silica gel using petroleum ether/EtOAc (5:1) as the eluent afforded the product as a yellow oil (29 mg, 0.11 mmol, 82%). TLC [petroleum ether/EtOAc (5:1)]: $R_f$ = 0.30. $^1$H NMR

(400 MHz, $CDCl_3$): $\delta$ = 7.00 (s, 1H, 4-*H*), 6.61 (s, 1H, 10-*H*), 6.25 (s, 1H, 9-*H*), 3.55 (t, 2H, $^3J$ = 8.2 Hz, 2-*H*), 3.07-2.96 (m, 2H, 3-*H*), 3.02 (broad s, 1H, N*H*), 2.12-2.07 (m, 1H, 5-$H_{(equiv)}$), 1.92 (qt, 1H, $^3J$ = 13.7 Hz, $^3J$ = 3.4 Hz, 6-$H_{(ax.)}$), 1.65-1.60 (m, 1H, 7-$H_{(equiv)}$), 1.61-1.56 (m, 1H, 6-$H_{(equiv)}$), 1.34 (s, 3H, $CH_2CCH_3$), 1.27 (s, 3H, $C(CH_3)_{2,equiv}$), 1.22 (s, 3H, $C(CH_3)_{2,ax.}$), 1.09 (dt, 1H, $^3J$ = 13.3 Hz, $^3J$ = 4.0 Hz, 7-$H_{(ax.)}$), 0.98 (dt, 1H, $^3J$ = 13.1 Hz, $^3J$ = 3.7 Hz, 5-$H_{(ax.)}$). $^{13}$C NMR (100 MHz, $CDCl_3$): $\delta$ = 163.7 (1C, *C*-8a), 149.9 (1C, *C*-10a), 146.8 (1C, *C*-4a), 141.3 (1C, *C*-9a), 125.2 (1C, *C*-3a), 120.4 (1C, *C*-9), 117.2 (1C, *C*-4), 102.5 (1C, *C*-10), 50.0 (1C, *C*-4b), 47.7 (1C, *C*-2), 42.5 (1C, *C*-7), 38.3 (1C, *C*-5), 35.3 (1C, *C*-8), 31.0 (1C, $C(CH_3)_{2,equiv}$), 29.7 (1C, *C*-3), 25.1 (1C, $C(CH_3)_{2,ax.}$), 23.5 (1C, $CH_2CCH_3$), 19.8 (1C, *C*-6). IR (diamond ATR): $\tilde{\nu}$ = 3367 (w), 3056 (w), 2956 (m), 2923 (s), 2862 (m), 2843 (m), 1612 (m), 1466 (s), 1366 (w), 1346 (w), 1306 (m), 1281 (s), 1233 (s), 1185 (w), 1145 (w), 1124 (m), 1055 (m), 1009 (w), 967 (w), 939 (w), 866 (s), 832 (w), 782 (w), 754 (m), 670 (m), 536 (s). UV/Vis (MeOH): $\lambda_{max}$ (log $\varepsilon$) = 317 (3.68), 308 (3.69), 236 (4.44). HRESIMS: calculated ($[C_{18}H_{24}N^+]$) 254.19033, found 254.19059 (1.02 ppm).

**3-Methyl-1-(1-(triisopropylsilyl)-indolin-6-yl)-pent-1-yn-3-ol (43):** *N*-Triisopropylsilyl-6-iodoindoline (**39**, 410 mg, 1.02 mmol, 1.00 equiv), $Pd(PPh_3)_2Cl_2$ (22 mg, 0.03 mmol, 0.03 equiv) and 3-methyl-pent-1-yn-3-ol (120 µL, 1.07 mmol, 1.05 equiv) were dissolved in freshly distilled and carefully degassed $NEt_3$ (10 mL) under $N_2$. The yellow suspension was degassed again for 30 min while separately CuI (6 mg, 0.03 mmol, 0.03 equiv) was dissolved in degassed $NEt_3$ (5 mL) under $N_2$. The CuI solution was then added to the reaction mixture and heated to 40 °C. At this temperature a second portion of 3-methyl-pent-1-yn-3-ol (54 µL, 0.46 mmol, 0.45 equiv) was added. The reaction mixture turned brown and was stirred for 2 h at 40 °C at which time the solution turned yellow again. The reaction mixture was filtered through Celite 545, rinsed with EtOAc (50 mL) and concentrated. Flash column chromatography on silica gel using petroleum ether/EtOAc (10:1), primed with petroleum ether/EtOAc/$NEt_3$ (10:1:0.1) as the eluent afforded the product as a yellow oil (365 mg, 0.98 mmol, 96%). TLC [petroleum ether/EtOAc (10:1)]: $R_f$ = 0.34. $^1$H NMR (400 MHz, $CDCl_3$): $\delta$ = 6.96 (d, 1H, $^3J$ = 7.4 Hz, 4-*H*), 6.68 (dd, 1H, $^3J$ = 7.5 Hz, $^4J$ = 1.3 Hz, 5-*H*), 6.62 (d, 1H, $^4J$ = 1.1 Hz, 7-*H*), 3.70 (t, 2H, $^3J$ = 8.6 Hz, 2-*H*), 2.96 (t, 2H, $^3J$ = 8.6 Hz, 3-*H*), 2.02 (s, 1H, O*H*), 1.78 (dq, 2H, $^3J$ = 7.5 Hz, $^4J$ = 2.0 Hz, $CH_3$-$CH_2$), 1.56 (s, 3H, C(OH)-$CH_3$), 1.43 (sept, 3H, $^3J$ = 7.5 Hz, $CH(CH_3)_2$), 1.13 (d, 18H, $^3J$ = 7.5 Hz, $CH(CH_3)_2$), 1.10 (t, 3H, $^3J$ = 7.5 Hz, $CH_3$-$CH_2$). $^{13}$C NMR (100 MHz, $CDCl_3$): $\delta$ = 152.8 (1C, *C*-7a), 132.8 (1C, *C*-3a), 123.8 (1C, *C*-4), 121.2 (1C, *C*-5), 121.0 (1C, *C*-6), 112.6 (1C, *C*-7), 90.5 (1C, C≡*C*COH), 84.8 (1C, *C*≡CCOH), 69.2 (1C, *C*-OH), 50.7 (1C, *C*-2), 36.8 (1C, $CH_3$-$CH_2$), 30.4 (1C, *C*-3), 29.3 (1C, C(OH)-$CH_3$), 18.5 (6C, $CH(CH_3)_2$), 12.9 (3C, $CH(CH_3)_2$), 9.1 (1C, $CH_3$-$CH_2$). IR

(diamond ATR): $\tilde{\nu}$ = 3354 (w), 2942 (m), 2866 (m), 2224 (w), 1600 (m), 1573 (w), 1489 (m), 1468 (m), 1417 (m), 1355 (w), 1326 (w), 1258 (m), 1214 (m), 1146 (w), 1109 (m), 1036 (m), 1014 (m), 985 (s), 964 (w), 911 (m), 881 (s), 850 (m), 805 (m), 683 (s), 641 (s), 603 (m), 579 (w). UV/Vis (MeOH): $\lambda_{max}$ (log $\varepsilon$) = 331 (3.61), 272 (4.01), 241 (4.53), 203 (4.16). HRESIMS: calculated ([$C_{23}H_{37}NOSiNa^+$]) 394.25366, found 394.25380 (0.36 ppm).

**(*Z*)-3-Methyl-1-(1-(triisopropylsilyl)-indolin-6-yl)-pent-1-en-3-ol (44):** 3-Methyl-1-(1-(triisopropylsilyl)-indolin-6-yl)-pent-1-yn-3-ol (**43**, 60 mg, 0.16 mmol, 1.00 equiv), Lindlar catalyst (5% Pd, 4 mg, 0.002 mmol, 0.01 equiv) and $K_2CO_3$ (10 mg, 0.07 mmol, 0.45 equiv) were dissolved under $N_2$ in EtOAc (3 mL). After exchange of the atmosphere against $H_2$ the mixture was stirred for 18 h at room temperature. The reaction mixture was filtered through Celite 545, rinsed with EtOAc (30 mL) and concentrated. Flash column chromatography on silica gel using petroleum ether/EtOAc (20:1), primed with petroleum ether/EtOAc/$NEt_3$ (20:1:0.2) as the eluent afforded the product as a colorless liquid (28 mg, 0.08 mmol, 47%). TLC [petroleum ether/EtOAc (20:1)]: $R_f$ = 0.32. $^1$H NMR (600 MHz, acetone-$d_6$): $\delta$ = 6.97 (d, 1H, $^3J$ = 7.4 Hz, 4-*H*), 6.90 (s, 1H, 7-*H*), 6.54 (d, 1H, $^3J$ = 7.4 Hz, 5-*H*), 6.33 (d, 1H, $^3J$ = 13.2 Hz, C*H*=CHCOH), 5.59 (d, 1H, $^3J$ = 13.2 Hz, CH=C*H*COH), 3.75 (t, 2H, $^3J$ = 8.6 Hz, 2-*H*), 3.17 (s, 1H, O*H*), 2.95 (t, 2H, $^3J$ = 8.5 Hz, 3-*H*), 1.67-1.40 (m, 2H, $CH_3$-C$H_2$), 1.67-1.40 (m, 3H, C*H*($CH_3$)$_2$), 1.25 (s, 3H, C(OH)-C$H_3$), 1.15 (d, 18H, $^3J$ = 7.4 Hz, CH(C$H_3$)$_2$), 0.89 (t, 3H, $^3J$ = 7.4 Hz, C$H_3$-$CH_2$). $^{13}$C NMR (150 MHz, acetone-$d_6$): $\delta$ = 153.3 (1C, *C*-7a), 139.0 (1C, CH=*C*HCOH), 137.5 (1C, *C*-6), 131.5 (1C, *C*-3a), 130.2 (1C, *C*H=CHCOH), 124.3 (1C, *C*-4), 119.6 (1C, *C*-5), 111.8 (1C, *C*-7), 73.7 (1C, *C*-OH), 51.6 (1C, *C*-2), 36.6 (1C, $CH_3$-*C*$H_2$), 30.8 (1C, *C*-3), 28.7 (1C, C(OH)-*C*$H_3$), 18.9 (6C, CH(*C*$H_3$)$_2$), 13.6 (3C, *C*H($CH_3$)$_2$), 8.7 (1C, *C*$H_3$-$CH_2$). IR (diamond ATR): $\tilde{\nu}$ = 3572 (w), 3455 (w), 2944 (m), 2866 (m), 1602 (m), 1575 (w), 1486 (m), 1462 (m), 1418 (m), 1367 (w), 1324 (w), 1251 (s), 1109 (m), 1037 (m), 1016 (m), 984 (s), 914 (w), 882 (s), 797 (w), 733 (w), 681 (s), 641 (s), 587 (m). UV/Vis (MeOH): $\lambda_{max}$ (log $\varepsilon$) = 318 (3.52), 259 (4.06), 217 (4.28). HRESIMS: calculated ([$C_{26}H_{43}NOSiNa^+$]) 396.26931, found 396.26963 (0.81 ppm).

**5-Ethyl-5-methyl-1,2,3,5-tetrahydrocyclopenta[*f*]indole (45):** (*Z*)-3-Methyl-1-(1-(triisopropylsilyl)-indolin-6-yl)-pent-1-en-3-ol (**44**, 32 mg, 0.09 mmol, 1.00 equiv) was dissolved in dry DCM (8 mL) under $N_2$ and cooled to 0 °C. A 1.0 M solution of $SnCl_4$ in DCM (130 µL, 0.13 mmol, 1.50 equiv) was added slowly and the reaction mixture was stirred for 5 min at 0 °C, followed by 4 h at room temperature. A saturated solution of $NaHCO_3$ (0.5 mL) was added to quench the reaction. After dilution with water (5 mL) and DCM (5 mL) the organic phase was washed with saturated $NaHCO_3$ (2 x 10 mL), water (10 mL) and brine

(10 mL), dried, filtered and concentrated. Flash column chromatography on silica gel using petroleum ether/EtOAc (5:1) as the eluent afforded the product as yellow oil (1.8 mg, 0.01 mmol, 11%). TLC [petroleum ether/EtOAc (5:1)]: $R_f$ = 0.33. $^1$H NMR (600 MHz, $CDCl_3$): $\delta$ = 7.02 (s, 1H, 4-*H*), 6.68 (s, 1H, 8-*H*), 6.55 (d, 1H, 7-*H*), 6.25 (d, 1H, 6-*H*), 3.60 (t, 2H, $^3J$ = 8.3 Hz, 2-*H*), 3.05 (t, 2H, $^3J$ = 8.3 Hz, 3-*H*), 1.79-1.63 (m, 2H, $CH_3$-C*H*$_2$), 1.26 (s, 3H, $CH_2$CC*H*$_3$), 0.65 (t, 3H, $^3J$ = 7.4 Hz, C*H*$_3$-$CH_2$). $^{13}$C NMR (150 MHz, $CDCl_3$): $\delta$ = 149.2 (1C, *C*-8a), 145.5 (1C, *C*-6), 145.2 (1C, *C*-4a), 143.1 (1C, *C*-7a), 128.8 (1C, *C*-7), 126.7 (1C, *C*-3a), 117.8 (1C, *C*-4), 103.8 (1C, *C*-8), 52.9 (1C, *C*-5), 47.7 (1C, *C*-2), 31.3 (1C, $CH_3$-*C*H$_2$), 29.9 (1C, *C*-3), 23.3 (1C, $CH_2$C*C*H$_3$), 9.4 (1C, *C*H$_3$-$CH_2$). IR (diamond ATR): $\tilde{\nu}$ = 3406 (w), 3260 (w), 3054 (w), 2961 (m), 2920 (w), 2851 (w), 1610 (m), 1459 (m), 1366 (m), 1260 (w), 1217 (w), 1097 (w), 1047 (w), 1017 (w), 913 (w), 872 (w), 799 (m), 756 (s), 730 (s), 694 (m), 666 (w), 593 (w). UV/Vis (MeOH): $\lambda_{max}$ (log $\varepsilon$) = 294 (3.84), 236 (4.12). HRESIMS: calculated ([$C_{14}H_{17}N^+$]) 200.14338, found 200.14346 (0.40 ppm).

**2-Methyl-4-(1-(triisopropylsilyl)indolin-6-yl)but-3-yn-2-ol:** *N*-Triisopropylsilyl-6-iodoindoline (**39**, 2.02 g, 5.03 mmol, 1.00 equiv), $Pd(PPh_3)_2Cl_2$ (106 mg, 0.15 mmol, 0.03 equiv) and 2-methyl-but-3-yn-2-ol (520 µL, 5.28 mmol, 1.05 equiv) were dissolved in freshly distilled and carefully degassed $NEt_3$ (46 mL) under Argon. The yellow suspension was degassed again for 30 min while separately CuI (29 mg, 0.15 mmol, 0.03 equiv) was dissolved in degassed $NEt_3$ (21 mL) under Argon. The CuI solution was then added to the reaction mixture and heated to 40 °C. At this temperature a second portion of 2-methyl-but-3-yn-2-ol (220 µL, 2.26 mmol, 0.45 equiv) was added. The reaction mixture turned brown and was stirred for 2 h at 40 °C. The reaction mixture was filtered through Celite 545, rinsed with EtOAc (100 mL) and concentrated. Flash column chromatography on silica gel using petroleum ether/EtOAc (10:1) as the eluent afforded the product as yellow oil (1.81 g, 5.03 mmol, quant.). TLC [petroleum ether/EtOAc (10:1)]: $R_f$ = 0.20. $^1$H NMR (400 MHz, $CDCl_3$): $\delta$ = 6.95 (d, 1H, $^3J$ = 7.5 Hz, 4-*H*), 6.68 (dd, 1H, $^3J$ = 7.4 Hz, $^4J$ = 1.3 Hz, 5-*H*), 6.61 (d, 1H, $^4J$ = 1.3 Hz, 7-*H*), 3.70 (t, 2H, $^3J$ = 8.6 Hz, 2-*H*), 2.95 (t, 2H, $^3J$ = 8.6 Hz, 3-*H*), 2.25 (s, 1H, O*H*), 1.60 (s, 6H, C(C*H*$_3$)$_2$), 1.43 (sept, 3H, $^3J$ = 7.6 Hz, C*H*($CH_3$)$_2$), 1.13 (d, 18H, $^3J$ = 7.7 Hz, CH(C*H*$_3$)$_2$). $^{13}$C NMR (100 MHz, $CDCl_3$): $\delta$ = 152.7 (1C, *C*-7a), 132.7 (1C, *C*-3a), 123.8 (1C, *C*-4), 121.2 (1C, *C*-5), 120.9 (1C, *C*-6), 112.5 (1C, *C*-7), 91.6 (1C, C≡*C*COH), 83.4 (1C, *C*≡CCOH), 65.6 (1C, *C*-OH), 50.7 (1C, *C*-2), 31.5 (2C, C(*C*H)$_3$)$_2$), 30.3 (1C, *C*-3), 18.4 (6C, CH(*C*H$_3$)$_2$), 12.8 (3C, *C*H($CH_3$)$_2$). IR (diamond ATR): $\tilde{\nu}$ = 3336 (w), 2944 (s), 2891 (m), 2865 (s), 2228 (w), 1600 (s), 1573 (w), 1489 (s), 1468 (s), 1417 (s), 1357 (m), 1326 (m), 1291 (w), 1225 (s), 1163 (m), 1145 (m), 1110 (m), 1040 (m), 1015 (m), 991 (s), 959 (s), 923 (m), 881 (s), 849 (m), 805 (s), 728 (w), 683 (s), 642 (s), 603 (m), 577 (m). UV/Vis (MeOH):

$\lambda_{max}$ (log $\varepsilon$) = 330 (3.59), 272 (3.99), 240 (4.52), 202 (4.20). HRESIMS: calculated ([$C_{22}H_{35}NOSiNa^+$]) 380.23801, found 380.23813 (0.31 ppm).

**2-Methyl-4-(1-(triisopropylsilyl)indolin-6-yl)but-3-yn-2-yl acetate (46):** 2-Methyl-4-(1-(triisopropylsilyl)indolin-6-yl)but-3-yn-2-ol (650 mg, 1.82 mmol, 1.00 equiv), $Ac_2O$ (430 µL, 4.54 mmol, 2.50 equiv) and DMAP (22 mg, 0.18 mmol, 0.10 equiv) were dissolved in pyridine (18 mL) under argon. The clear solution slowly turned orange and was stirred for 72 h at room temperature. The reaction mixture was quenched with aq. $NaHCO_3$ (10 mL) and extracted with EtOAc (3 x 15 mL). The organic phase was washed with $H_2O$ (15 mL) and brine (15 mL), dried with $MgSO_4$, filtered and concentrated. Flash column chromatography on silica gel using petroleum ether/EtOAc (50:1) as the eluent afforded the product as a colorless solid (633 mg, 1.58 mmol, 87%). TLC [petroleum ether/EtOAc (50:1)]: $R_f$ = 0.21. $^1$H NMR (400 MHz, $CDCl_3$): $\delta$ = 6.95 (d, 1H, $^3J$ = 7.4 Hz, 4-*H*), 6.71 (dd, 1H, $^3J$ = 7.5 Hz, $^4J$ = 1.3 Hz, 5-*H*), 6.60 (d, 1H, $^4J$ = 1.2 Hz, 7-*H*), 3.69 (t, 2H, $^3J$ = 8.6 Hz, 2-*H*), 2.95 (t, 2H, $^3J$ = 8.6 Hz, 3-*H*), 2.03 (s, 3H, (C=O)C*H*$_3$), 1.75 (s, 6H, C(C*H*$_3$)$_2$), 1.43 (sept, 3H, $^3J$ = 7.5 Hz, C*H*(CH$_3$)$_2$), 1.13 (d, 18H, $^3J$ = 7.6 Hz, CH(C*H*$_3$)$_2$). $^{13}$C NMR (100 MHz, $CDCl_3$): $\delta$ = 169.3 (1C, (*C*=O)CH$_3$), 152.6 (1C, *C*-7a), 132.8 (1C, *C*-3a), 123.8 (1C, *C*-4), 121.6 (1C, *C*-5), 120.9 (1C, *C*-6), 112.6 (1C, *C*-7), 88.3 (1C, Aryl-C≡*C*), 85.3 (1C, Aryl-*C*≡C), 72.9 (1C, *C*-OAc), 50.7 (1C, *C*-2), 30.4 (1C, *C*-3), 29.1 (2C, C(*C*H$_3$)$_2$), 22.1 (1C, (C=O)*C*H$_3$), 18.5 (6C, CH(*C*H$_3$)$_2$), 12.9 (3C, *C*H(CH$_3$)$_2$). IR (diamond ATR): $\tilde{\nu}$ = 2944 (m), 2891 (w), 2866 (m), 2227 (w), 1744 (s), 1600 (m), 1489 (m), 1469 (m), 1418 (m), 1364 (m), 1327 (w), 1299 (w), 1232 (s), 1194 (w), 1147 (m), 1127 (m), 1109 (m), 1040 (m), 1014 (m), 993 (m), 961 (m), 938 (w), 881 (m), 848 (m), 803 (m), 683 (m), 641 (m), 607 (m), 581 (m). UV/Vis (MeOH): $\lambda_{max}$ (log $\varepsilon$) = 336 (3.58), 277 (3.99), 243 (4.50). HRESIMS: calculated ([$C_{24}H_{37}NO_2SiNa^+$]) 422.24858, found 422.24863 (0.12 ppm).

**3-Methyl-1-(1-(triisopropylsilyl)indolin-6-yl)pent-1-yn-3-yl acetate (47):** 3-Methyl-1-(1-(triisopropylsilyl)indolin-6-yl)pent-1-yn-3-ol (**43**, 200 mg, 0.54 mmol, 1.00 equiv), $Ac_2O$ (130 µL, 1.35 mmol, 2.50 equiv) and DMAP (7 mg, 0.05 mmol, 0.10 equiv) were dissolved in pyridine (3 mL) under argon. The clear solution slowly turned orange and was stirred for 72 h at room temperature. The reaction was quenched with aq. $NaHCO_3$ (3 mL) and extracted with EtOAc (3 x 5 mL). The organic phase was washed with $H_2O$ (5 mL) and brine (5 mL), dried with $MgSO_4$ and concentrated. Flash column chromatography on silica gel using petroleum ether/EtOAc (30:1) as the eluent afforded the product as colorless oil (187 mg, 0.45 mmol, 84%). TLC [petroleum ether/EtOAc (30:1)]: $R_f$ = 0.33. $^1$H NMR (400 MHz, $CDCl_3$): $\delta$ = 6.95 (d, 1H, $^3J$ = 7.3 Hz, 4-*H*), 6.71 (dd, 1H, $^3J$ = 7.4 Hz, $^4J$ = 1.3 Hz, 5-*H*), 6.62 (d, 1H,

$^4J$ = 1.1 Hz, 7-*H*), 3.69 (t, 2H, $^3J$ = 8.6 Hz, 2-*H*), 2.95 (t, 2H, $^3J$ = 8.6 Hz, 3-*H*), 2.07 (dq, 1H, $^3J$ = 7.4 Hz, $^2J$ = 13.5 Hz, $CH_3$-C$H_2$), 2.04 (s, 3H, (C=O)C$H_3$), 1.92 (dq, 1H, $^3J$ = 7.4 Hz, $^2J$ = 13.5 Hz, $CH_3$-C$H_2$), 1.74 (s, 3H, C(OAc)-C$H_3$), 1.42 (sept, 3H, $^3J$ = 7.4 Hz, C*H*($CH_3$)$_2$), 1.13 (d, 18H, $^3J$ = 7.4 Hz, CH(C$H_3$)$_2$), 1.07 (t, 3H, $^3J$ = 7.4 Hz, C$H_3$-$CH_2$). $^{13}$C NMR (100 MHz, $CDCl_3$): $\delta$ = 169.6 (1C, (*C*=O)$CH_3$), 152.7 (1C, *C*-7a), 132.8 (1C, *C*-3a), 123.8 (1C, *C*-4), 121.6 (1C, *C*-5), 121.0 (1C, *C*-6), 112.8 (1C, *C*-7), 87.4 (1C, Aryl-C≡*C*), 86.4 (1C, Aryl-*C*≡C), 76.7 (1C, *C*-OAc), 50.8 (1C, *C*-2), 34.6 (1C, $CH_3$-*C*$H_2$), 30.4 (1C, *C*-3), 26.2 (1C, C(OAc)-*C*$H_3$), 22.1 (1C, (C=O)*C*$H_3$), 18.5 (6C, CH(*C*$H_3$)$_2$), 12.9 (3C, *C*H($CH_3$)$_2$), 8.7 (1C, *C*$H_3$-$CH_2$). IR (diamond ATR): $\tilde{\nu}$ = 2943 (m), 2866 (m), 2227 (w), 1744 (s), 1600 (m), 1489 (m), 1467 (m), 1418 (m), 1366 (m), 1327 (w), 1236 (s), 1160 (w), 1143 (w), 1108 (m), 1034 (m), 1014 (m), 985 (m), 940 (w), 882 (m), 850 (m), 806 (m), 683 (m), 641 (m), 606 (w), 578 (w). UV/Vis (MeOH): $\lambda_{max}$ (log $\varepsilon$) = 334 (3.60), 273 (3.99), 242 (4.51), 203 (4.16). HRESIMS: calculated ([$C_{25}H_{39}NO_2SiNa^+$]) 436.26423, found 436.26433 (0.23 ppm).

**5,5-Dimethyl-1-(triisopropylsilyl)-2,3,5,6-tetrahydrocyclopenta[*f*]indol-7-one (48) from 46:** 2-Methyl-4-(1-(triisopropylsilyl)indolin-6-yl)but-3-yn-2-yl acetate (**46**, 81 mg, 0.203 mmol, 1.00 equiv) and $PtI_2$ (9.0 mg, 0.020 mmol, 0.10 equiv) were dissolved in dry toluene (4 mL) under argon. After exchange of the atmosphere against CO the reaction mixture was stirred for 2 h at 80 °C and the solution turned orange. The mixture was allowed to cool to room temperature. Flash column chromatography on silica gel using petroleum ether/EtOAc (50:1) as the eluent afforded the product as yellow oil with blue fluorescence under UV light (24 mg, 0.067 mmol, 33%). TLC [petroleum ether/EtOAc (50:1)]: $R_f$ = 0.11. $^1$H NMR (400 MHz, $CDCl_3$): $\delta$ = 7.13 (s, 1H, 4-*H*), 6.76 (s, 1H, 8-*H*), 3.76 (t, 2H, $^3J$ = 8.5 Hz, 2-*H*), 3.04 (t, 2H, $^3J$ = 8.6 Hz, 3-*H*), 2.54 (s, 2H, (C=O)C$H_2$), 1.48 (sept, 3H, $^3J$ = 7.6 Hz, C*H*($CH_3$)$_2$), 1.36 (s, 6H, C(C$H_3$)$_2$), 1.13 (d, 18H, $^3J$ = 7.5 Hz, CH(C$H_3$)$_2$). $^{13}$C NMR (100 MHz, $CDCl_3$): $\delta$ = 205.7 (1C, *C*=O), 155.3 (1C, *C*-4a), 152.8 (1C, *C*-8a), 142.3 (1C, *C*-3a), 135.1 (1C, *C*-7a), 118.7 (1C, *C*-4), 101.6 (1C, *C*-8), 53.7 (1C, *C*-6), 50.9 (1C, *C*-2), 37.5 (1C, *C*-5), 30.7 (1C, *C*-3), 30.2 (2C, C(*C*$H_3$)$_2$), 18.5 (6C, CH(*C*$H_3$)$_2$), 12.8 (3C, *C*H($CH_3$)$_2$). IR (diamond ATR): $\tilde{\nu}$ = 2948 (m), 2890 (m), 2865 (m), 1700 (s), 1611 (m), 1468 (s), 1440 (s), 1364 (m), 1336 (w), 1295 (s), 1264 (s), 1172 (w), 1134 (m), 1056 (m), 1040 (m), 1015 (m), 998 (s), 954 (m), 922 (m), 881 (s), 850 (m), 807 (w), 752 (m), 729 (w), 684 (m), 641 (m), 612 (w), 570 (m). UV/Vis (MeOH): $\lambda_{max}$ (log $\varepsilon$) = 380 (3.55), 250 (4.46), 201 (4.20). HRESIMS: calculated ([$C_{22}H_{35}NOSiNa^+$]) 380.23801, found 380.23817 (0.42 ppm).

**5-Ethyl-5-methyl-1-(triisopropylsilyl)-2,3,5,6-tetrahydrocyclopenta[*f*]indol-7(1*H*)-one (49):** 3-Methyl-1-(1-(triisopropylsilyl)indolin-6-yl)pent-1-yn-3-yl acetate (**47**, 50 mg,

0.128 mmol, 1.00 equiv) and $PtI_2$ (6.0 mg, 0.013 mmol, 0.10 equiv) were dissolved in dry toluene (2 mL) under argon. After exchange of the atmosphere against CO the reaction was stirred for 2 h at 80 °C and the solution turned orange. The reaction mixture was allowed to cool to room temperature and concentrated. Flash column chromatography on silica gel using petroleum ether/EtOAc (50:1) as the eluent afforded the product as yellow oil (14 mg, 0.038 mmol, 29%). TLC [petroleum ether/EtOAc (50:1)]: $R_f = 0.19$. $^1$H NMR (400 MHz, $CDCl_3$): $\delta = 7.07$ (s, 1H, 4-*H*), 6.76 (s, 1H, 8-*H*), 3.76 (t, 2H, $^3J = 8.4$ Hz, 2-*H*), 3.03 (t, 2H, $^3J = 8.4$ Hz, 3-*H*), 2.59 (d, 1H, $^2J = 18.7$ Hz, (C=O)C*H*$_2$), 2.37 (d, 1H, $^2J = 18.7$ Hz, (C=O)C*H*$_2$), 1.68 (dq, 1H, $^3J = 7.4$ Hz, $^2J = 18.7$ Hz, $CH_3$-C*H*$_2$), 1.63 (dq, 1H, $^3J = 7.4$ Hz, $^2J = 18.7$ Hz, $CH_3$-C*H*$_2$), 1.48 (sept, 3H, $^3J = 7.8$ Hz, C*H*($CH_3$)$_2$), 1.33 (s, 3H, C*H*$_3$), 1.12 (d, 18H, $^3J = 8.0$ Hz, CH(C*H*$_3$)$_2$), 0.75 (t, 3H, $^3J = 7.4$ Hz, C*H*$_3$-$CH_2$). $^{13}$C NMR (100 MHz, $CDCl_3$): $\delta = 206.0$ (1C, *C*=O), 154.2 (1C, *C*-4a), 152.8 (1C, *C*-8a), 142.3 (1C, *C*-3a), 136.0 (1C, *C*-7a), 118.9 (1C, *C*-4), 101.6 (1C, *C*-8), 50.9 (1C, *C*-2), 50.5 (1C, *C*-6), 41.2 (1C, *C*-5), 34.9 (1C, $CH_3$-*C*H$_2$), 30.7 (1C, *C*-3), 27.9 (1C, *C*H$_3$), 18.5 (6C, CH(*C*H$_3$)$_2$), 12.8 (3C, *C*H($CH_3$)$_2$), 9.4 (1C, *C*H$_3$-$CH_2$). IR (diamond ATR): $\tilde{\nu} = 2959$ (m), 2943 (m), 2892 (m), 2865 (s), 1688 (s), 1610 (w), 1464 (m), 1442 (m), 1367 (w), 1339 (w), 1308 (w), 1259 (s), 1015 (s), 920 (w), 881 (s), 845 (m), 794 (s), 674 (s), 659 (m), 644 (m), 567 (m). UV/Vis (MeOH): $\lambda_{max}$ (log $\varepsilon$) = 362 (3.33), 310 (3.35), 249 (4.29), 203 (4.00). HRESIMS: calculated ([$C_{23}H_{37}NOSiNa^+$]) 394.25366, found 394.25369 (0.08 ppm).

**5,5-Dimethyl-1-(triisopropylsilyl)-1,2,3,5-tetrahydrocyclopenta[*f*]indol-7-yl acetate (50):** $PPh_3AuCl$ (3.1 mg, 0.006 mmol, 0.05 equiv) was dissolved in dry DCM (8 mL) under argon and $AgClO_4$ (1.0 mg, 0.005 mmol, 0.04 equiv) was added in the absence of light. The silver salt was dissolved by sonication while the solution turned pale. After filtration into a Schlenk flask under argon through a pad of Celite 545, which was rinsed with DCM (1 mL), 2-methyl-4-(1-(triisopropylsilyl)indolin-6-yl)but-3-yn-2-yl acetate (**46**, 50 mg, 0.125 mmol, 1.00 equiv) was dissolved in dry DCM (1 mL) and added. The solution slowly turned yellow and was stirred for 72 h at room temperature. After concentration of the organic phase flash column chromatography on silica gel using petroleum ether/EtOAc (100:1) as the eluent afforded the product as colorless solid (40 mg, 0.101 mmol, 81%). TLC [petroleum ether/EtOAc (100:1)]: $R_f = 0.25$. $^1$H NMR (400 MHz, $CDCl_3$): $\delta = 7.01$ (s, 1H, 4-*H*), 6.48 (s, 1H, 8-*H*), 6.09 (s, 1H, 6-*H*), 3.73 (t, 2H, $^3J = 8.6$ Hz, 2-*H*), 2.98 (t, 2H, $^3J = 8.6$ Hz, 3-*H*), 2.28 (s, 3H, (C=O)C*H*$_3$), 1.43 (sept, 3H, $^3J = 7.5$ Hz, C*H*($CH_3$)$_2$), 1.32 (s, 6H, C(C*H*$_3$)$_2$), 1.14 (d, 18H, $^3J = 7.5$ Hz, CH(C*H*$_3$)$_2$). $^{13}$C NMR (100 MHz, $CDCl_3$): $\delta = 168.1$ (1C, (*C*=O)$CH_3$), 151.6 (1C, *C*-8a), 146.5 (1C, *C*-7), 141.0 (1C, *C*-4a), 136.1 (1C, *C*-7a), 130.2 (1C, *C*-3a), 127.0 (1C, *C*-6), 117.2 (1C, *C*-4), 100.4 (1C, *C*-8), 51.1 (1C, *C*-2), 45.3 (1C, *C*-5), 30.6 (1C, *C*-3), 25.3 (2C, C(*C*H$_3$)$_2$),

21.2 (1C, (C=O)$CH_3$), 18.6 (6C, CH($CH_3$)$_2$), 12.9 (3C, $C$H($CH_3$)$_2$). IR (diamond ATR): $\tilde{\nu}$ = 2947 (m), 2866 (m), 2843 (m), 1768 (m), 1615 (m), 1591 (m), 1564 (m), 1463 (m), 1361 (m), 1334 (m), 1280 (m), 1255 (m), 1208 (m), 1190 (s), 1154 (m), 1128 (m), 1097 (m), 1056 (w), 1035 (m), 1016 (m), 994 (m), 945 (m), 926 (w), 884 (m), 845 (m), 813 (m), 735 (m), 682 (m), 643 (s), 576 (s), 558 (m), 539 (w). UV/Vis (MeOH): $\lambda_{max}$ (log $\varepsilon$) = 340 (3.52), 244 (4.41), 201 (4.04). HRESIMS: calculated ([$C_{24}H_{37}NO_2SiNa^+$]) 422.24858, found 422.24876 (0.43 ppm).

**5-Ethyl-5-methyl-1-(triisopropylsilyl)-1,2,3,5-tetrahydrocyclopenta[*f*]indol-7-yl acetate (51):** $PPh_3AuCl$ (2.5 mg, 0.005 mmol, 0.04 equiv) was dissolved in dry DCM (8 mL) under argon and $AgBF_4$ (0.7 mg, 0.004 mmol, 0.03 equiv) was added in the absence of light. 3-Methyl-1-(1-(triisopropylsilyl)indolin-6-yl)pent-1-yn-3-yl acetate (**47**, 50 mg, 0.121 mmol, 1.00 equiv) was dissolved in dry DCM (1 mL) and slowly added. The solution turned yellow and was stirred for 4 h at room temperature. The reaction mixture was filtered through a pad of Celite 545 and rinsed with DCM (50 mL). After concentration of the organic phase flash column chromatography on silica gel using petroleum ether/EtOAc (60:1) as the eluent afforded the product as colorless solid (26 mg, 0.064 mmol, 53%). TLC [petroleum ether/EtOAc (60:1)]: $R_f$ = 0.35. $^1$H NMR (400 MHz, $CDCl_3$): $\delta$ = 6.95 (s, 1H, 4-*H*), 6.47 (s, 1H, 8-*H*), 6.03 (s, 1H, 6-*H*), 3.73 (t, 2H, $^3J$ = 8.6 Hz, 2-*H*), 2.98 (t, 2H, $^3J$ = 8.5 Hz, 3-*H*), 2.28 (s, 3H, (C=O)$CH_3$), 1.76 (dq, 1H, $^3J$ = 7.4 Hz, $^2J$ = 13.4 Hz, $CH_3$-$CH_2$), 1.66 (dq, 1H, $^3J$ = 7.4 Hz, $^2J$ = 13.4 Hz, $CH_3$-$CH_2$), 1.29 (s, 3H, $CH_3$), 1.42 (sept, 3H, $^3J$ = 7.8 Hz, $CH$($CH_3$)$_2$), 1.14 (d, 18H, $^3J$ = 7.4 Hz, CH($CH_3$)$_2$), 0.72 (t, 3H, $^3J$ = 7.5 Hz, $CH_3$-$CH_2$). $^{13}$C NMR (100 MHz, $CDCl_3$): $\delta$ = 168.1 (1C, ($C$=O)$CH_3$), 151.6 (1C, *C*-8a), 147.1 (1C, *C*-7), 140.0 (1C, *C*-4a), 136.9 (1C, *C*-7a), 130.1 (1C, *C*-3a), 125.1 (1C, *C*-6), 117.5 (1C, *C*-4), 100.3 (1C, *C*-8), 51.1 (1C, *C*-2), 49.4 (1C, *C*-5), 31.8 (1C, $CH_3$-$CH_2$), 30.6 (1C, *C*-3), 23.4 (1C, $CH_3$), 21.2 (1C, (C=O)$CH_3$), 18.6 (6C, CH($CH_3$)$_2$), 12.9 (3C, $C$H($CH_3$)$_2$), 9.7 (1C, $CH_3$-$CH_2$). IR (diamond ATR): $\tilde{\nu}$ = 2946 (m), 2892 (w), 2866 (m), 1773 (m), 1602 (w), 1567 (w), 1459 (m), 1365 (m), 1335 (m), 1264 (m), 1202 (s), 1154 (m), 1132 (m), 1097 (m), 1044 (m), 1013 (m), 993 (m), 924 (w), 881 (s), 842 (m), 814 (m), 735 (w), 682 (m), 642 (m), 576 (m). UV/Vis (MeOH): $\lambda_{max}$ (log $\varepsilon$) = 344 (3.50), 285 (3.72), 246 (4.40), 224 (4.24). HRESIMS: calculated ([$C_{25}H_{39}NO_2SiNa^+$]) 436.26423, found 436.26436 (0.30 ppm).

**5,5-Dimethyl-1-(triisopropylsilyl)-2,3,5,6-tetrahydrocyclopenta[*f*]indol-7-one (48) from 50:** 5,5-Dimethyl-1-(triisopropylsilyl)-1,2,3,5-tetrahydrocyclopenta[*f*]indol-7-yl acetate (**50**, 30 mg, 0.075 mmol, 1.00 equiv) was dissolved in MeOH (1 mL) and THF (0.5 mL). A 30 wt % solution of NaOMe in MeOH (17 µL, 0.090 mmol, 1.20 equiv) was added and the solution

was stirred for 1 h at room temperature. The reaction was quenched with saturated $NH_4Cl$ solution (1 mL), extracted with EtOAc (3 x 3 mL), dried with $MgSO_4$, filtered and concentrated. Flash column chromatography on silica gel using petroleum ether/EtOAc (100:1) as the eluent afforded the product as slightly yellow oil with blue fluorescence under UV light (26 mg, 0.072 mmol, 96%). TLC [petroleum ether/EtOAc (100:1)]: $R_f$ = 0.13. $^1$H NMR (400 MHz, $CDCl_3$): $\delta$ = 7.13 (s, 1H, 4-*H*), 6.76 (s, 1H, 8-*H*), 3.76 (t, 2H, $^3J$ = 8.5 Hz, 2-*H*), 3.04 (t, 2H, $^3J$ = 8.6 Hz, 3-*H*), 2.54 (s, 2H, (C=O)C$H_2$), 1.48 (sept, 3H, $^3J$ = 7.6 Hz, C*H*($CH_3$)$_2$), 1.36 (s, 6H, C(C$H_3$)$_2$), 1.13 (d, 18H, $^3J$ = 7.5 Hz, CH(C$H_3$)$_2$). $^{13}$C NMR (100 MHz, $CDCl_3$): $\delta$ = 205.7 (1C, *C*=O), 155.3 (1C, *C*-4a), 152.8 (1C, *C*-8a), 142.3 (1C, *C*-3a), 135.1 (1C, *C*-7a), 118.7 (1C, *C*-4), 101.6 (1C, *C*-8), 53.7 (1C, *C*-6), 50.9 (1C, *C*-2), 37.5 (1C, *C*-5), 30.7 (1C, *C*-3), 30.2 (2C, C(*C*$H_3$)$_2$), 18.5 (6C, CH(*C*$H_3$)$_2$), 12.8 (3C, *C*H($CH_3$)$_2$). IR (diamond ATR): $\tilde{\nu}$ = 2948 (m), 2890 (m), 2865 (m), 1700 (s), 1611 (m), 1468 (s), 1440 (s), 1364 (m), 1336 (w), 1295 (s), 1264 (s), 1172 (w), 1134 (m), 1056 (m), 1040 (m), 1015 (m), 998 (s), 954 (m), 922 (m), 881 (s), 850 (m), 807 (w), 752 (m), 729 (w), 684 (m), 641 (m), 612 (w), 570 (m). UV/Vis (MeOH): $\lambda_{max}$ (log $\varepsilon$) = 380 (3.55), 250 (4.46), 201 (4.20). HRESIMS: calculated ([$C_{22}H_{35}NOSiNa^+$]) 380.23801, found 380.23817 (0.42 ppm).

## 3. References:


[S1] Li, B. T. Y.; White, J. M.; Hutton, C. A. *Aust. J. Chem.* **2010**, *63*, 438-444.

[S2] Marsch, N.; Jones, P. G.; Lindel, T. *Beilstein J. Org. Chem.* **2015**, *11*, 1700-1706.

[S3] Dudnik, A. S.; Chernyak, N.; Huang, C.; Gevorgyan, V. *Angew. Chem. Int. Ed.* **2010**, *49*, 8729-8732.

## 4. Spectra

$^{1}$H NMR ($CDCl_3$, 400 MHz)

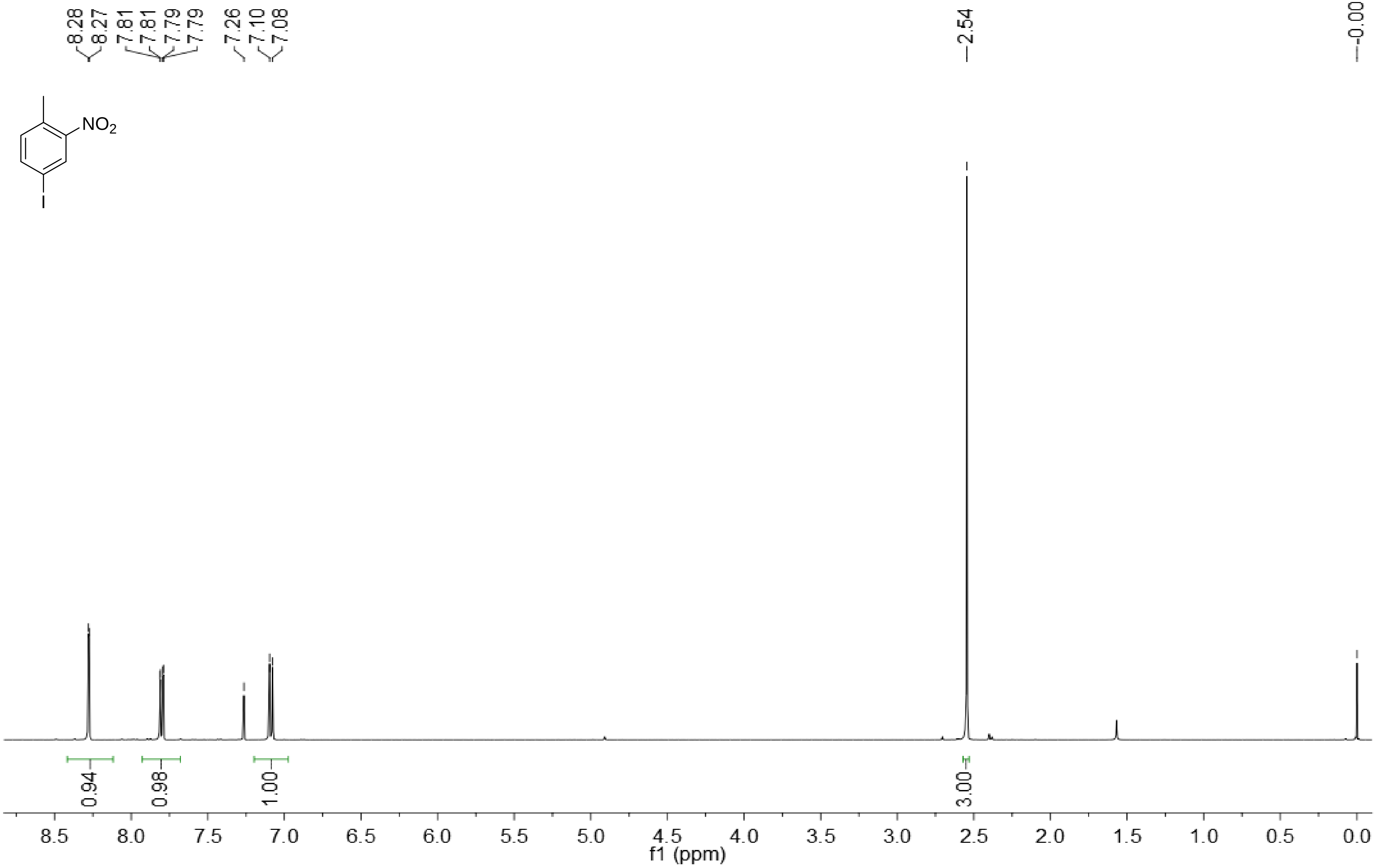

$^{13}C$ NMR ($CDCl_3$, 100 MHz)

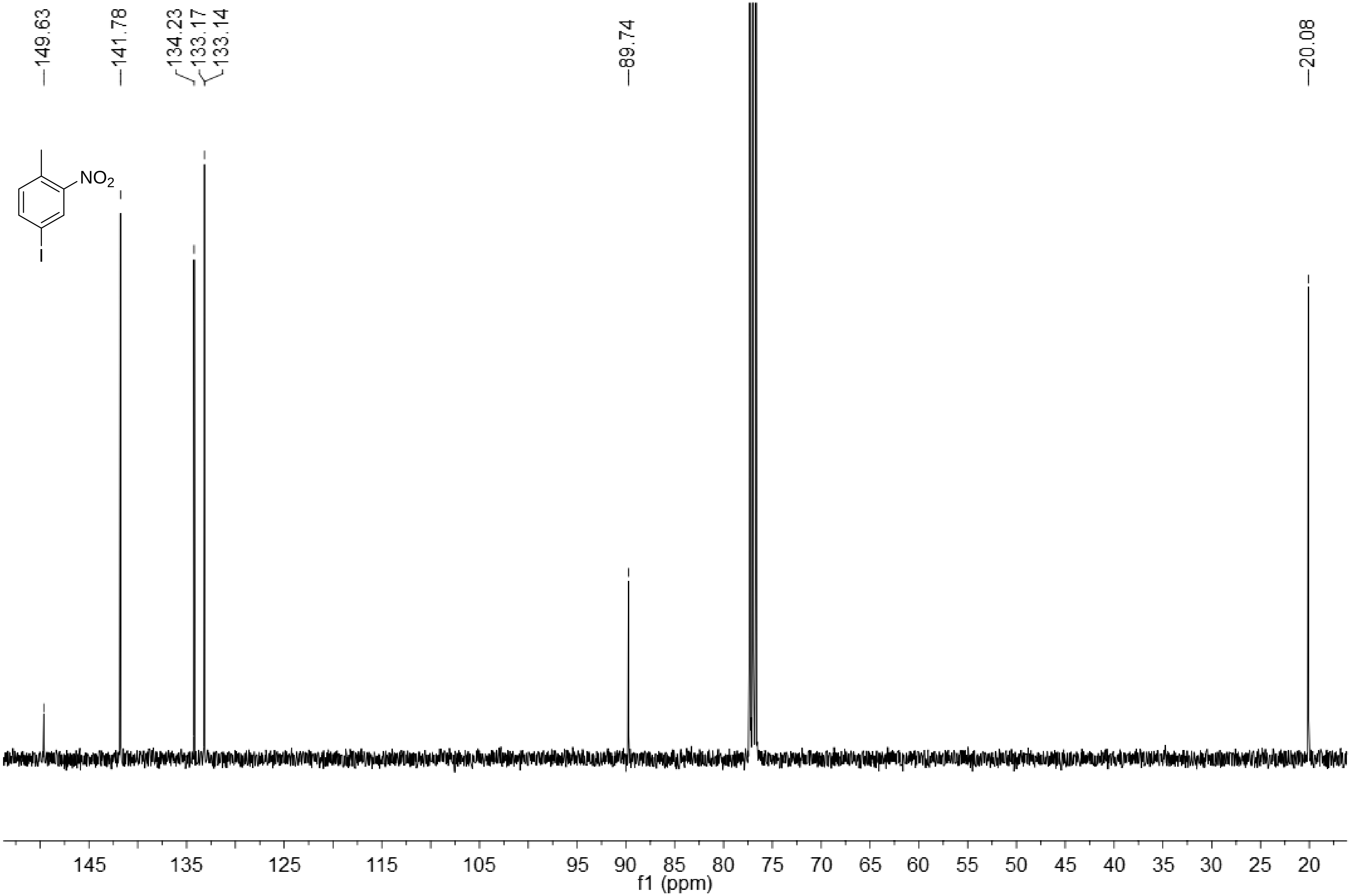

$^1$H NMR ($CDCl_3$, 400 MHz)

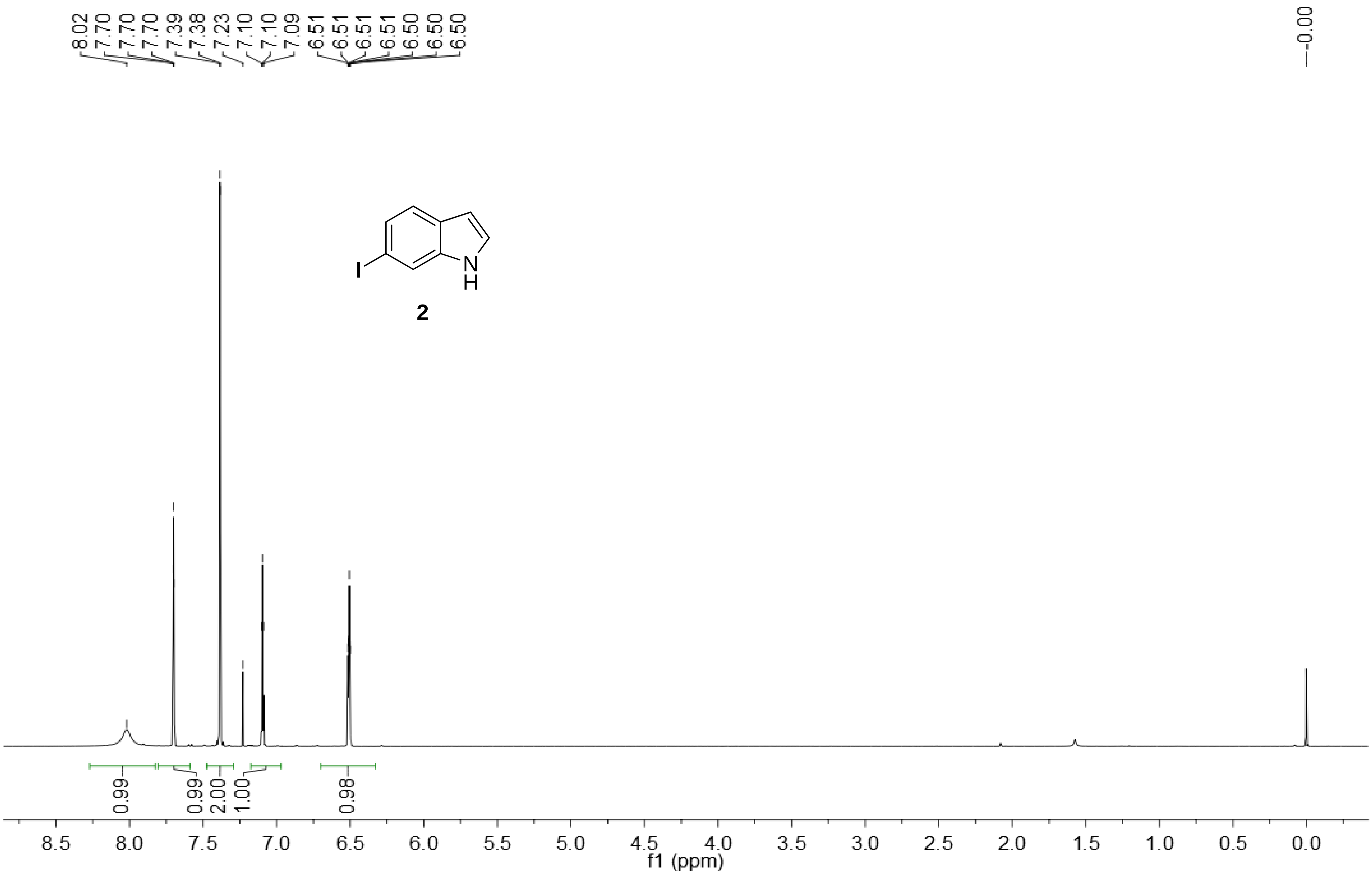

$^{13}C$ NMR ($CDCl_3$, 100 MHz)

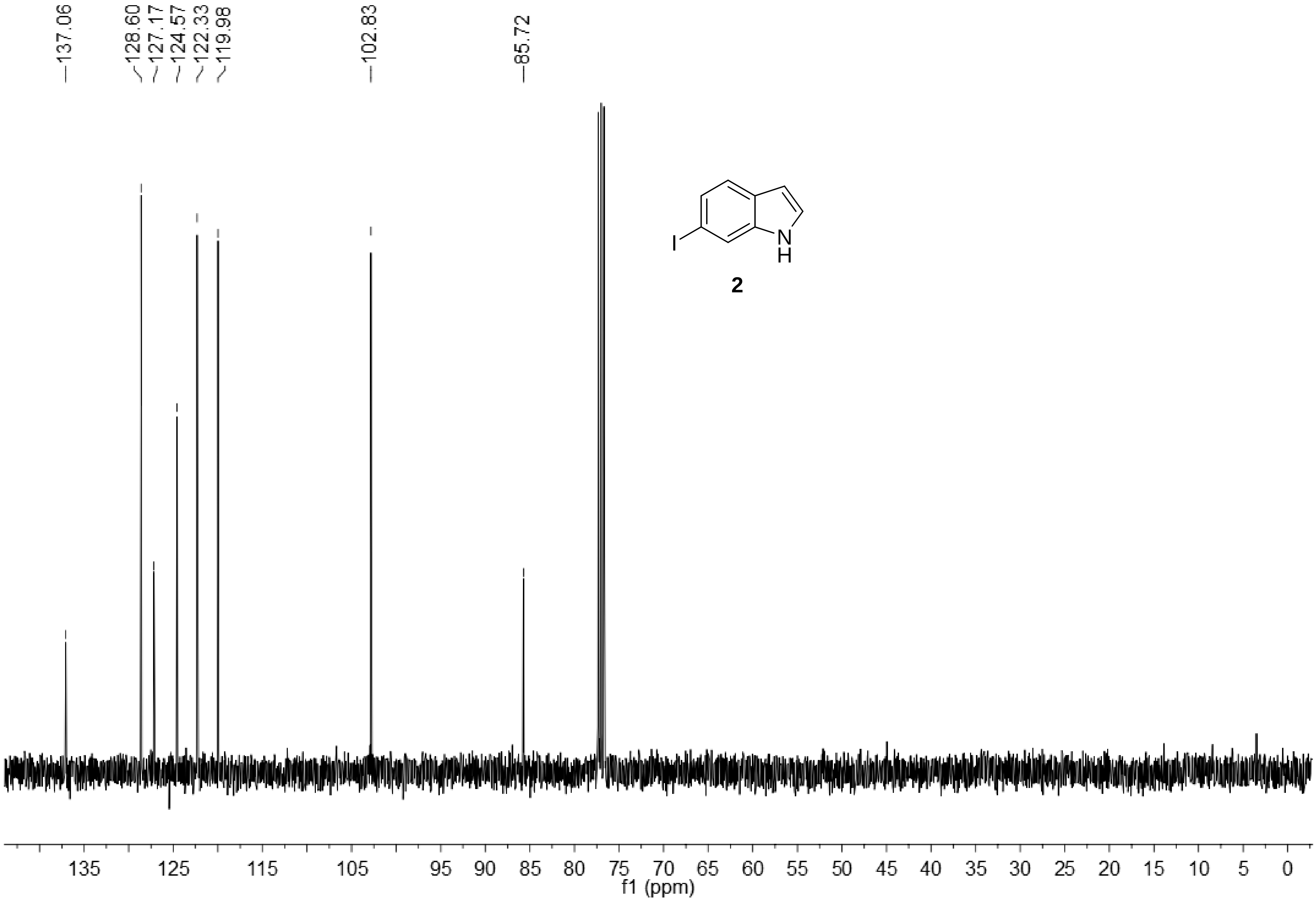

$^{1}H$ NMR ($CDCl_3$, 400 MHz)

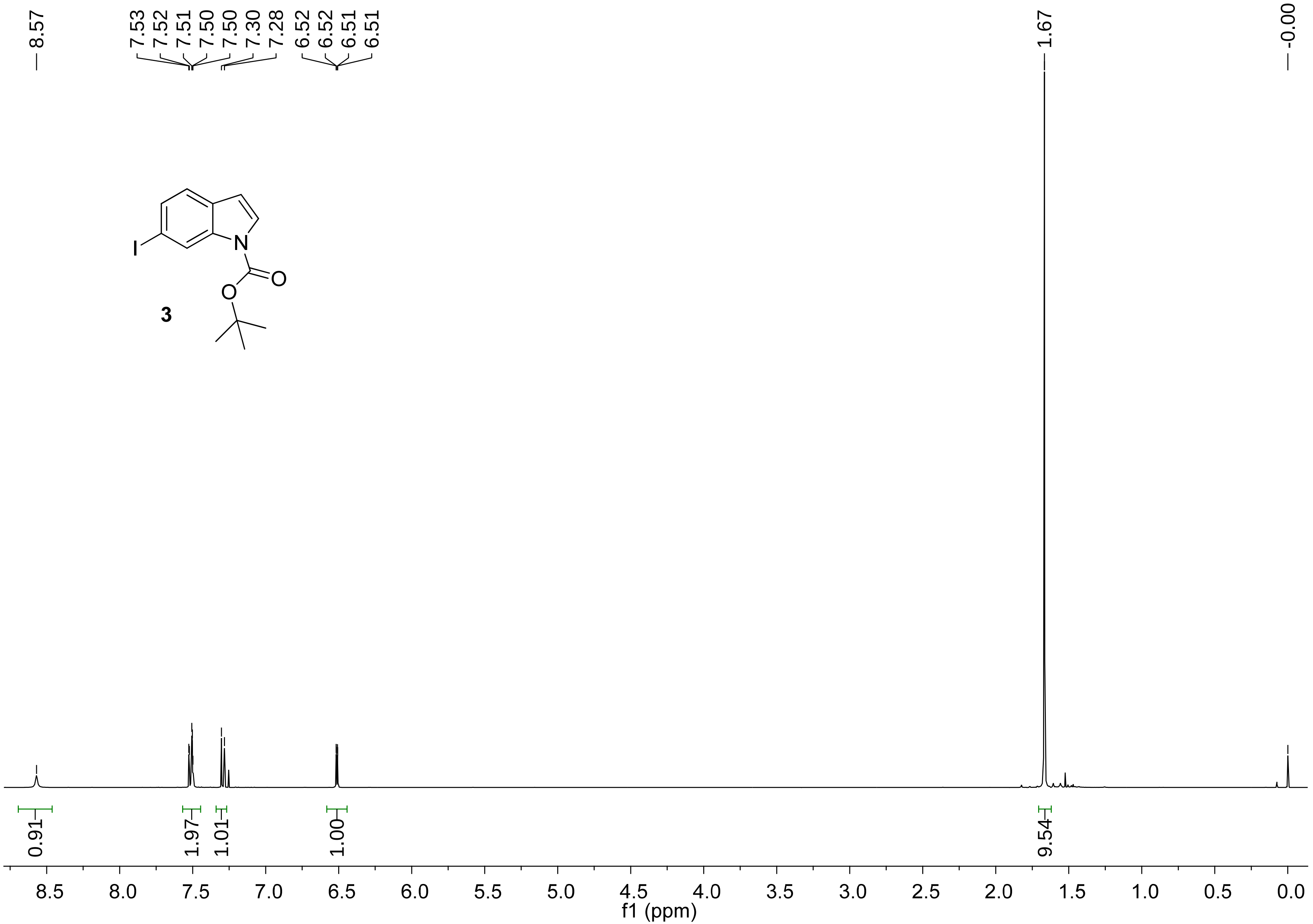

$^{13}C$ NMR ($CDCl_3$, 100 MHz)

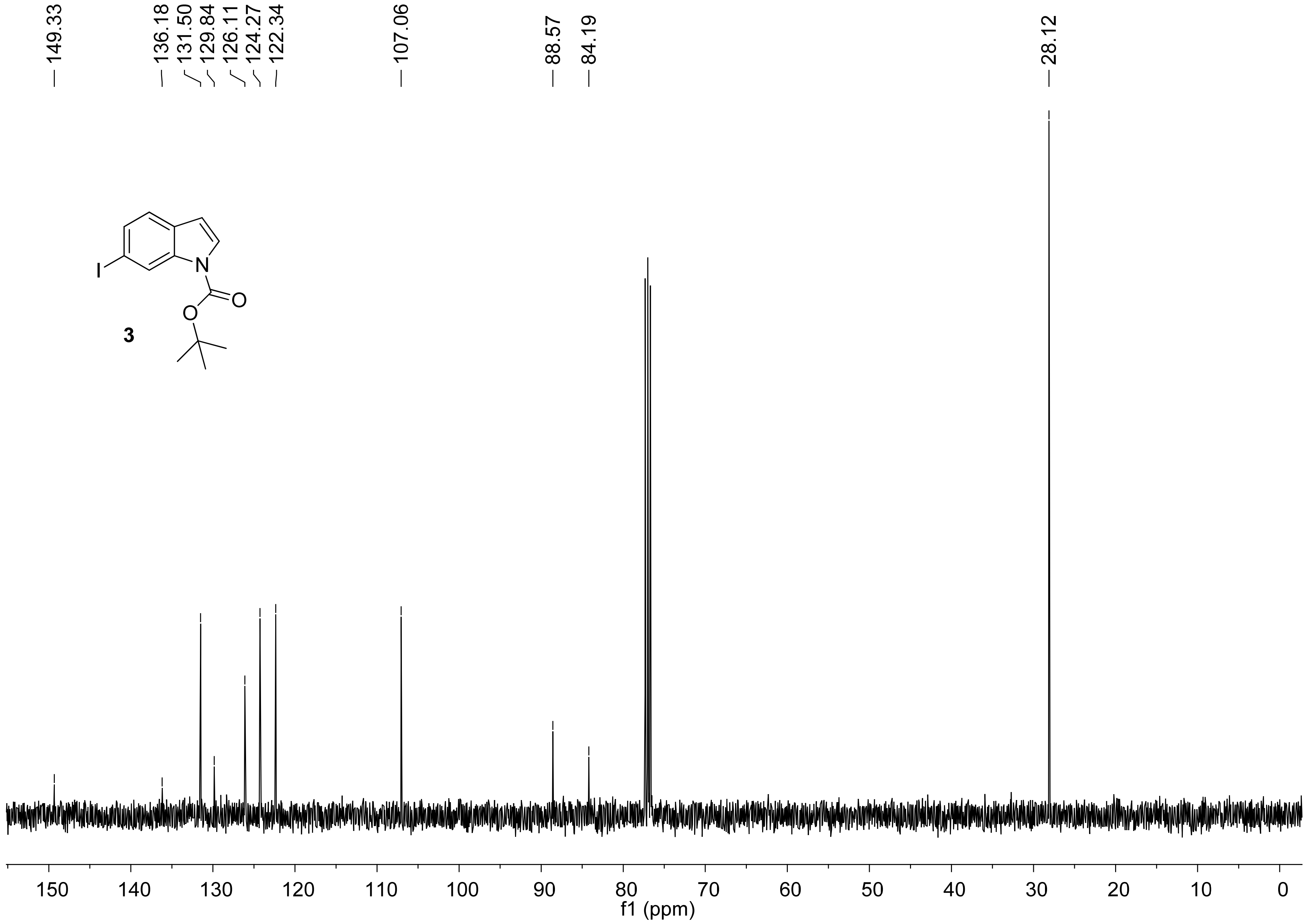

$^{1}$H NMR ($CDCl_3$, 400 MHz, referenced to solvent signal)

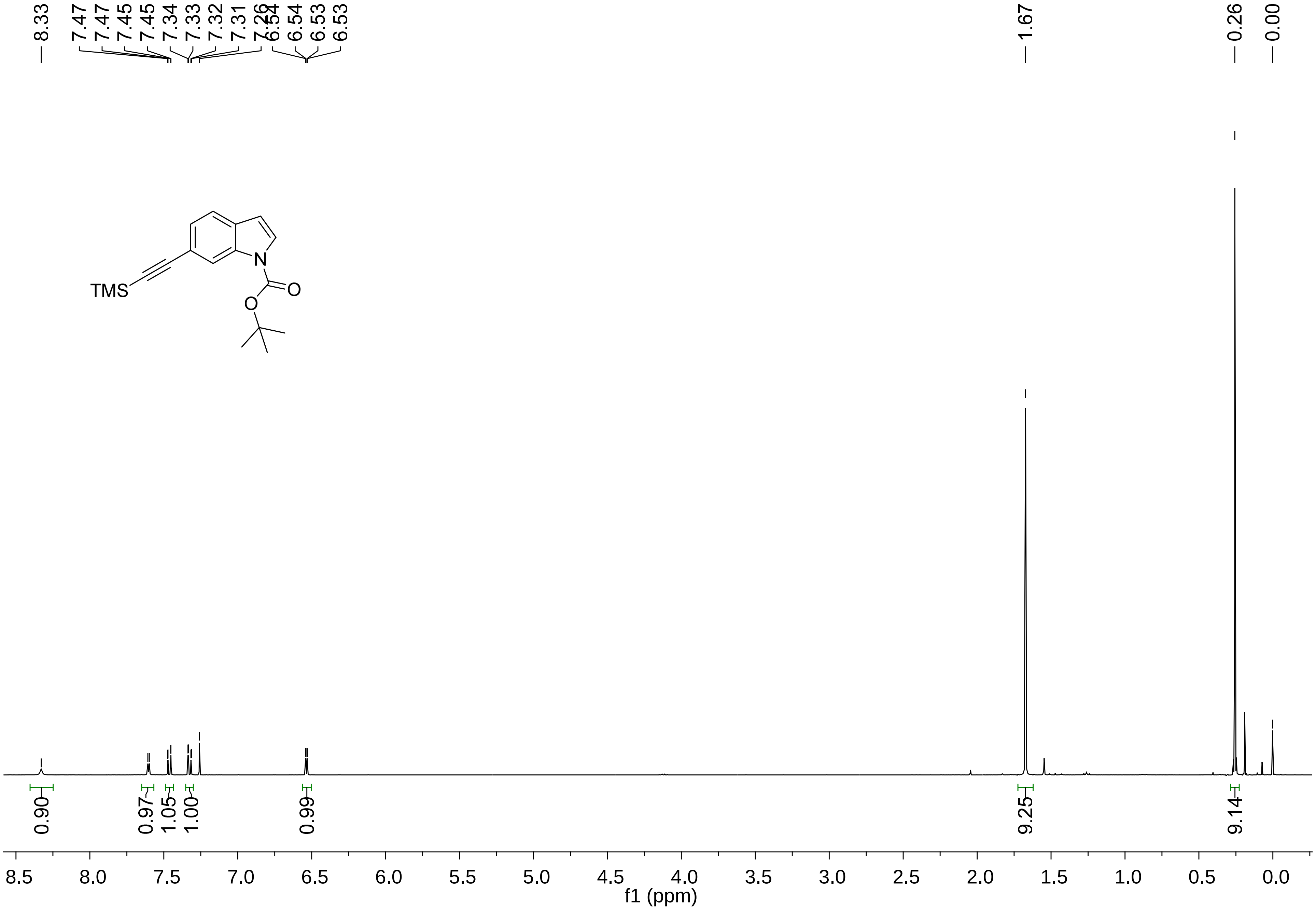

$^{13}C$ NMR ($CDCl_3$, 100 MHz, referenced to solvent signal)

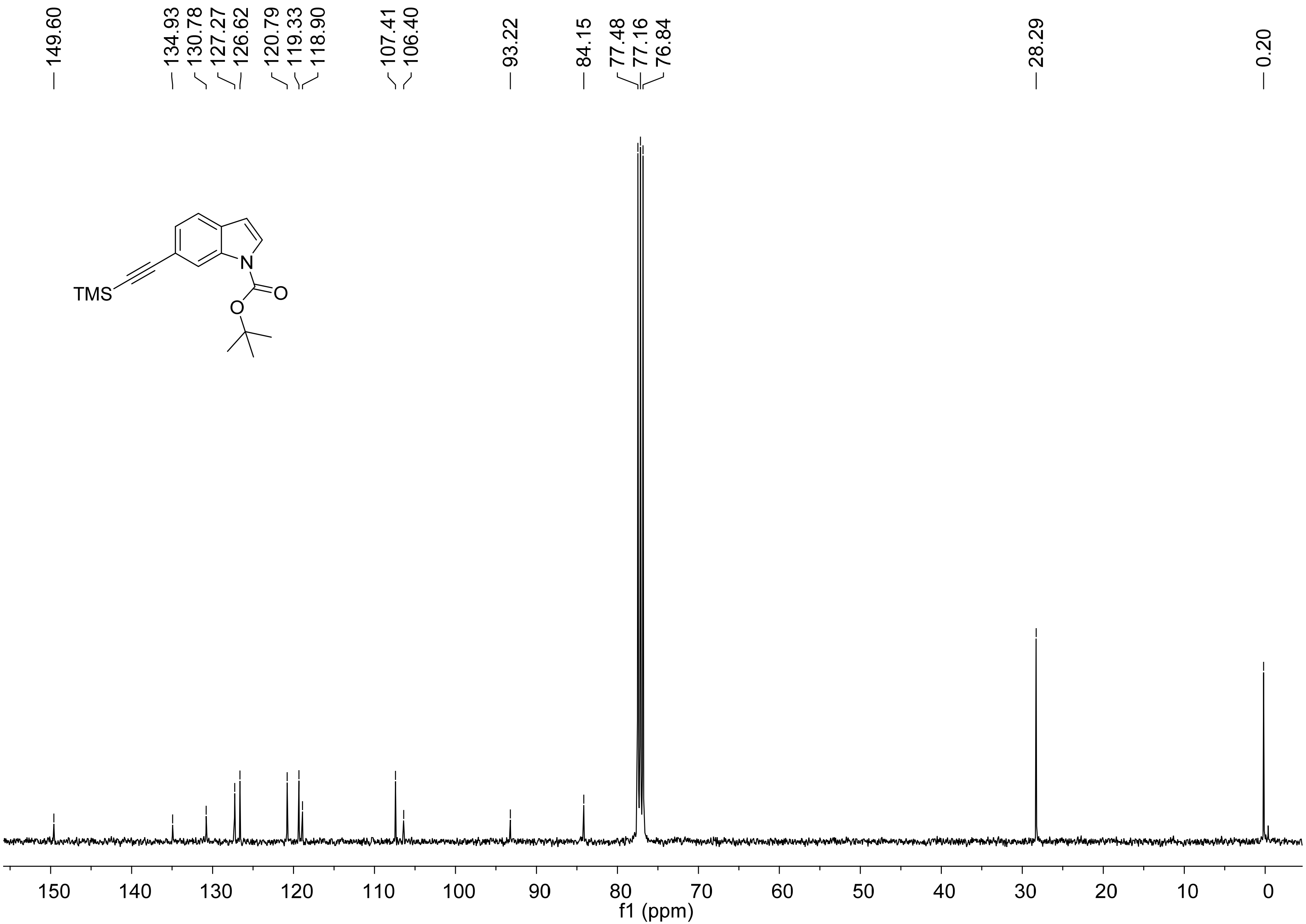

$^{1}$H NMR ($CDCl_3$, 400 MHz)

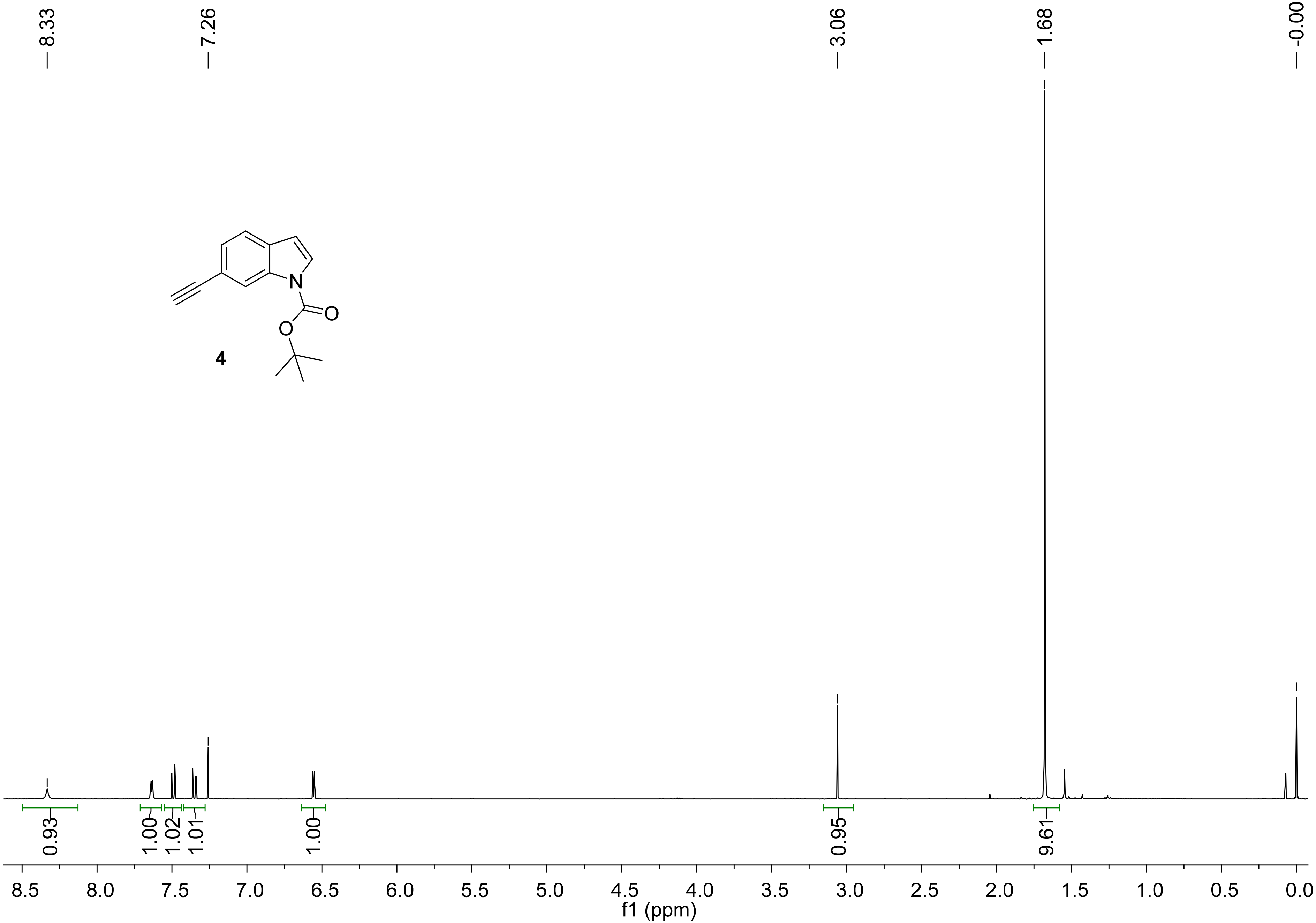

$^{13}C$ NMR ($CDCl_3$, 100 MHz)

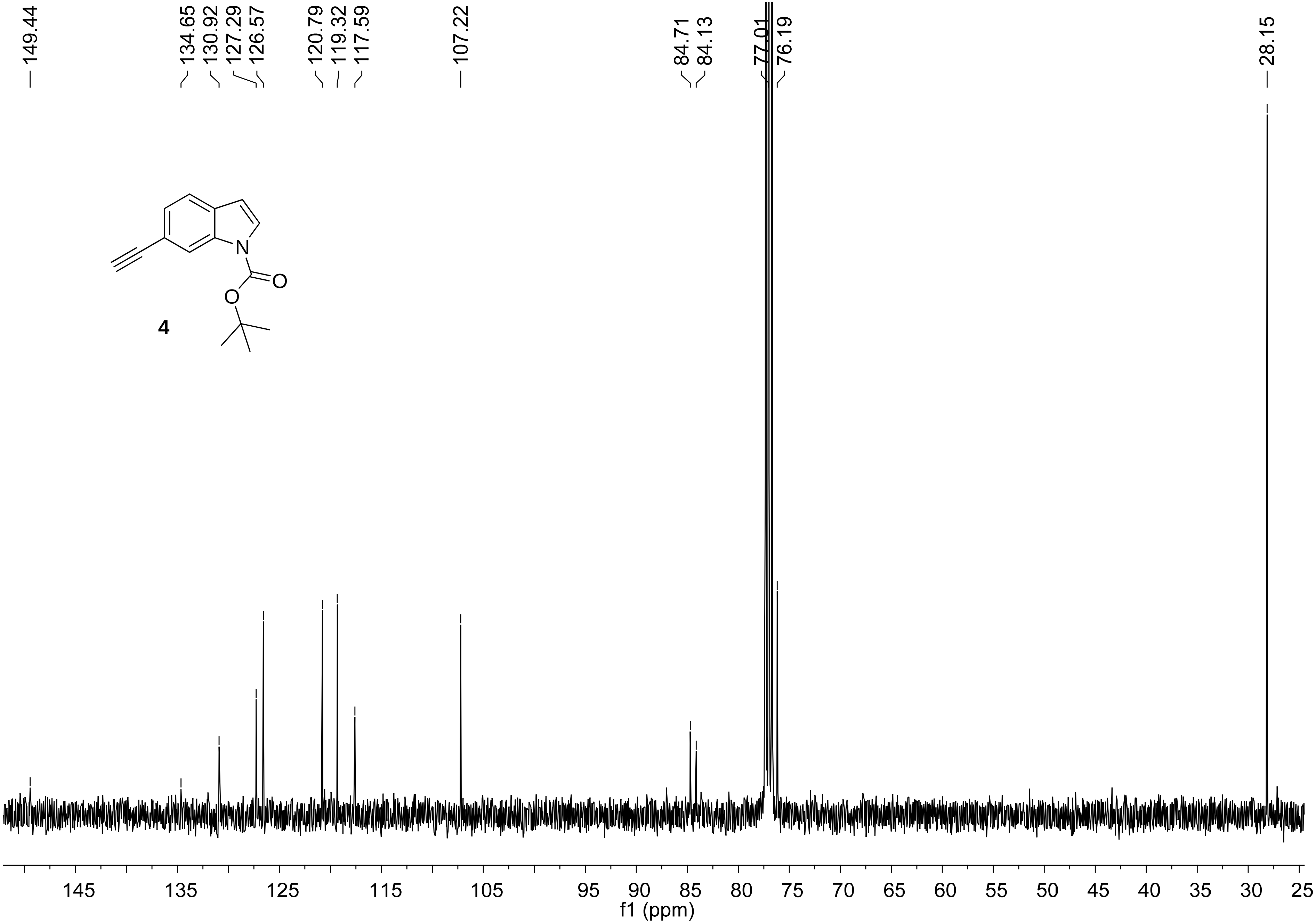

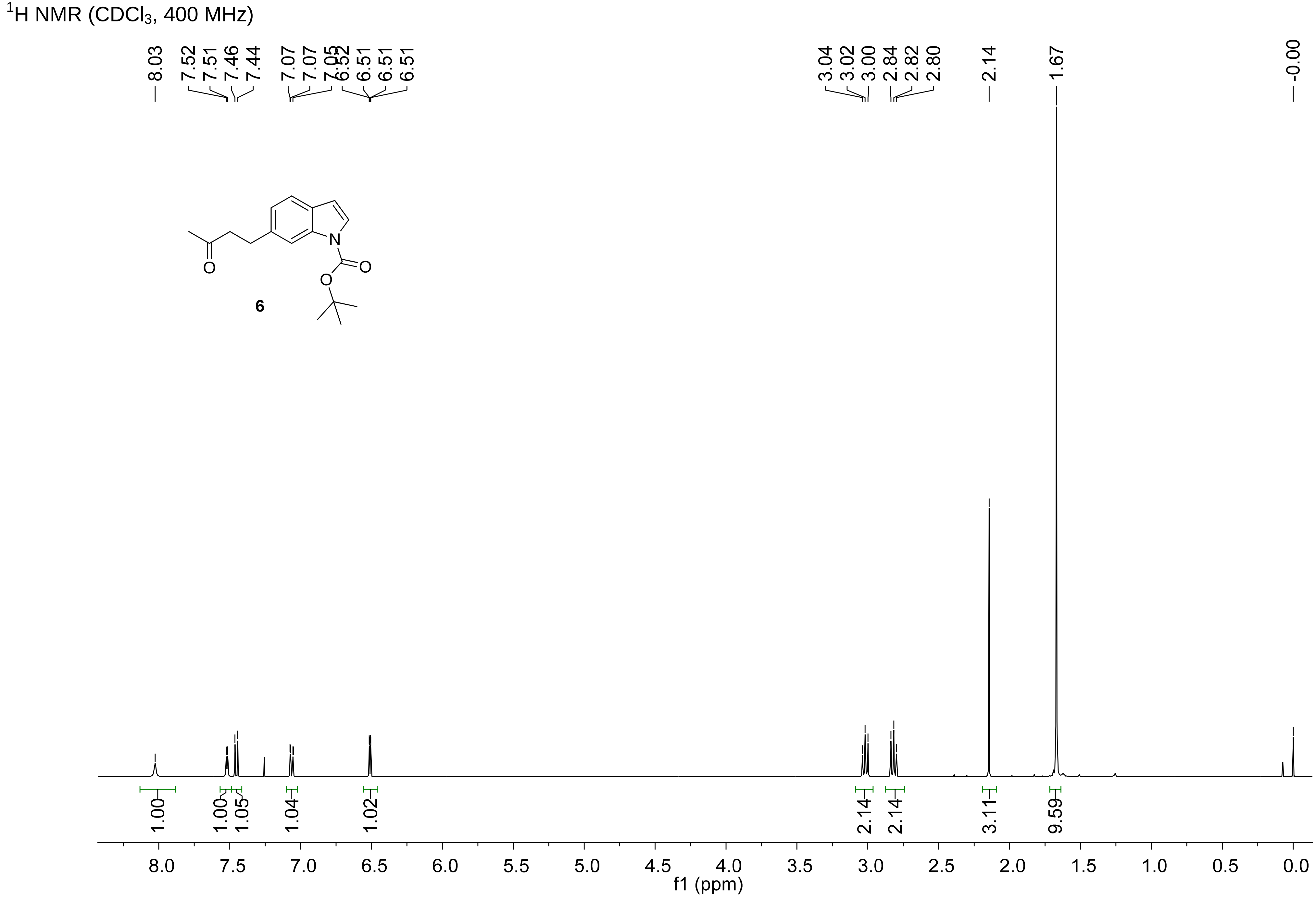

$^{1}$H NMR (CDCl3, 400 MHz)
8.03
7.52
7.51
7.46
7.44
7.07
7.07
7.05
6.52
6.51
6.51
6.51
3.04
3.02
3.00
2.84
2.82
2.80
2.14
1.67
-0.00
6
1.00
1.00
1.05
1.04
1.02
2.14
2.14
3.11
9.59
f1 (ppm)

$^{13}$C NMR ($CDCl_3$, 100 MHz)

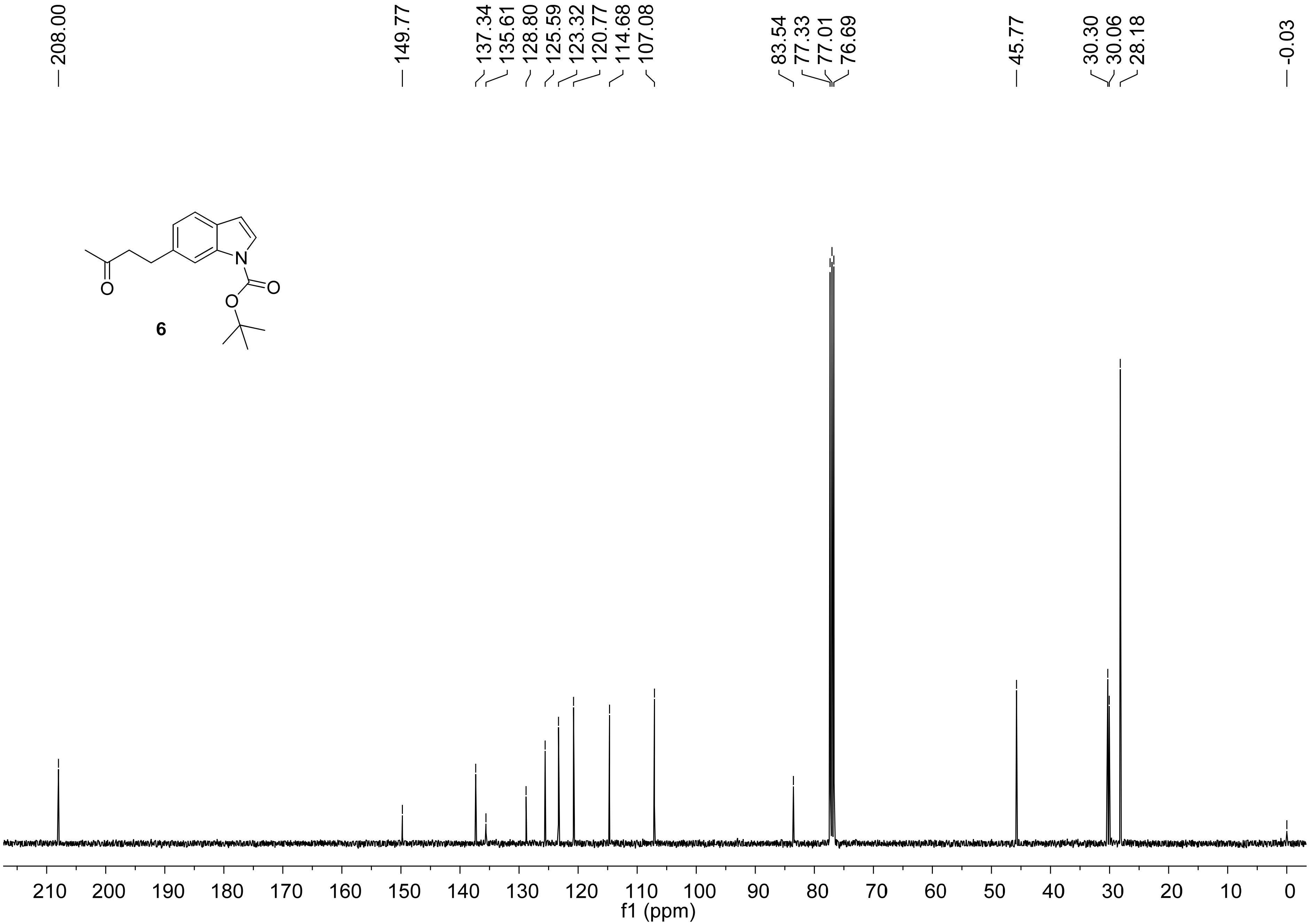

$^{1}H$ NMR ($CDCl_3$, 400 MHz)

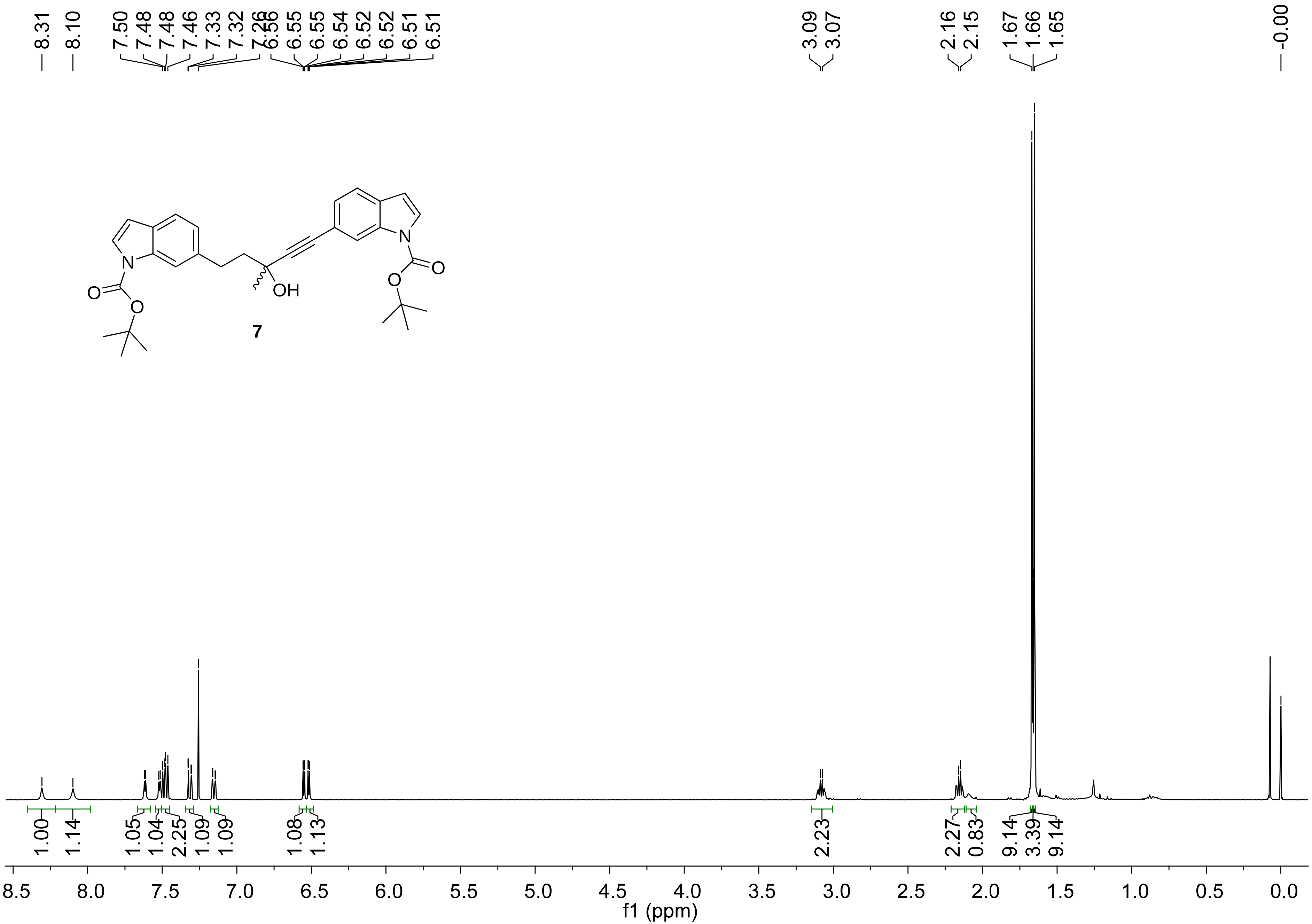

$^{13}C$ NMR ($CDCl_3$, 100 MHz)

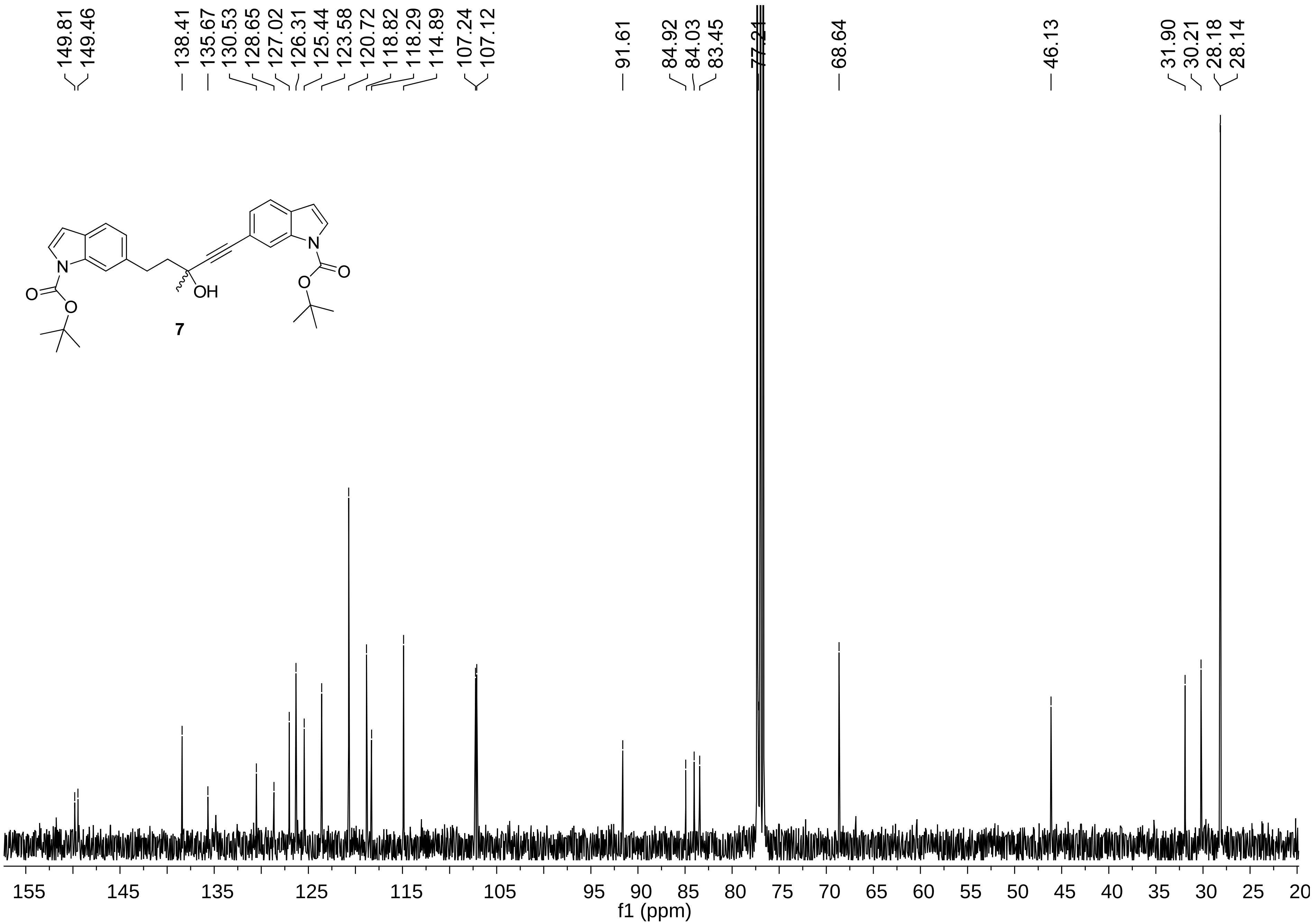

$^{1}H$ NMR ($CDCl_3$, 600 MHz)

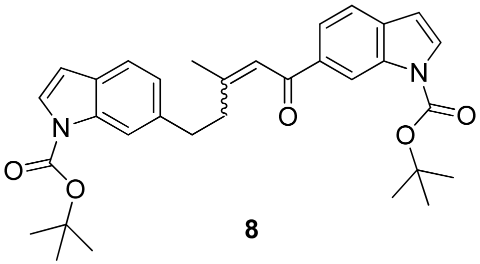

8

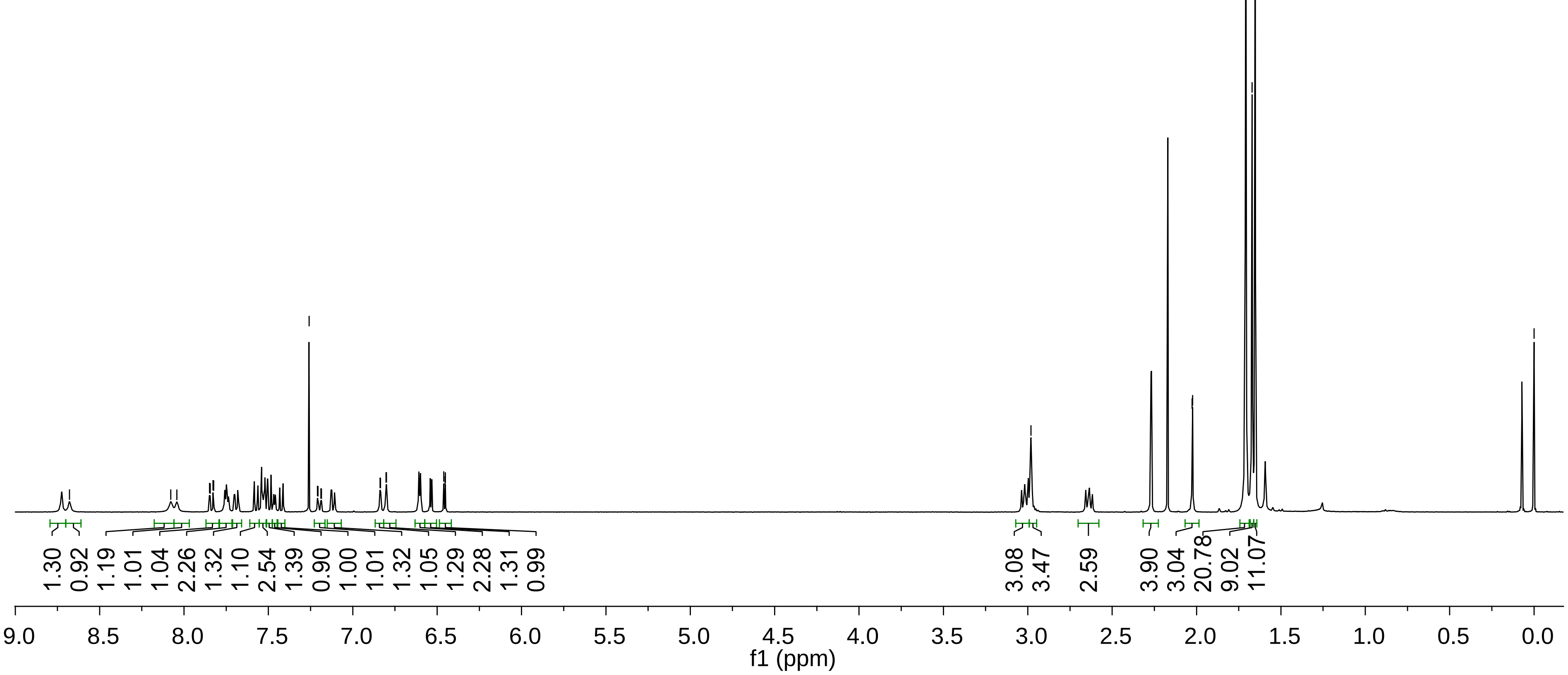

$^{13}C$ NMR ($CDCl_3$, 150 MHz)

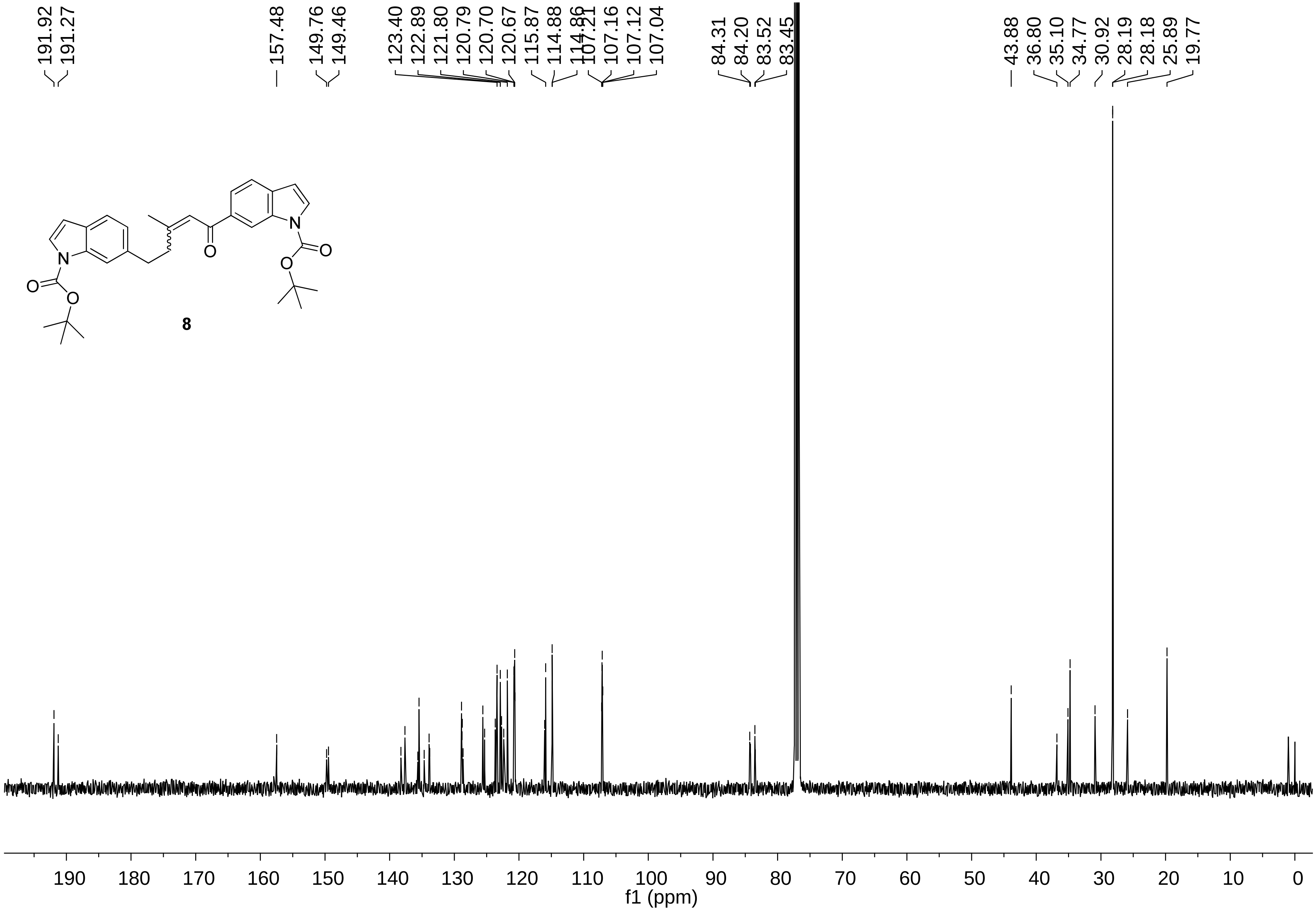


8

$^{1}$H NMR ($CDCl_3$, 400 MHz)

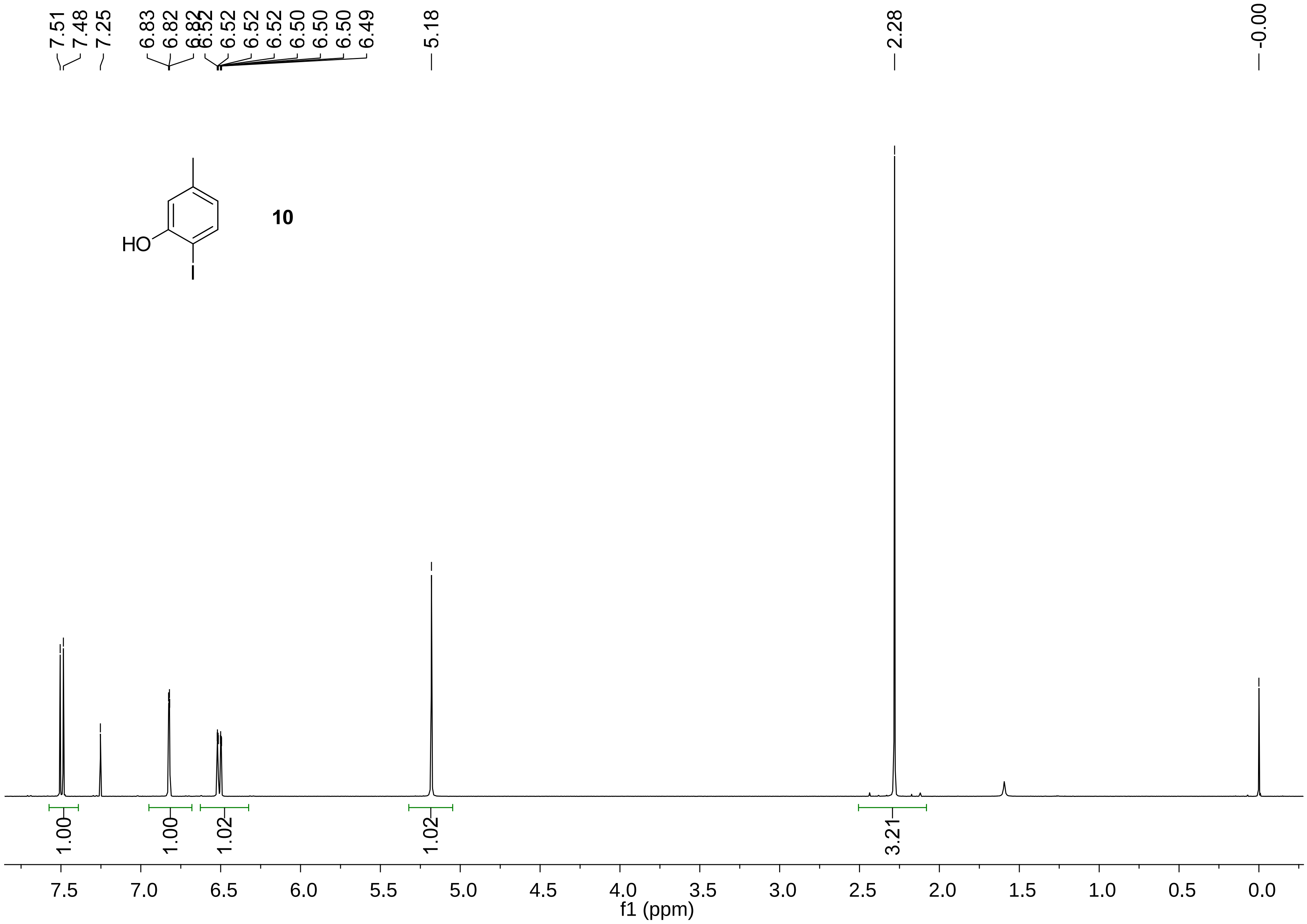

$^{13}C$ NMR ($CDCl_3$, 100 MHz)

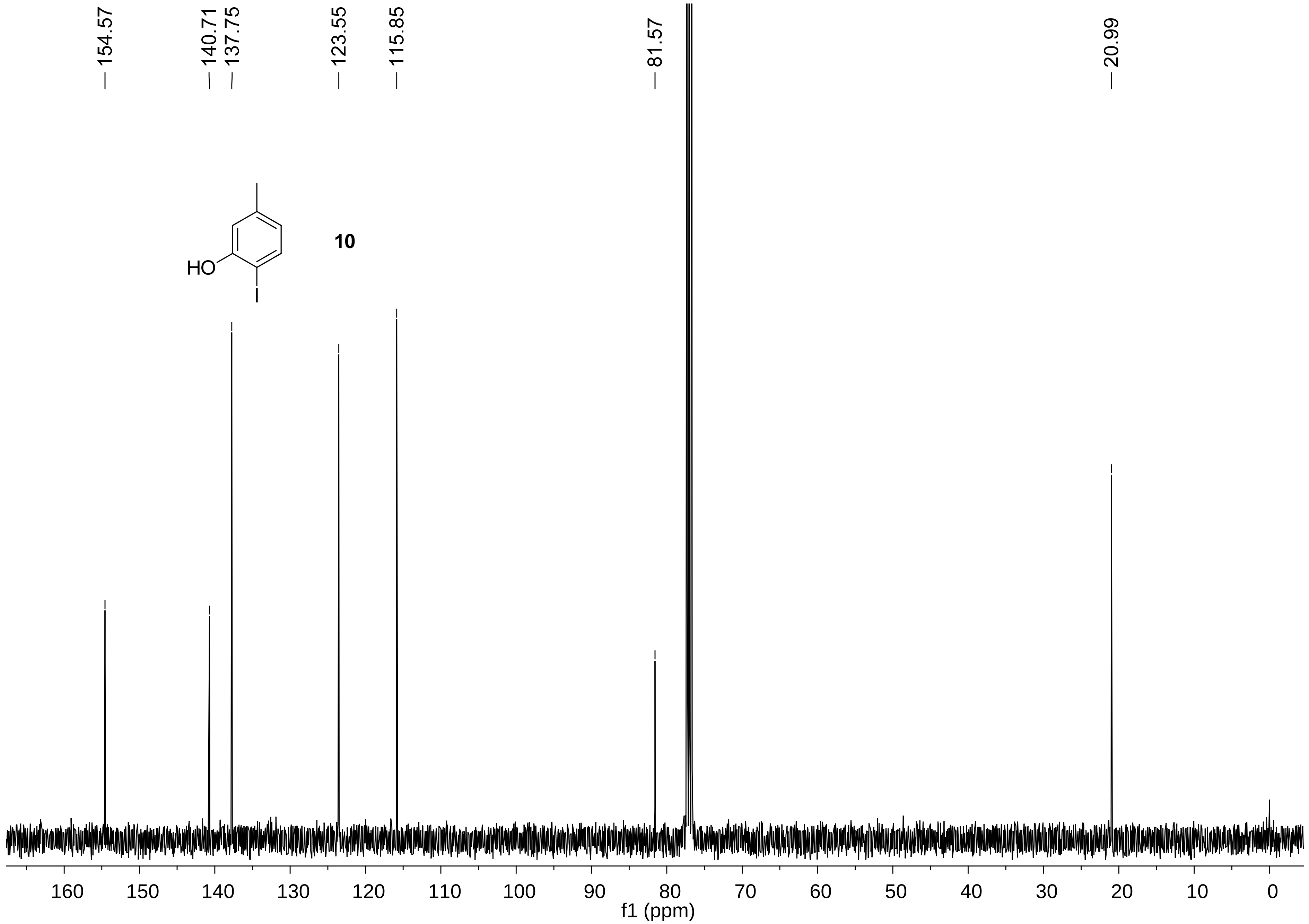

$^{1}H$ NMR ($CDCl_3$, 400 MHz)

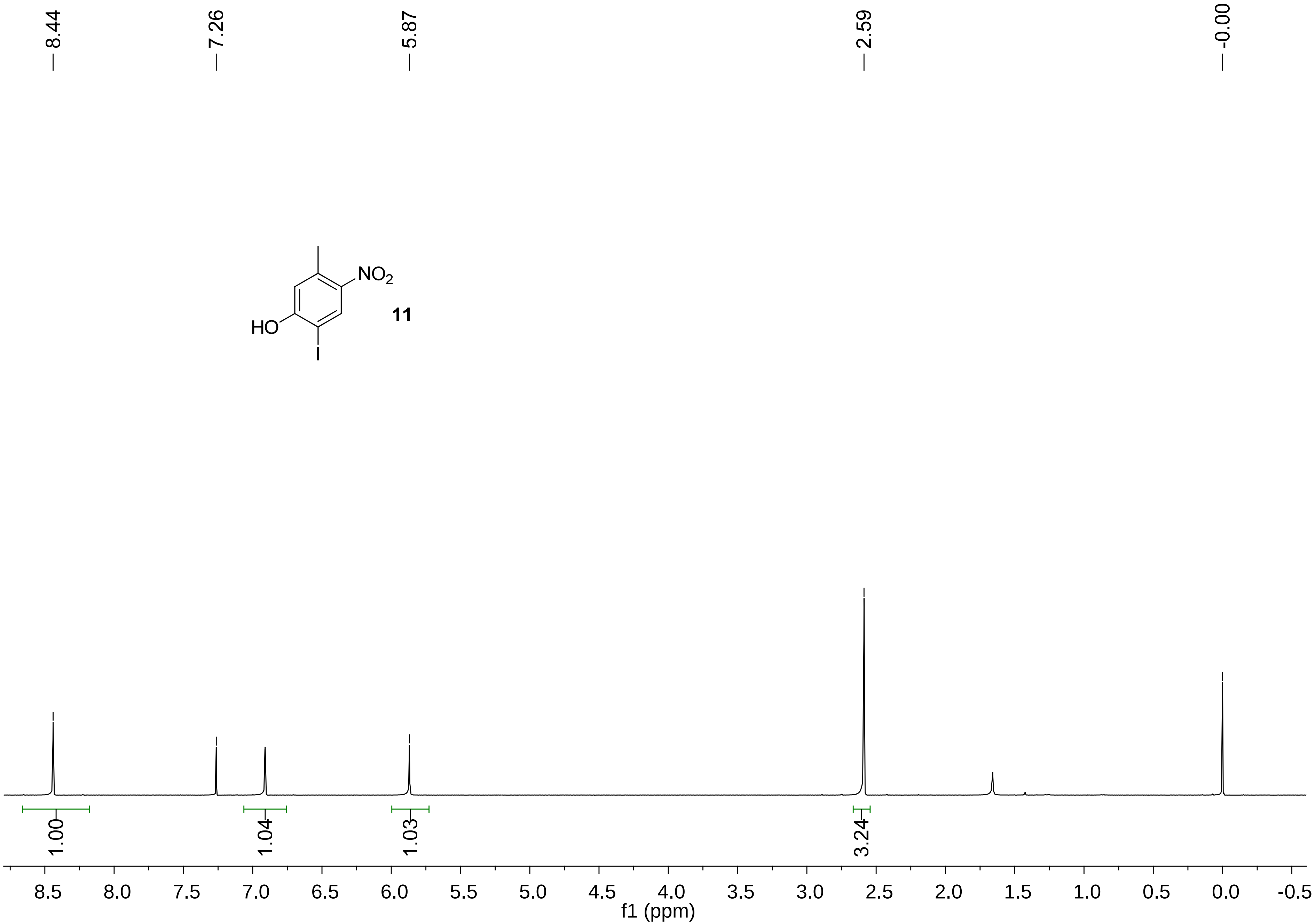

$^{13}C$ NMR ($CDCl_3$, 100 MHz)

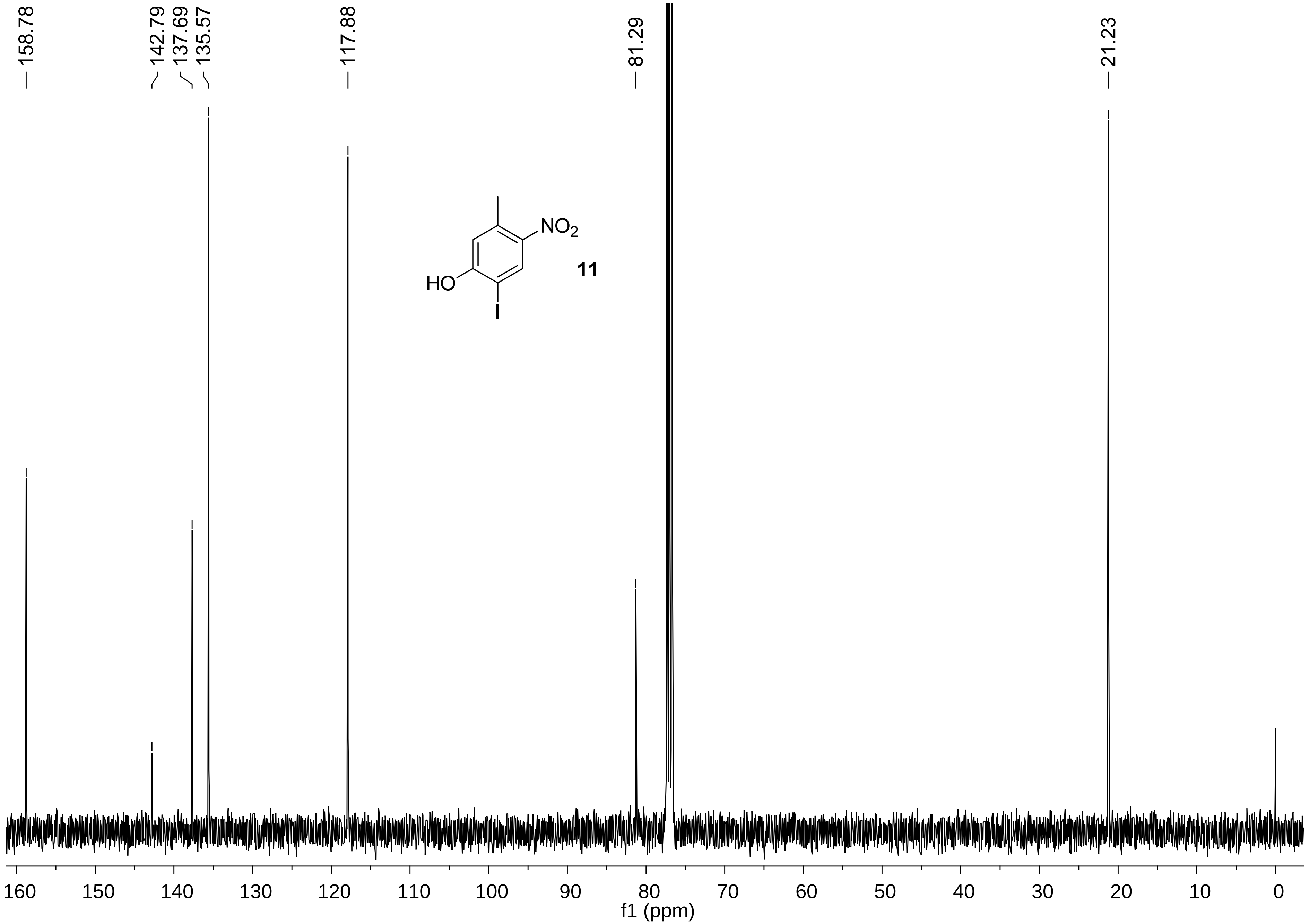

$^{1}$H NMR ($CDCl_3$, 400 MHz)

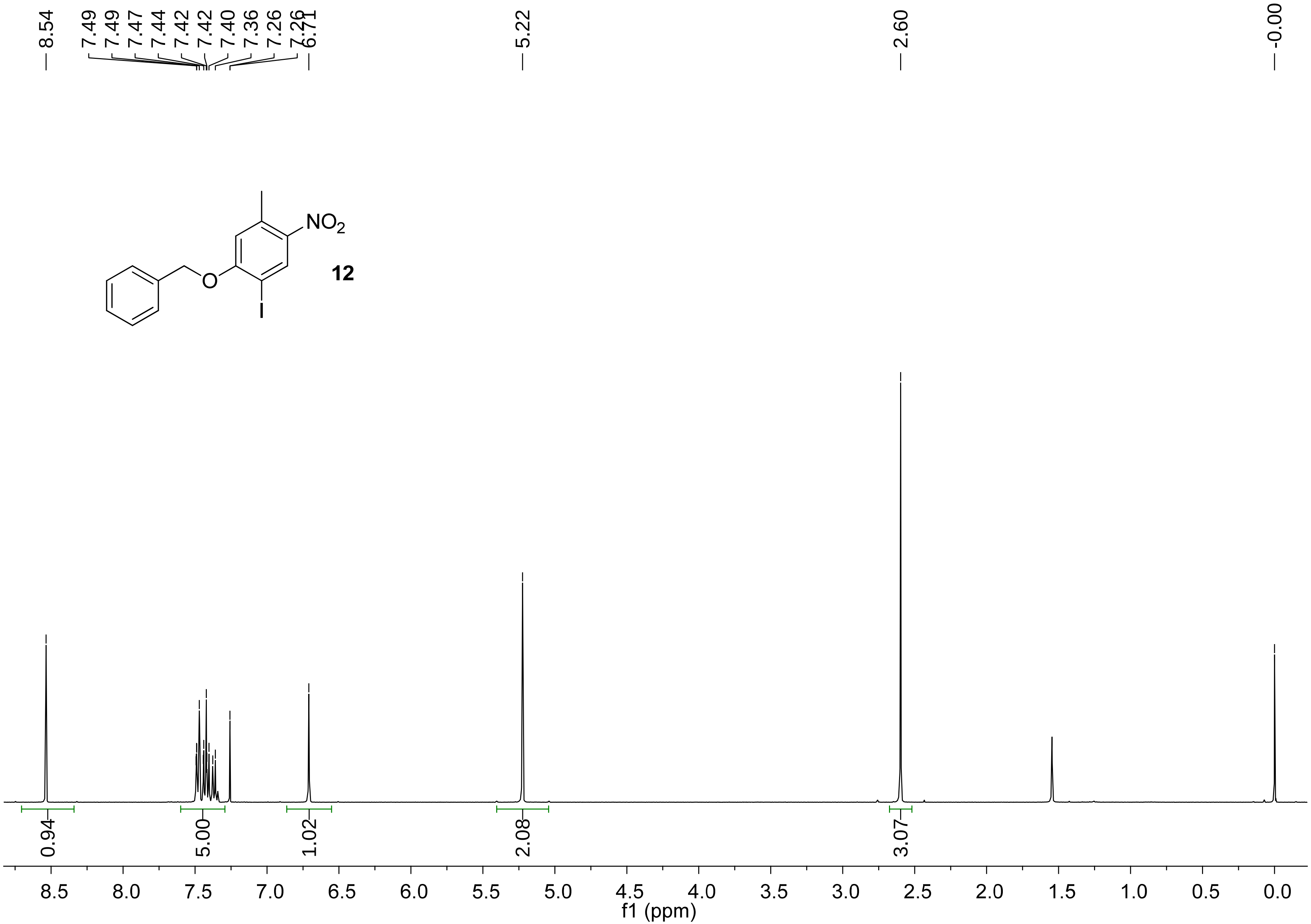


**12**

$^{13}C$ NMR ($CDCl_3$, 100 MHz)

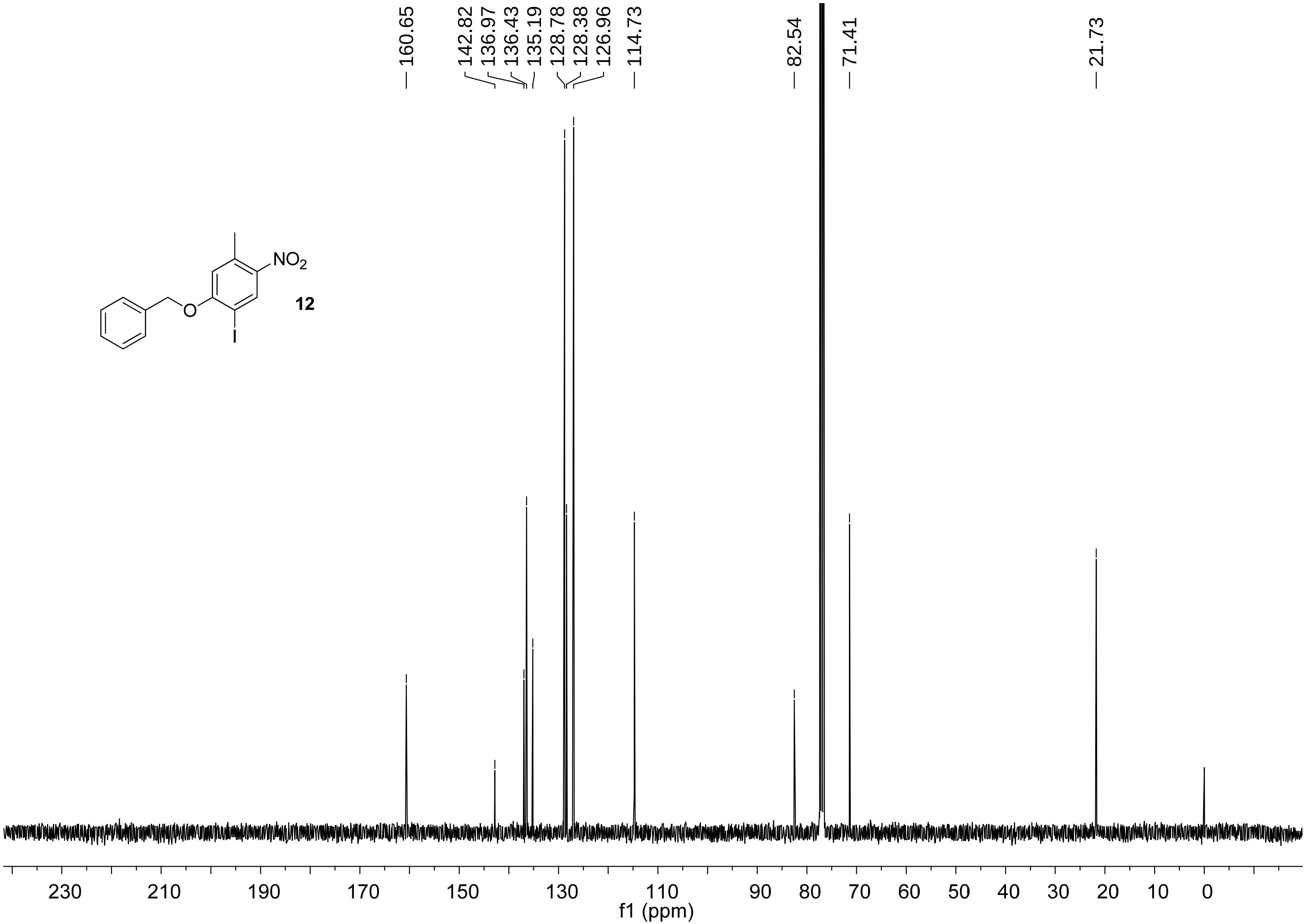

$^1$H NMR ($CDCl_3$, 400 MHz)

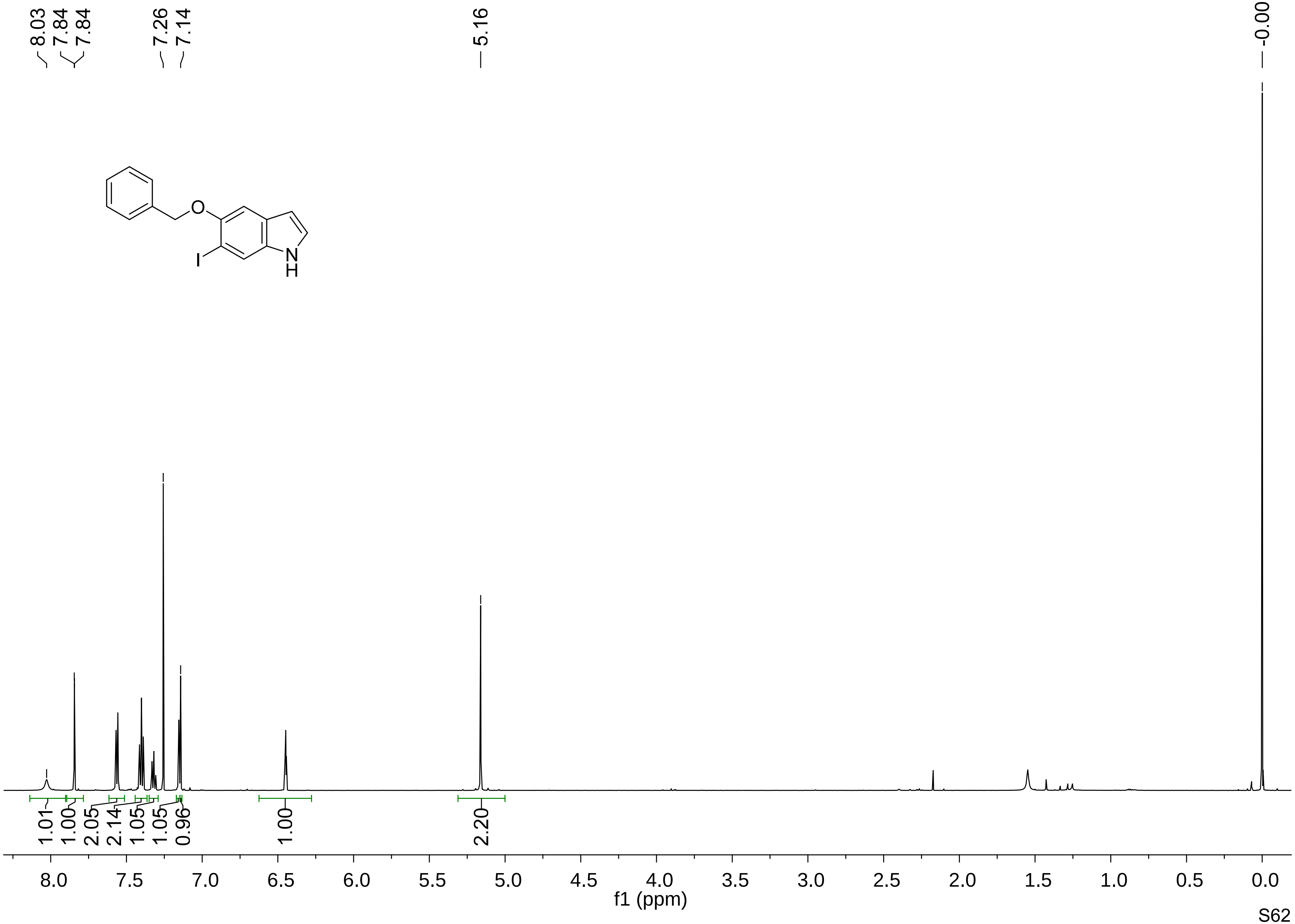

$^{13}C$ NMR ($CDCl_3$, 100 MHz)

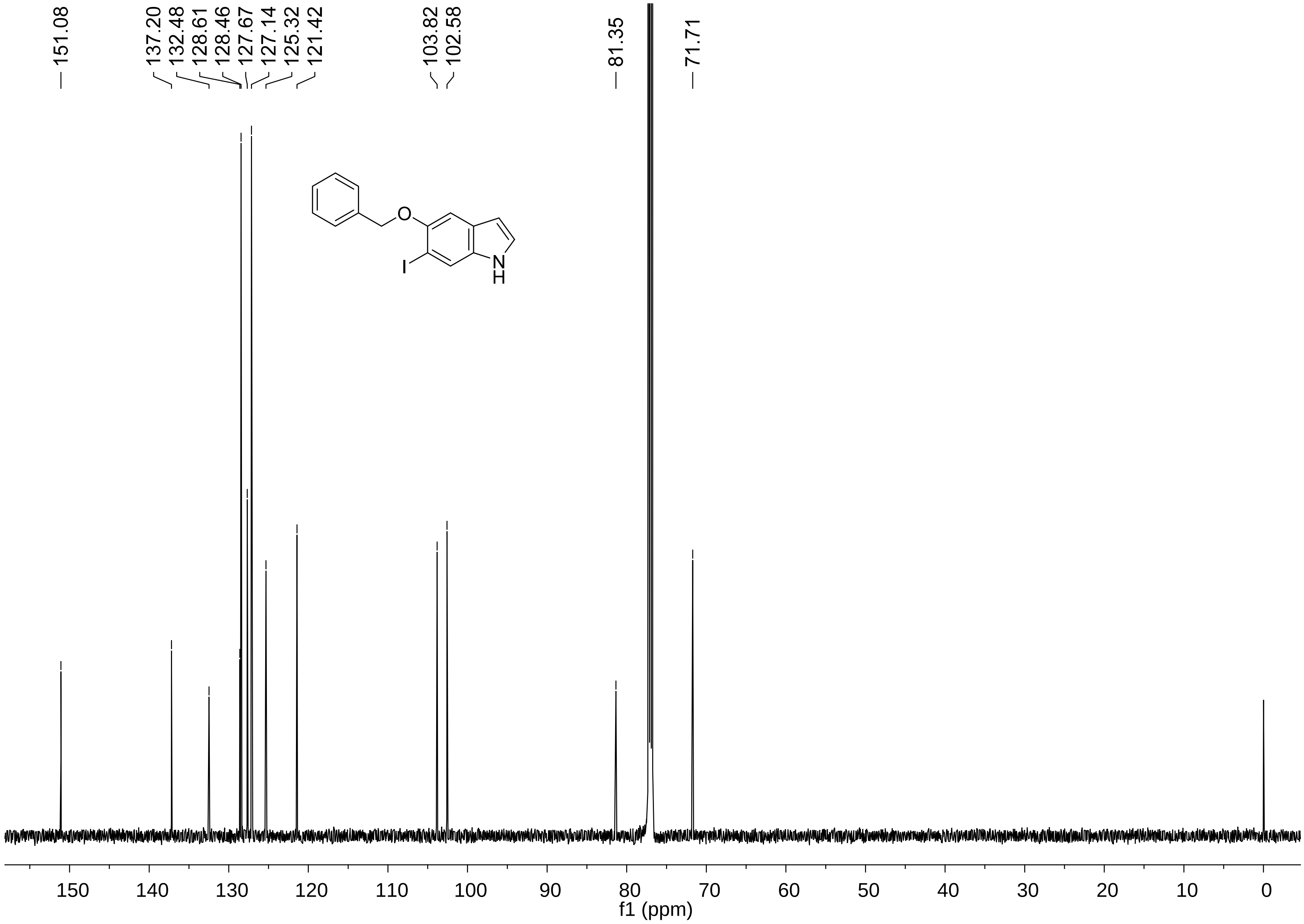

$^{1}H$ NMR ($CDCl_3$, 400 MHz)

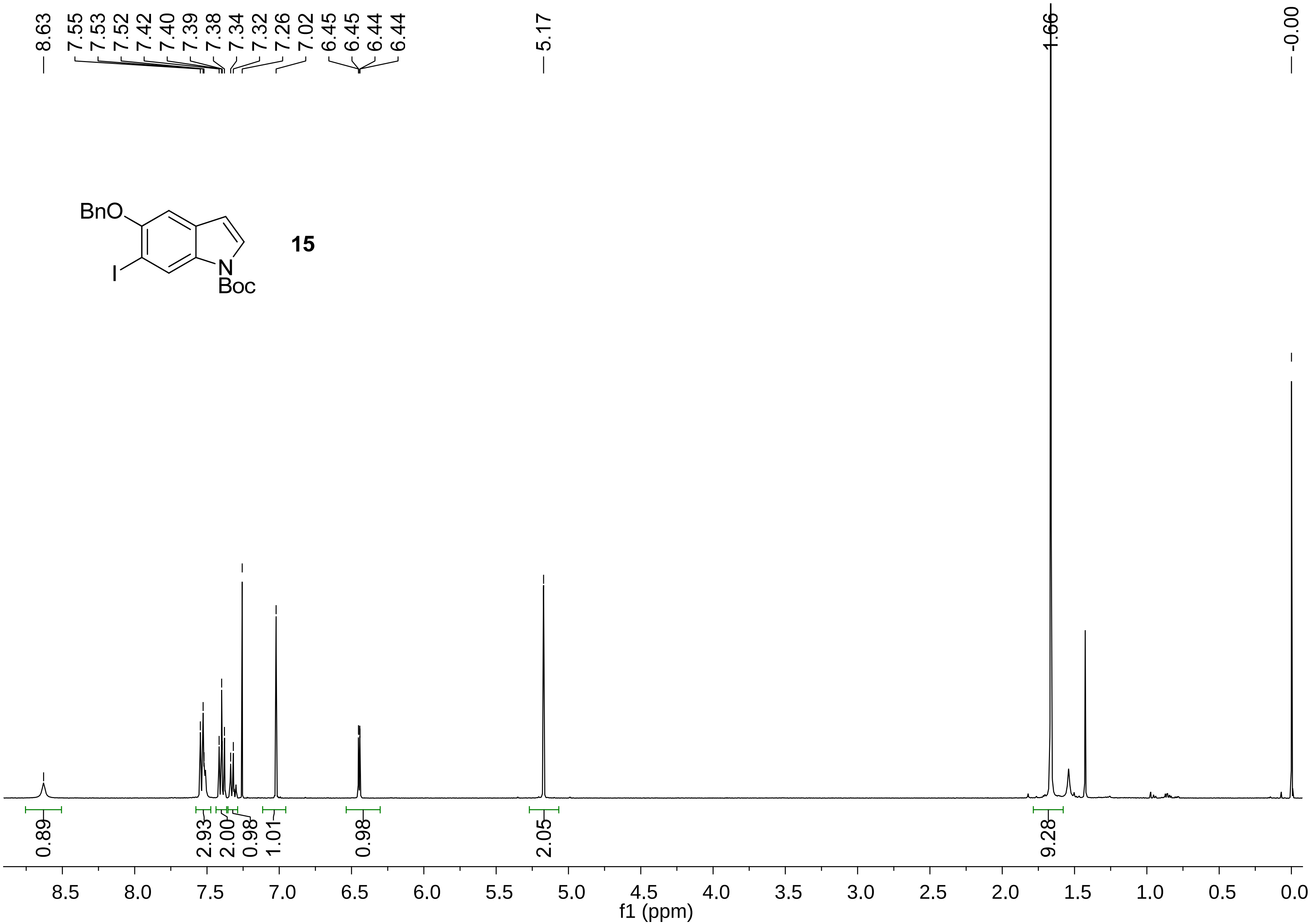

$^{13}C$ NMR ($CDCl_3$, 100 MHz)

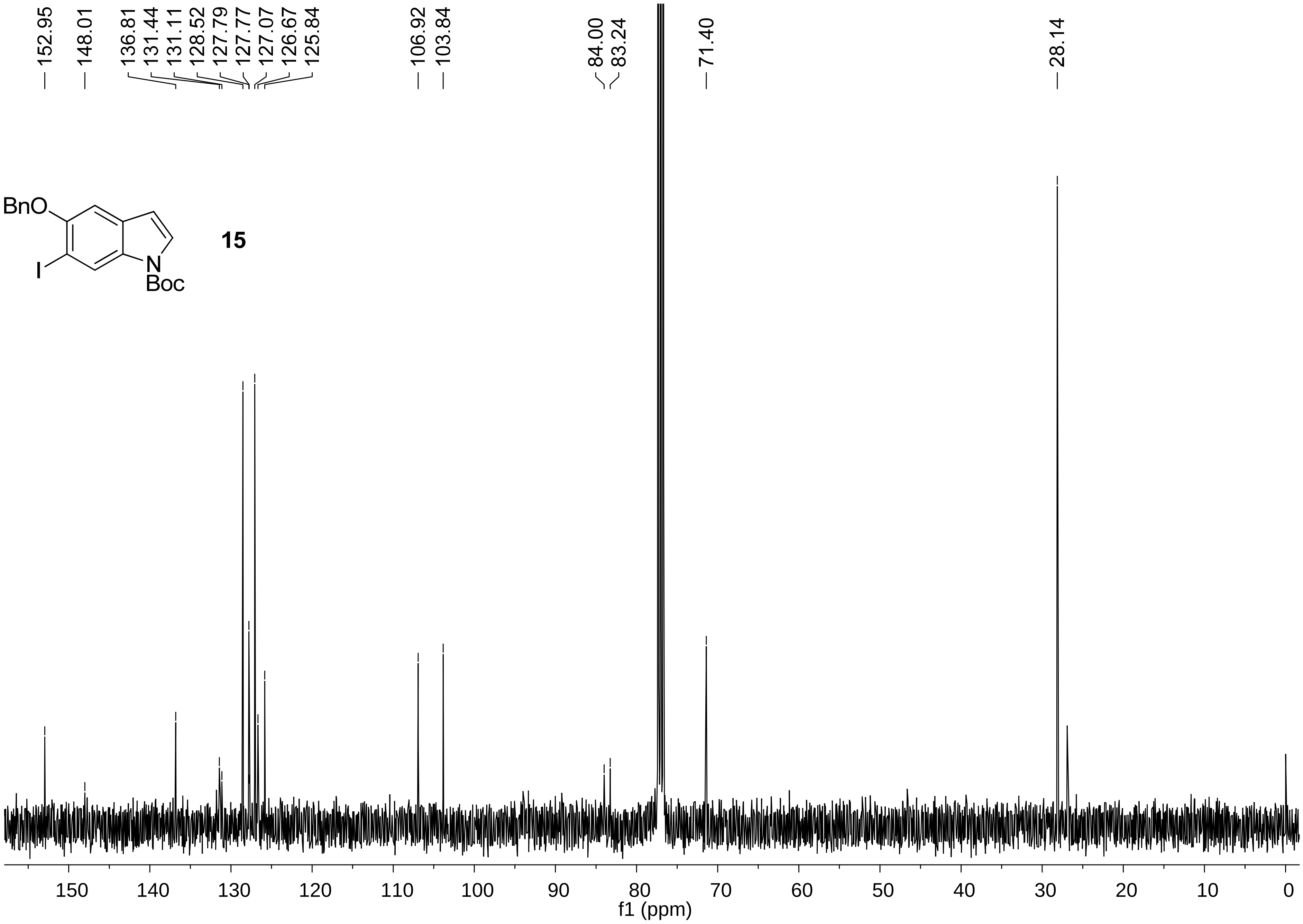

$^{1}H$ NMR ($CDCl_3$, 400 MHz)

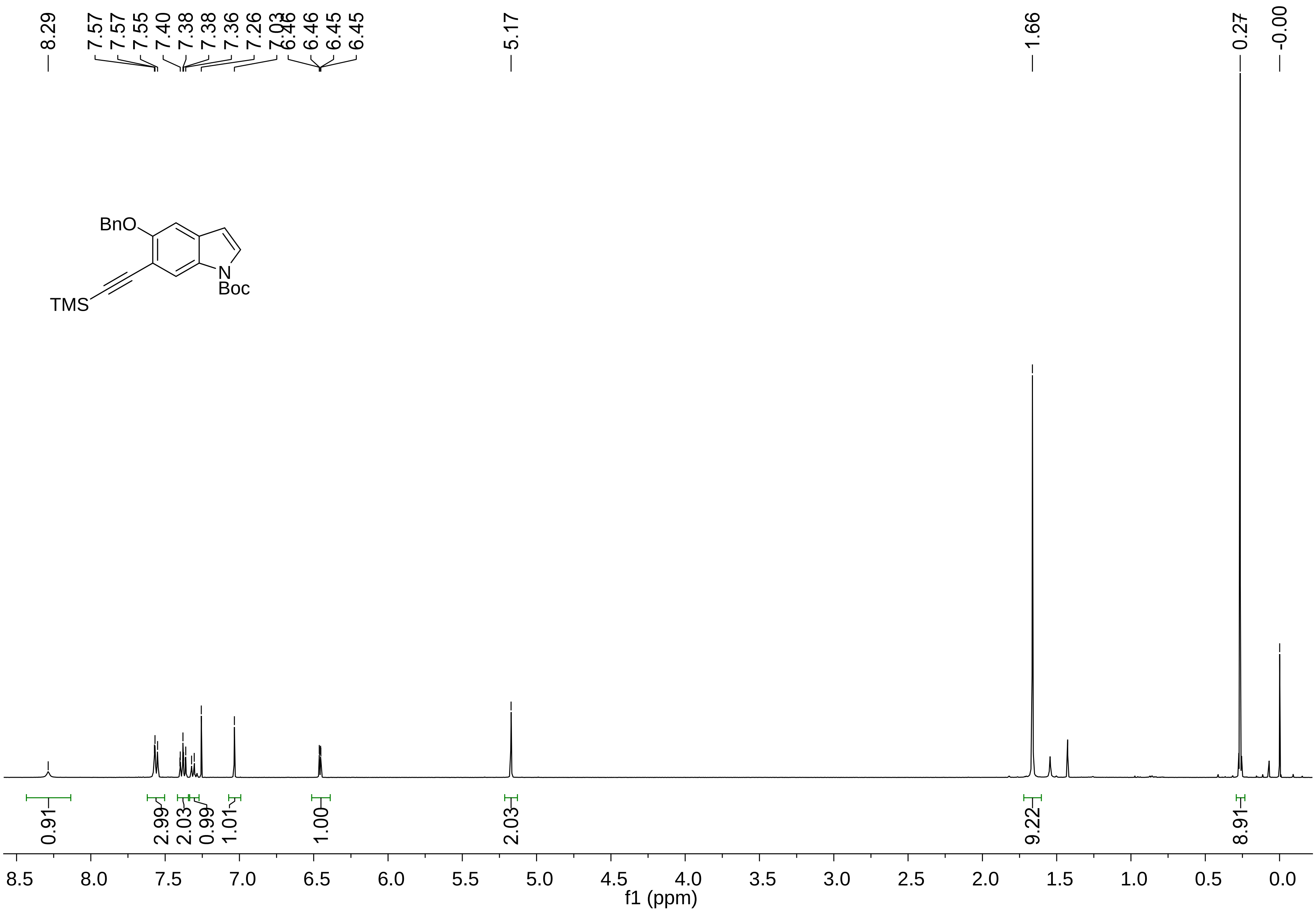

$^{13}C$ NMR ($CDCl_3$, 100 MHz)

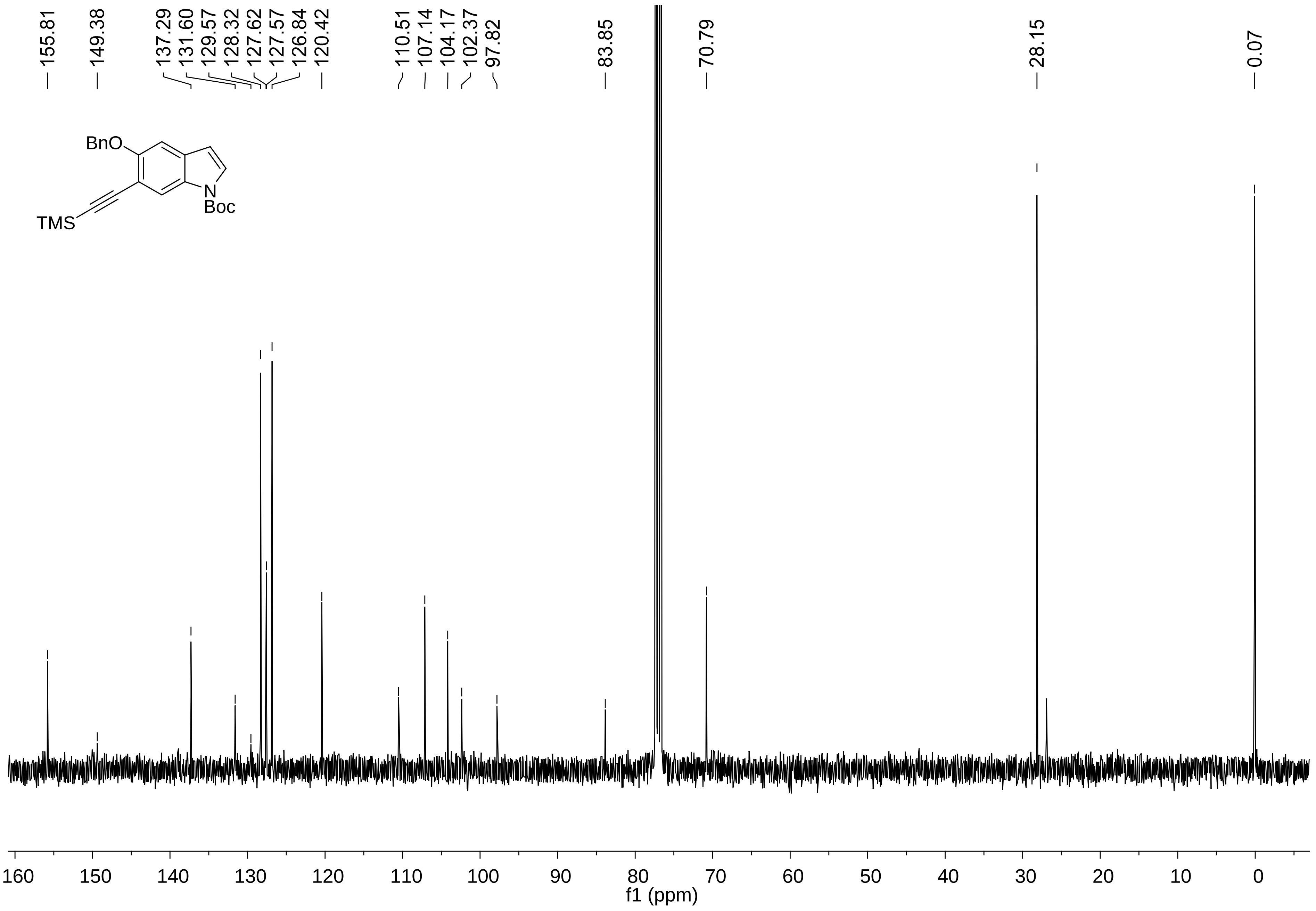

$^{1}$H NMR ($CDCl_3$, 400 MHz)

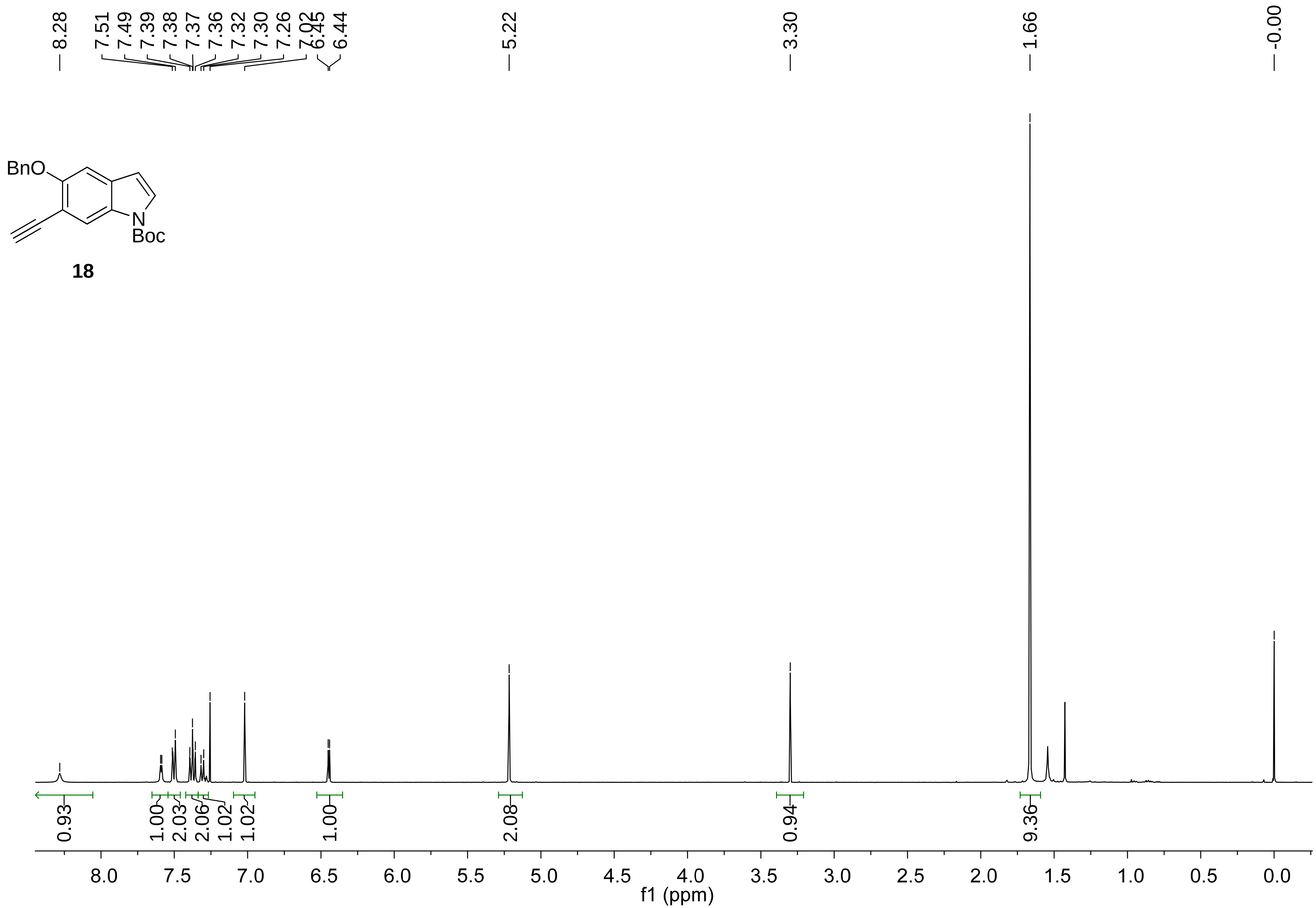

$^{13}C$ NMR ($CDCl_3$, 100 MHz)

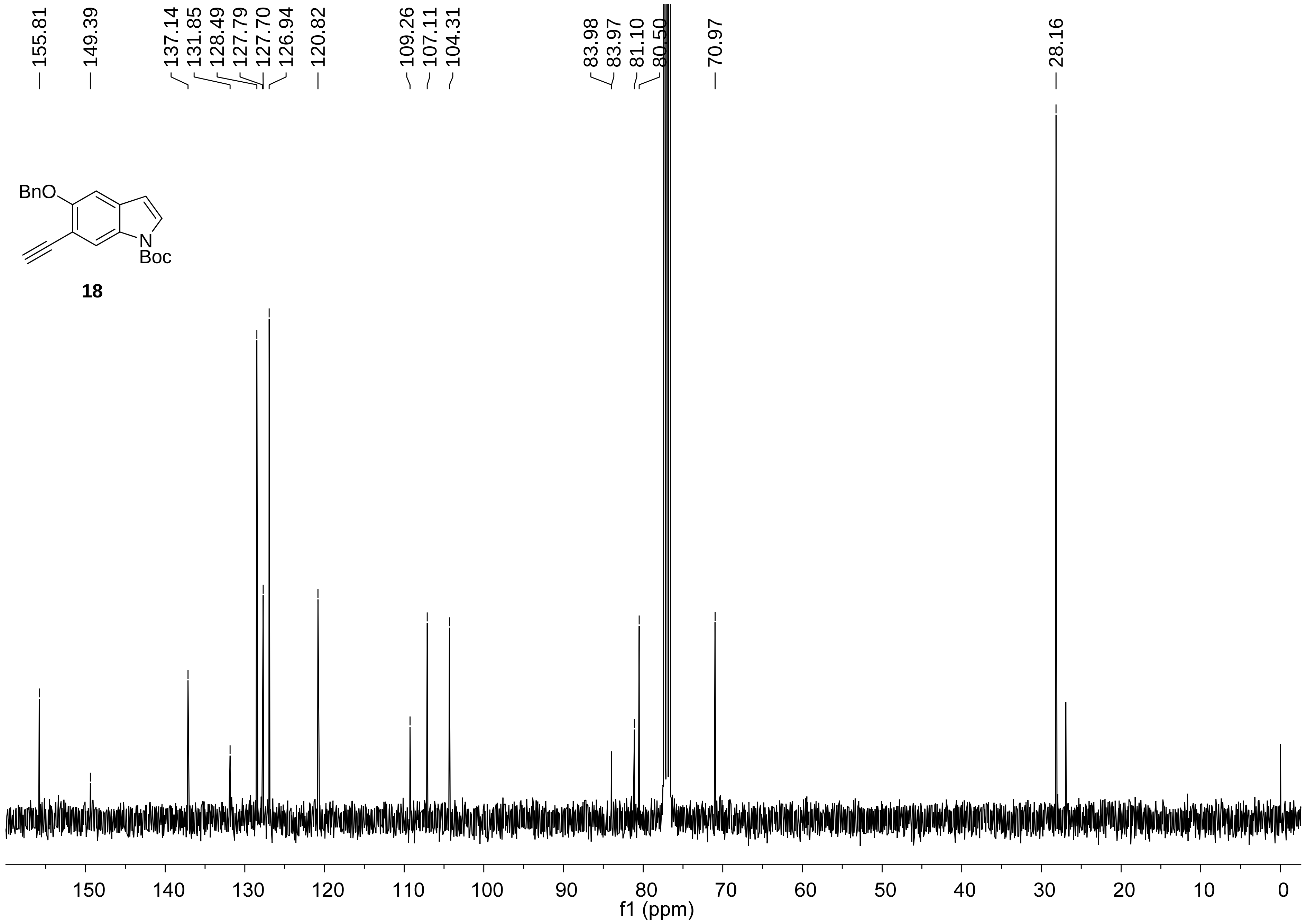


**18**

$^{1}$H NMR (CDCl$_3$, 600 MHz)

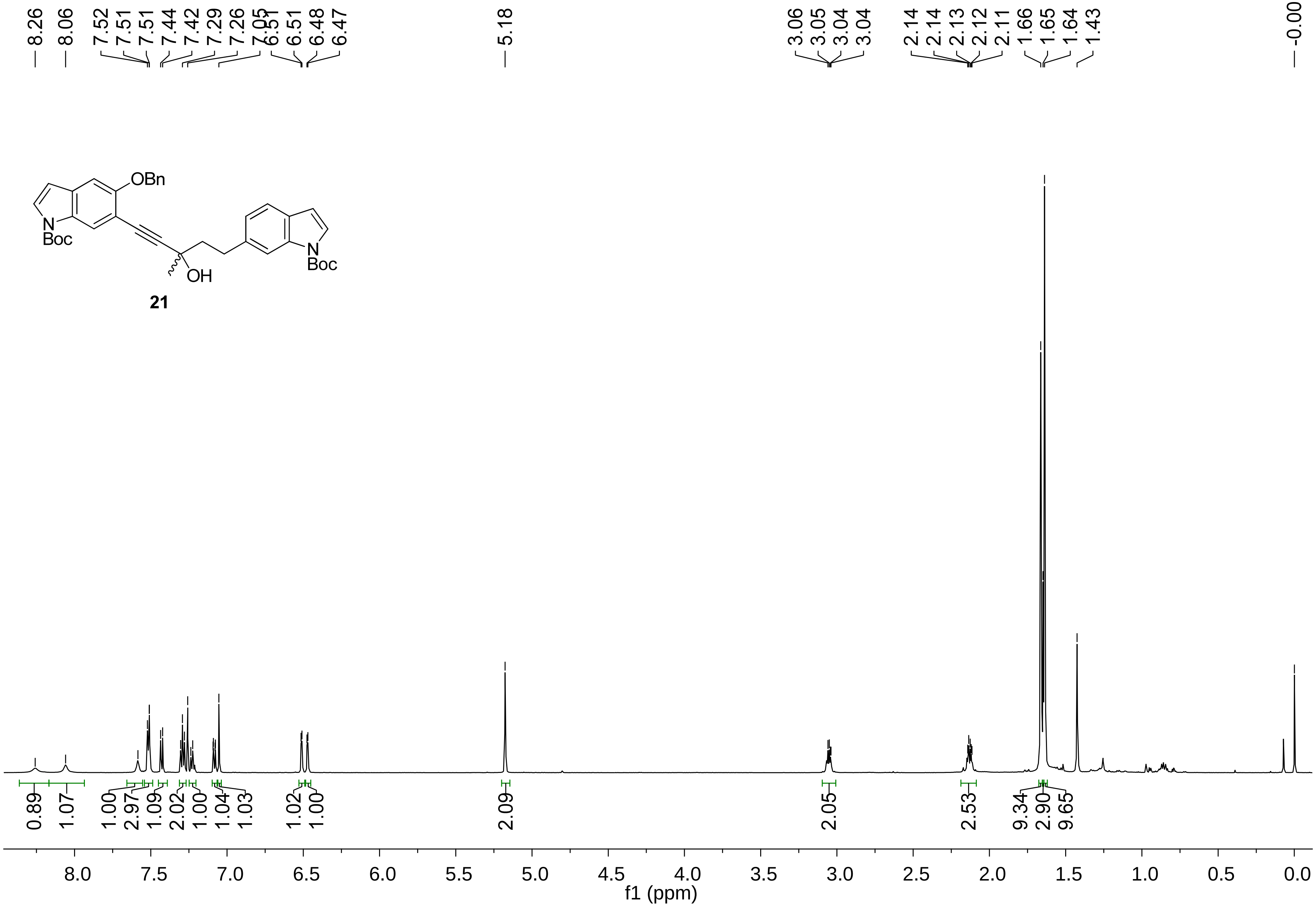

$^{13}C$ NMR ($CDCl_3$, 150 MHz)

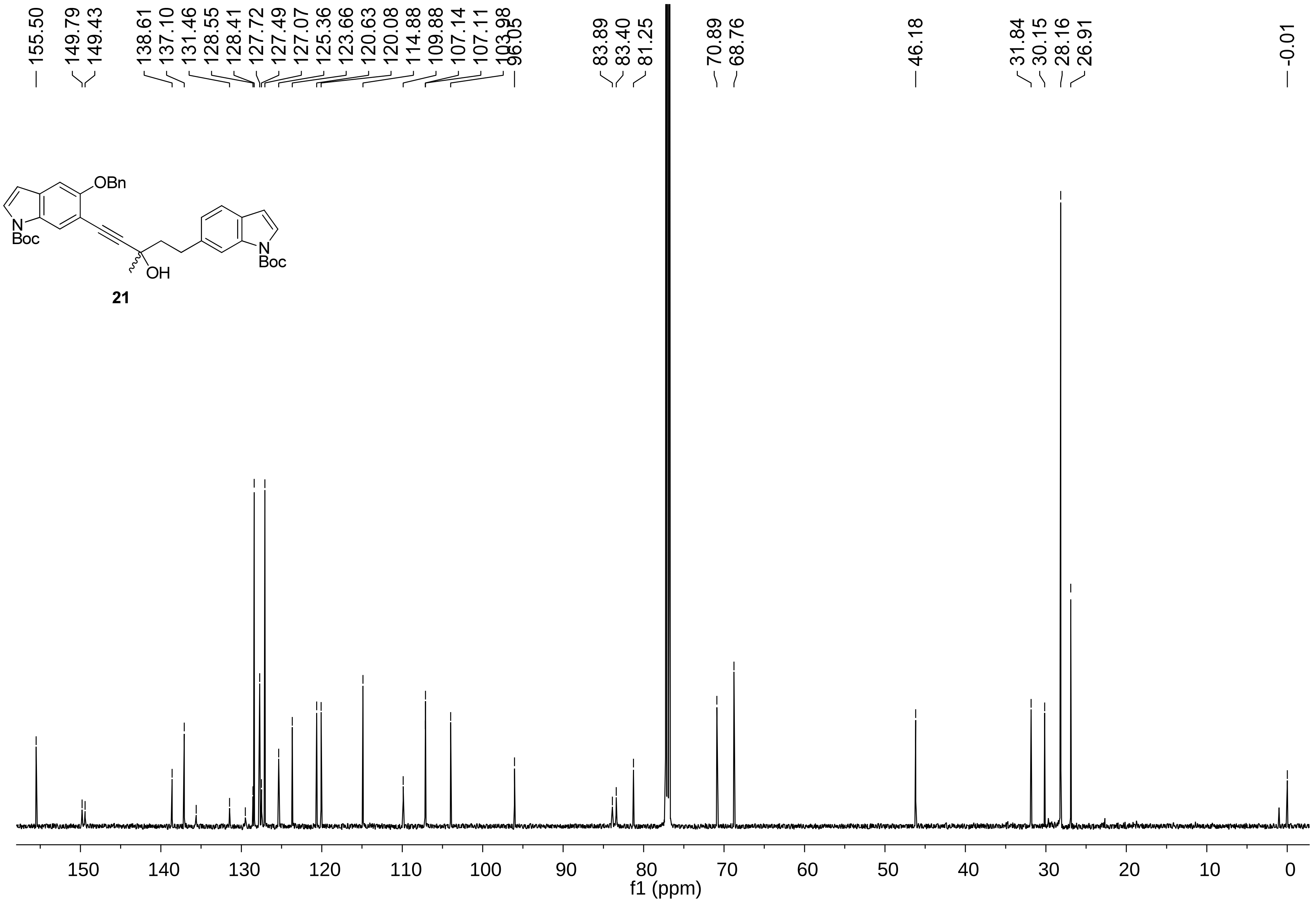

$^{1}H$ NMR ($CDCl_3$, 600 MHz)

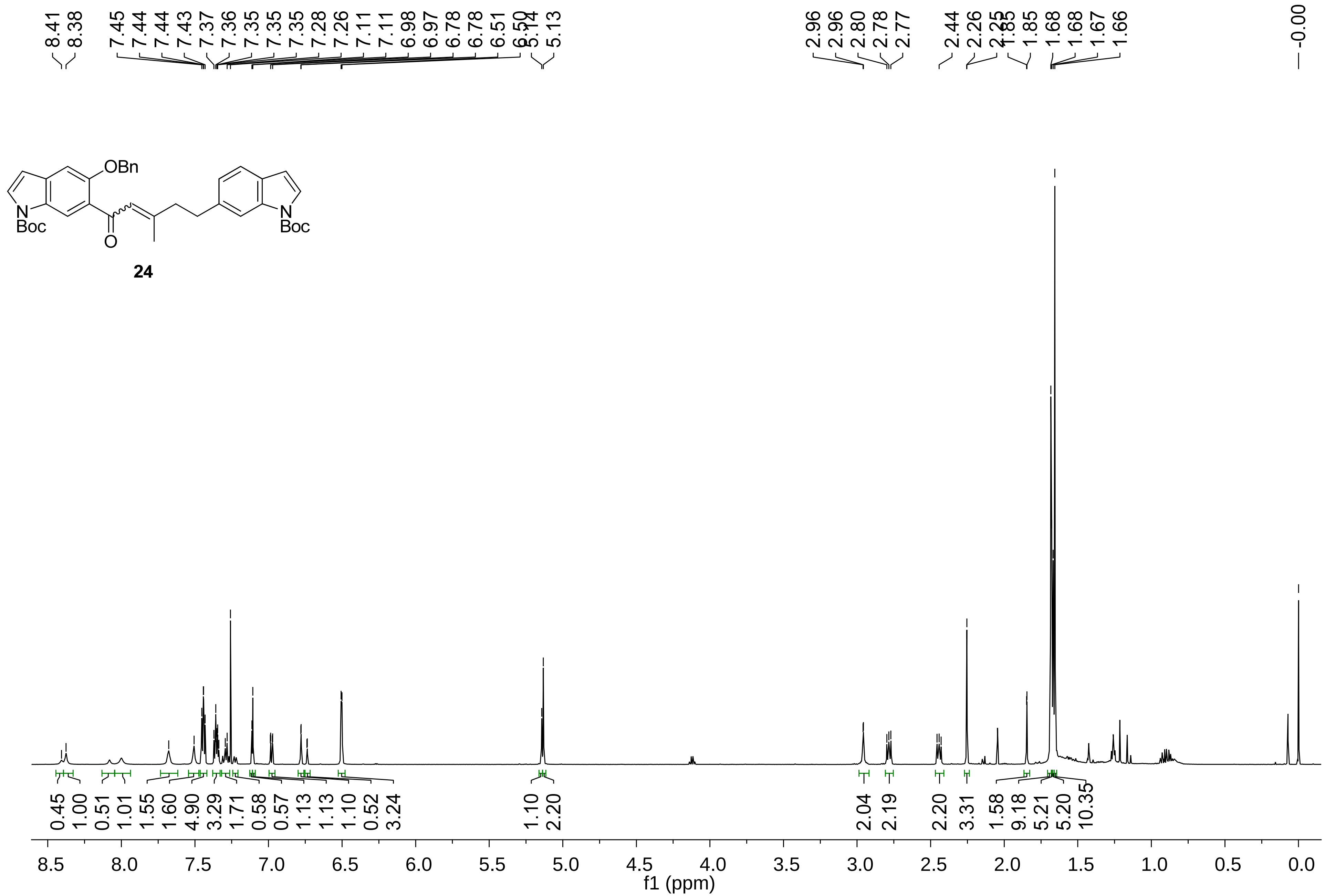


**24**

$^{13}C$ NMR ($CDCl_3$, 150 MHz)

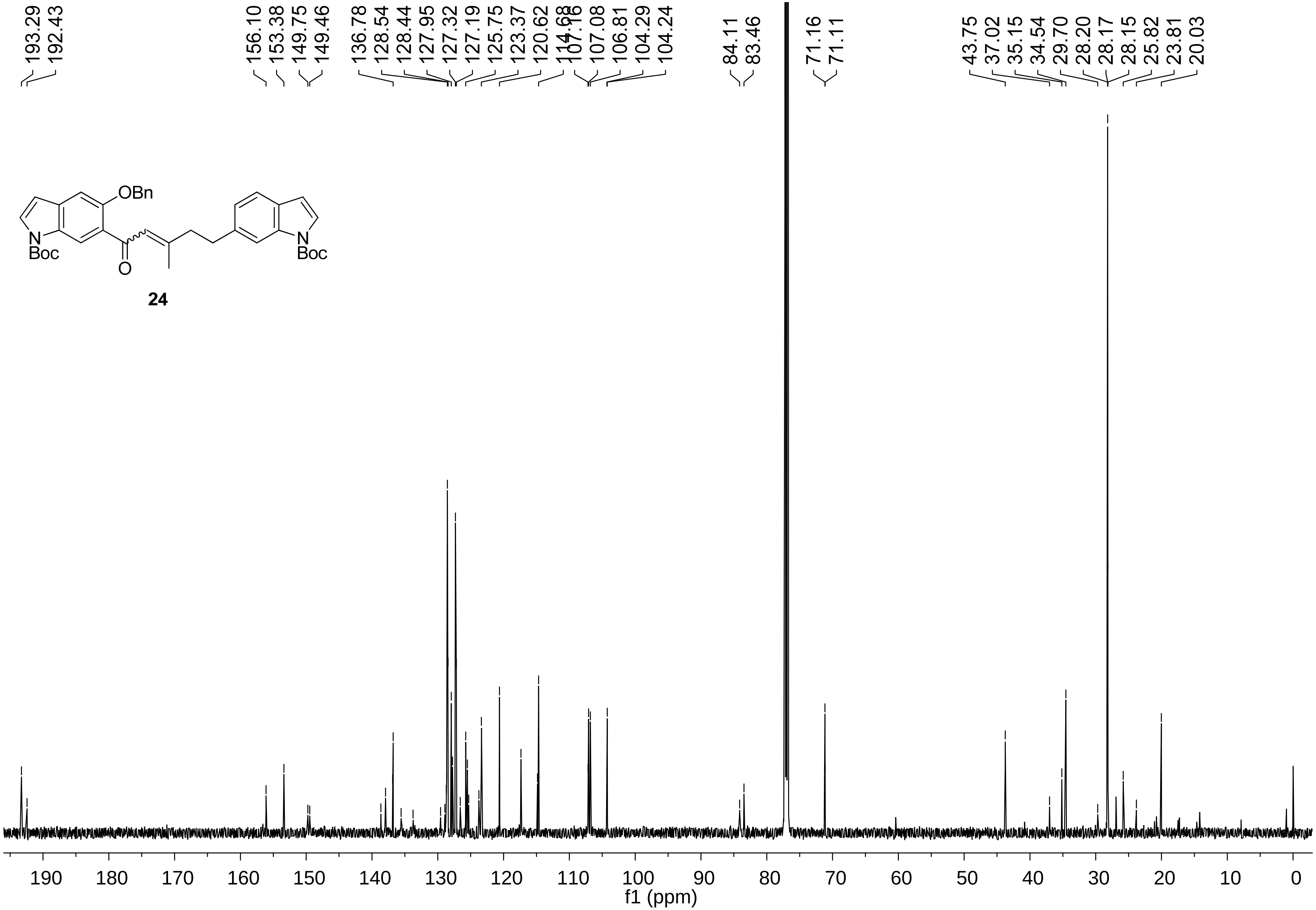

$^{1}$H NMR (CDCl$_3$, 400 MHz)

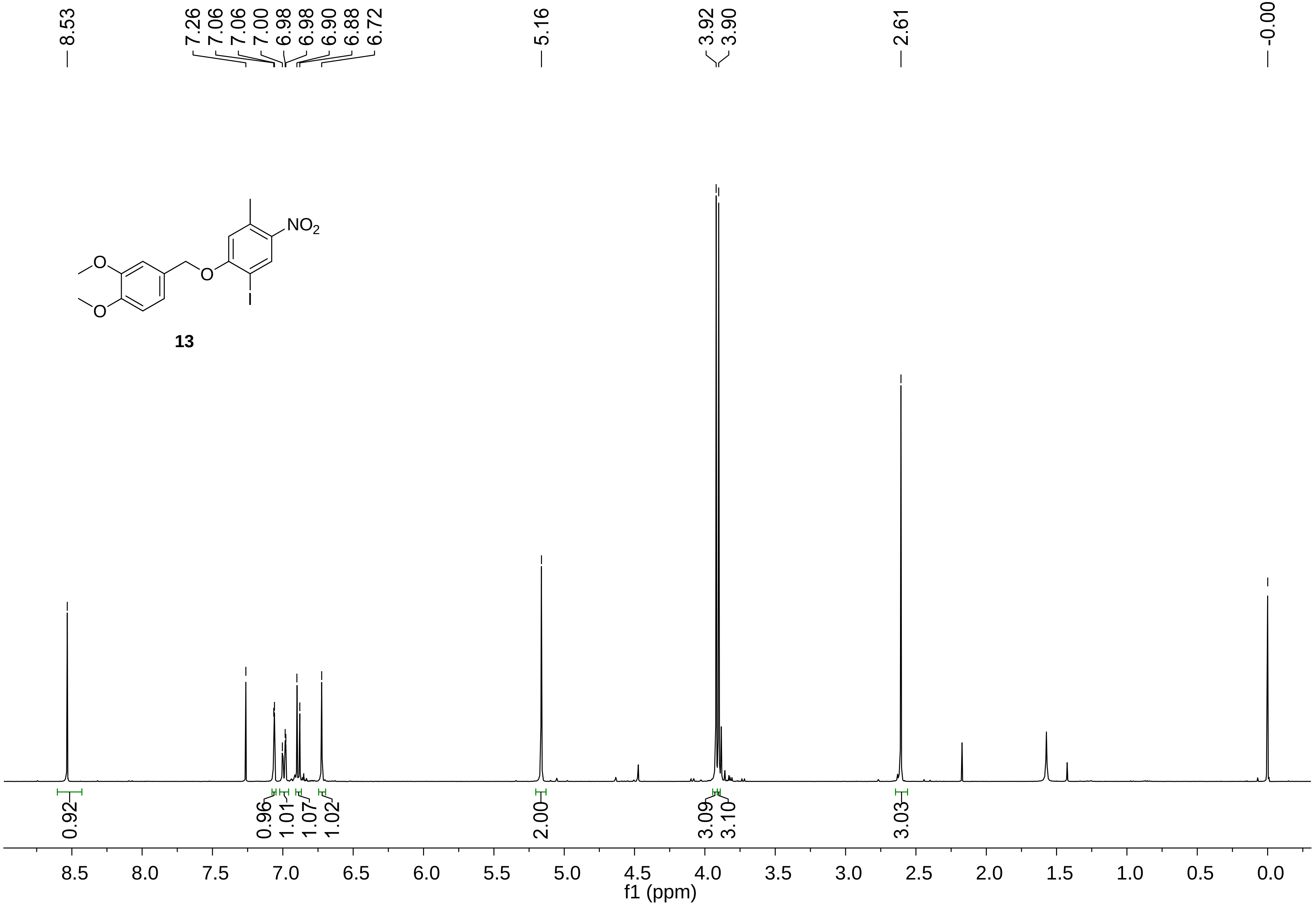

$^{13}C$ NMR ($CDCl_3$, 100 MHz)

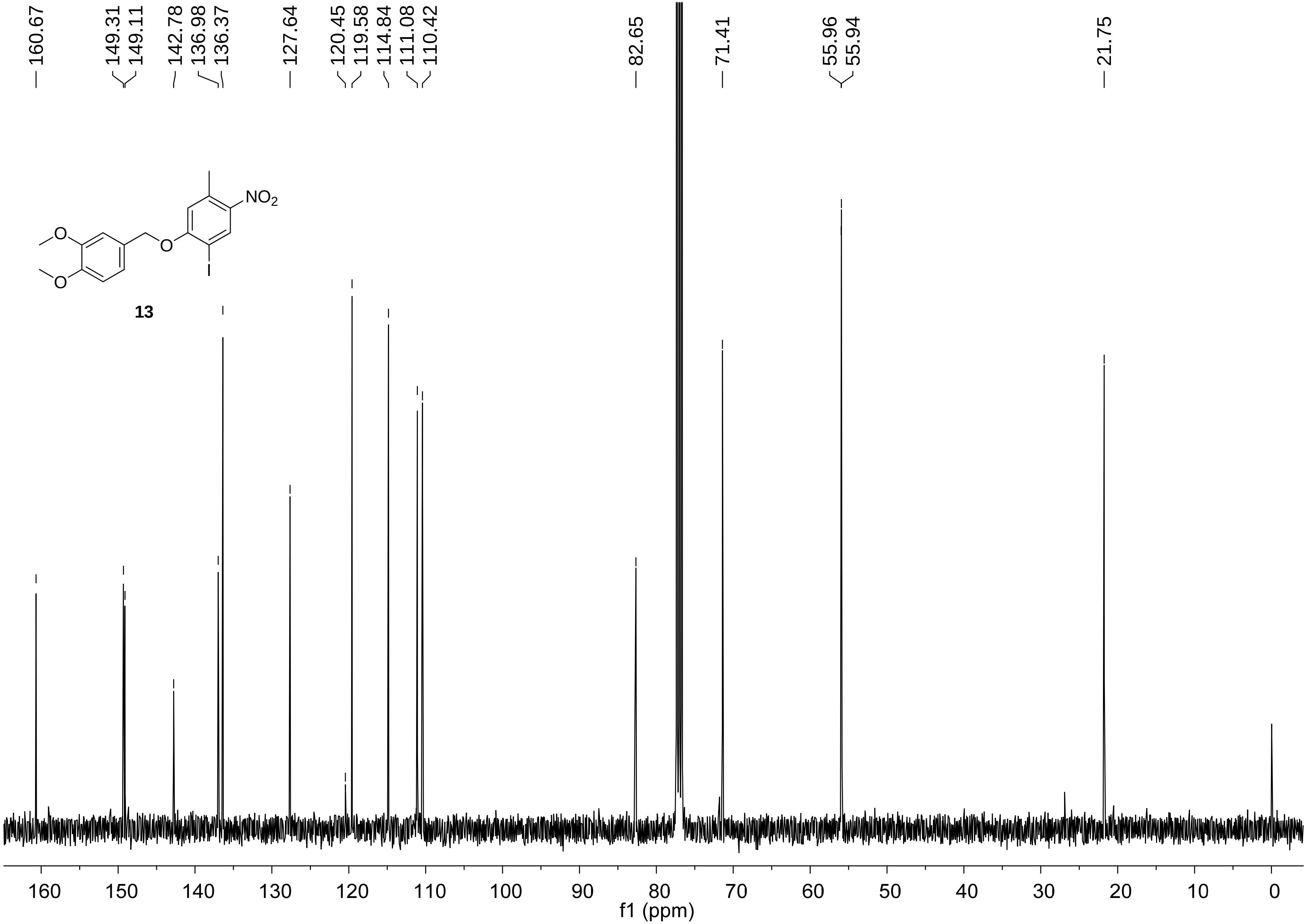

$^{1}$H NMR ($CDCl_3$, 400 MHz)

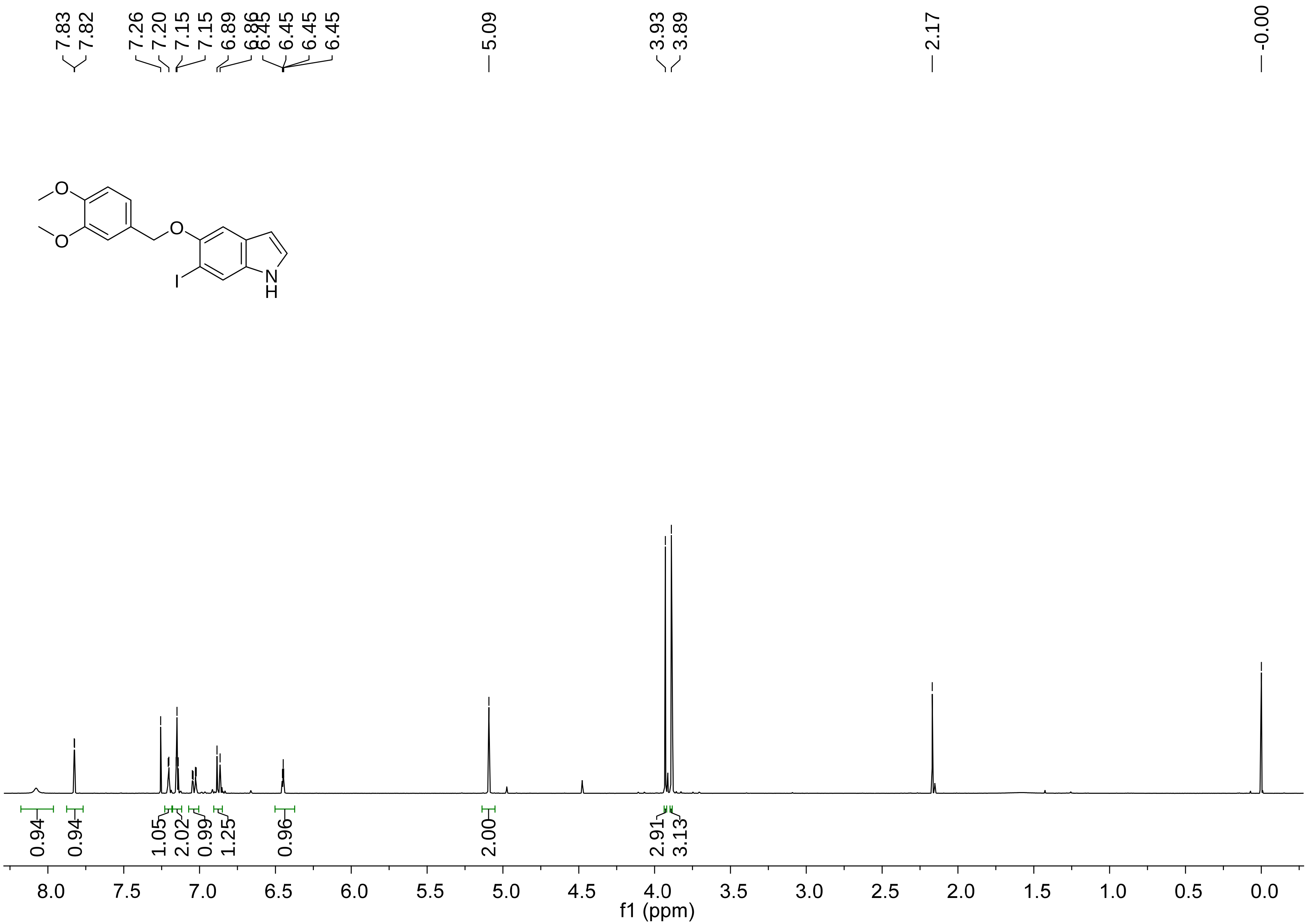

$^{13}C$ NMR ($CDCl_3$, 100 MHz)

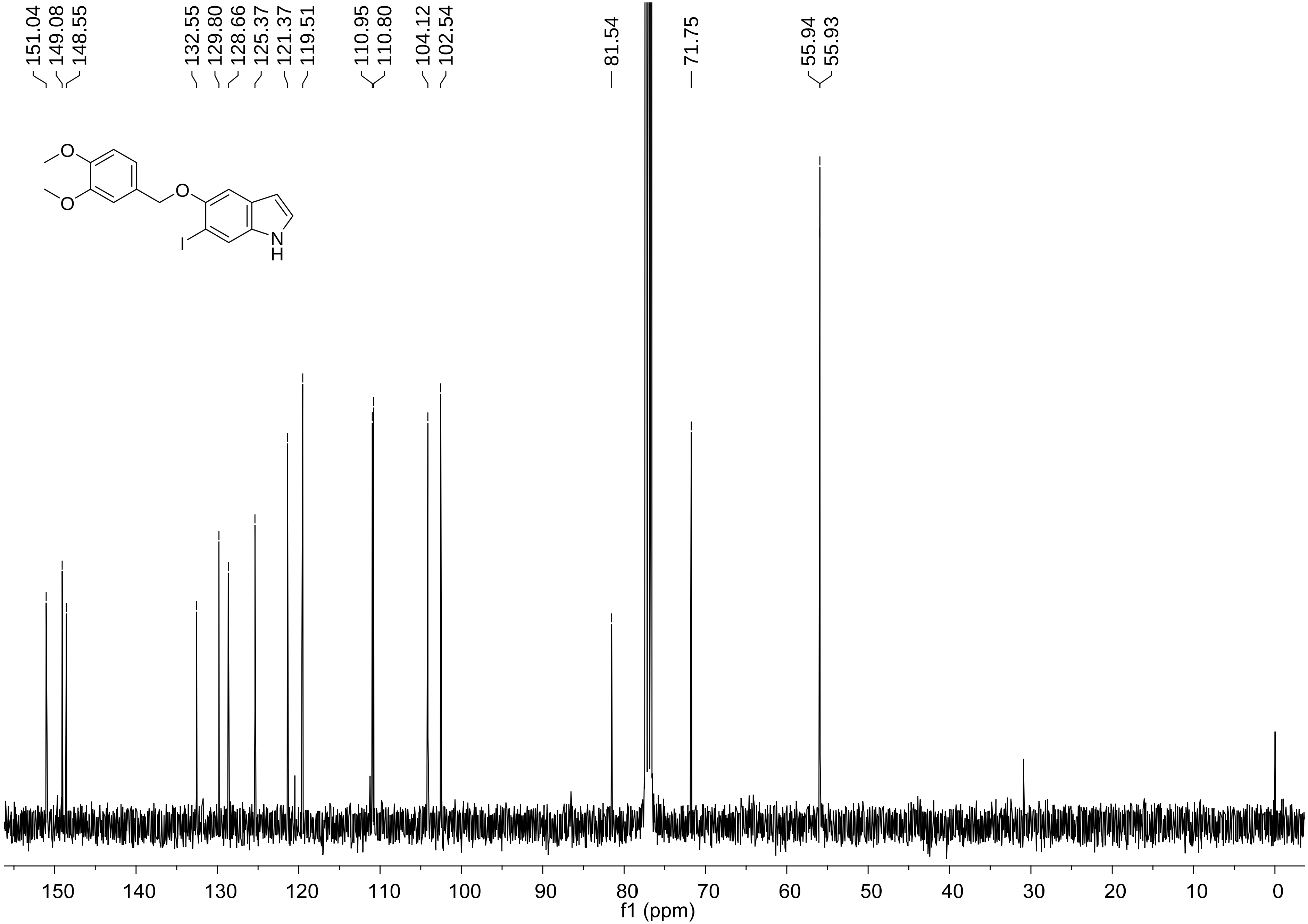

$^{1}H$ NMR ($CDCl_3$, 400 MHz)

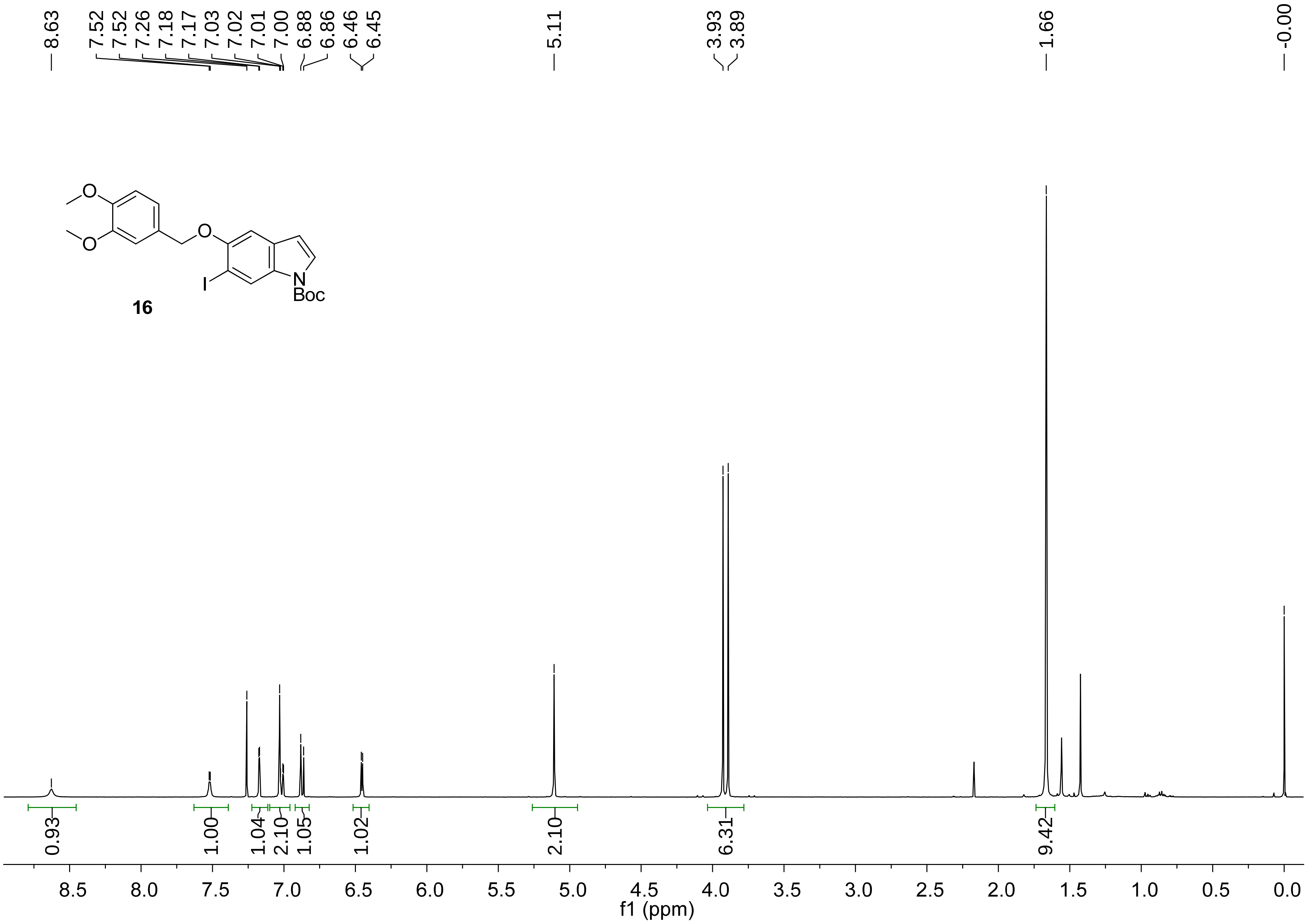

$^{13}C$ NMR ($CDCl_3$, 100 MHz)

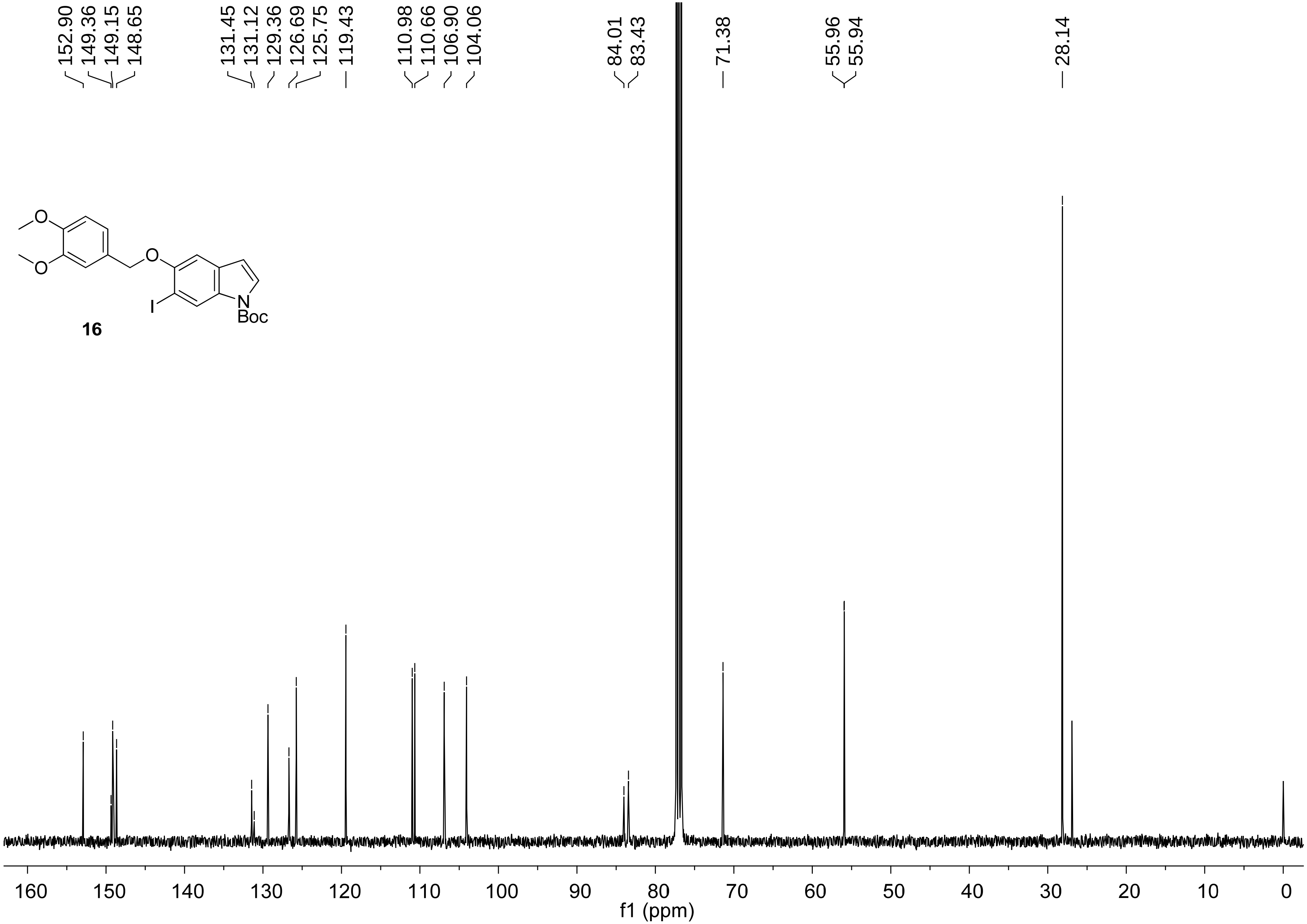

$^{1}H$ NMR ($CDCl_3$, 400 MHz)

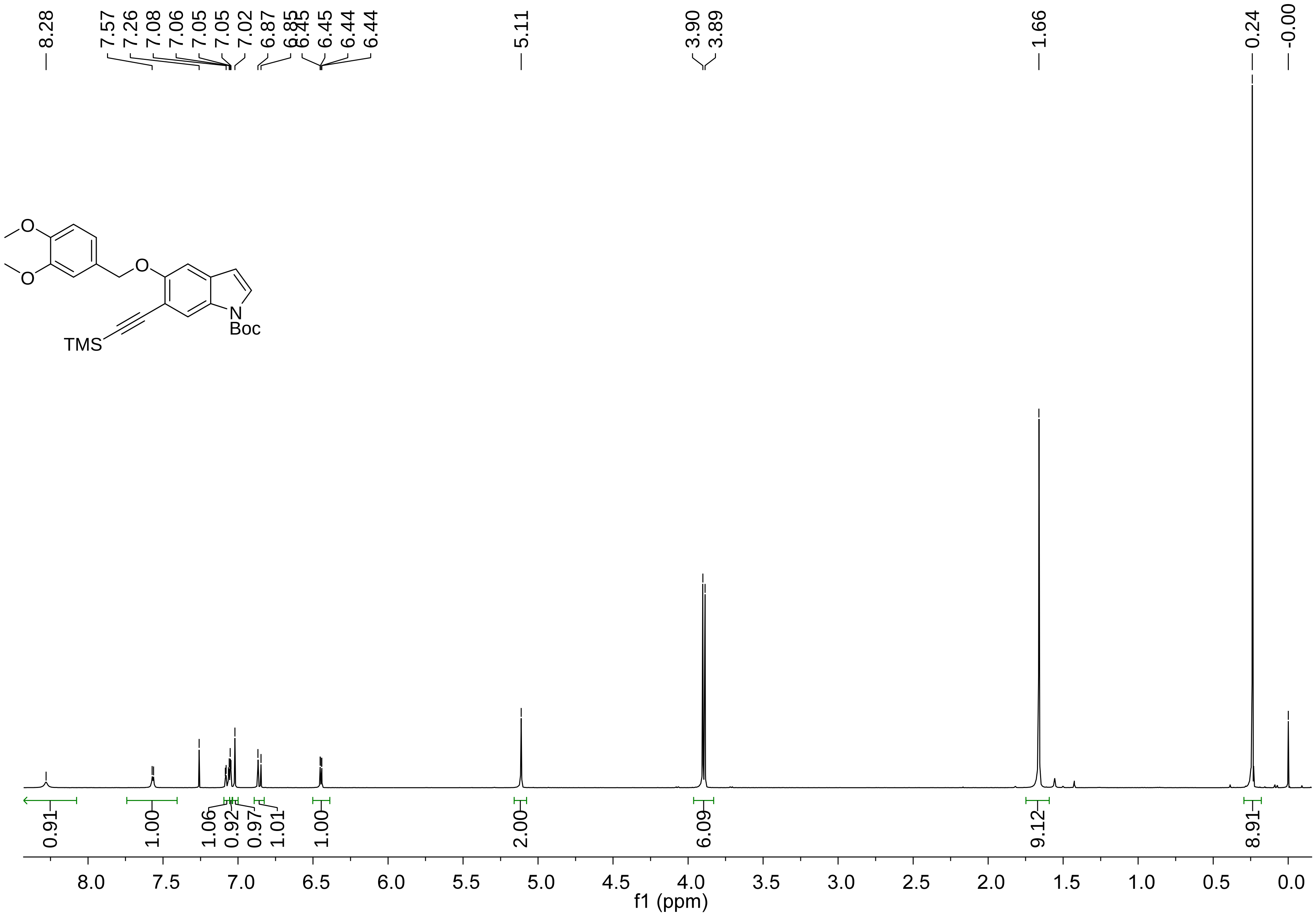

$^{13}C$ NMR ($CDCl_3$, 100 MHz)

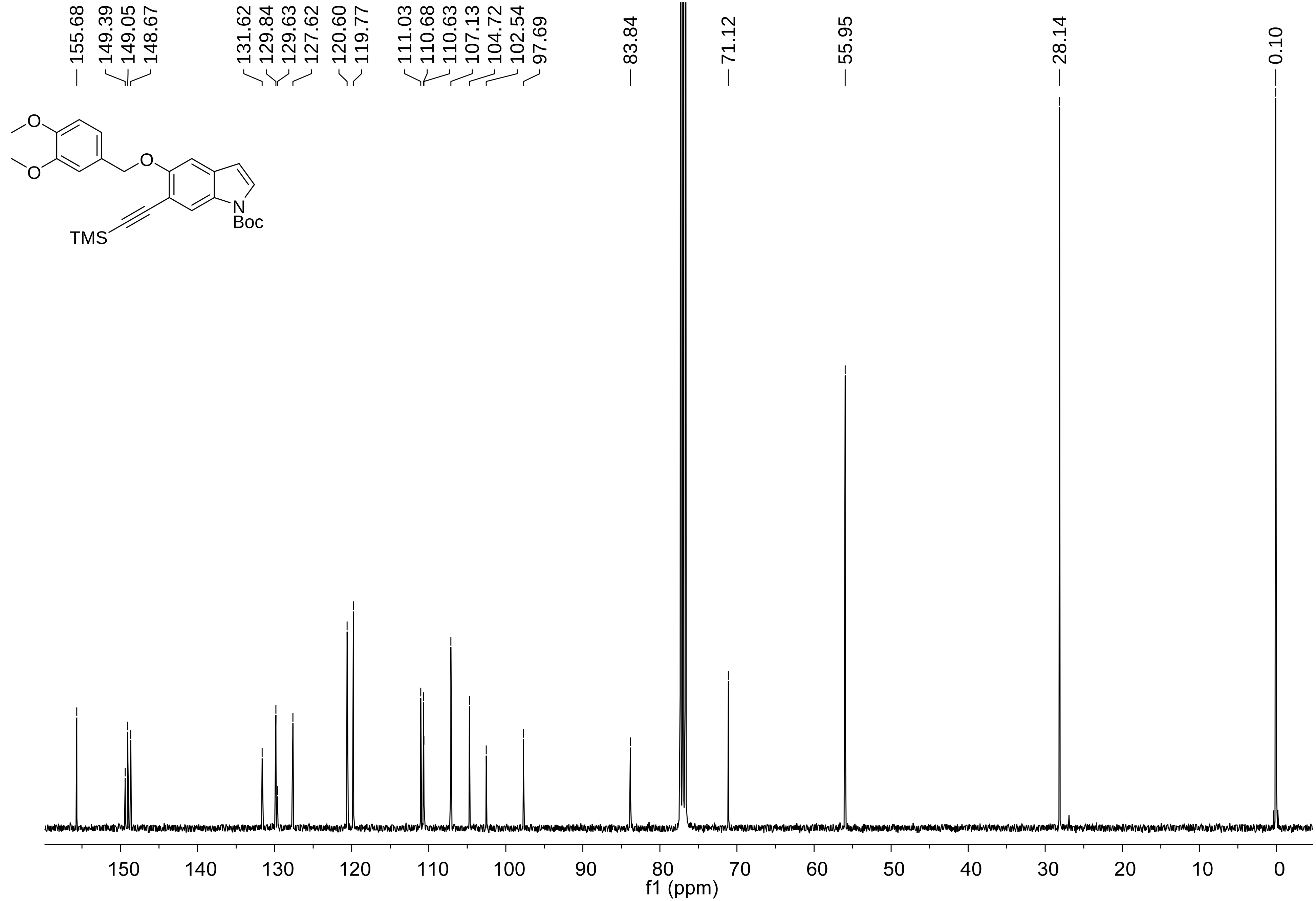

$^{1}H$ NMR ($CDCl_3$, 400 MHz)

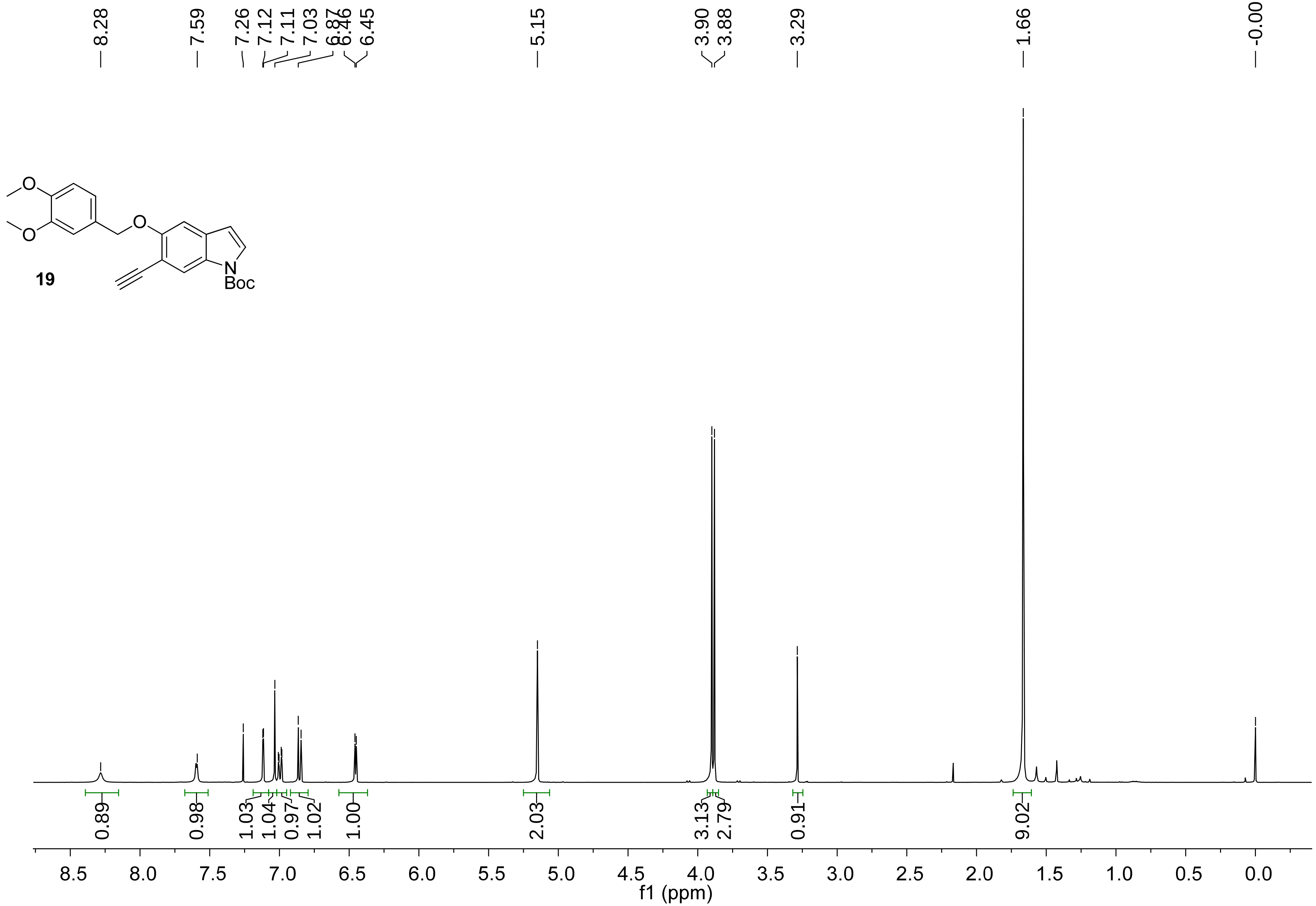

$^{13}C$ NMR ($CDCl_3$, 100 MHz)

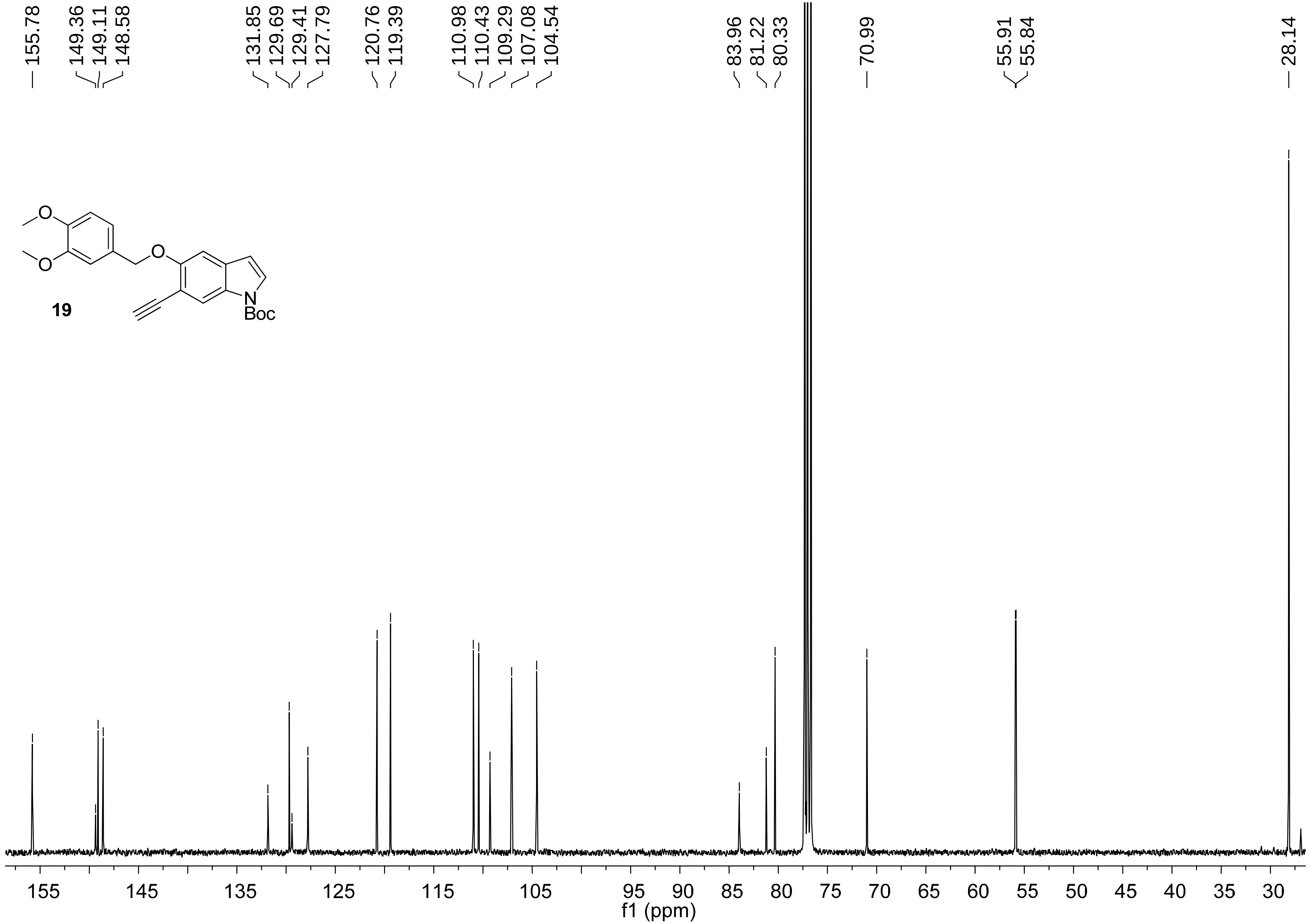

$^1H$ NMR ($CDCl_3$, 400 MHz)

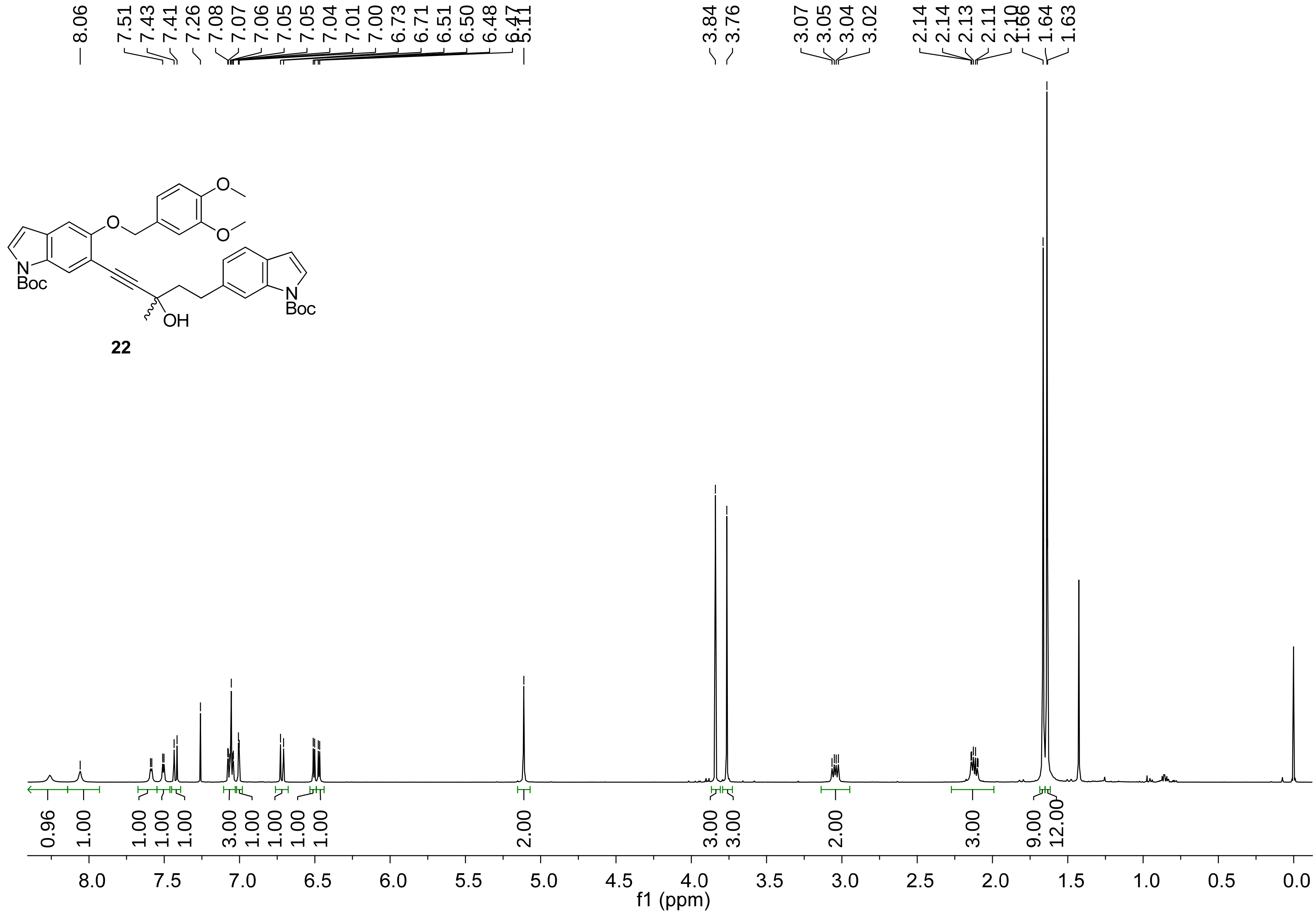


**22**

$^{13}C$ NMR ($CDCl_3$, 100 MHz)

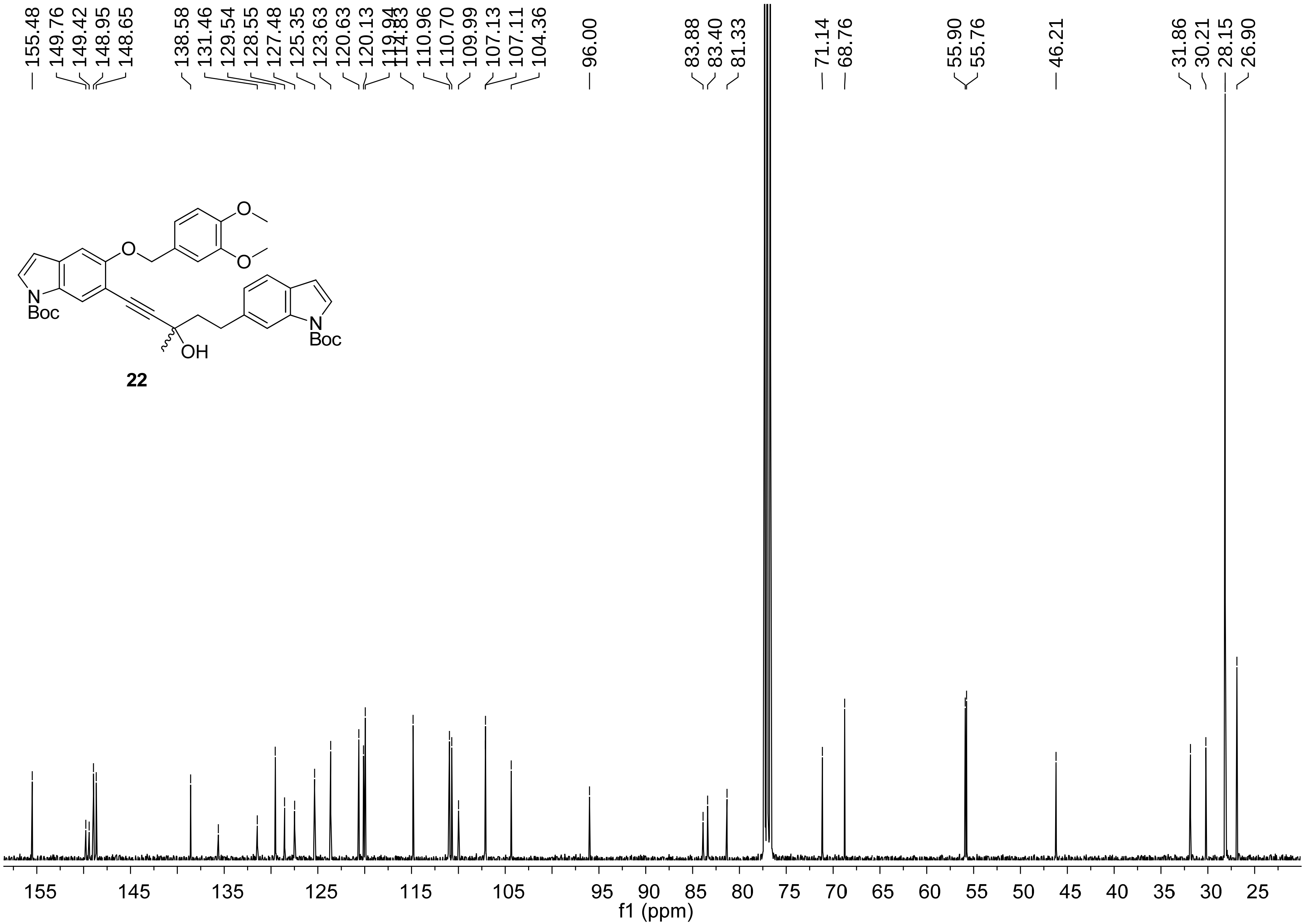


**22**

$^{1}H$ NMR ($CDCl_3$, 400 MHz)

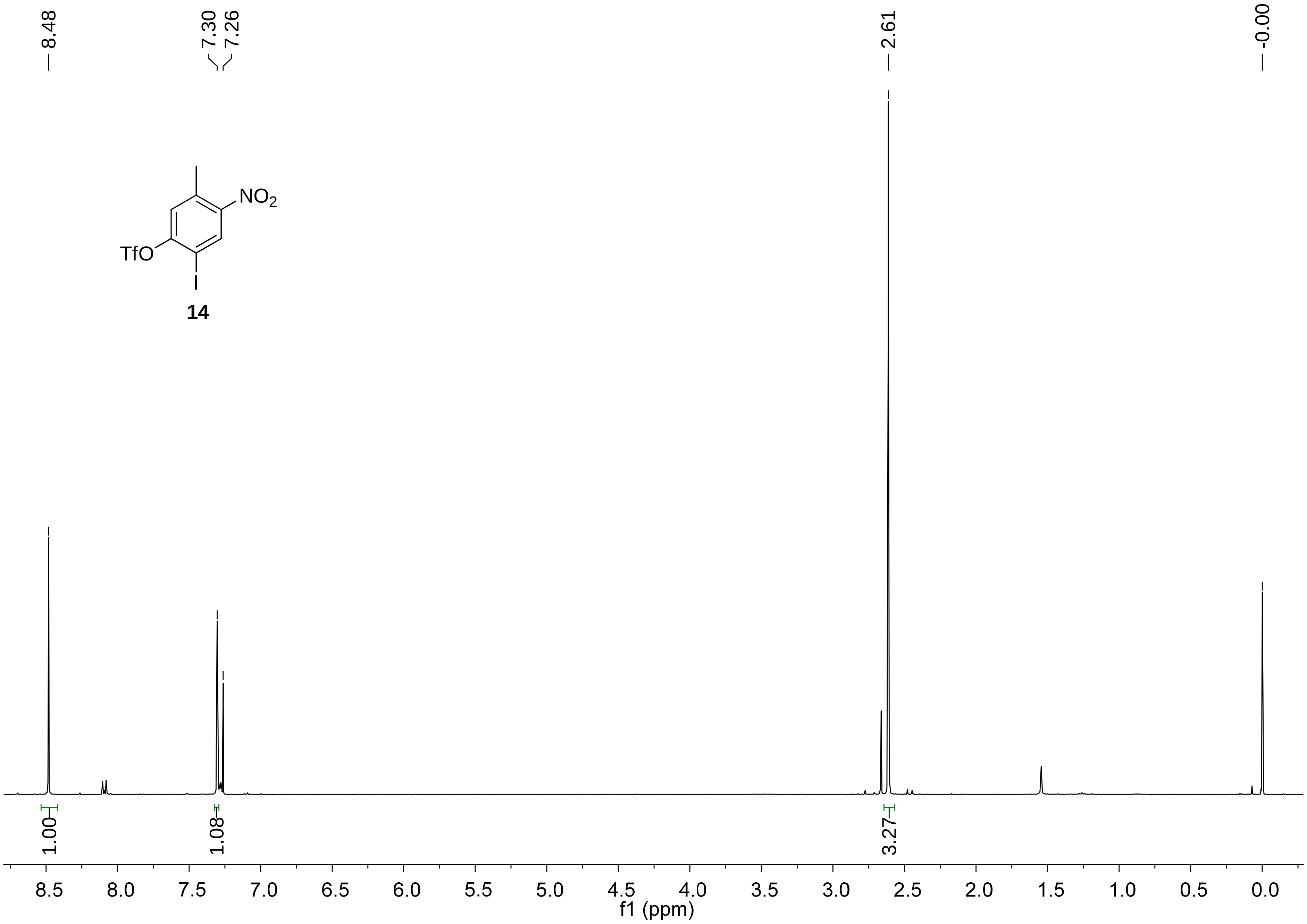

$^{13}C$ NMR ($CDCl_3$, 100 MHz)

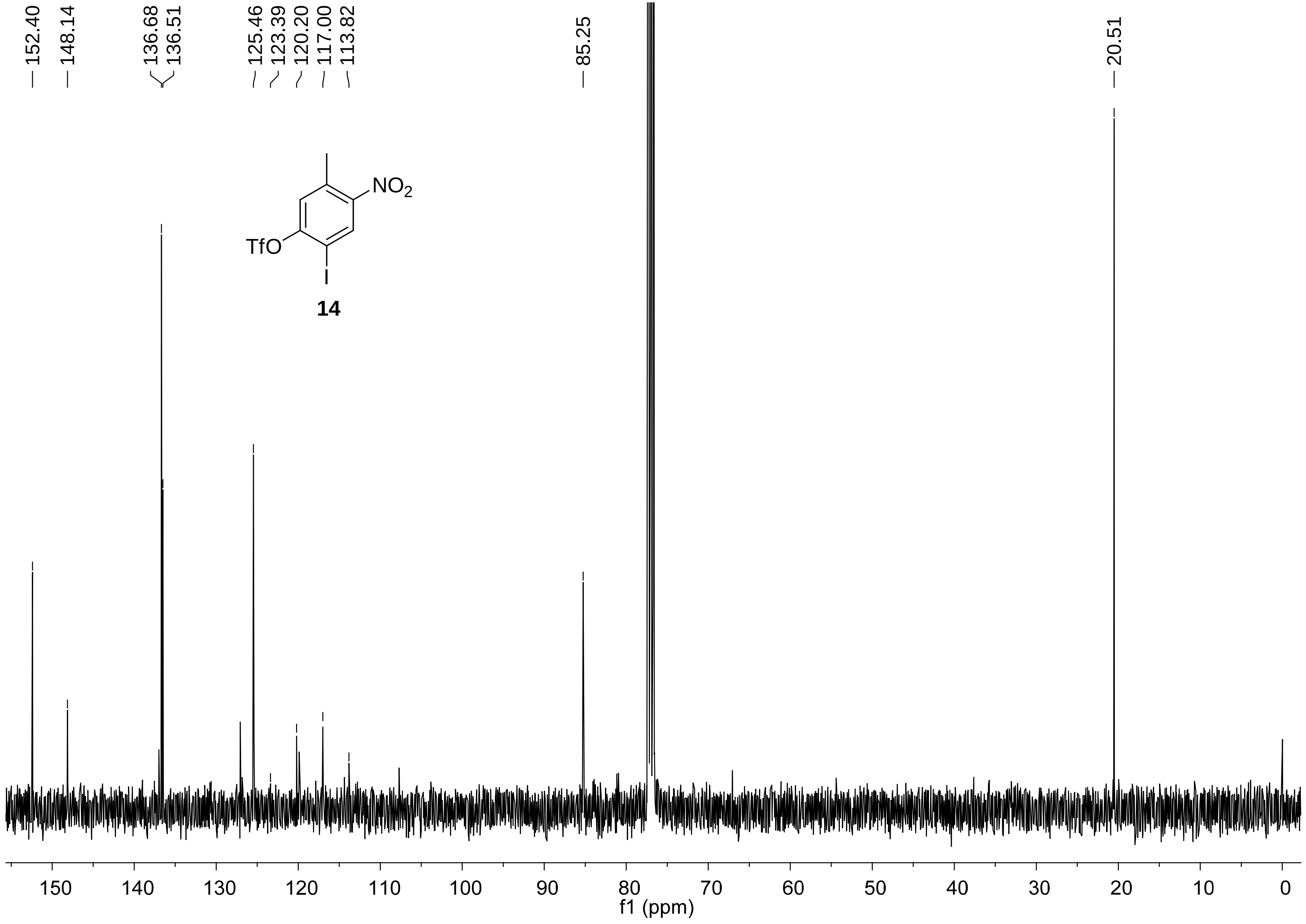

$^{1}H$ NMR ($CDCl_3$, 400 MHz)

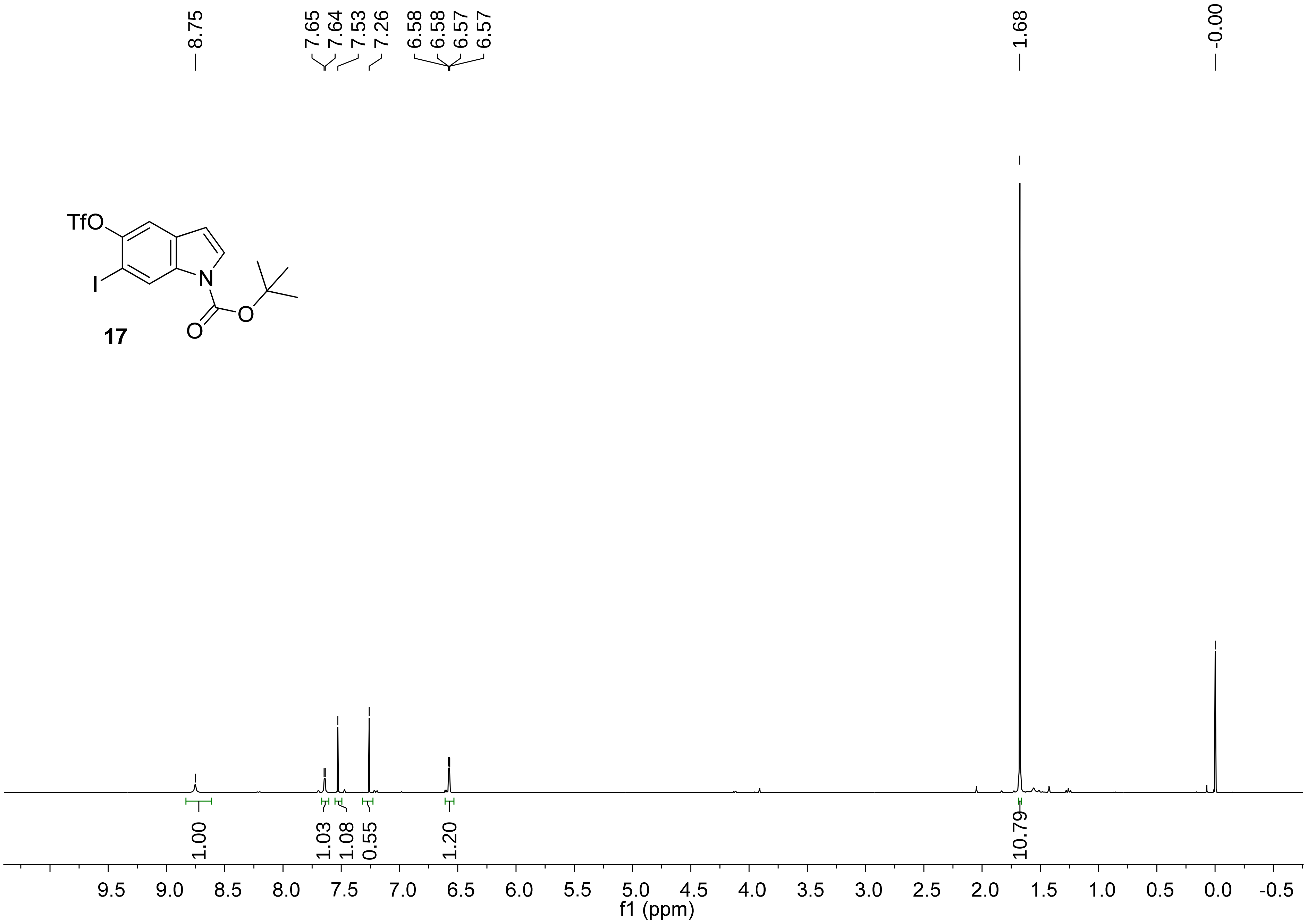

$^{13}C$ NMR ($CDCl_3$, 100 MHz)

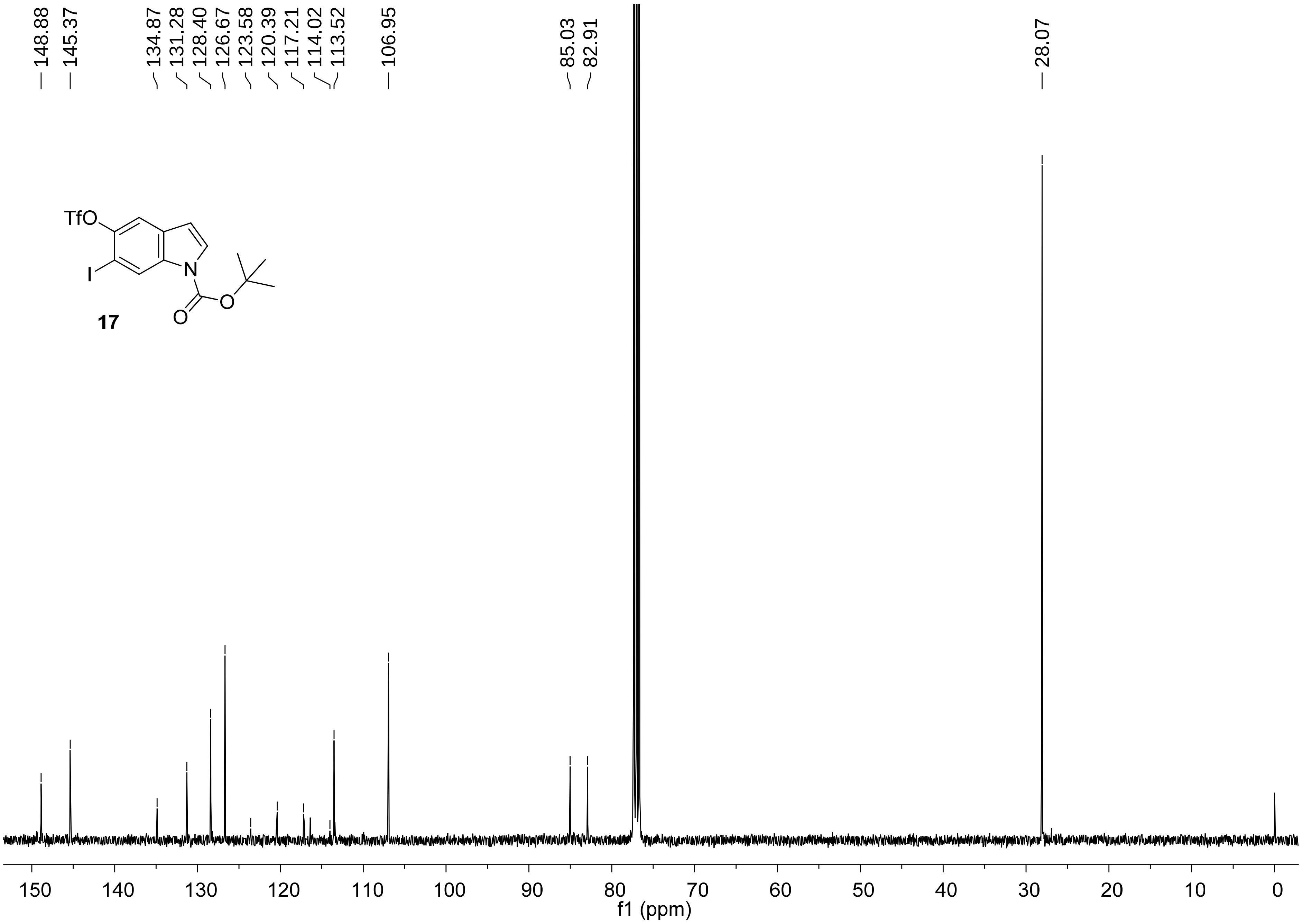

$^{19}F$ NMR ($CDCl_3$, 376 MHz)

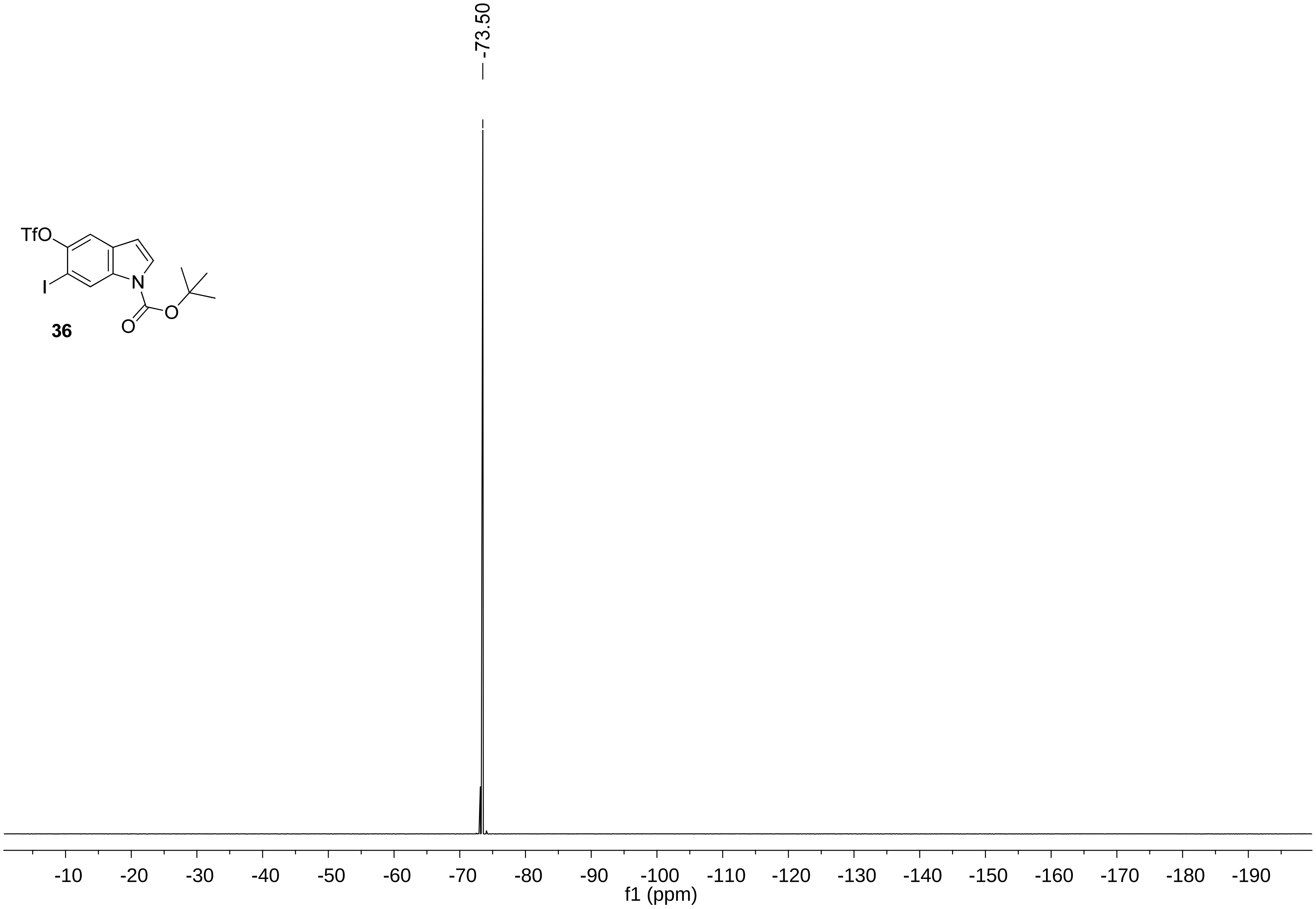

$^{1}H$ NMR ($CDCl_3$, 400 MHz)

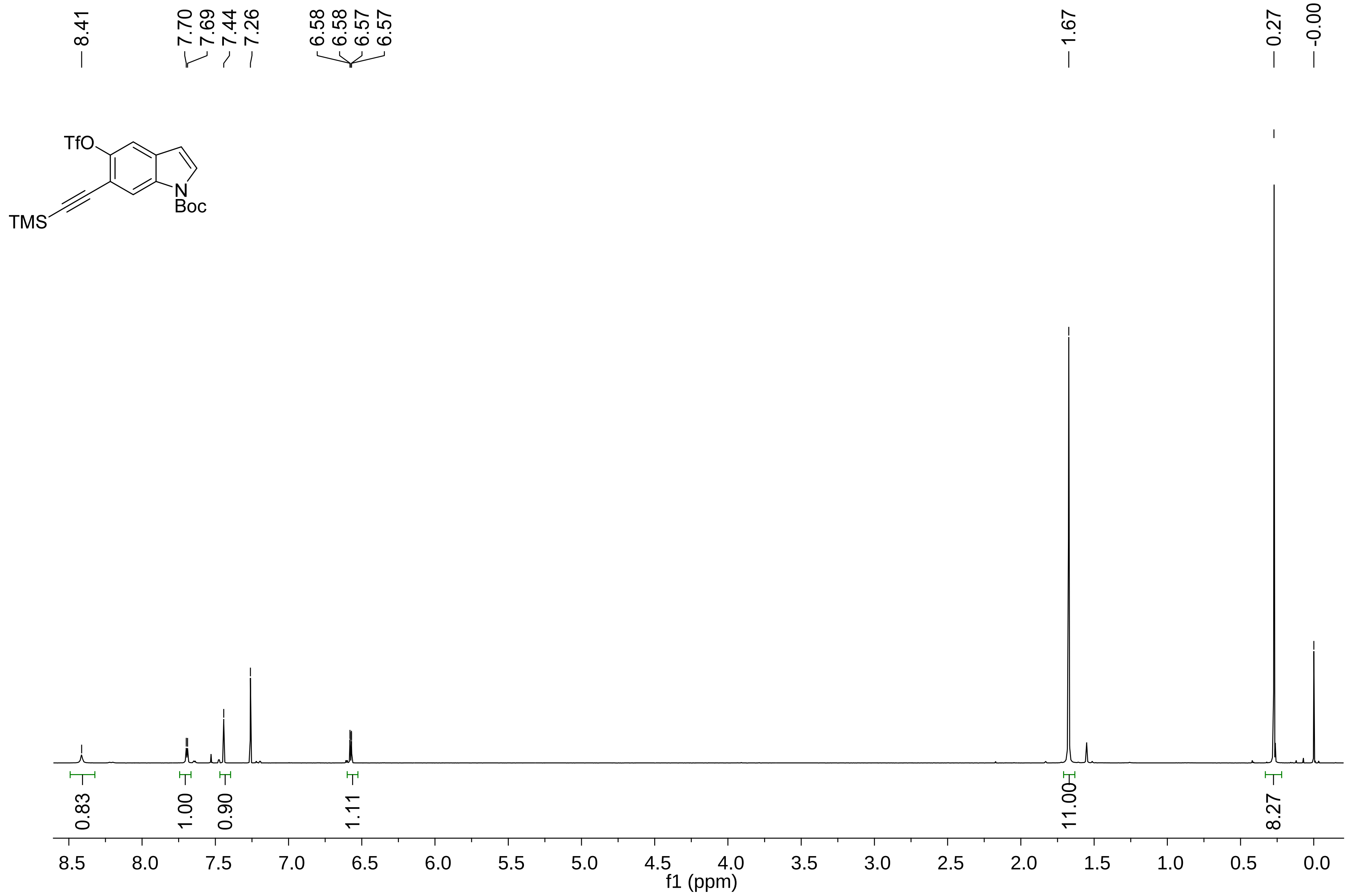

$^{13}C$ NMR ($CDCl_3$, 100 MHz)

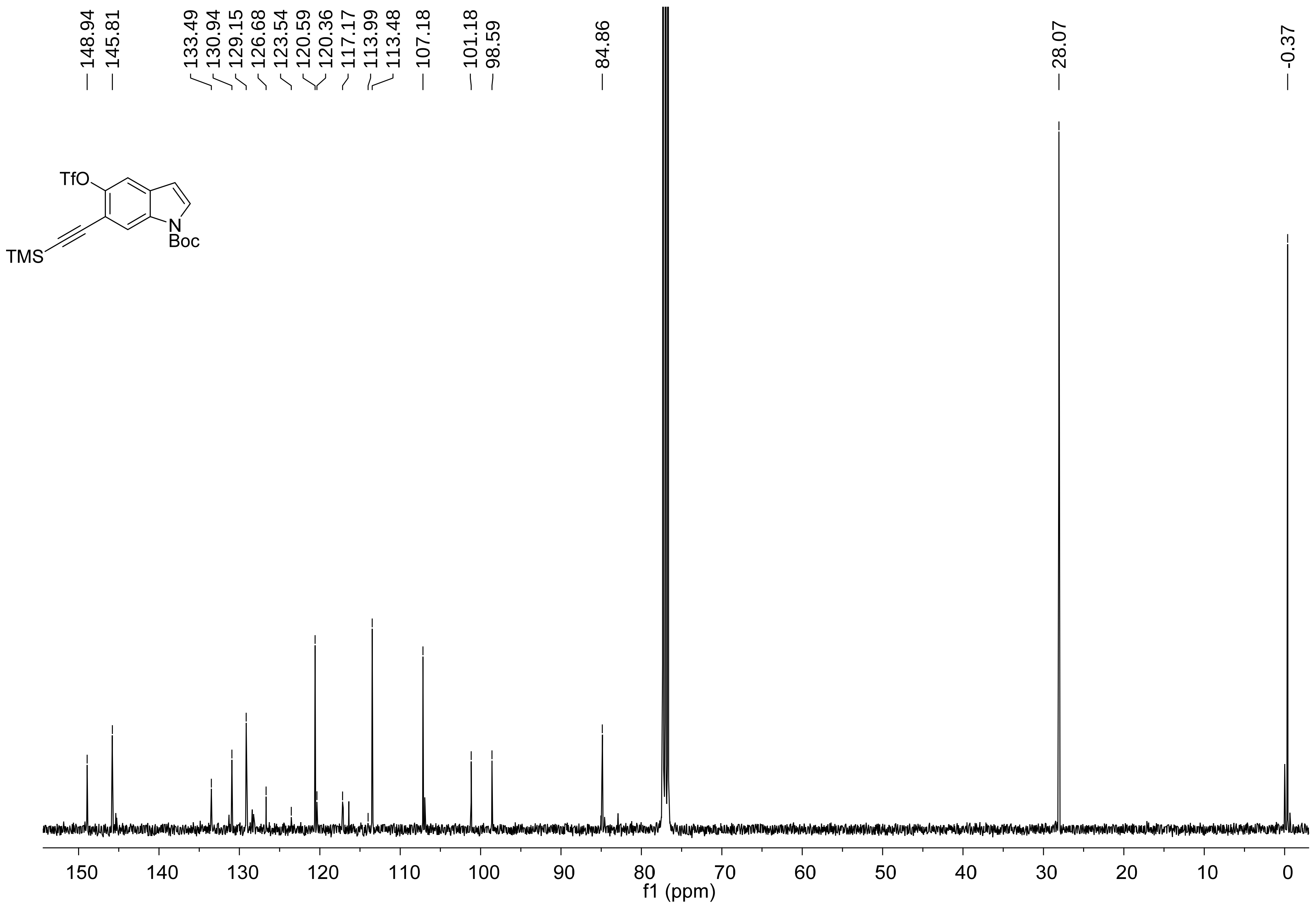

$^{19}F$ NMR ($CDCl_3$, 376 MHz)

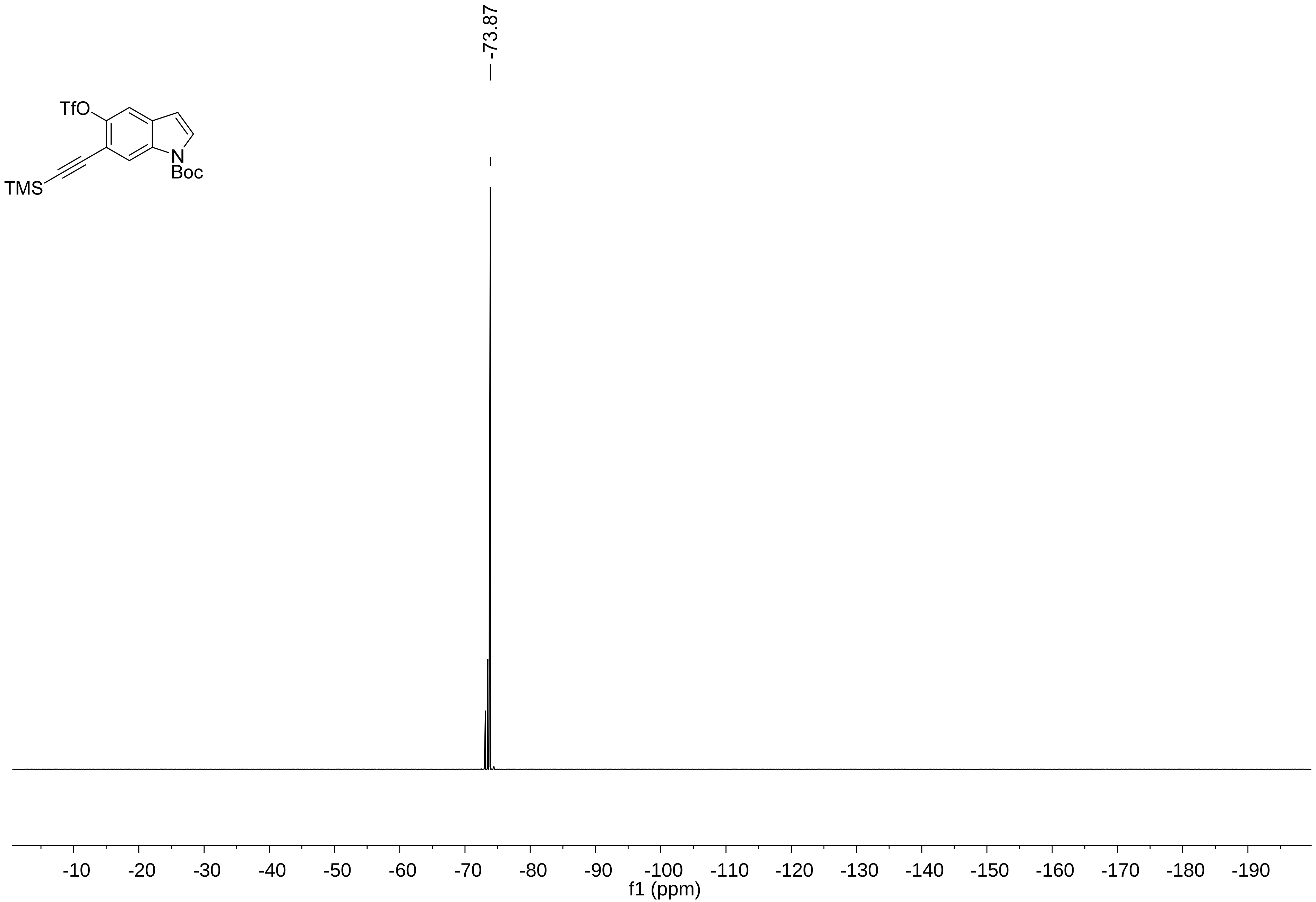

$^{1}H$ NMR ($CDCl_3$, 600 MHz)

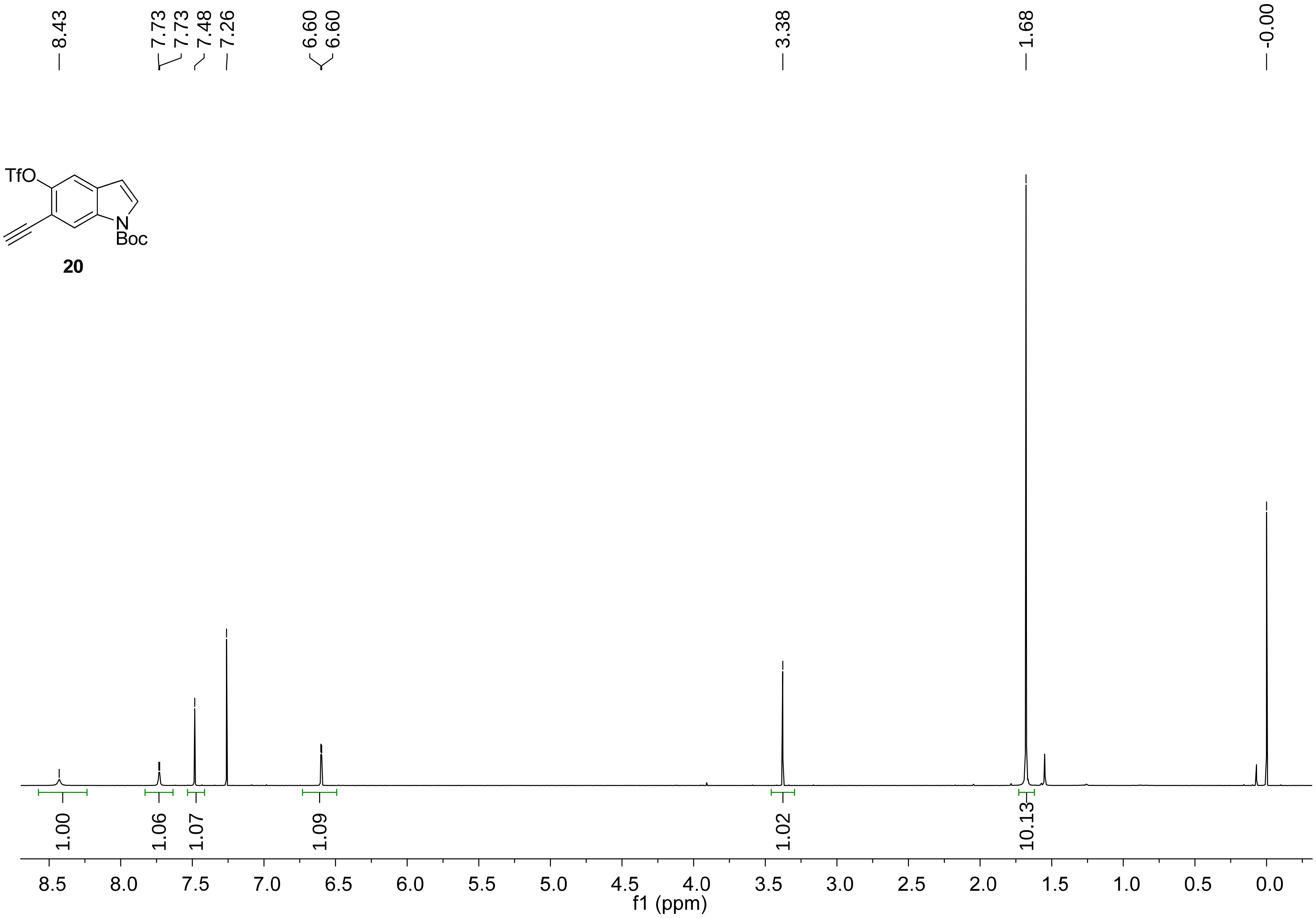

$^{13}C$ NMR ($CDCl_3$, 150 MHz)

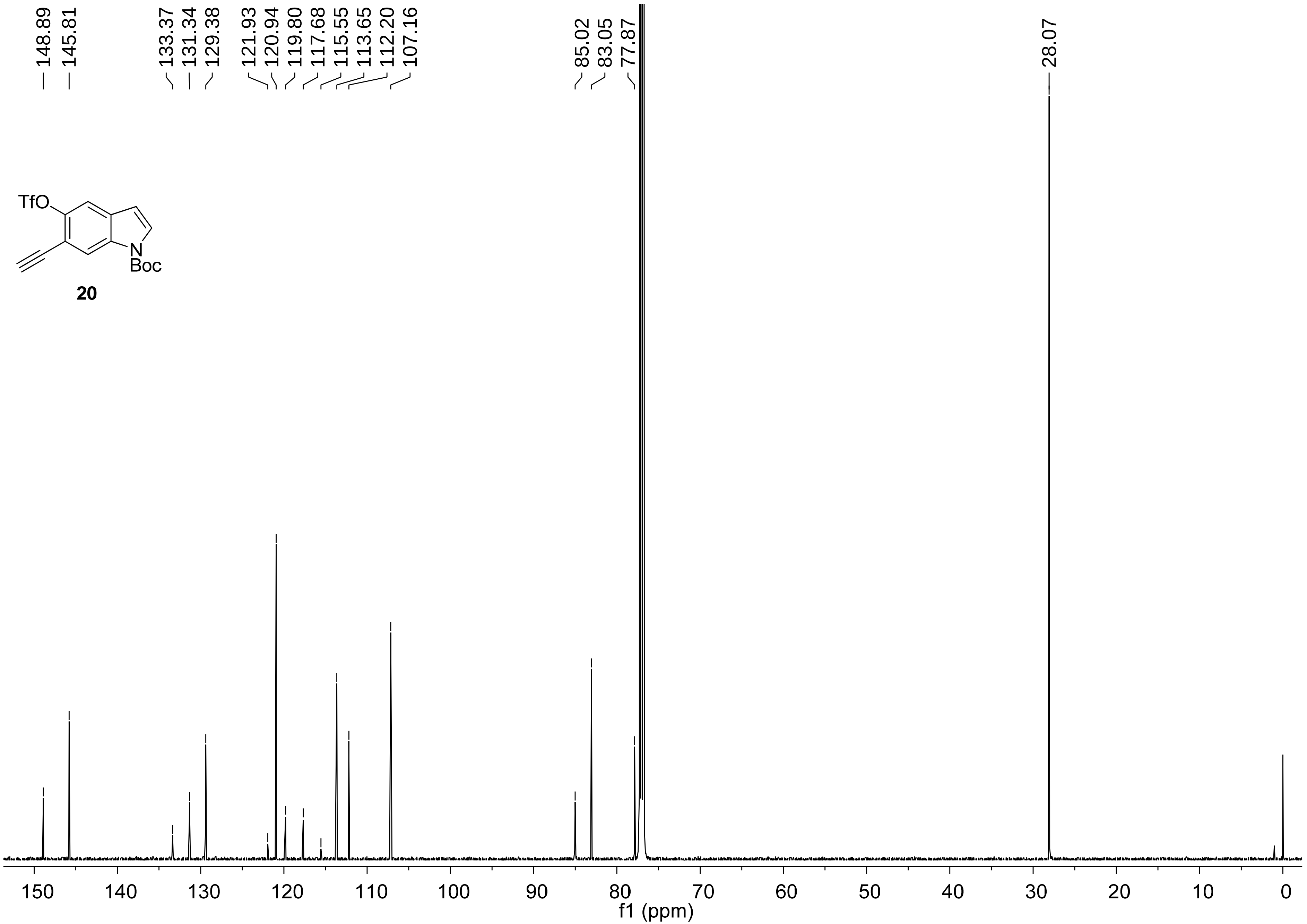


20

$^{19}F$ NMR ($CDCl_3$, 376 MHz)

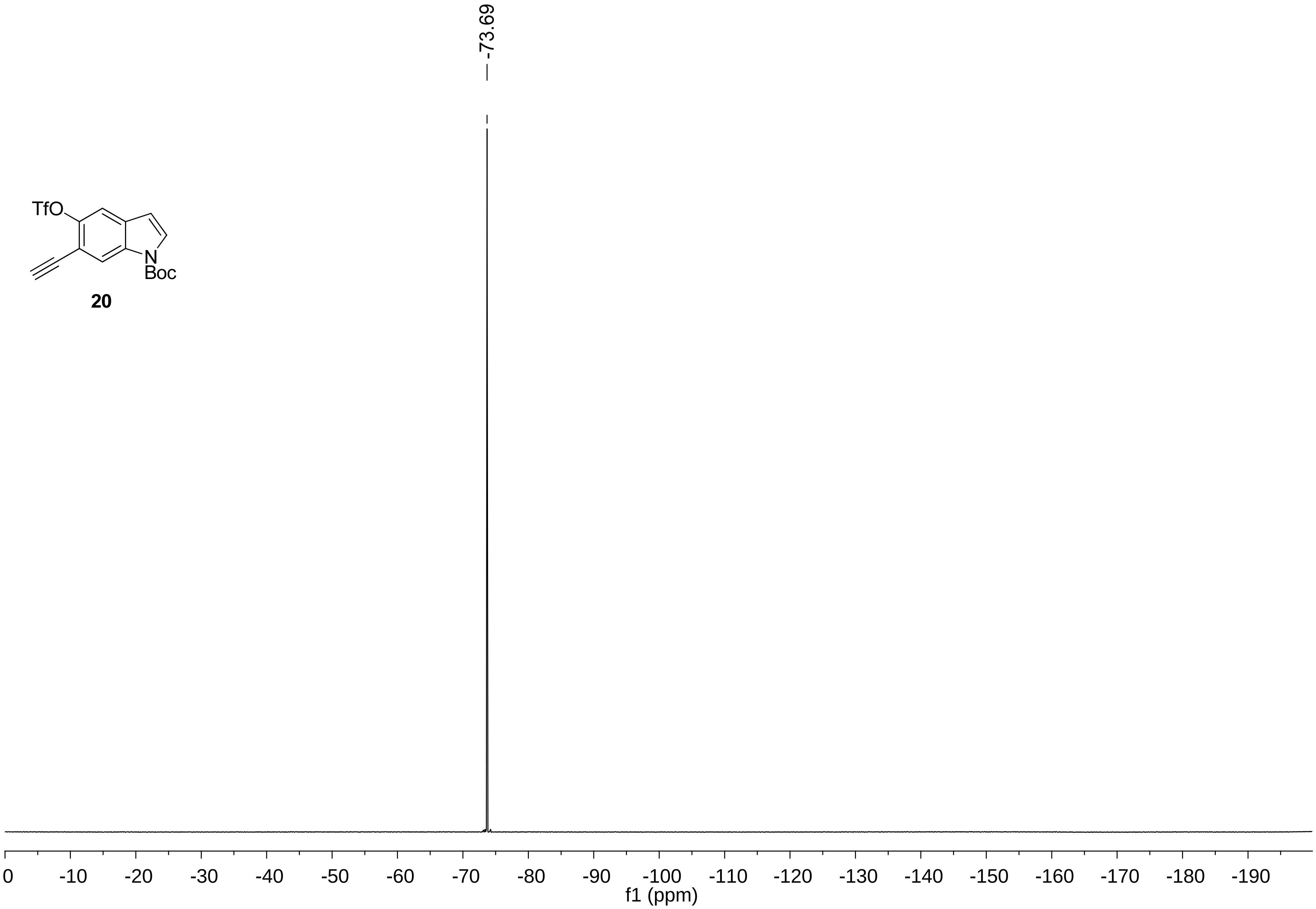


**20**

$^{1}$H NMR ($CDCl_3$, 600 MHz)

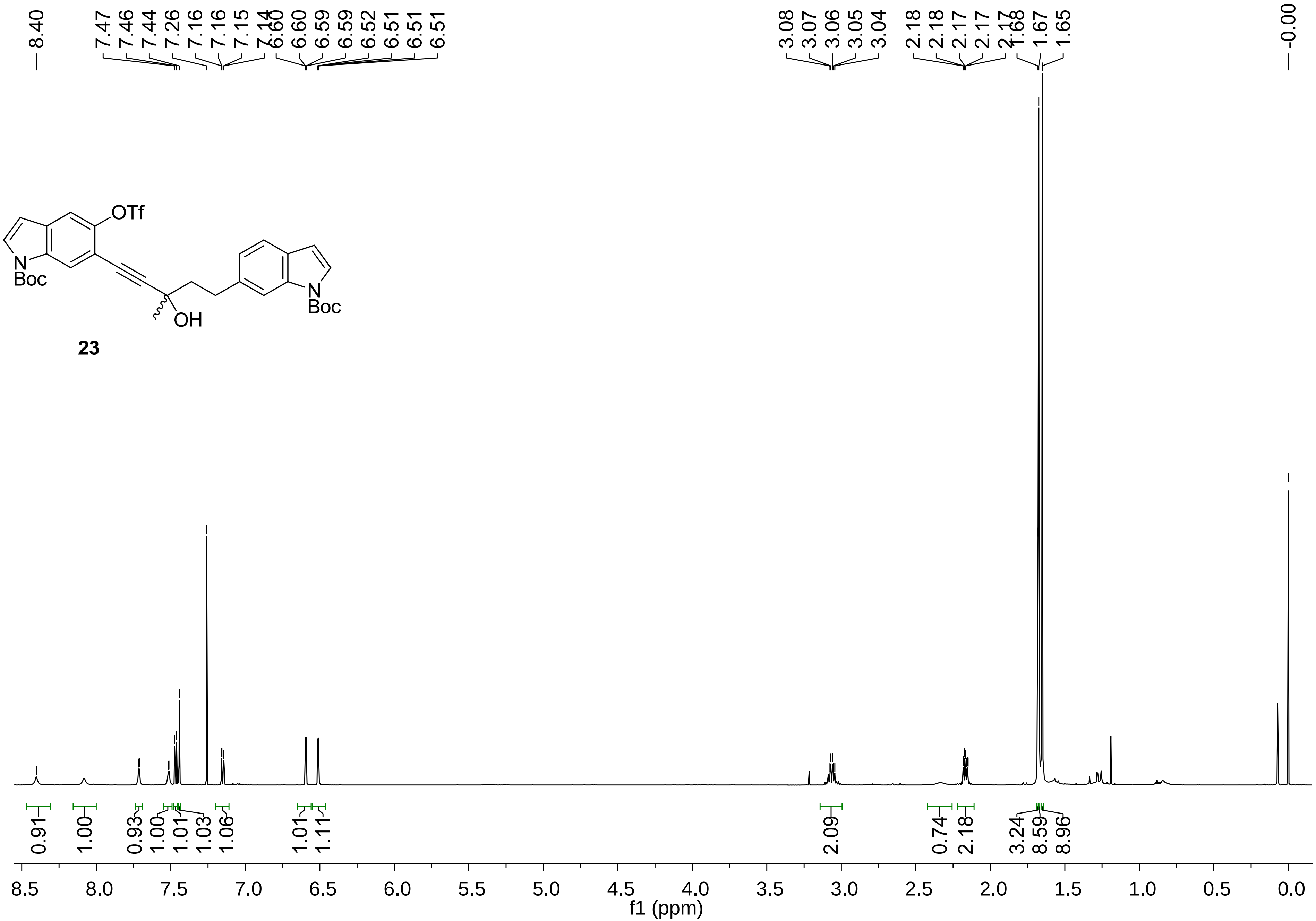

$^{13}C$ NMR ($CDCl_3$, 150 MHz)

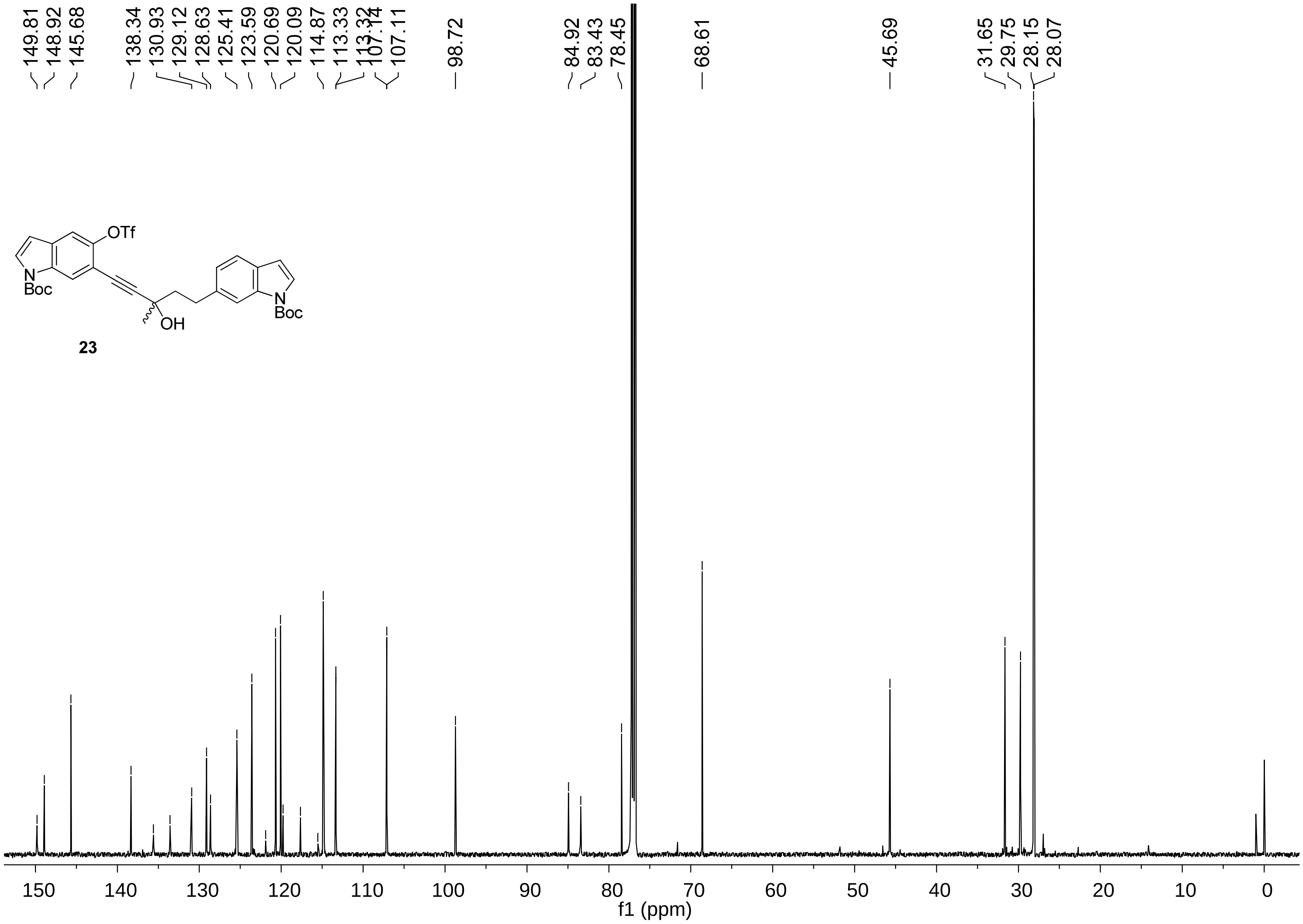

$^{19}F$ NMR ($CDCl_3$, 376 MHz)

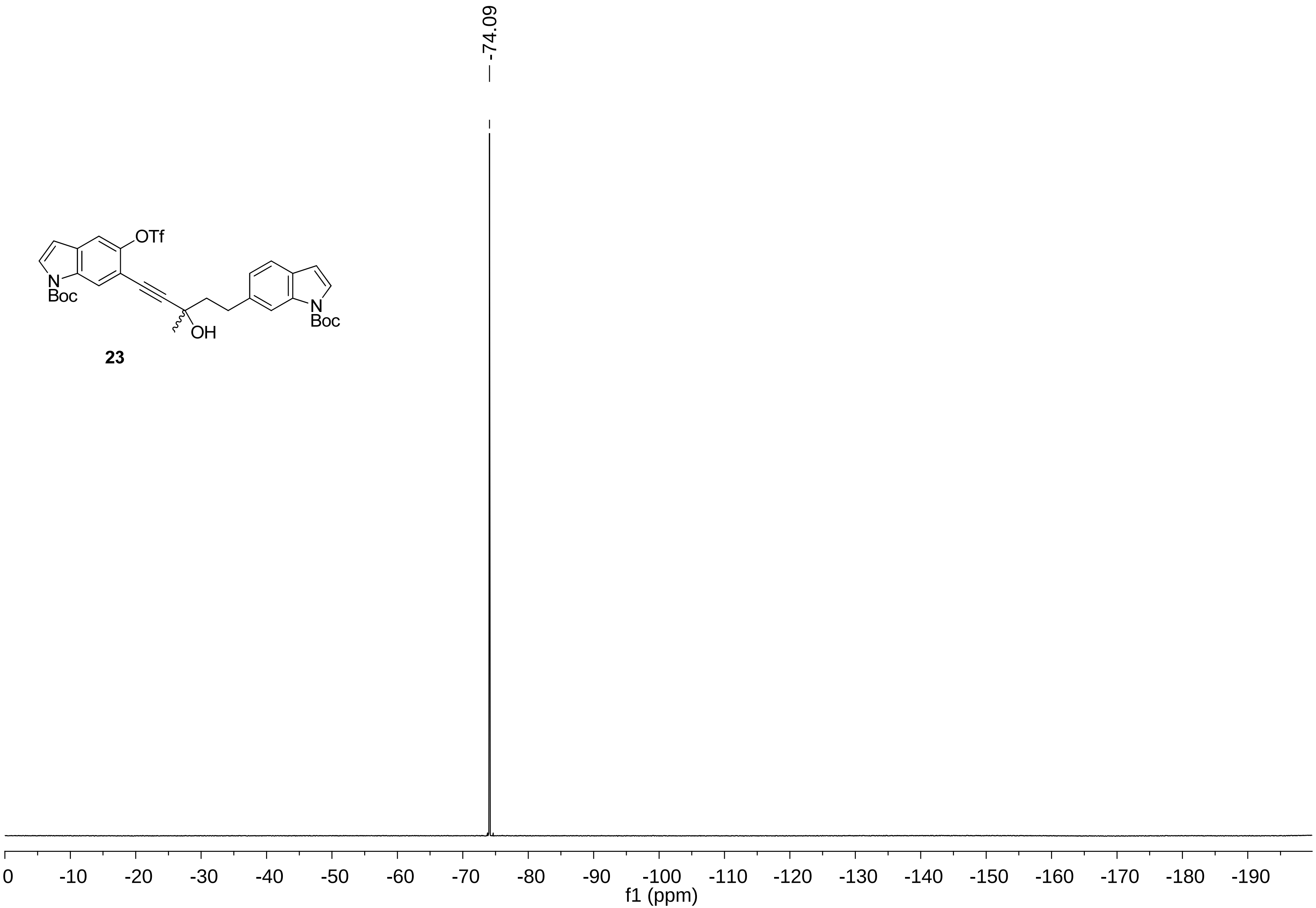

$^1$H NMR ($CDCl_3$, 600 MHz)

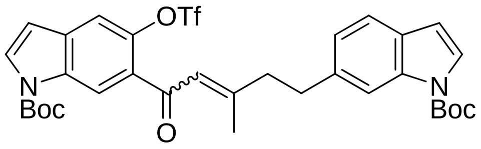


**26**

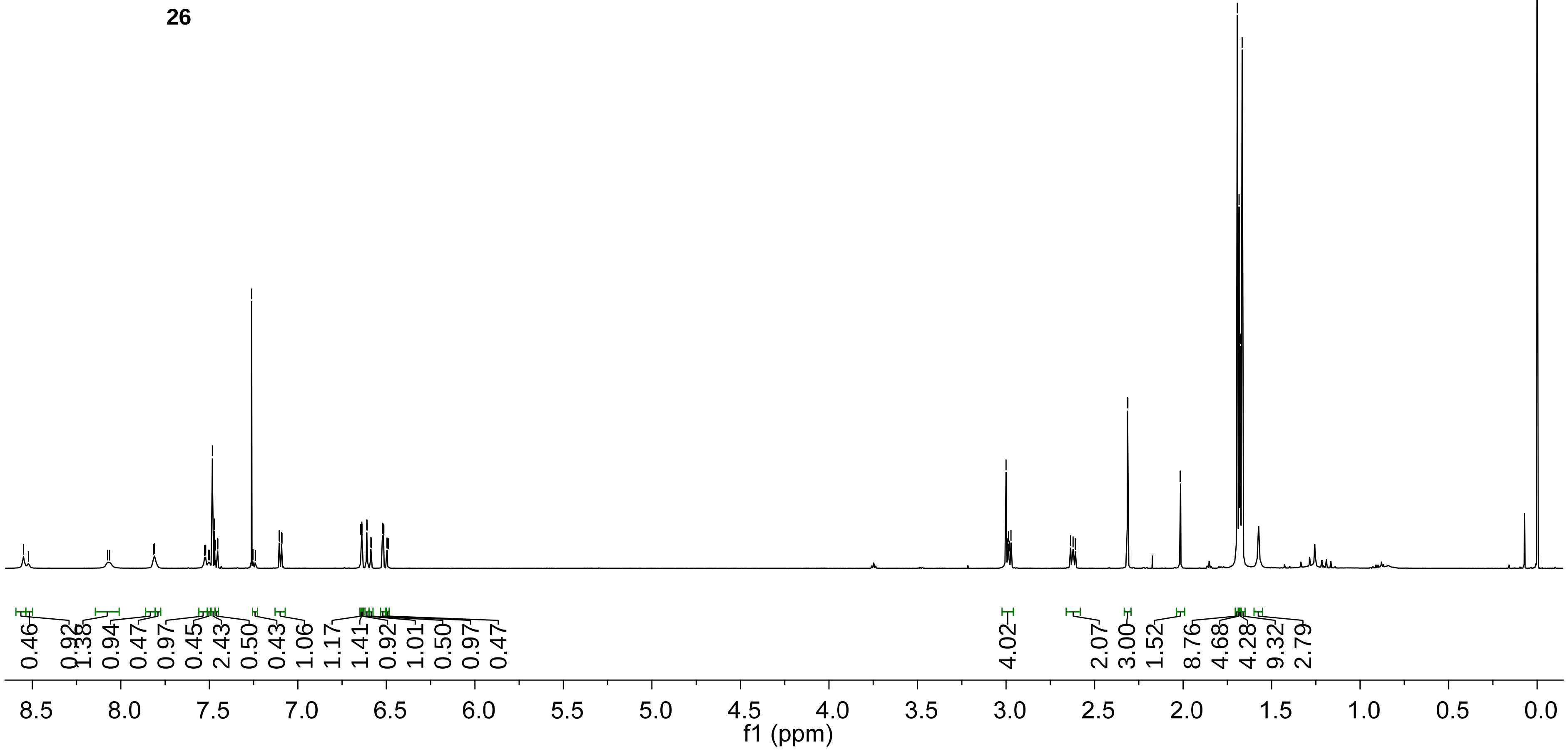

$^{13}C$ NMR ($CDCl_3$, 150 MHz)

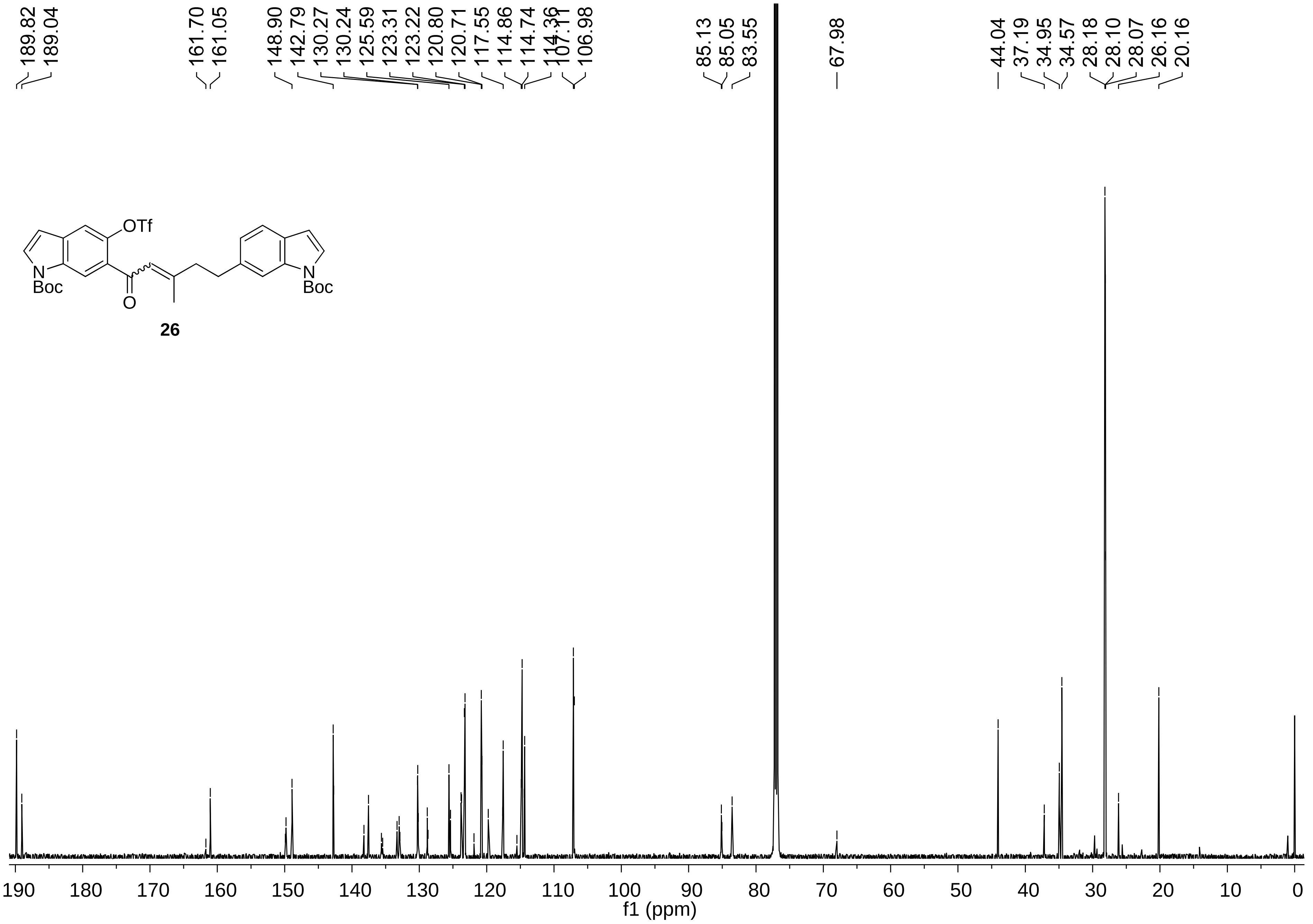

$^{19}F$ NMR ($CDCl_3$, 376 MHz)

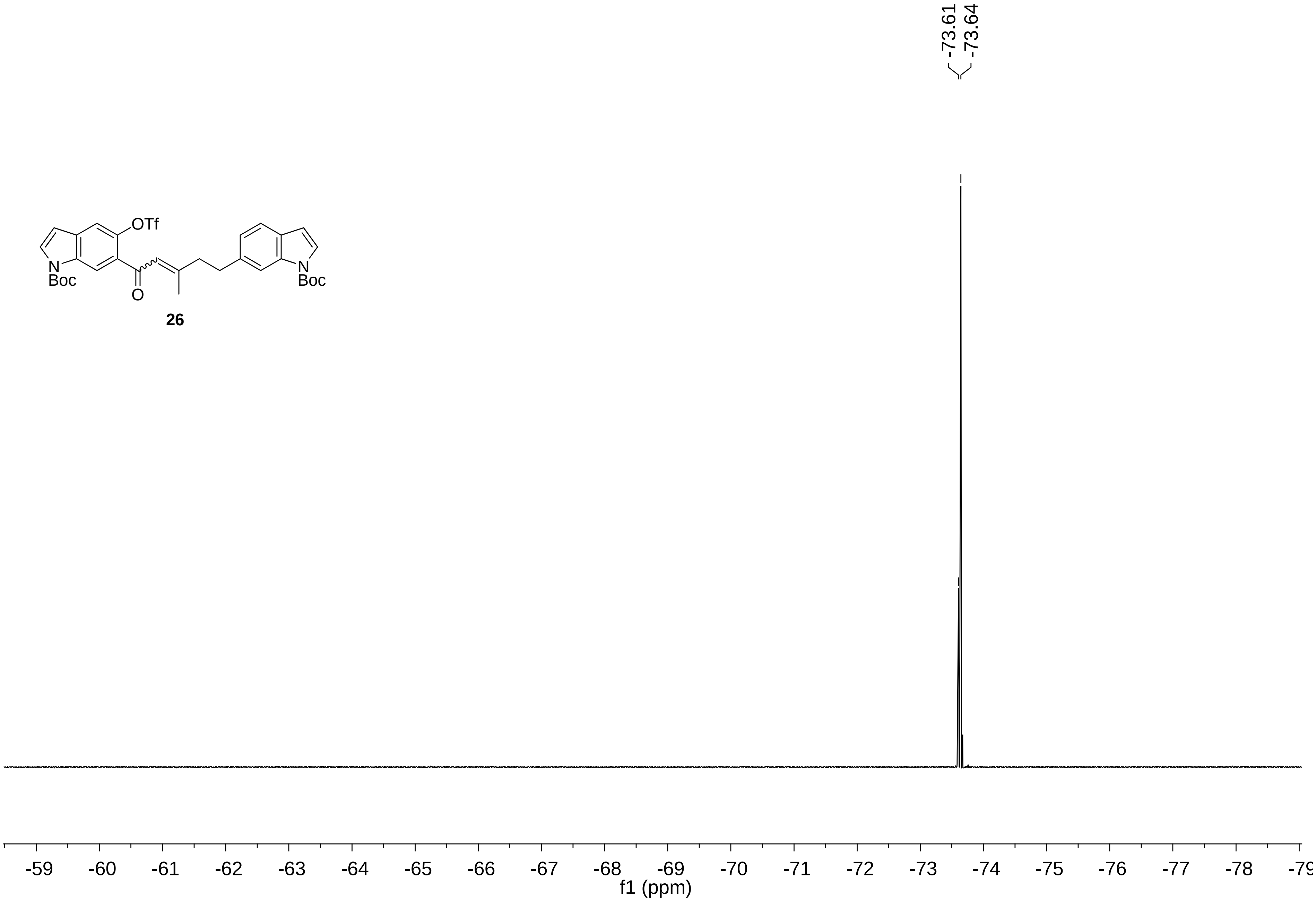

$^{1}H$ NMR (400 MHz, $CDCl_3$)

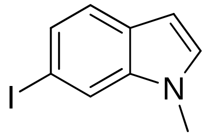


**28**

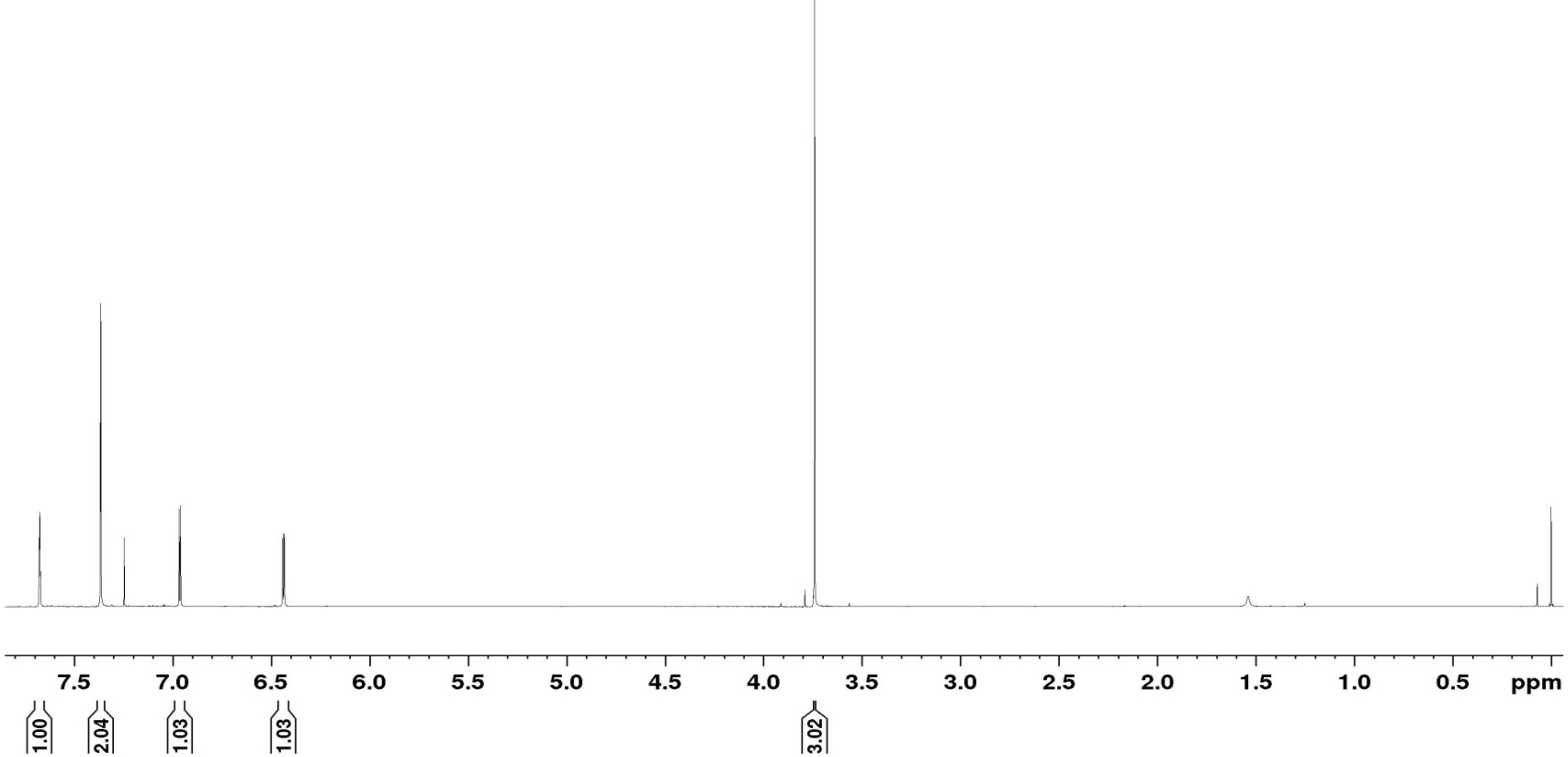

$^{13}C$ NMR (100 MHz, $CDCl_3$)

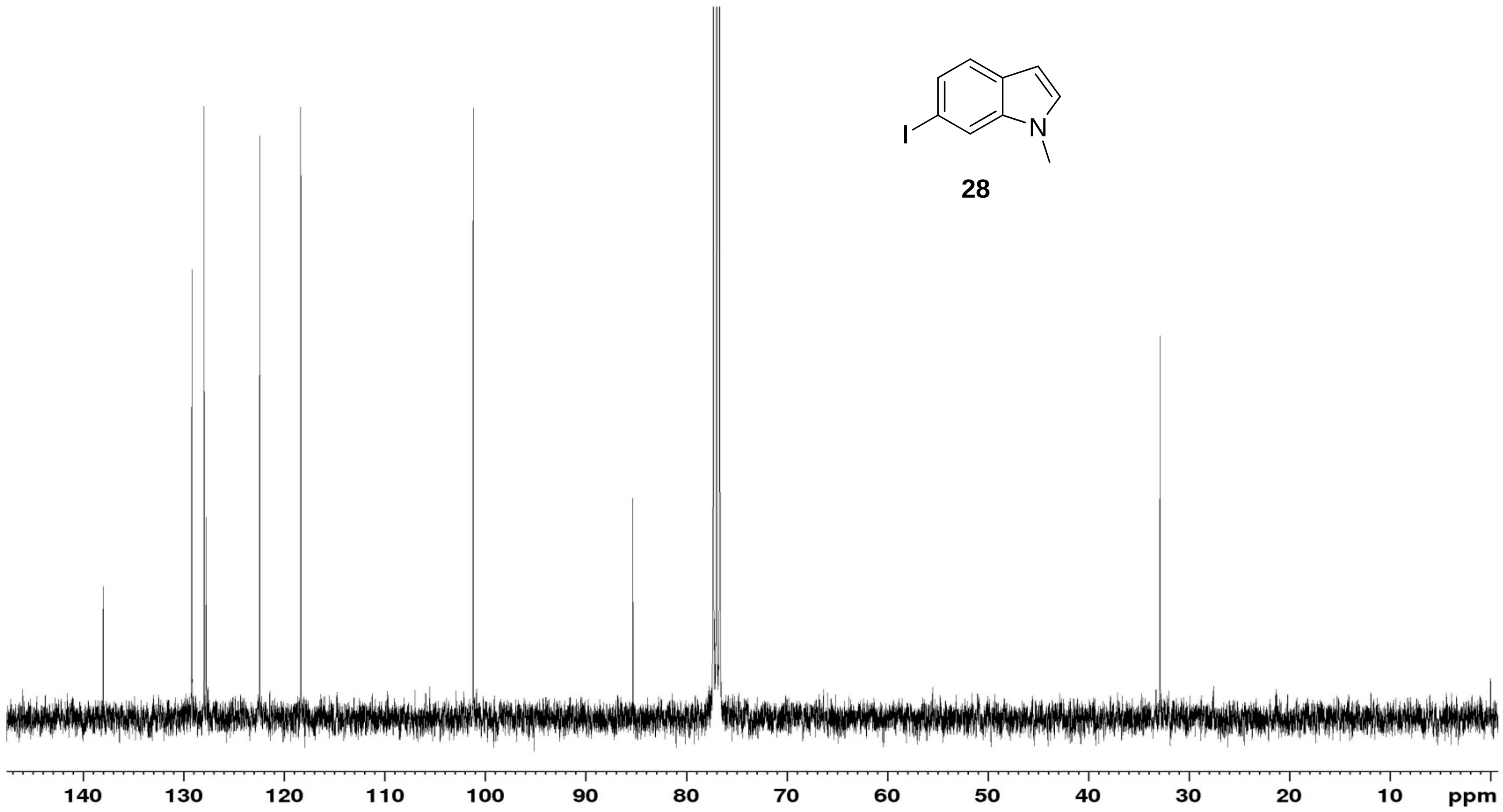


28

$^{1}H$ NMR (400 MHz, acetone-$d_6$)

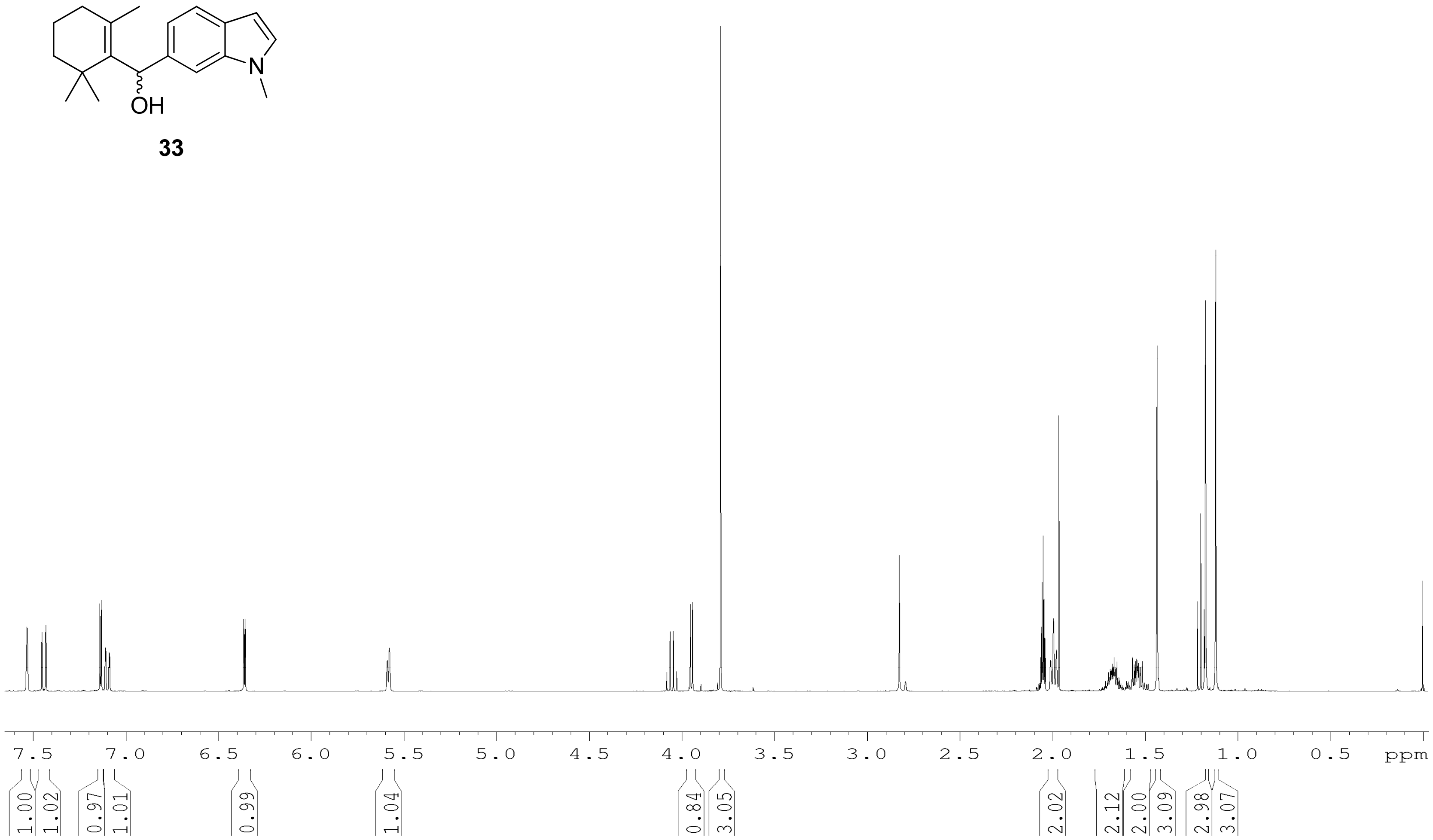

$^{13}C$ NMR (100 MHz, acetone-$d_6$)

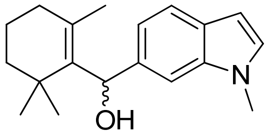


**33**

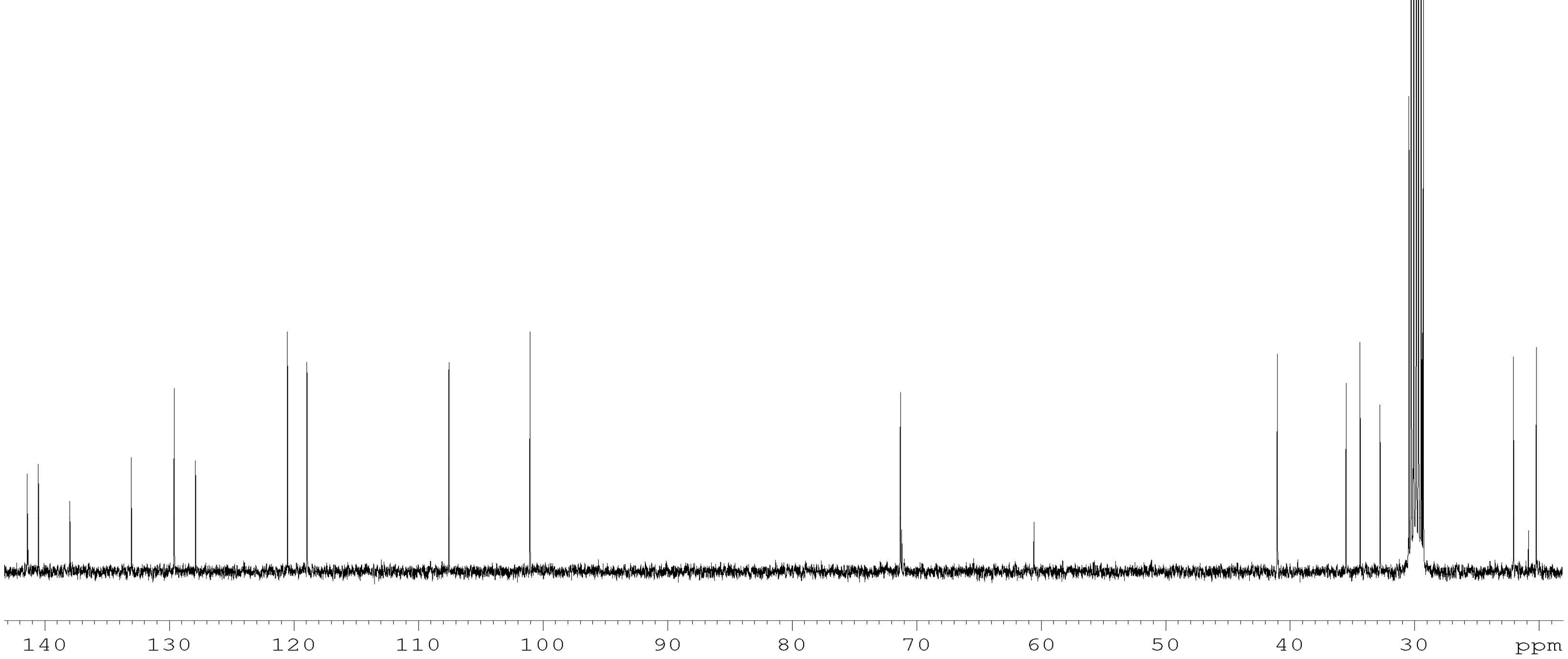

$^{1}H$ NMR (600 MHz, $CDCl_3$)

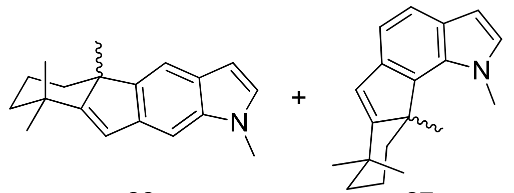

36 37

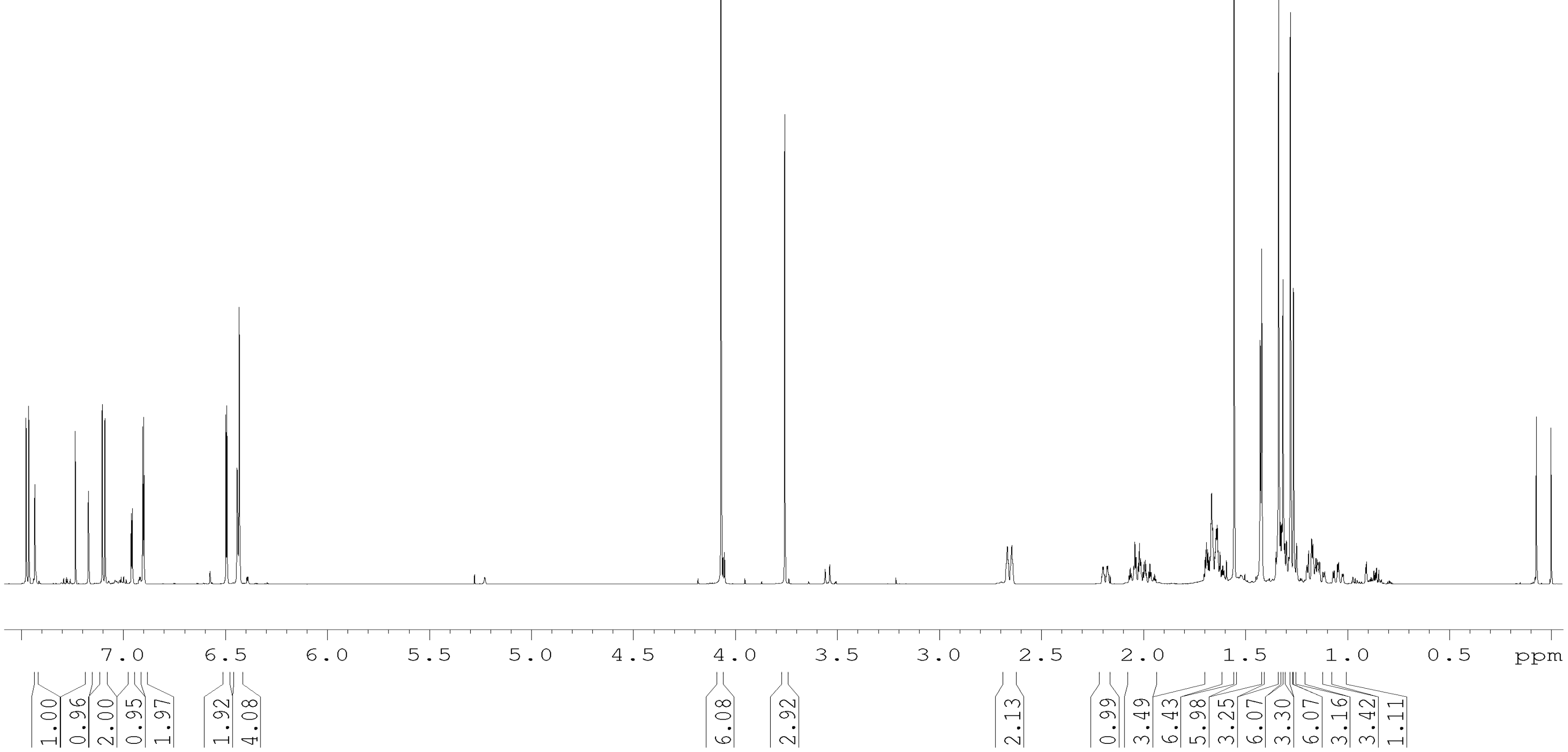

$^{13}C$ NMR (150 MHz, $CDCl_3$)

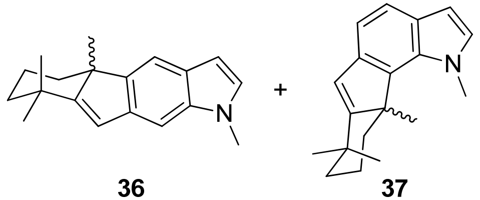

**36** + **37**

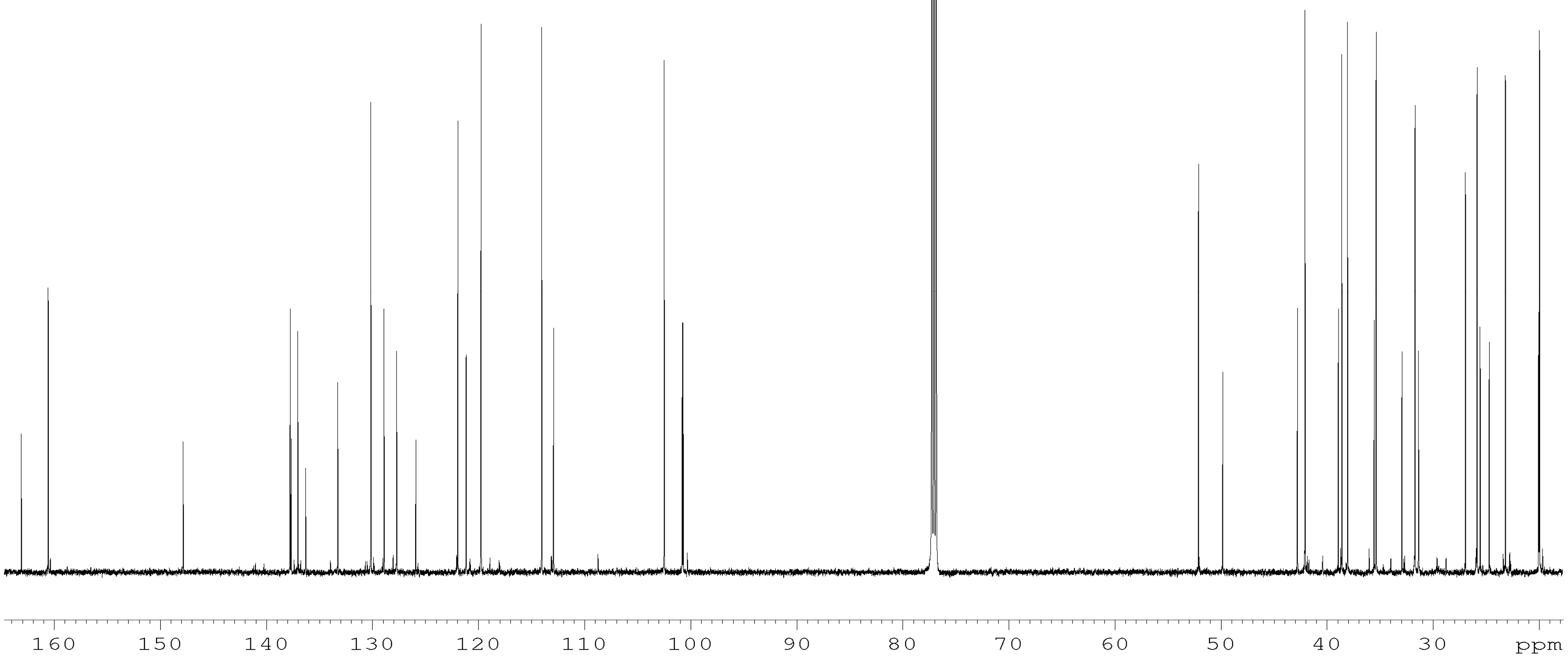

$^{1}H$ NMR (400 MHz, $CDCl_3$)

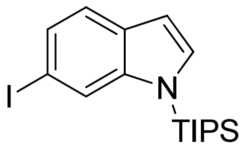

**29**

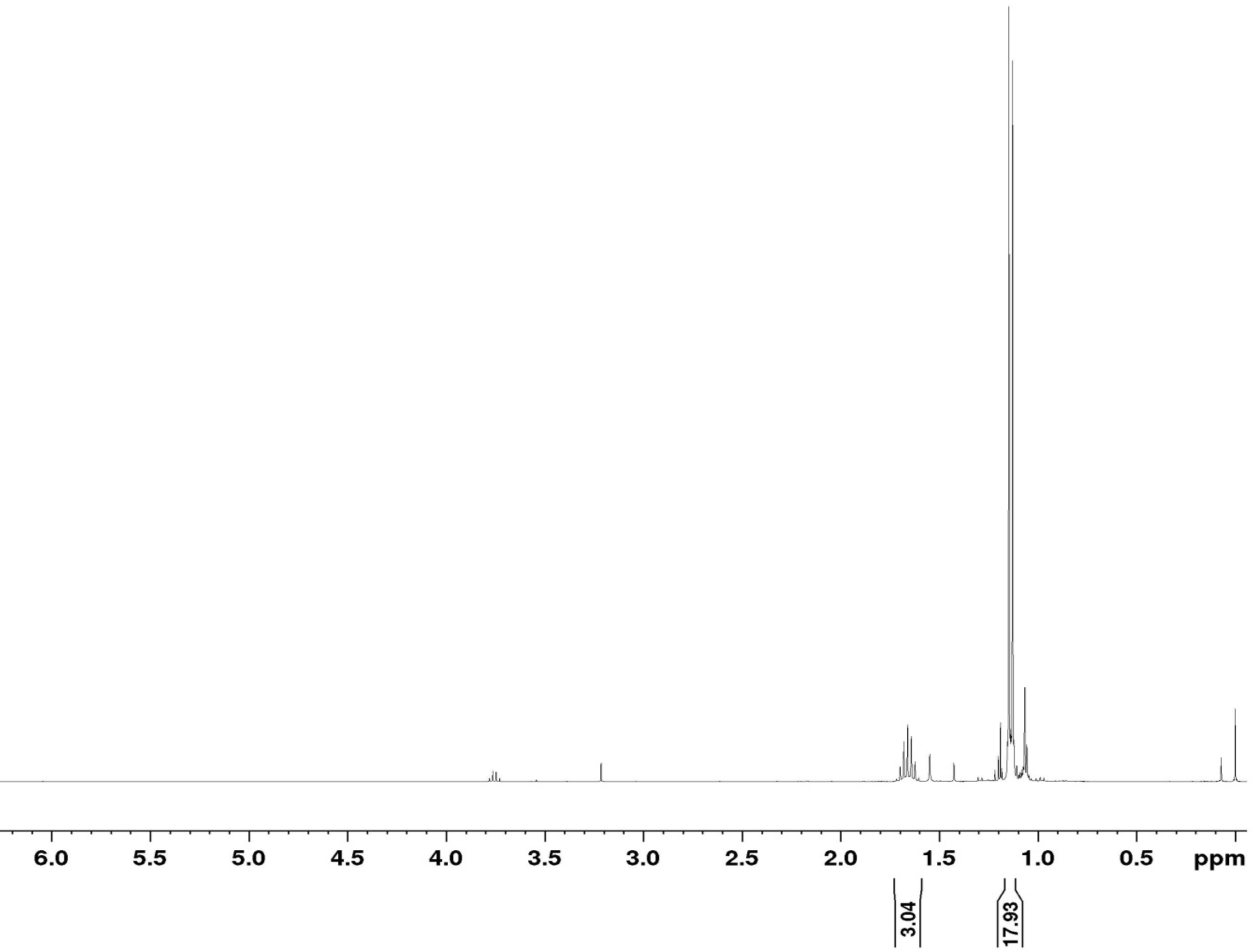

$^{13}C$ NMR (100 MHz, $CDCl_3$)

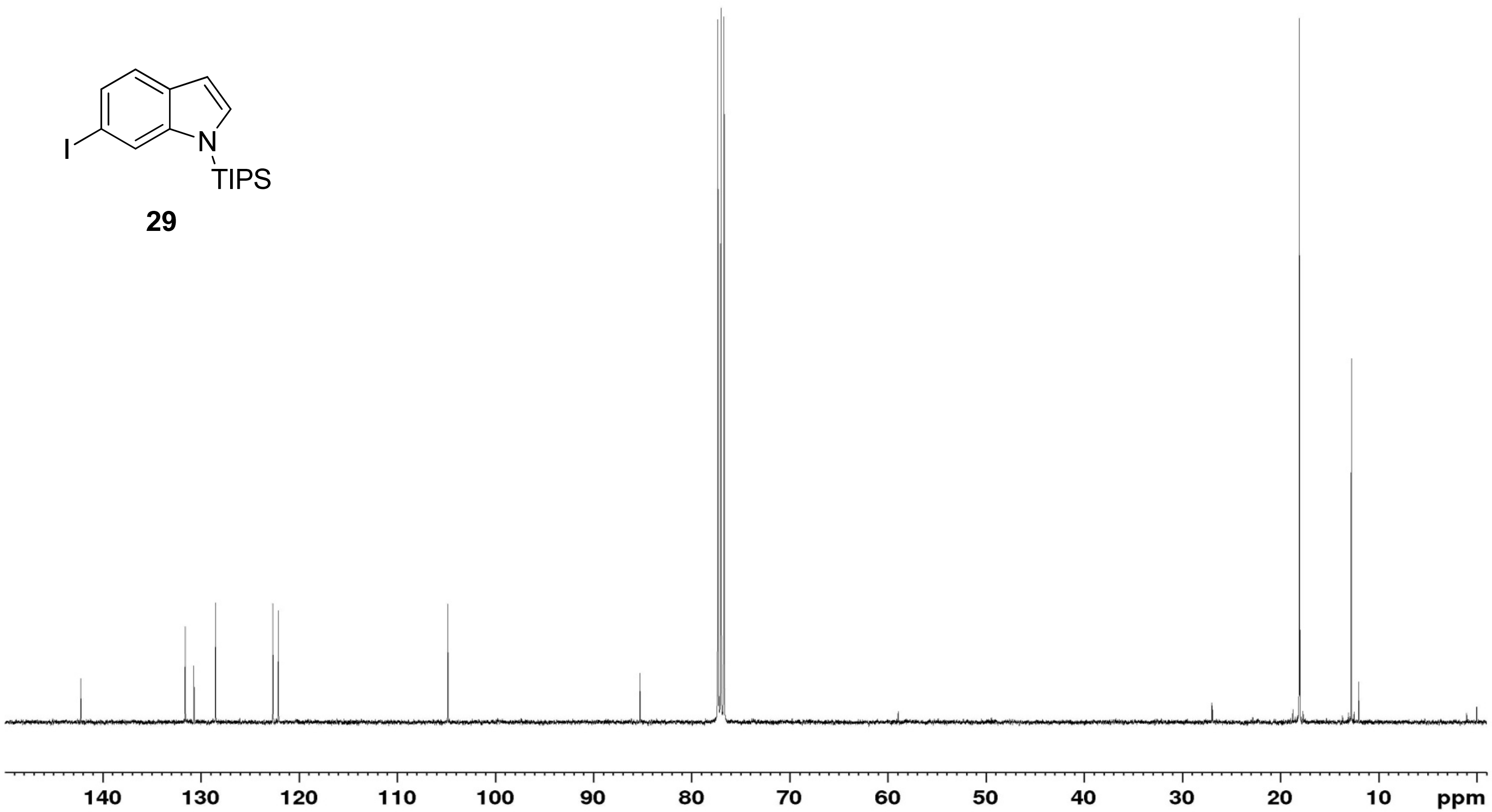


**29**

$^{1}H$ NMR (400 MHz, acetone-$d_6$)

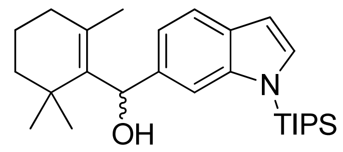


**33**

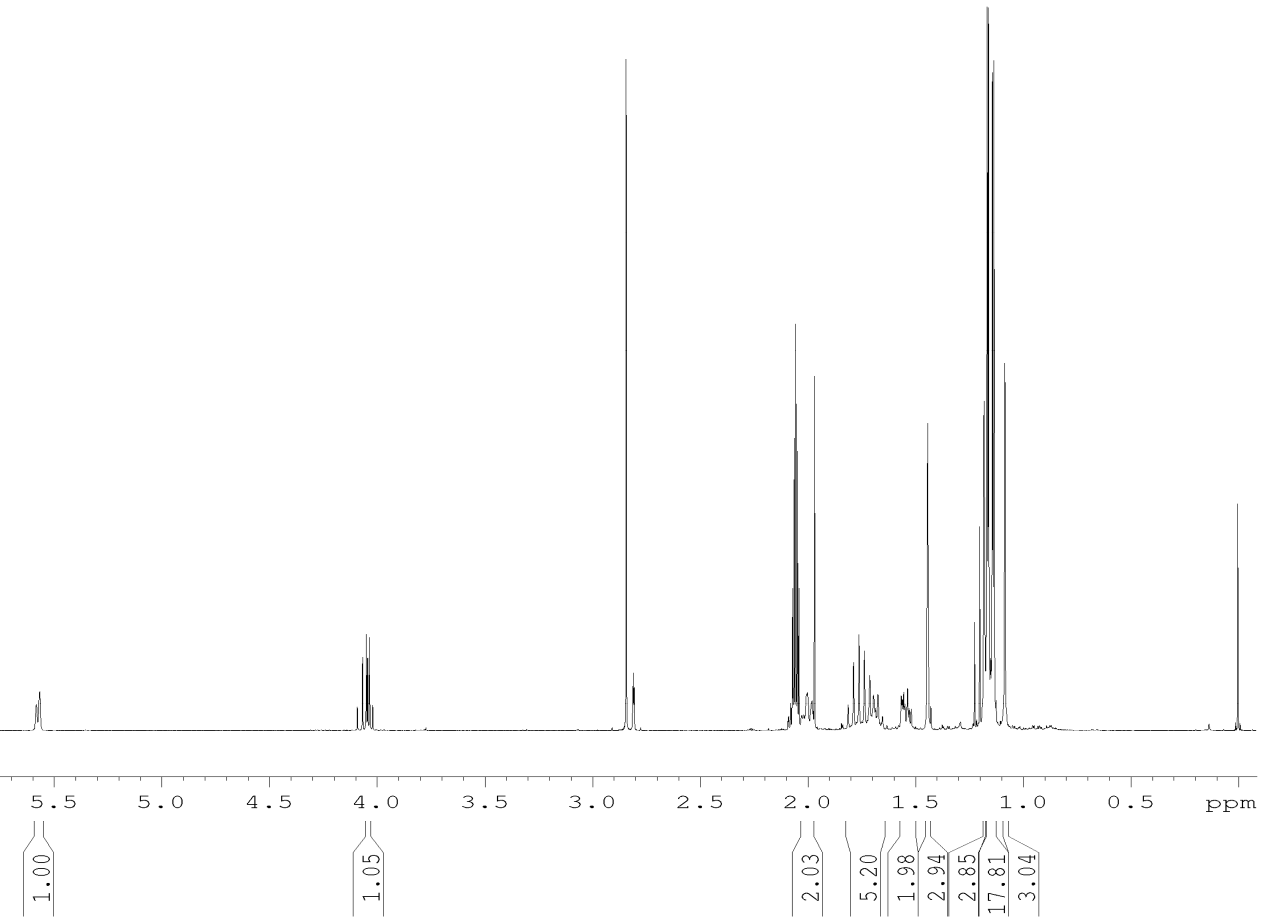

$^{13}C$ NMR (100 MHz, acetone-$d_6$)

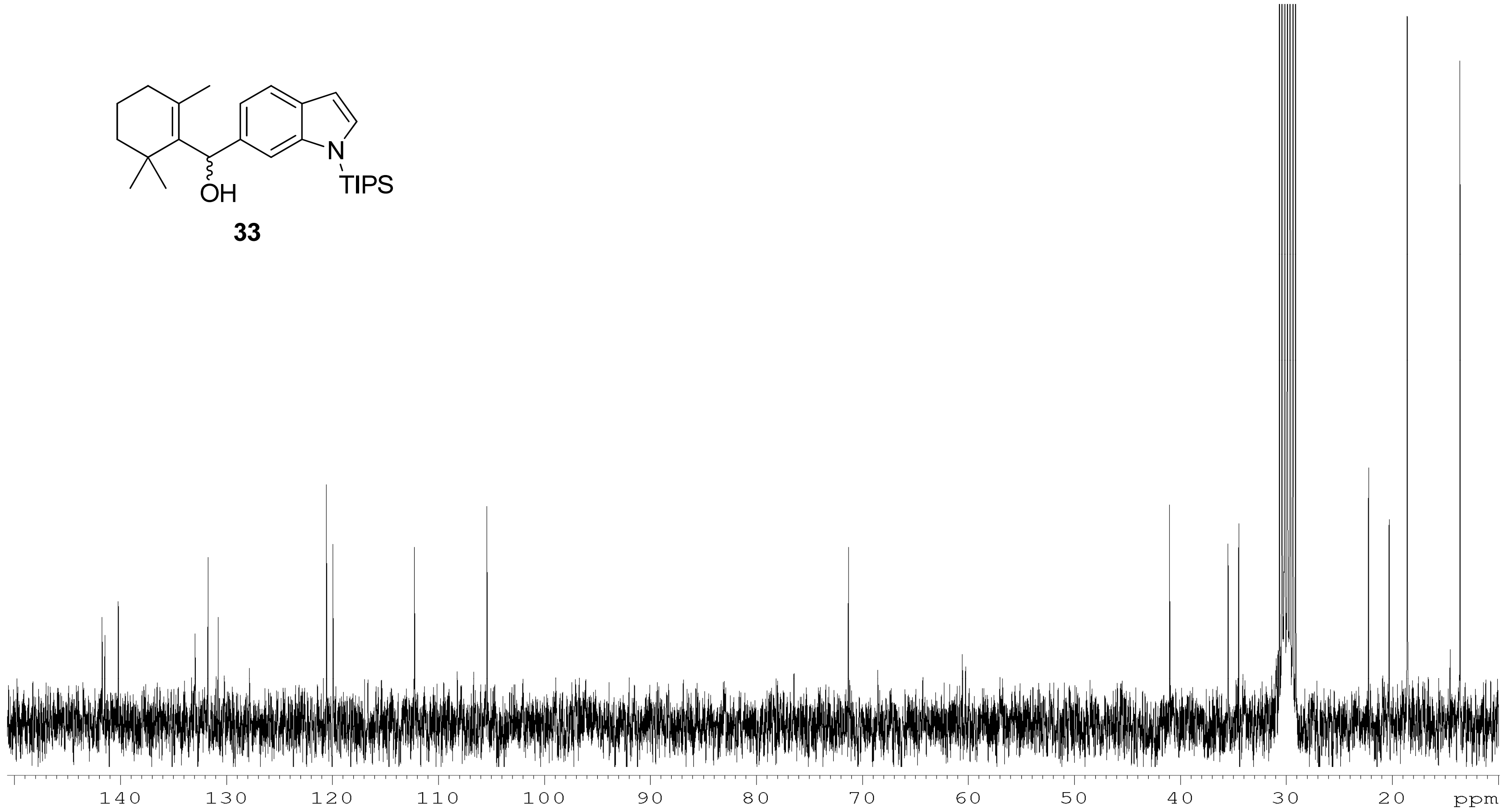

$^{1}H$ NMR (400 MHz, $CDCl_3$)

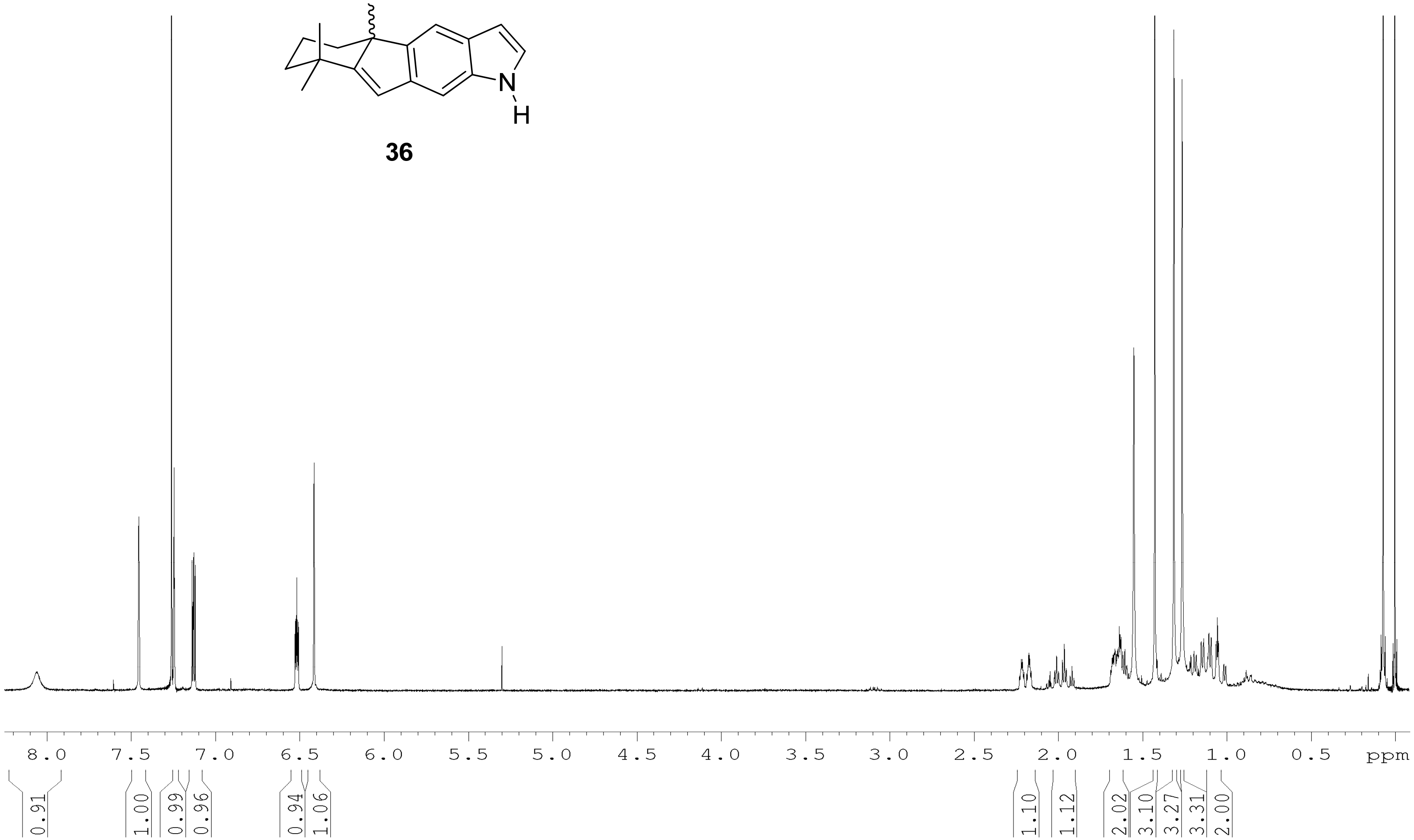

$^{13}C$ NMR (100 MHz, $CDCl_3$)

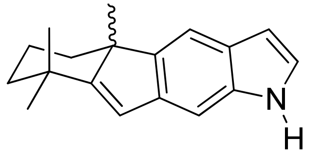

**36**

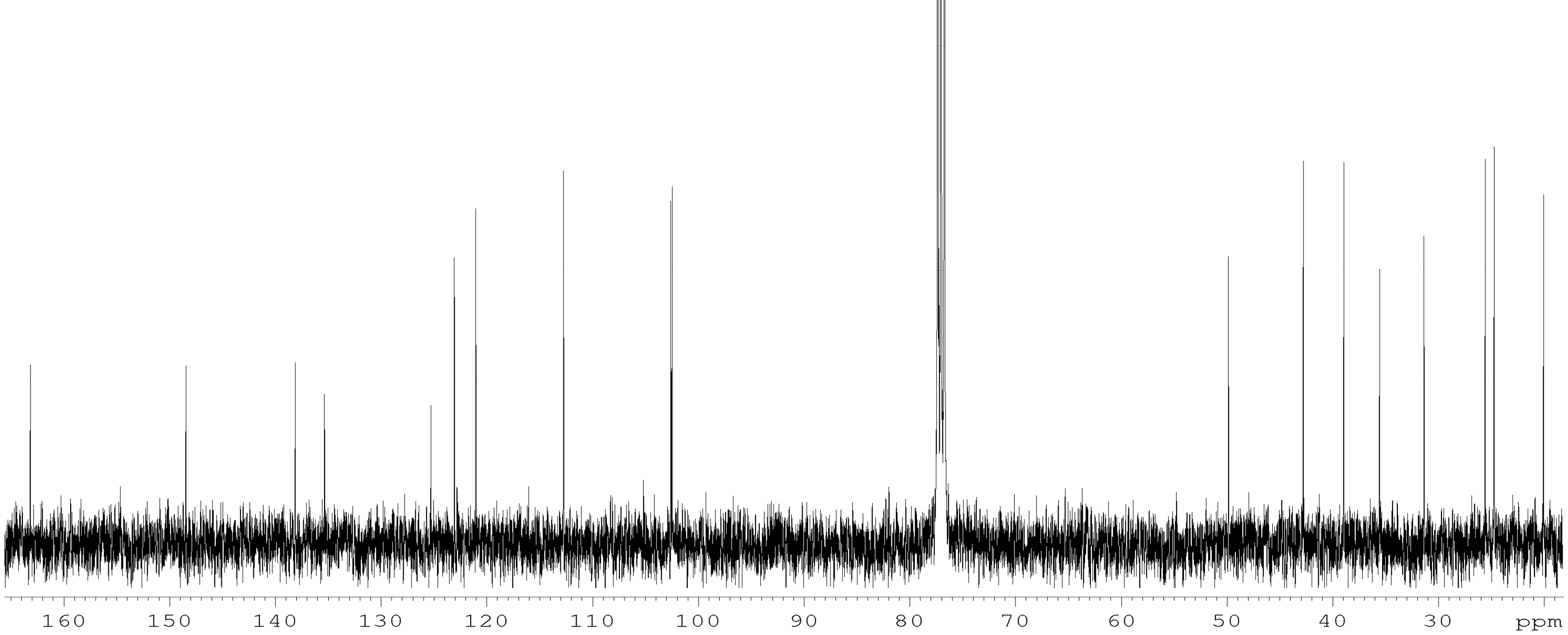

$^{1}H$ NMR (400 MHz, $CDCl_3$)

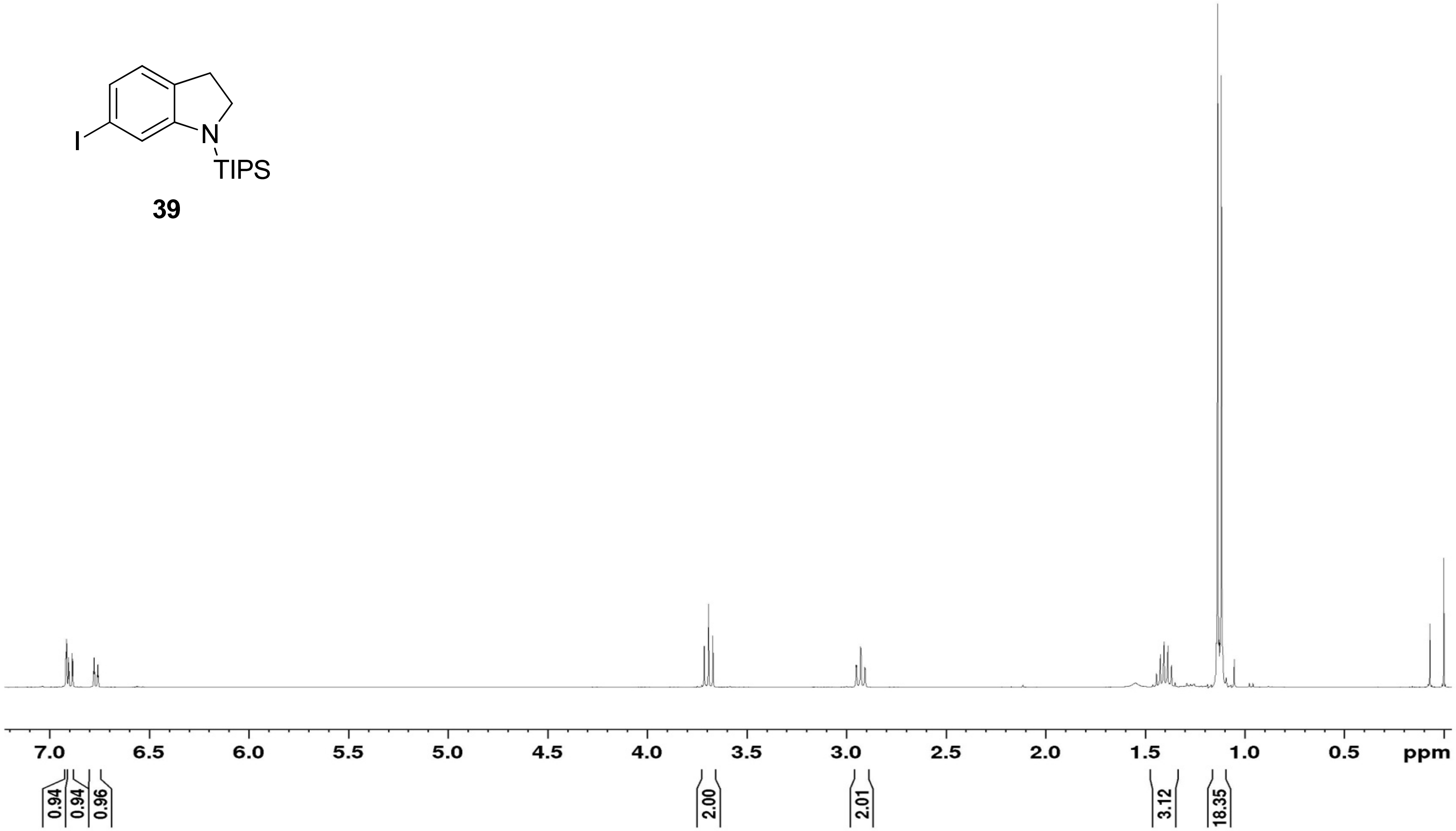

$^{13}C$ NMR (100 MHz, $CDCl_3$)

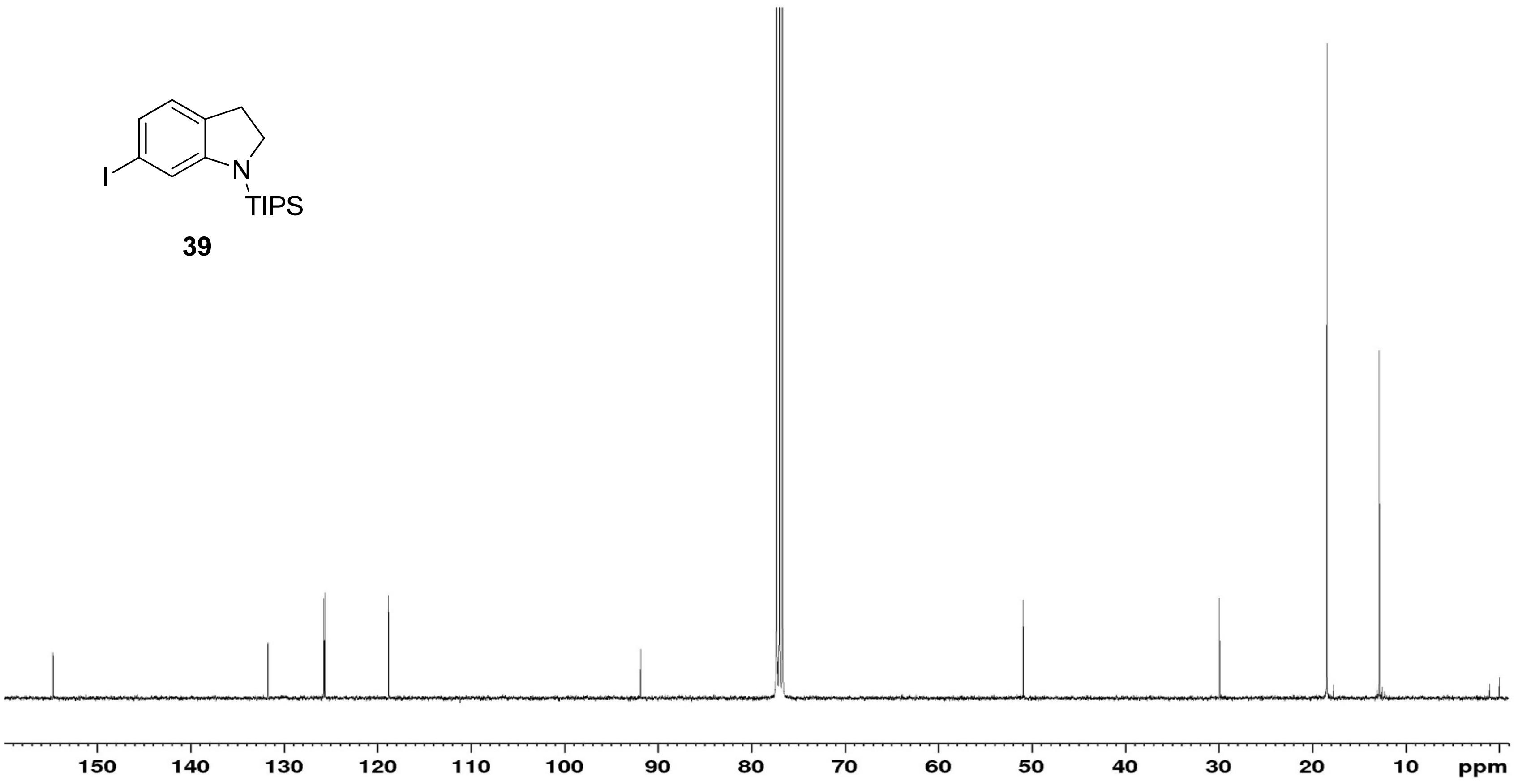


**39**

$^{1}H$ NMR (400 MHz, acetone-$d_6$)

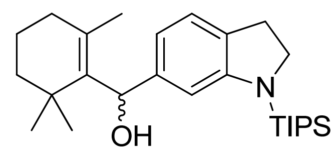


**40**

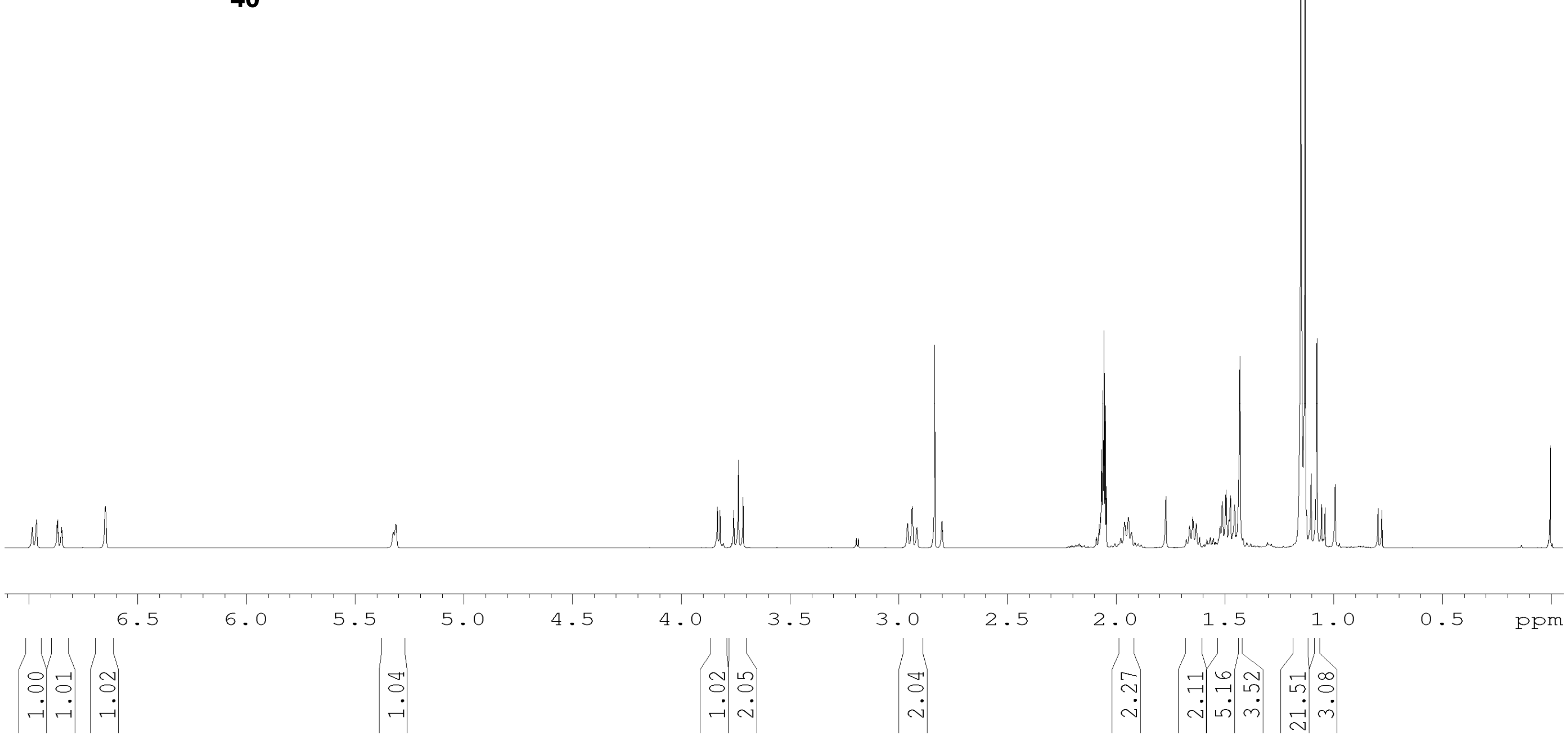

$^{13}$C NMR (100 MHz, acetone-$d_6$)

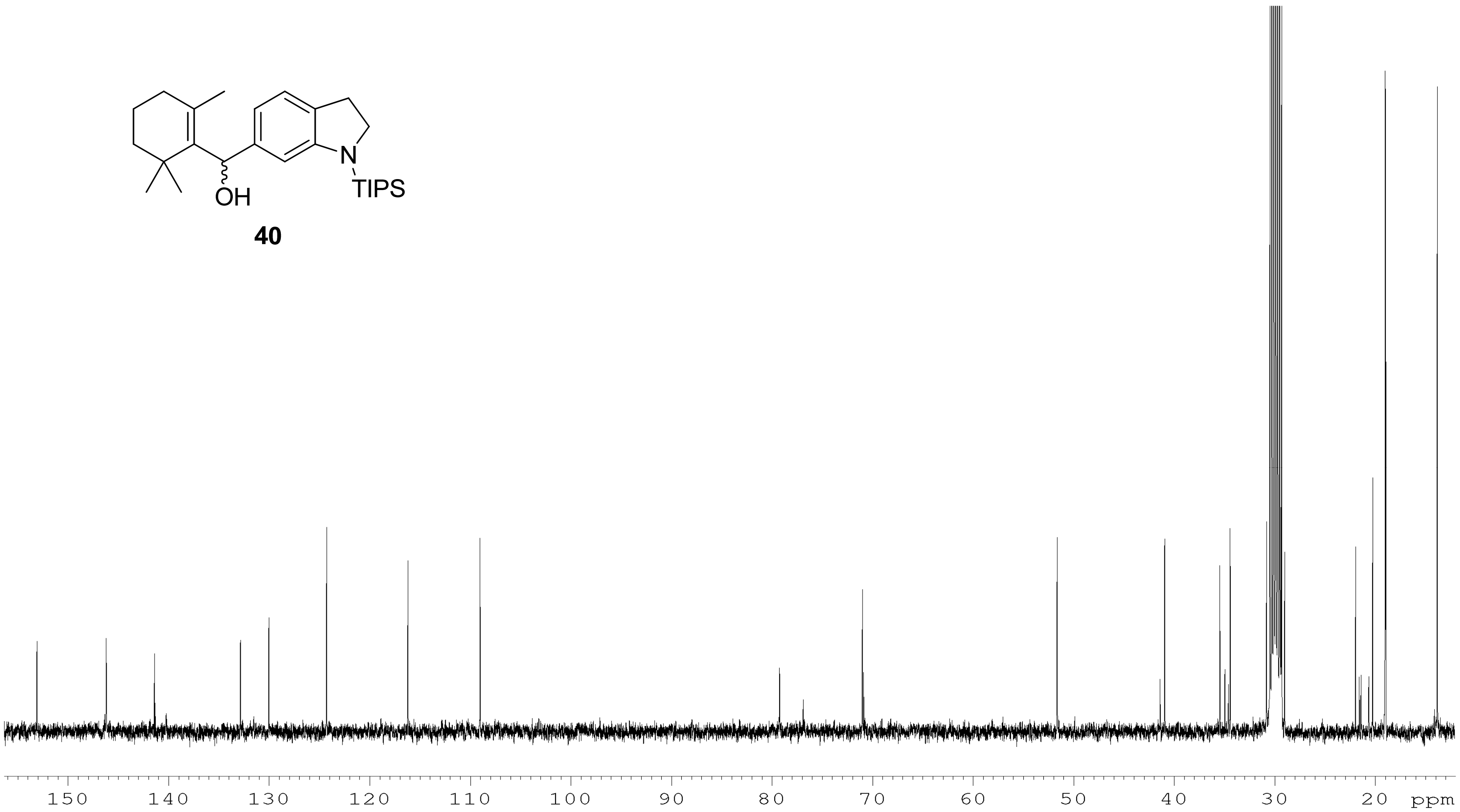

$^{1}H$ NMR (400 MHz, $CDCl_3$)

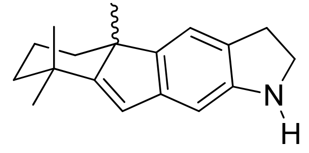

**41**

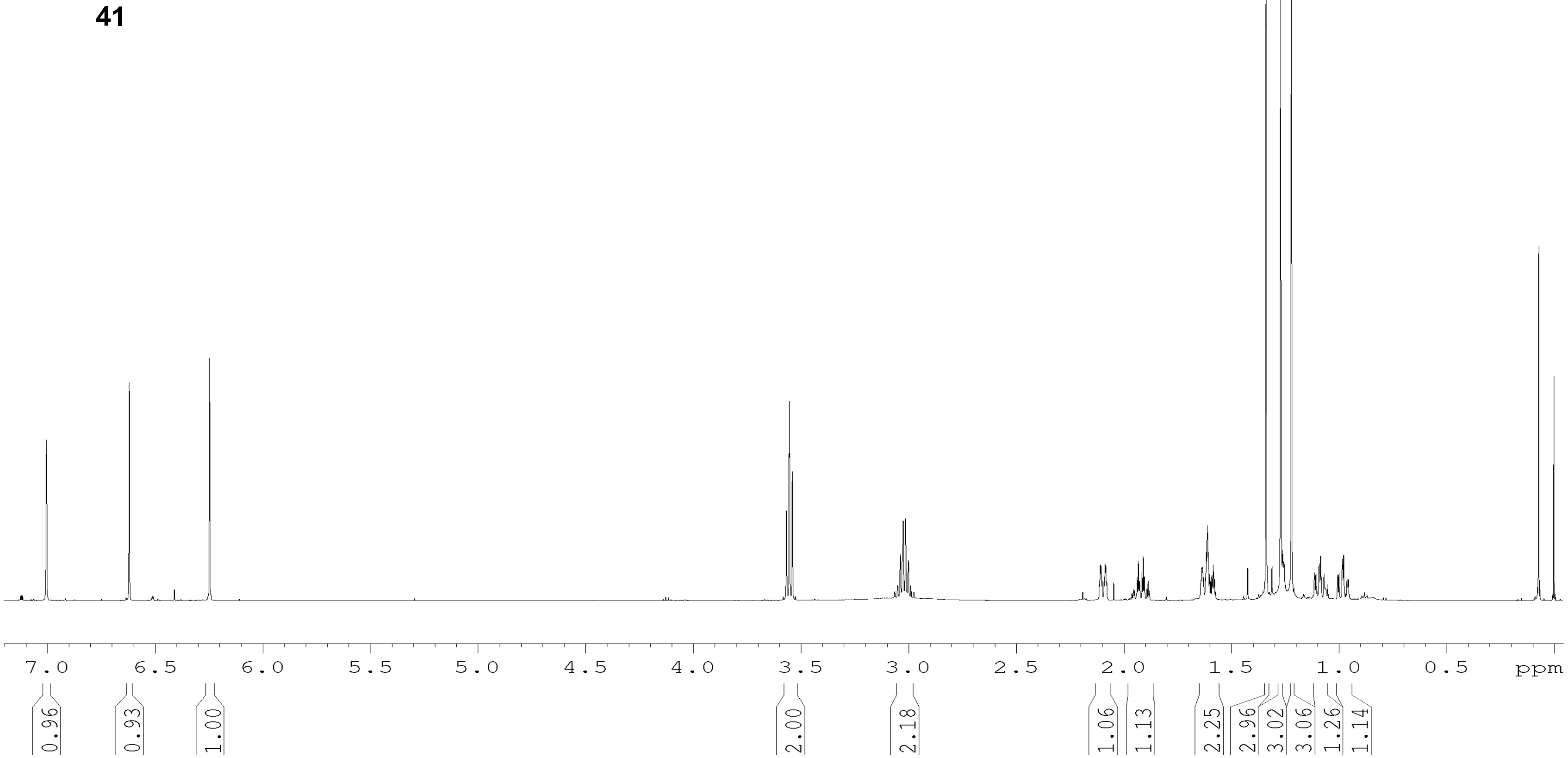

$^{13}C$ NMR (100 MHz, $CDCl_3$)

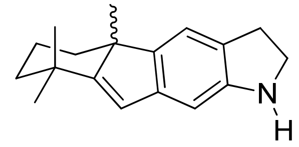


**41**

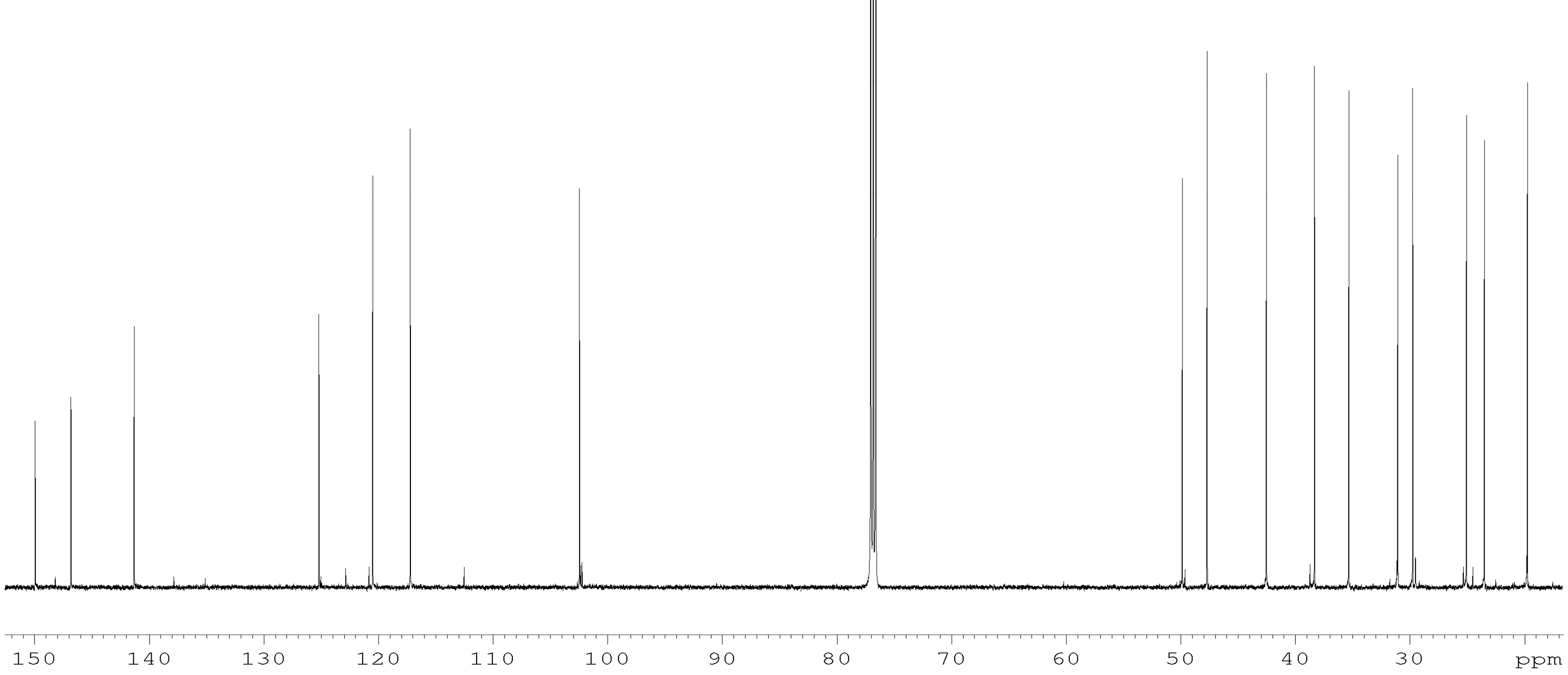

$^1$H NMR (400 MHz, $CDCl_3$)

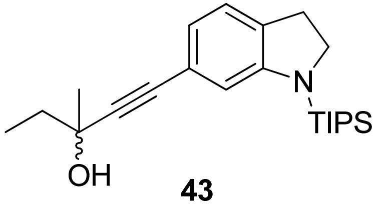


**43**

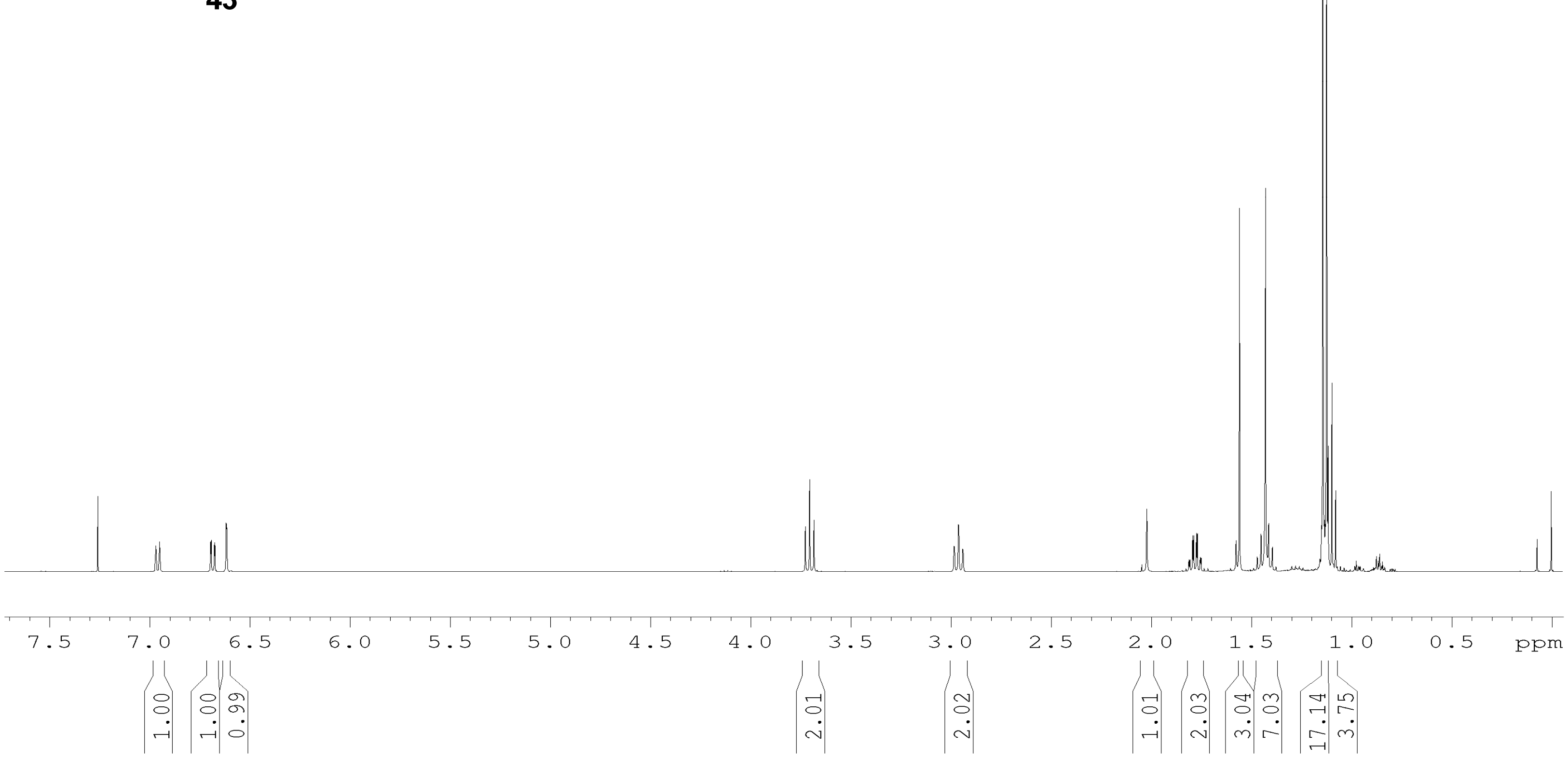

$^{13}C$ NMR (100 MHz, $CDCl_3$)

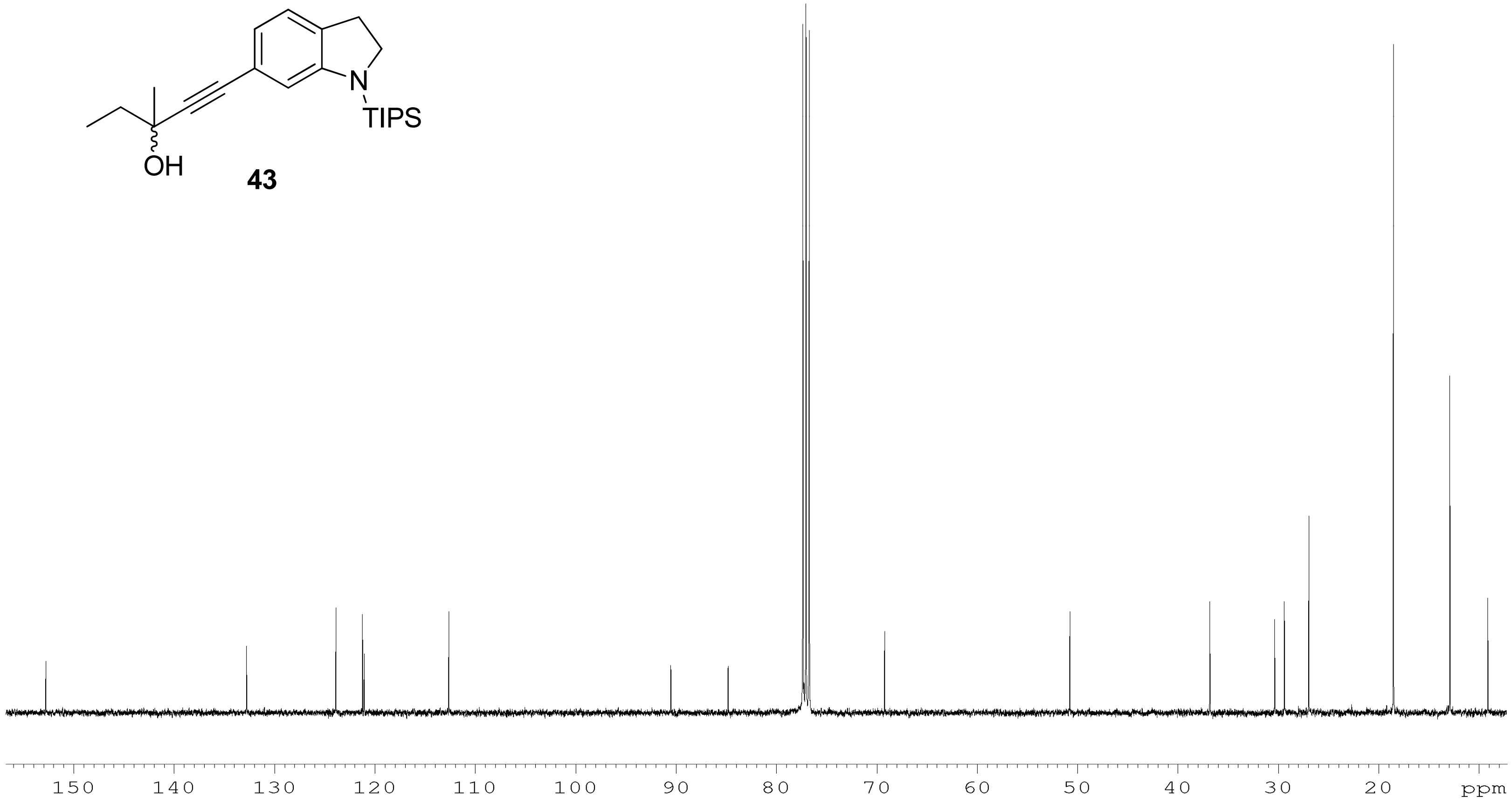


**43**

$^{1}$H NMR (200 MHz, acetone-$d_6$)

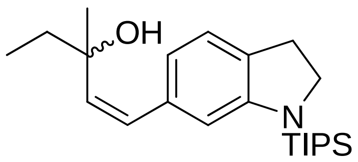


**44**

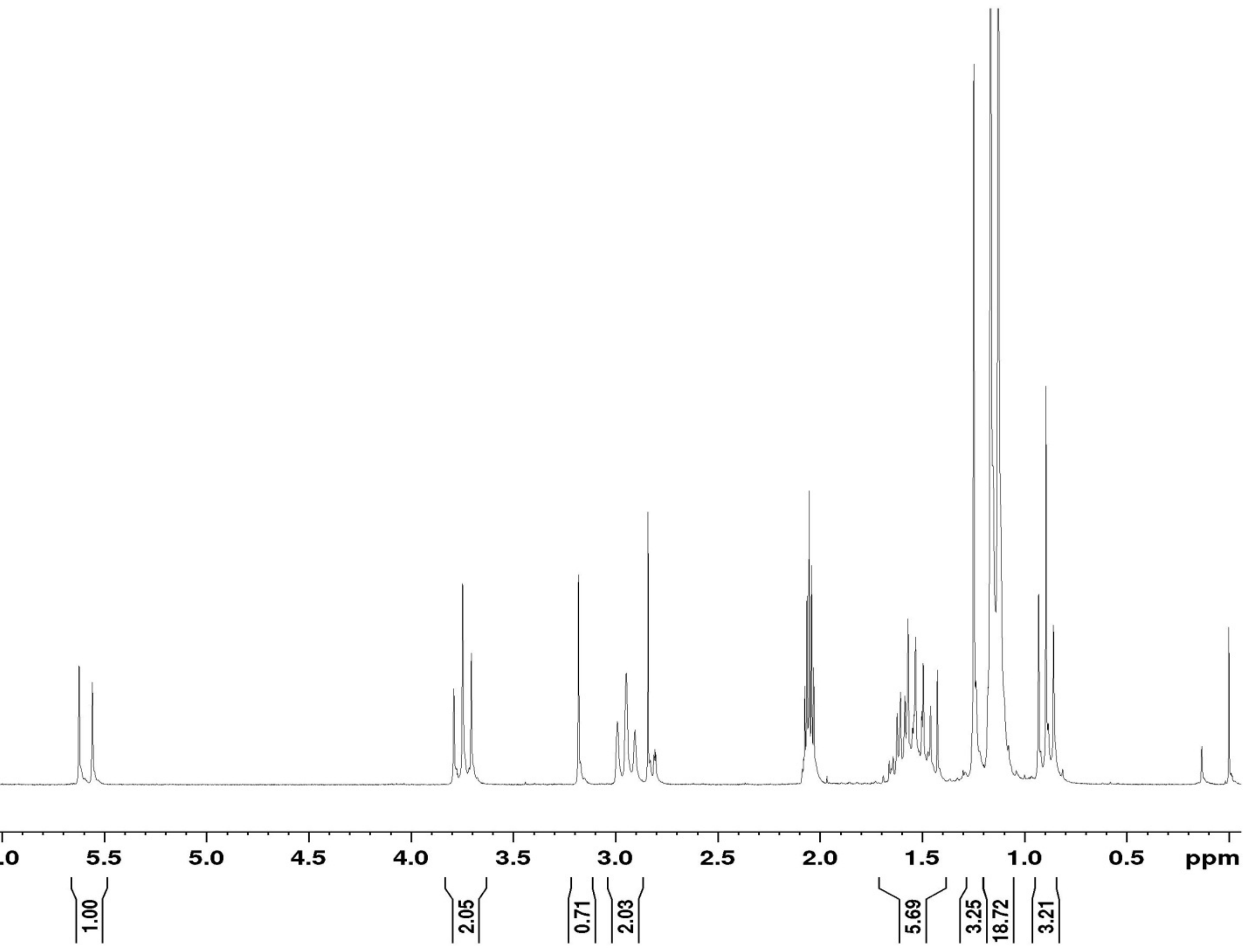

$^{13}C$ NMR (150 MHz, acetone-$d_6$)

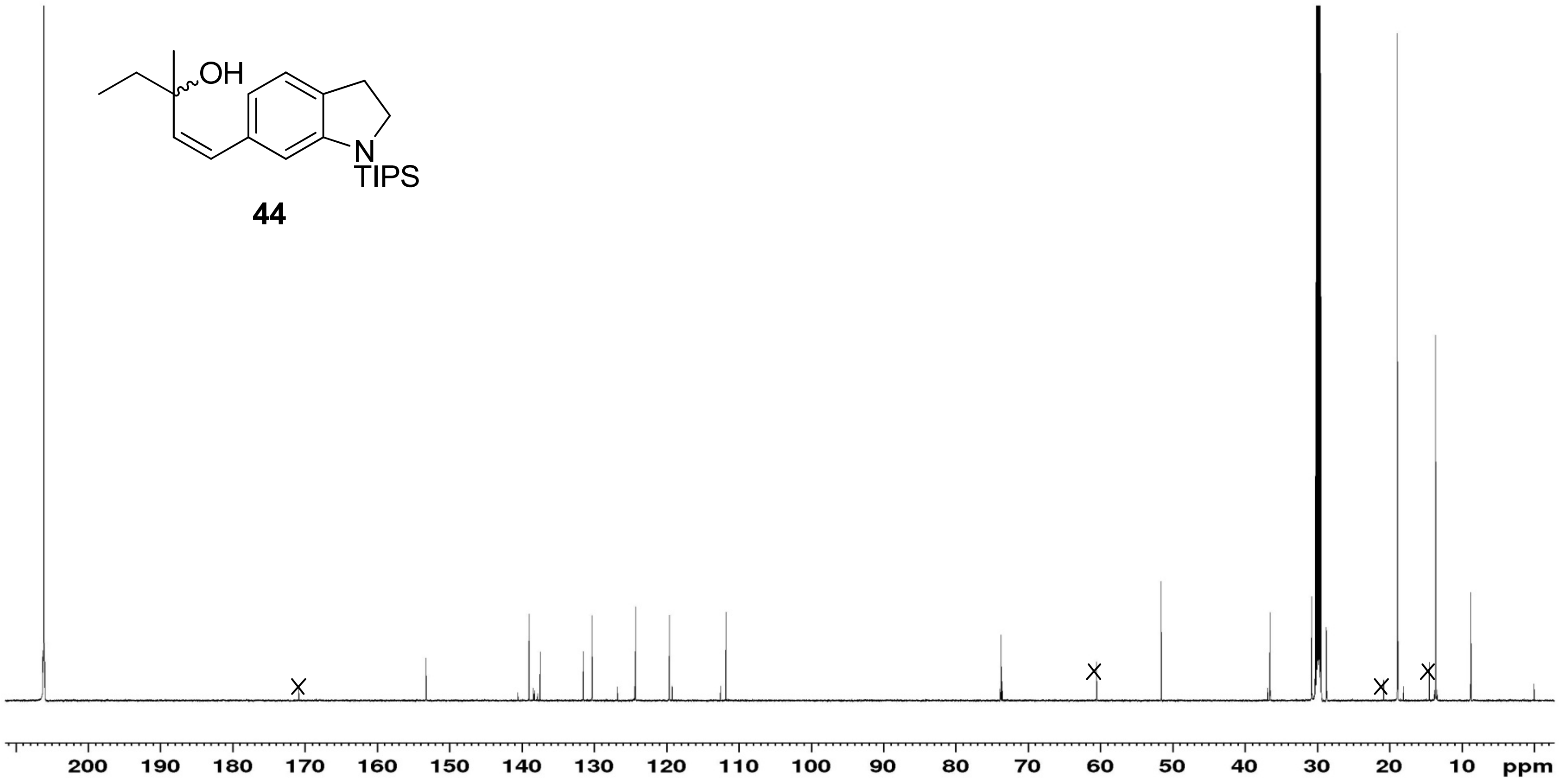

$^1H$ NMR (400 MHz, $CDCl_3$)

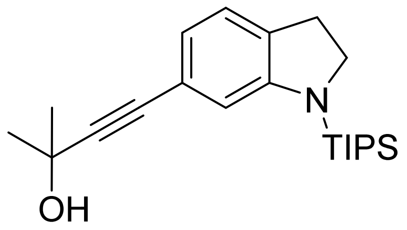


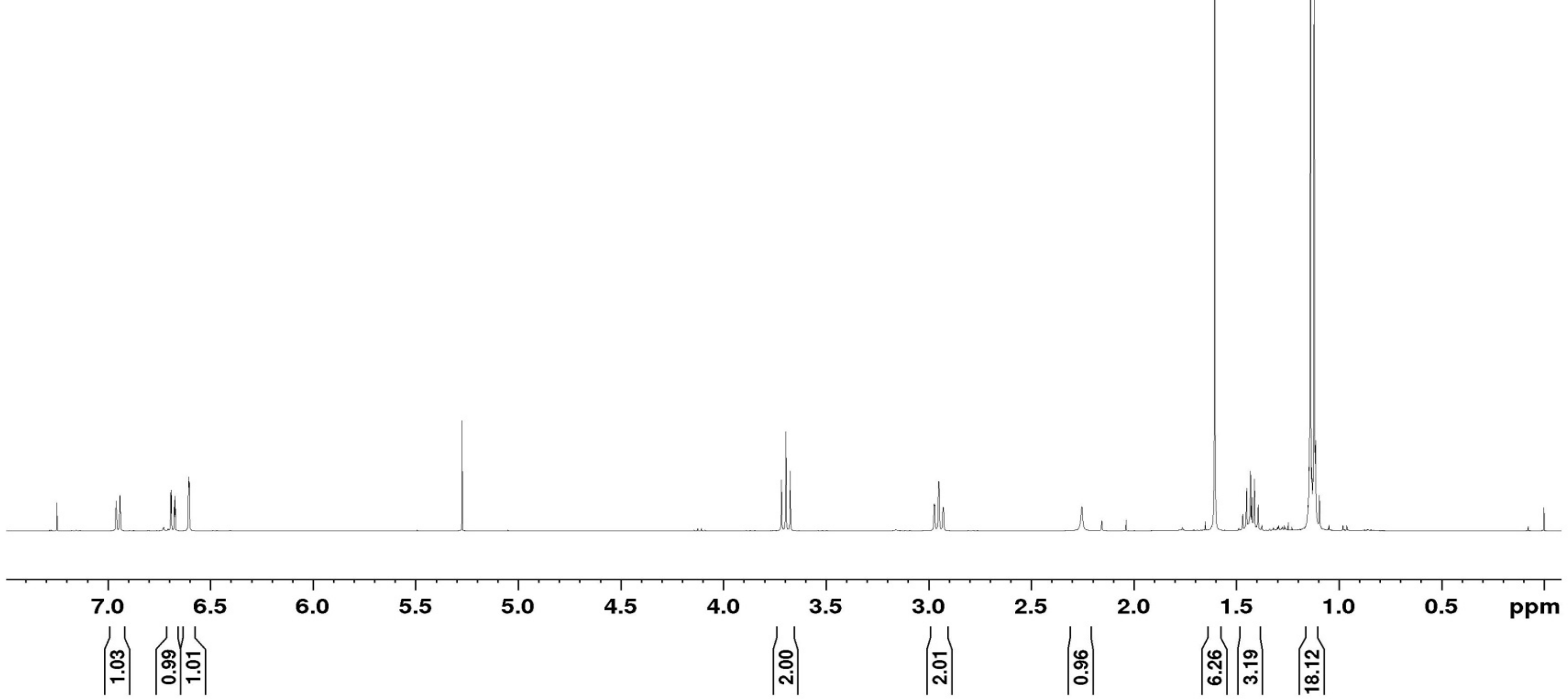

$^{13}C$ NMR (100 MHz, $CDCl_3$)

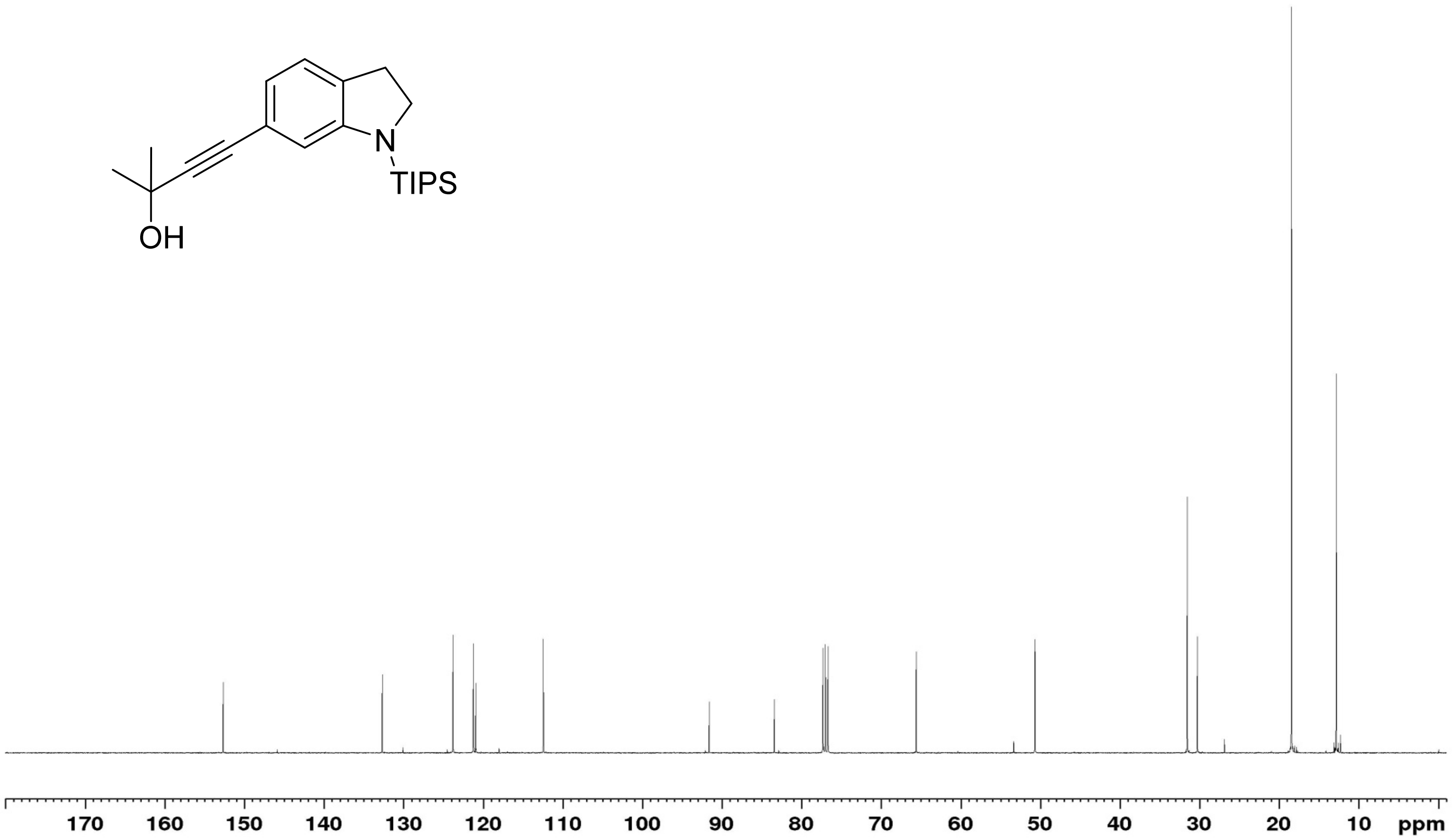

$^1$H NMR (400 MHz, $CDCl_3$)

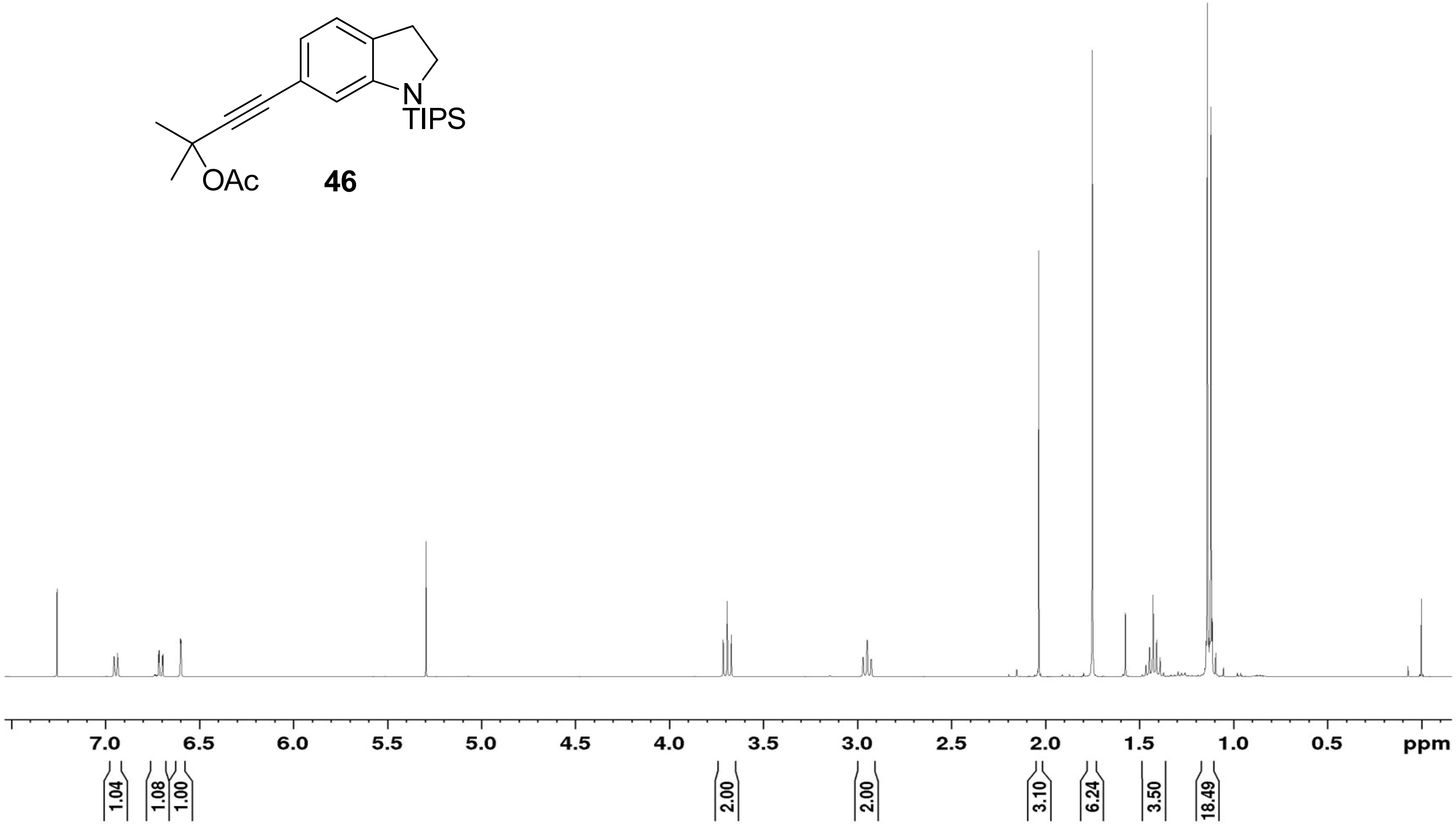

$^{13}C$ NMR (100 MHz, $CDCl_3$)

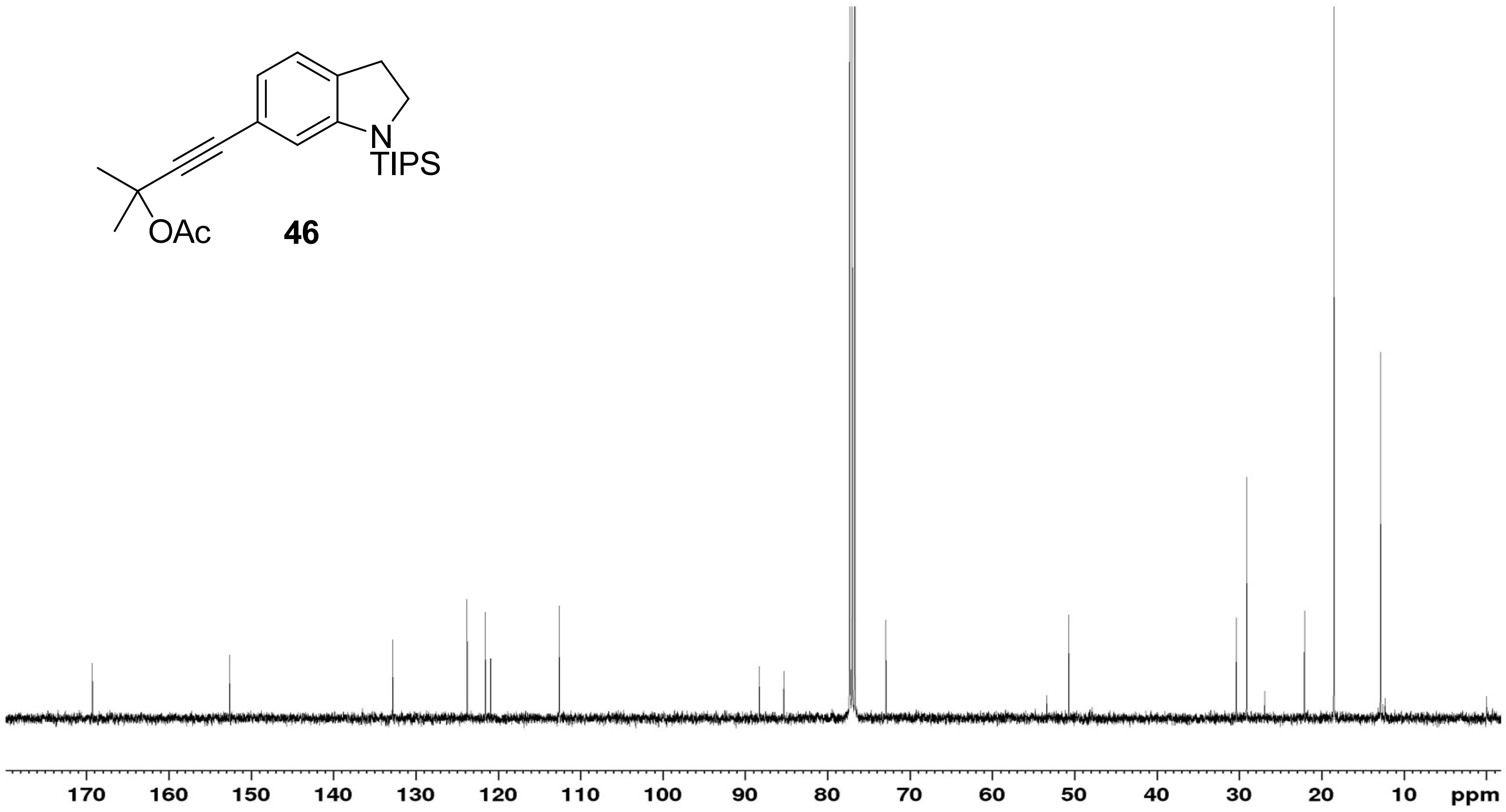

$^1$H NMR (400 MHz, $CDCl_3$)

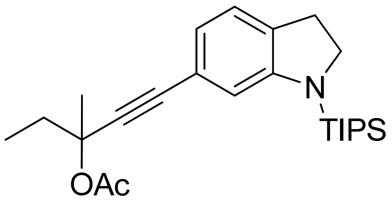


**47**

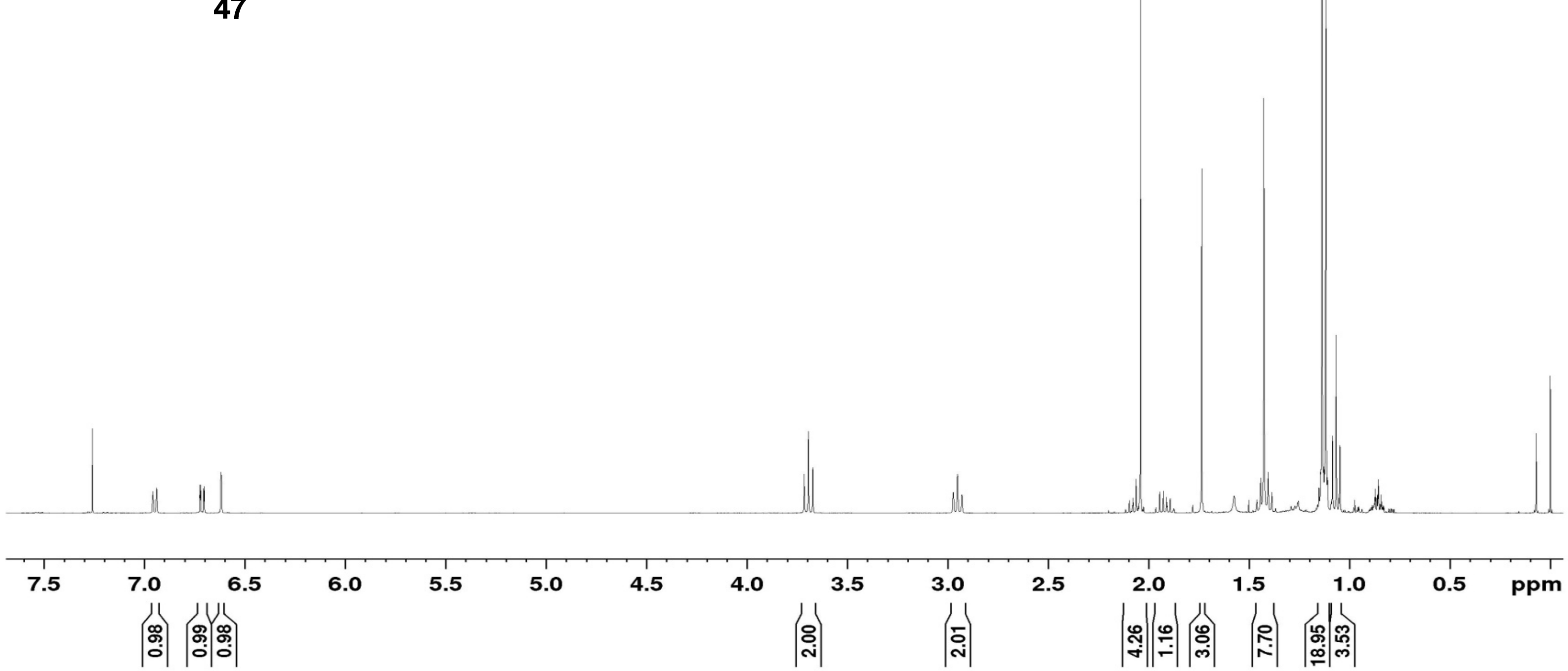

$^{13}C$ NMR (100 MHz, $CDCl_3$)

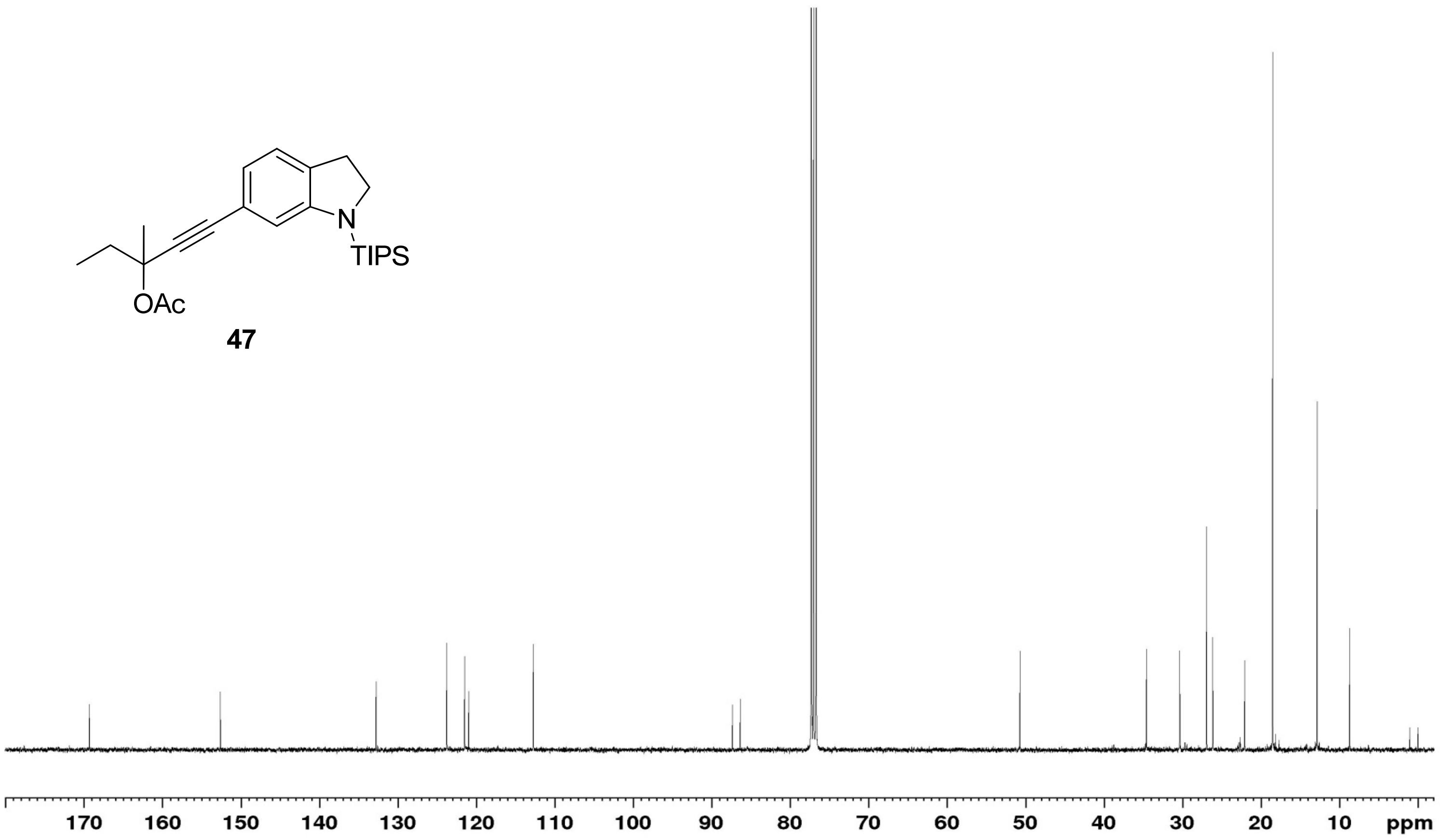


**47**

$^{1}H$ NMR (400 MHz, $CDCl_3$)

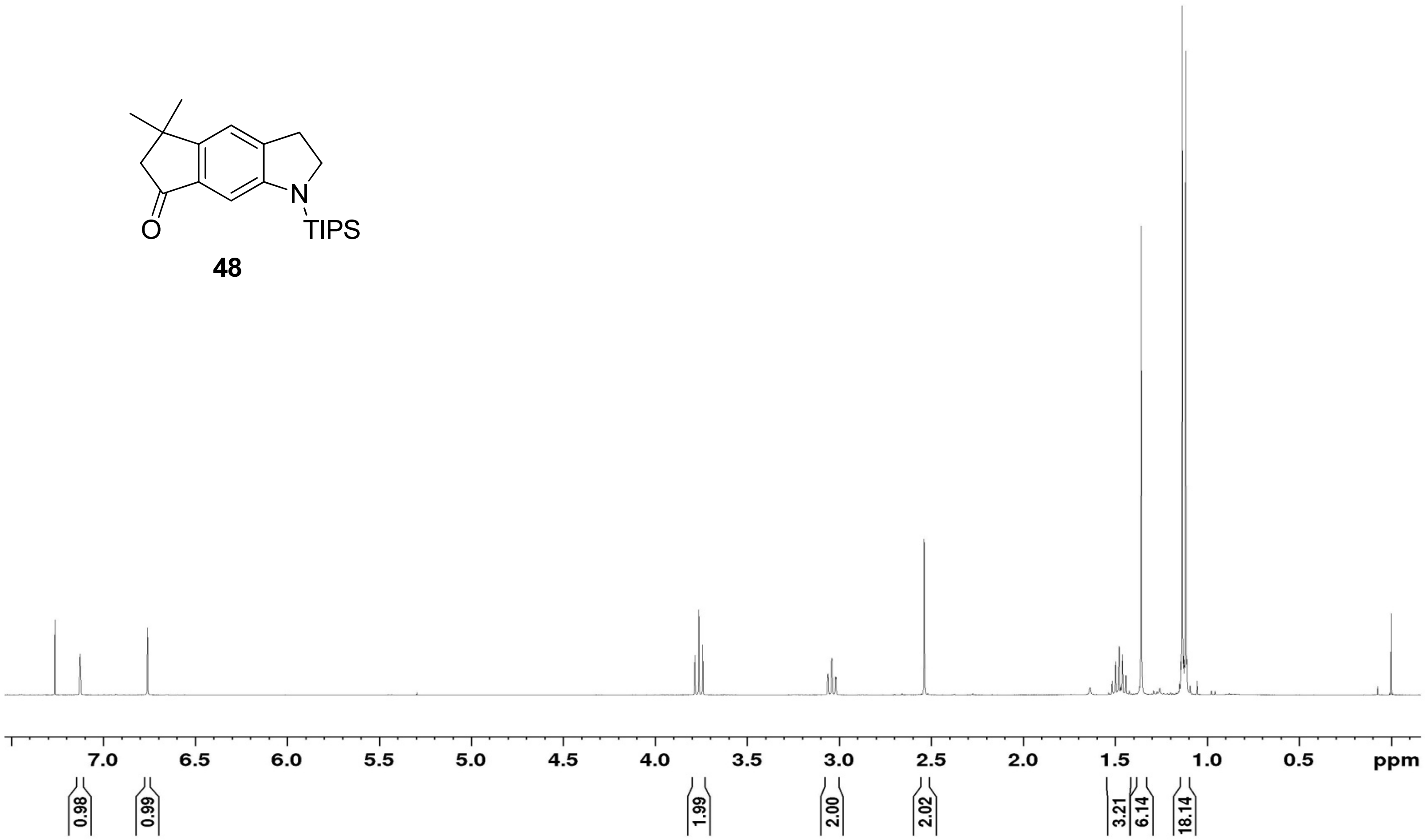


**48**

$^{13}C$ NMR (100 MHz, $CDCl_3$)

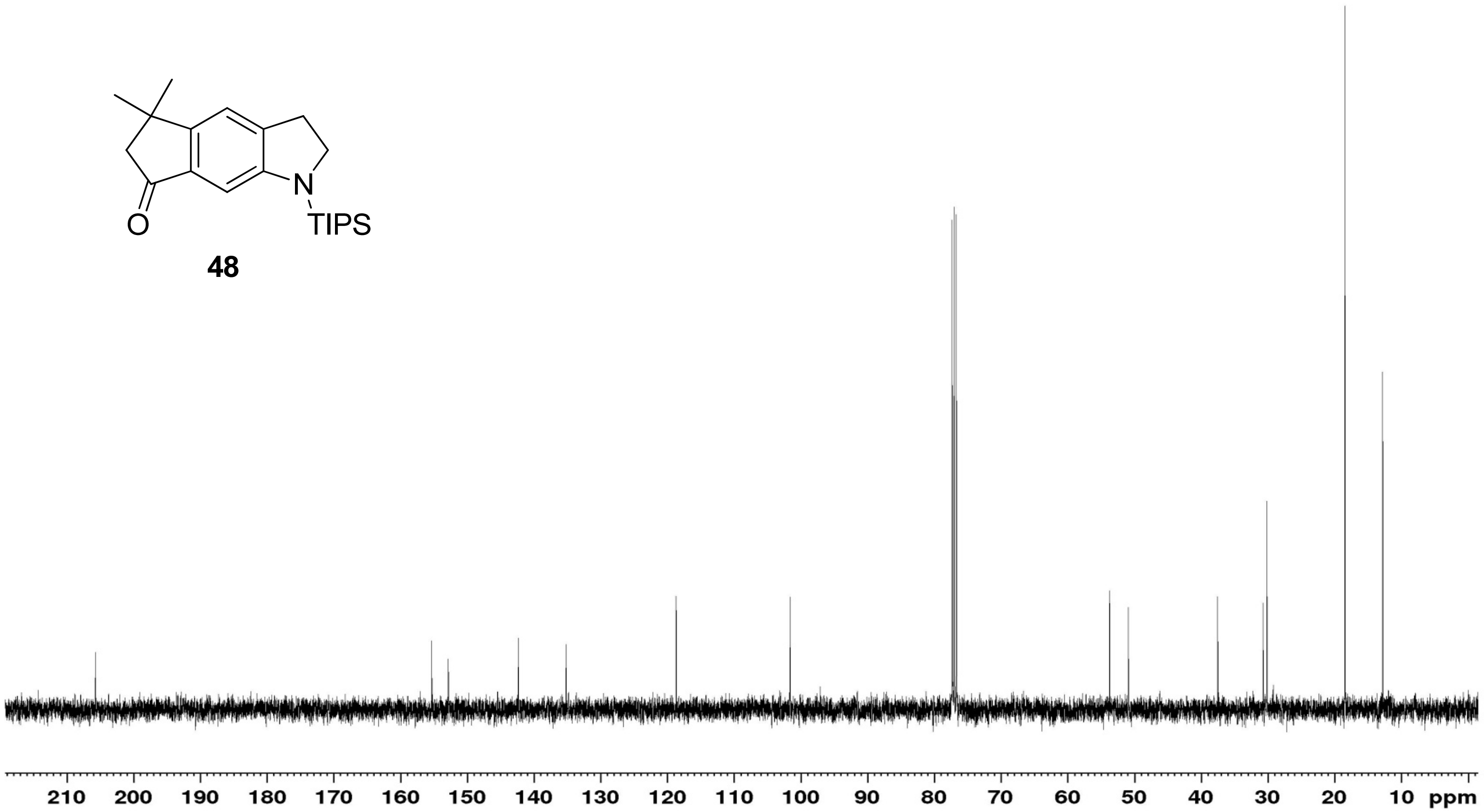

$^{1}H$ NMR (400 MHz, $CDCl_3$)

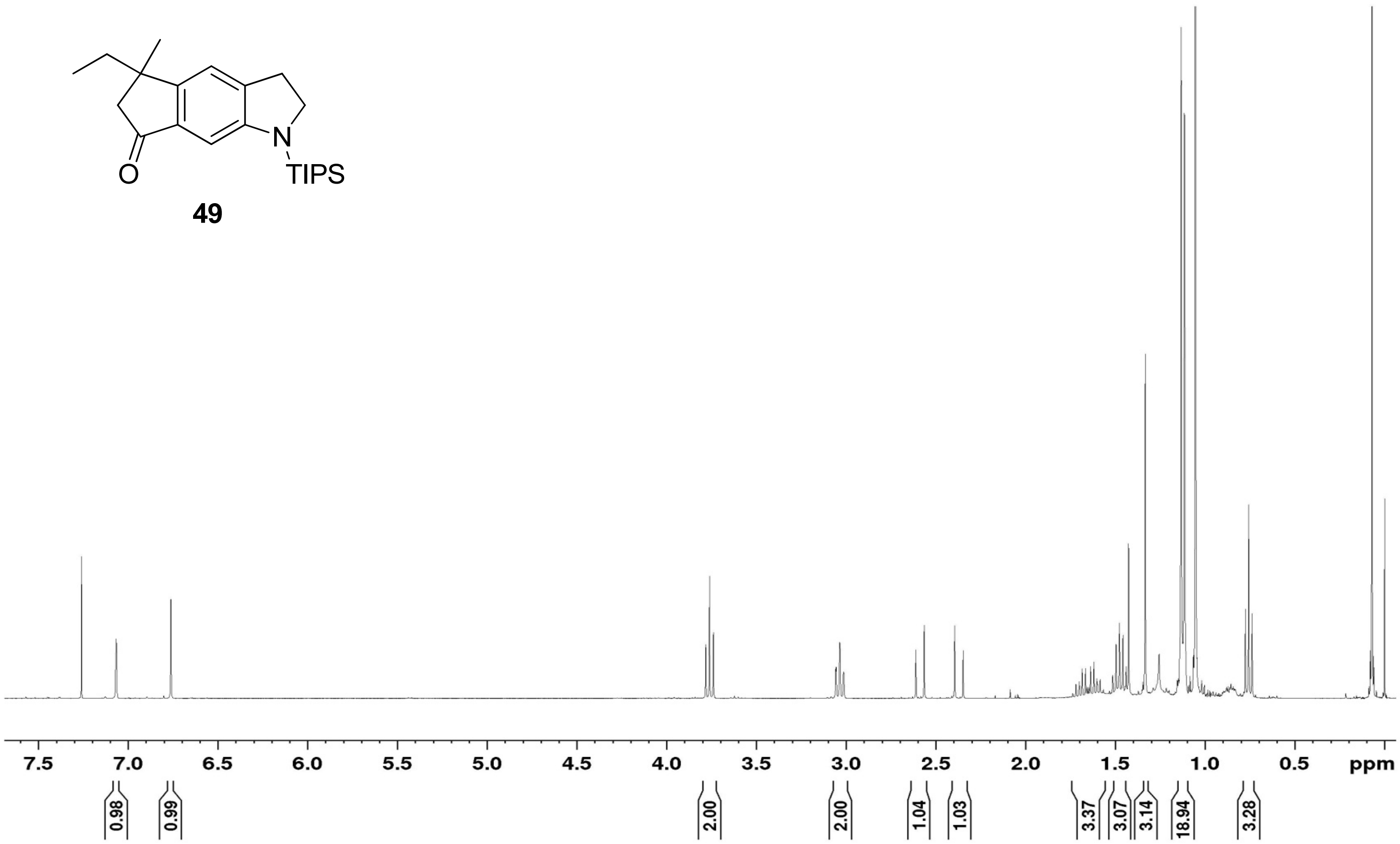

$^{13}$C NMR (100 MHz, $CDCl_3$)

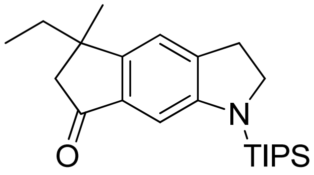


**49**

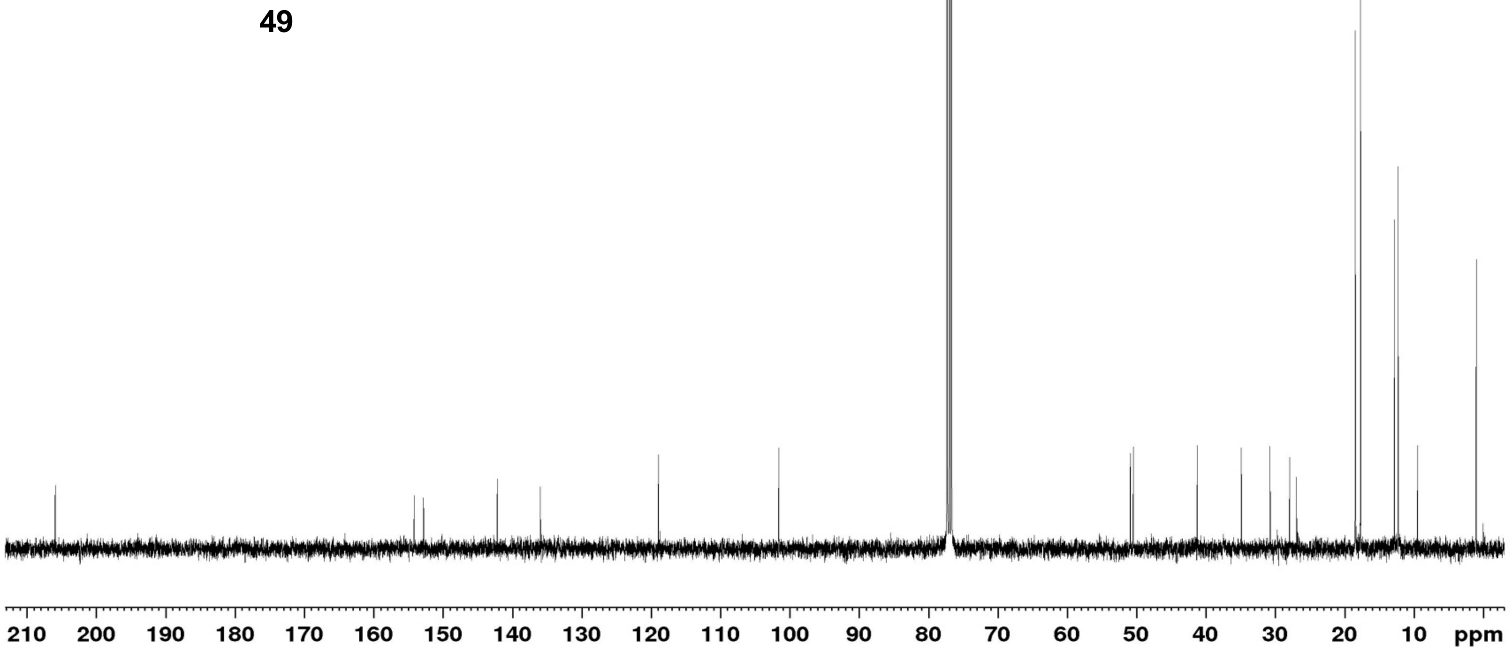

$^{1}H$ NMR (400 MHz, $CDCl_3$)

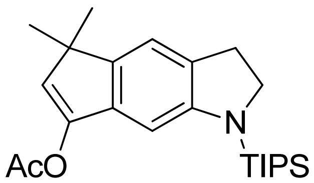


**50**

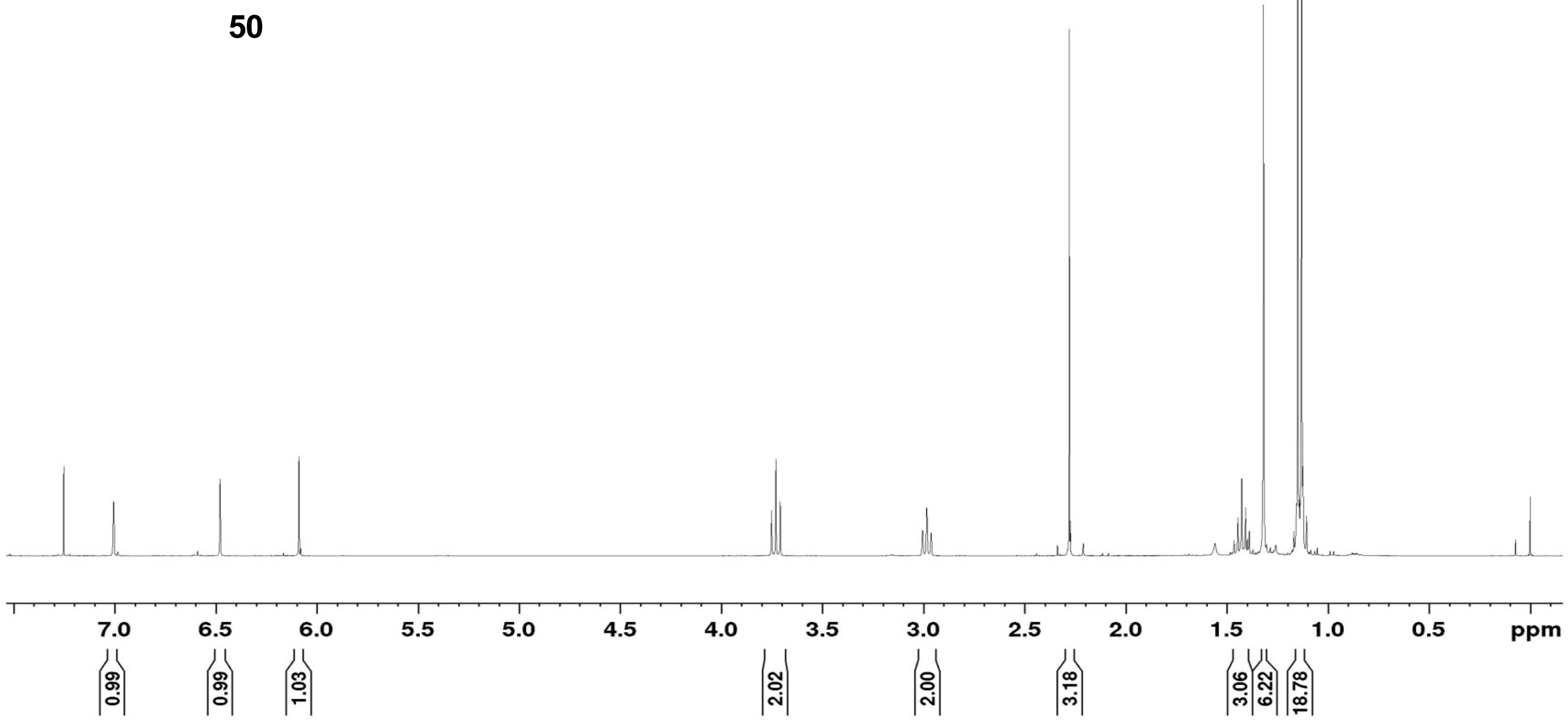

$^{13}C$ NMR (100 MHz, $CDCl_3$)

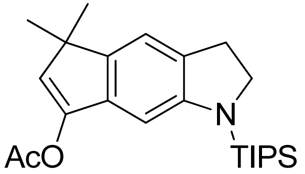


**50**

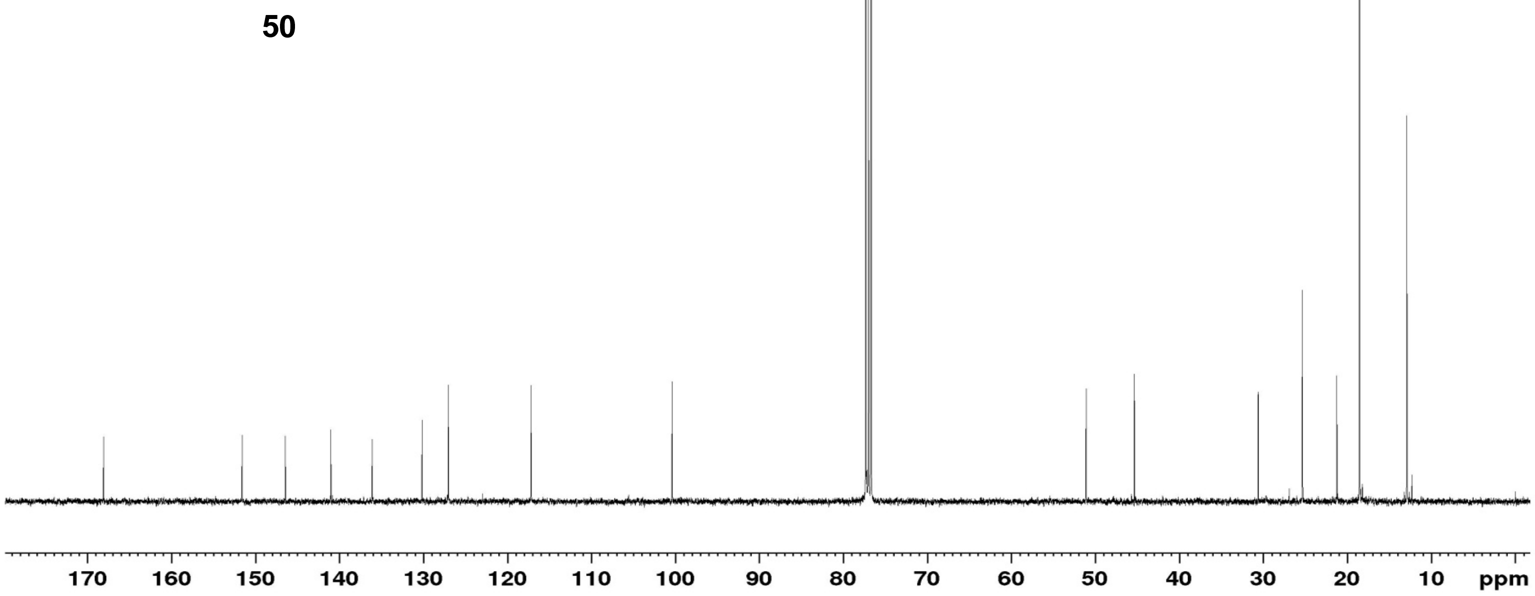

$^{1}$H NMR (400 MHz, $CDCl_3$)

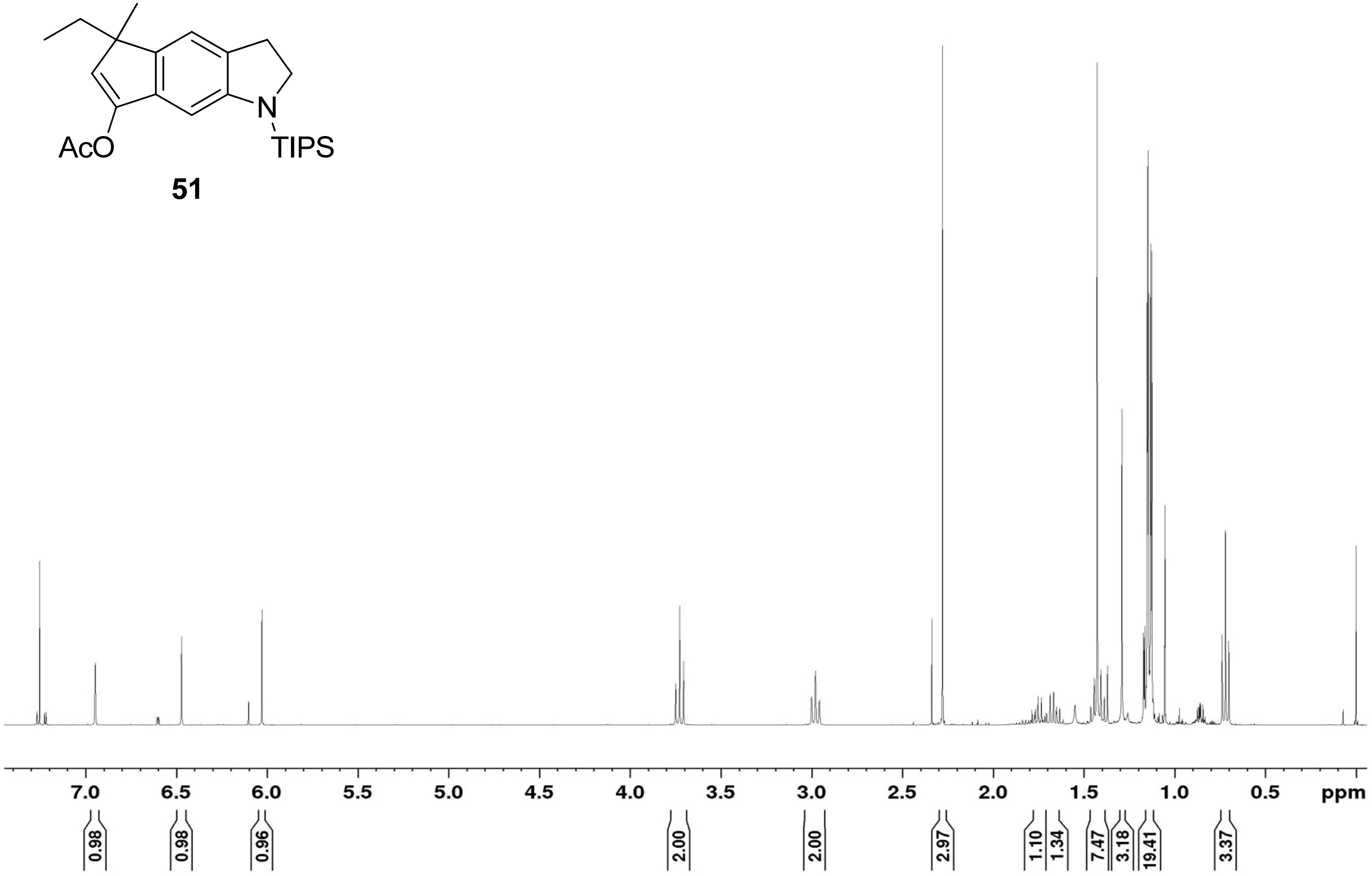

$^{13}C$ NMR (100 MHz, $CDCl_3$)

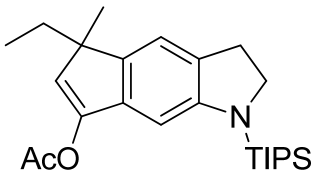


**51**

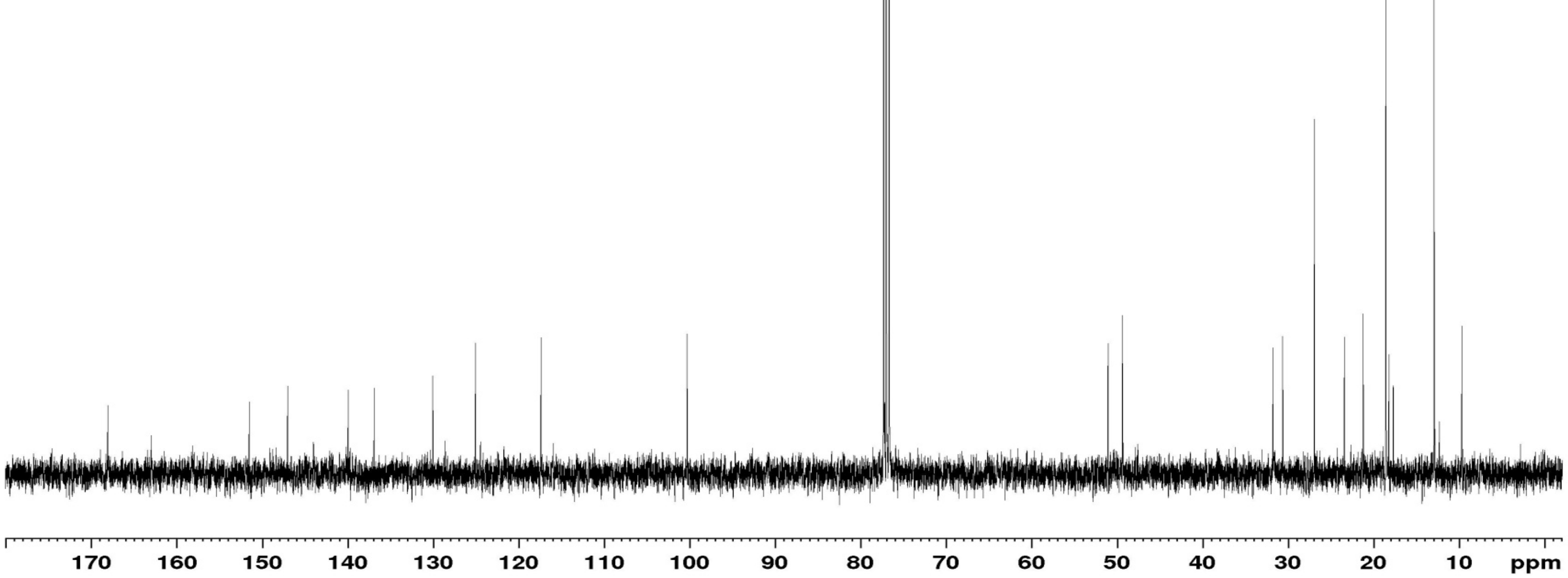

Supplement: File 1 — Experimental procedures and NMR spectra. [file Beilstein_J_Org_Chem-12-334-s001.pdf]
